# Studies on the Effect of Positive and Negative Charges on the ^77^Se NMR Shifts of Selenones and Selenenyls of N-heterocyclic Carbenes of Imidazolium-4,5-dicarboxylates

**DOI:** 10.1021/acs.joc.4c02581

**Published:** 2025-02-04

**Authors:** Lucas Pruschinski, Sean Ray Kahnert, Colin Herzberger, Jan C. Namyslo, Andreas Schmidt

**Affiliations:** Institute of Organic Chemistry, Clausthal University of Technology, Leibnizstraße 6, 38678 Clausthal-Zellerfeld, Germany

## Abstract

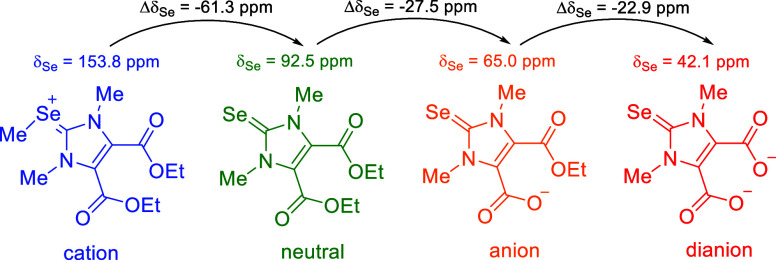

1,3-Imidazole-4,5-dicarboxylic acid derivatives were investigated with respect to the properties of the underlying N-heterocyclic carbenes. Their selenium adducts were prepared as diesters, monoesters, and dianionic dicarboxylates. Se-methylation yielded selenenyls (cationic selenoethers). ^77^Se NMR shifts of selenium adducts are considered a measure of the electronic properties of the underlying N-heterocyclic carbene. Since they are very sensitive to external (solvent, pH, temperature, reference reagent, reference method) and internal parameters (substituent effects, steric effects, nonclassical H-bonding, anisotropy), difficulties can arise in reliably interpreting these values. Our model compounds allow the systematic investigation of the influence of charges on the ^77^Se NMR shifts since both structural changes and changes of the measurement conditions have been minimized. Depending on the charge, the ^77^Se NMR shifts cover the range from 42.1 ppm (dianionic dicarboxylate) via 65.0 ppm (monoanionic dicarboxylate) and 92.5 ppm (neutral diester) to 153.8 ppm (cationic selenoether) in DMSO-*d*_6_. The signals also show considerable solvent and pH dependence, which was investigated with a selection of NMR solvents (MeOD, CDCl_3_, CD_2_Cl_2_, CD_3_COOD, CD_3_CN, acetone-d_6_, THF-*d*_8_, pyridine-d_5_, toluene-d_8_, DMSO-*d*_6_) and pH values in D_2_O. Linear relationships were found between the HOMO and LUMO energies and the ^77^Se NMR shifts, respectively.

## Introduction

**Scheme 1:**
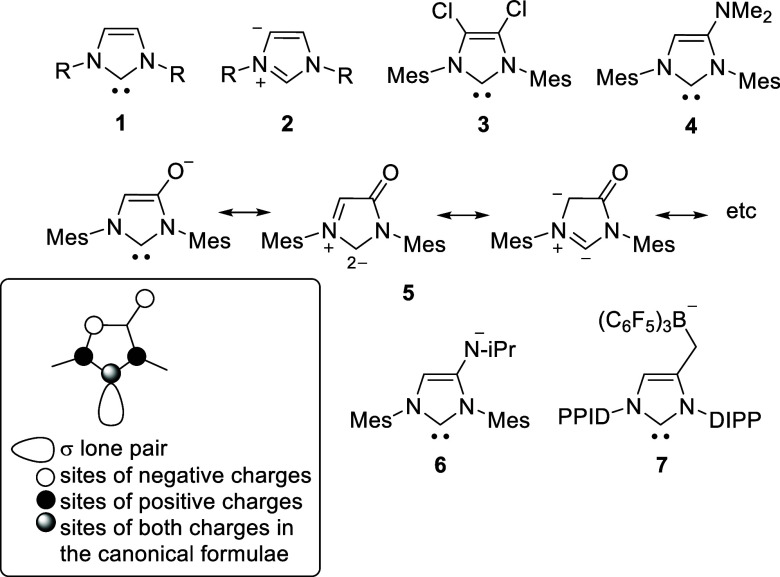
N-Heterocyclic Carbenes of Imidazole

N-Heterocyclic carbenes have become an integral part of modern chemistry^1^ and in the course of progressive research in recent decades, a whole series of different heterocyclic ring systems have been converted into N-heterocyclic carbenes. These include benzimidazoles,^2^ 1,2,3-triazoles,^3^ 1,2,4-triazoles,^4^ pyrazoles,^5^ 1,3-oxazoles,^6^ indazoles,^7^ sydnones^8^ and other 5-membered heterocycles as well as their benzoannelated derivatives. However, the most intensively studied N-heterocyclic carbene and the one often used in metal-catalyzed reactions is possibly the classical N-heterocyclic carbene of imidazole **1**, imidazol-2-ylidene, which finally set the impressive development of carbene chemistry in motion with its first successful isolation by Arduengo (R = adamantyl) (Scheme 1).^9^ This NHC must be distinguished from the anomalous N-heterocyclic carbenes of the same ring system, imidazol-4-ylidene **2**.^10^ Many substitution patterns of the NHCs of imidazol-2-ylidene **1** have since been synthesized, studied and described. These include those equipped with electron-withdrawing substituents such as **3**(11) or electron-pushing substituents such as **4**,^12^ which are conjugated to the carbene center.

**Scheme 2:**
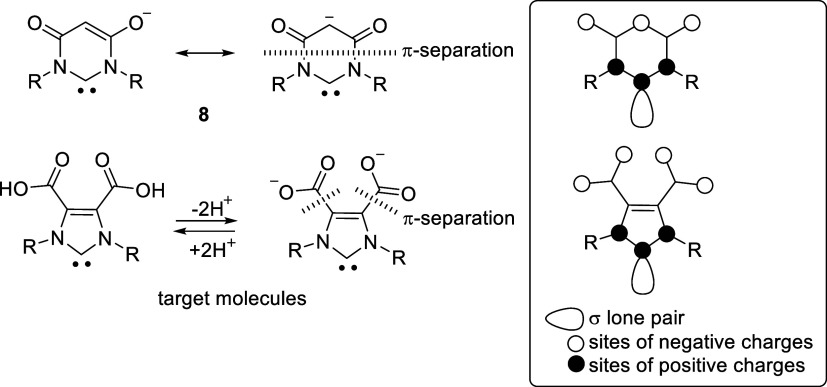
Cross-conjugated Anionic NHCs

Anionic N-heterocyclic carbenes of imidazole have been described, which carry an anionic substituent that can be in conjugation with the heteroaromatic center, as in **5**(13) and **6**,^14^ or not, as in **7**.^15^ Thus, the conjugated NHC **5** can be described by a series of canonical formulas to illustrate the influence of the negative charge according to the rules of mesomerism, some of which are shown in Scheme 1. Analogous mesomeric structures exist for the imidazolium-4-aminide **6**, but not for the carbene **7** which was generated from a nonconjugated zwitterion as carbene precursor. The charge distribution of **5**, **6**, and related N-heterocyclic carbenes with conjugated anionic substituents in position 4 or 5 according to the rules of mesomerism is shown in Scheme 1. Typical for conjugated systems are positions of both charges that coincide with the carbene carbon atoms.^16^ In contrast to carbenes **5** and **6**, which are conjugated, the pyrimidinium-based NHC **8**(17) is cross-conjugated and, according to the rules of mesomerism, has two separate π-systems in which the charges are delocalized (Scheme 2). If this type of conjugation is transferred to the 5-membered imidazole, derivatives of imidazolium-4,5-dicarboxylates can be designed, which are the target and test molecules of this report. Hitherto only one reference reports on adducts from the perspective of their biological and specifically agricultural activities.^18^

**Figure 1:**
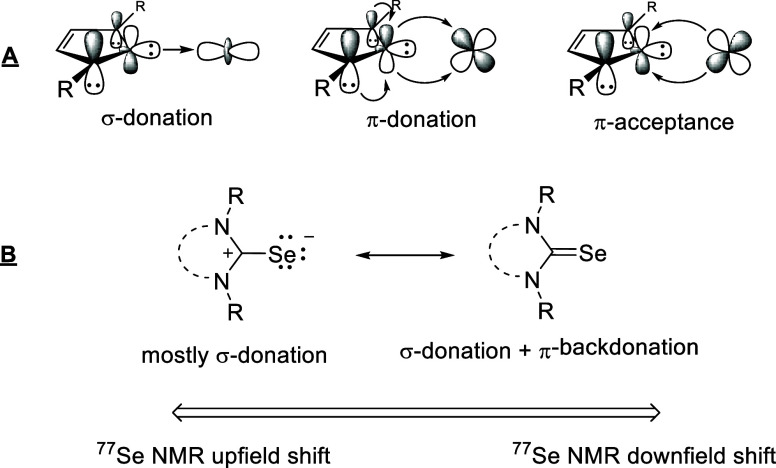
(A) π–Architecture and (B) idea of using ^77^Se NMR values as a measure of electronic interactions.

Carbene-metal bonds are characterized by the σ-donicity of the carbene on the one hand and their ability to form π-back bonds on the other,^19^ whereby π-donor properties of NHC ligands have also been described.^20^Figure 1A illustrates the orbitals involved in this binding situation.

**Figure 2:**
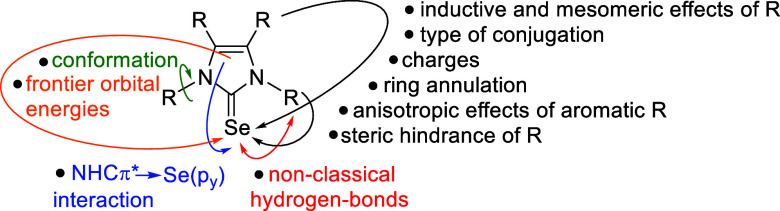
Some internal parameters which influence the ^77^Se NMR resonance frequency.

Distinguishing the σ- and π-portion of these bonds is not easy. Thus, the Tolman Electronic Parameter (TEP)^21^ tends to average over the total influence.^22^ Many methods are now available for characterizing the electronic properties of N-heterocyclic carbenes. In addition to the TEP values already mentioned, these include the Huynh Electronic Parameter (HEP),^23^ the ^31^P NMR shifts of the phosphinidene adducts of NHCs,^24^ the analysis of the ^1^*J*_CH_ coupling constants of the hetarenium salts from which NHCs can be generated with bases,^25^ the ^1^*J*_CSe_ coupling constants of the NHC selenium adducts^25^ and a number of other methods, which are summarized comparatively in review articles.^26^ The ^77^Se NMR chemical shifts of the selenium adducts of N-heterocyclic carbenes, also called selenones, selons or (when urea increments are present) selenoureas, are considered to be indicators of the π-acceptor properties of the underlying NHC.^27^ The basic idea is described by the two mesomeric structures in Figure 1B: The less π-backdonation, the more highfield shifted the ^77^Se NMR resonance frequency, since the dipolar notation is given more weight. However, ^77^Se NMR shifts are very sensitive to many influences, the overall effect of which cannot be determined by simply adding up the individual effects; additivity of the substituent effects is often not observable. This fact makes the interpretation of the ^77^Se NMR shifts with respect to the classification of electronic effects or with respect to a ranking of potential carbene structures with increasing or decreasing donor or acceptor properties as ligands very difficult, often even contradictory and counterintuitive. For example, the chemical shift of ^77^Se NMR is determined by the underlying heterocyclic ring system, although substituent effects such as the σ values of Hammett^28^ or the σ* values of Taft^29^ are not additive even within a series of the same heterocyclic system.^22a,30^ The steric requirement of tert-butyl groups and others leads to nonclassical hydrogen bonds via the free electron pairs of the selenium and σ*_CH_ - orbitals,^31^ which, like the binding of electrophiles to the selenium atom,^32^ leads to significant downfield shifts of the ^77^Se NMR signals. In addition, anisotropy effects and, related to this, the conformation of the substituents^22b^ and interactions of the π* orbital of the NHC with the Se(p_*y*_) orbital take influence.^33^ Apart from these internal parameters, which affect the value of the ^77^Se NMR chemical shift, external parameters such as the NMR solvent,^25,34−36^ the referencing method,^30,36−39^ the temperature,^36,37,40,41^ the concentration of the reference reagent^34,42^ and, in the case of internal referencing, even interactions between the reference reagent and the substance to be measured^36^ influence these values. These external parameters can add up to over 80 ppm and the influences of the different substituents to over 130 ppm.^36^Figure 2 shows a selection of different factors that can influence the ^77^Se NMR resonance frequencies, making it often impossible to reliably interpret the electronic properties of the underlying N-heterocyclic carbene. A review article has been written on this subject.^35^

In order to investigate individual parameters that influence the ^77^Se NMR resonance frequencies, it is necessary to rigorously restrict the variables while minimizing changes in the substitution patterns. This makes the imidazole-4,5-dicarboxylic acid derivatives shown in Scheme 2 ideal systems for investigating the influence of charges on the ^77^Se NMR shifts. This is because the esters in positions 4 and 5 can be saponified or partially saponified and thus provide anions or dianions from a neutral starting material. In this way, the influence of charges can be studied without changing the heterocyclic ring system, with chemically closely related substituents and without significant changes in steric hindrance. Nonclassical hydrogen bond formation is also excluded, and the retention of substituents on the ring prevents conformational influences as well as anisotropy effects as they are not aromatic. Even the conjugation type remains constant, namely cross-conjugated. Except for the charges that influence the frontier orbital energies, factors influencing the ^77^Se NMR shift are therefore minimized. The contribution of the charge to the ^77^Se NMR shifts can be clearly identified in these model molecules. Continuing our interest in the chemistry of mesomeric betaines,^43^ their overlap with the class of N-heterocyclic carbenes^44^ and Se chemistry,^45^ we report on our results here.

## Results and Discussion

### Syntheses

**Scheme 3:**
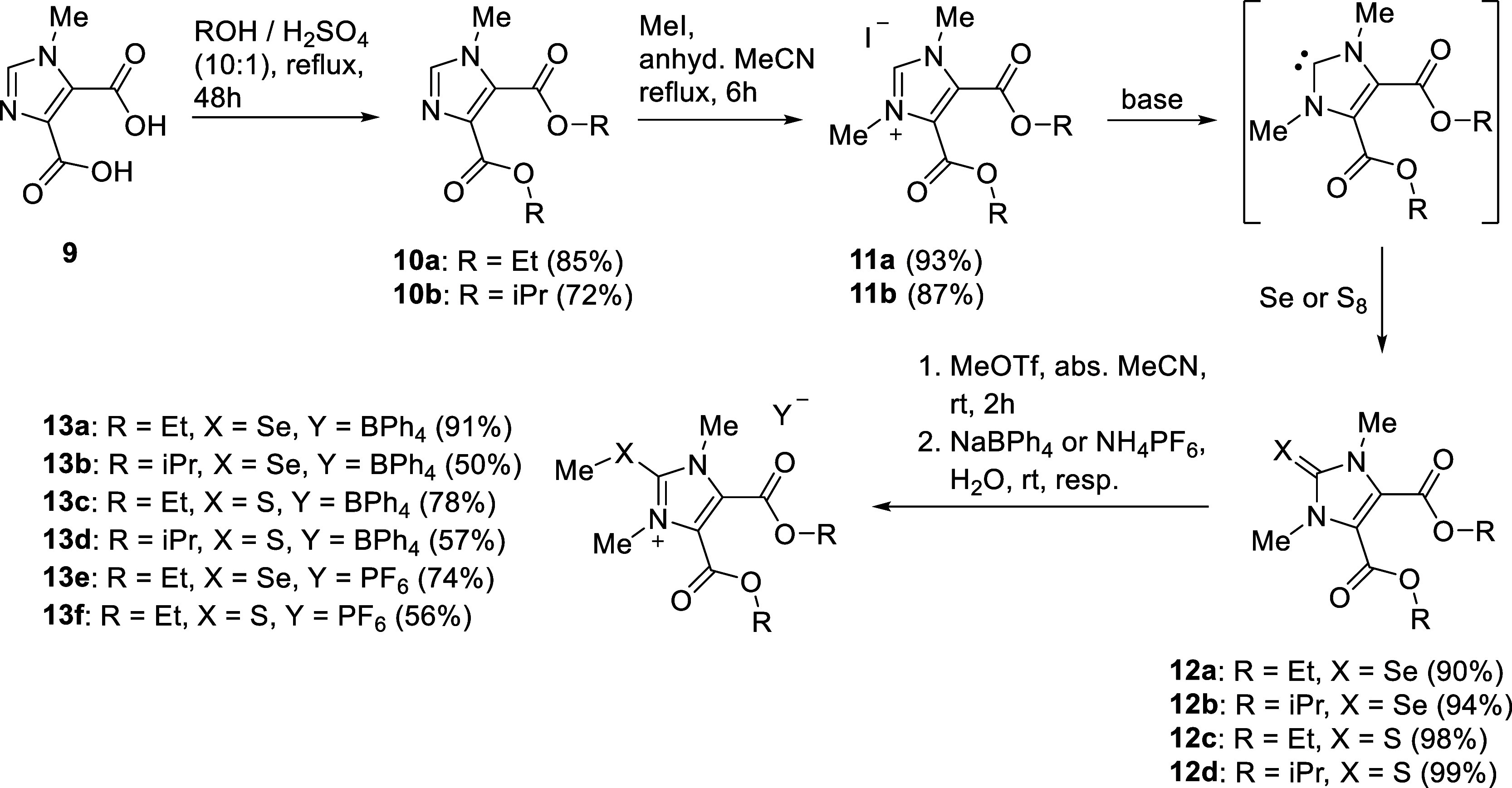
Synthesis of Thiones and Selenones of Imidazol-4,5-dicarboxylic Acid and Their Selenoethers

1-Methylimidazole-4,5-dicarboxylic acid **9** was first converted into the corresponding diethyl or diisopropyl esters **10a**(46) and **10b** and finally reacted with methyliodide under inert gas atmosphere in anhydrous acetonitrile, whereupon the 1,3-dimethylimidazolium salts **11a,b** were formed in very good yields (Scheme 3). The carbene relative energy of formation values (CREF values)^47^ were introduced as a quantitative index to determine the tendency of hetarenium compounds and mesomeric betaines to form N-heterocyclic carbenes. The heterolytic cleavage of the C–H bond at the position of carbene formation, i.e., the 2- or 4-position in imidazole, involves the transfer of both bond electrons to a free σ orbital. The CREF values of the imidazolium salts **11a** and **11b** were calculated at the B3LYP/6-311++G** level in order to compare the results with literature values of other imidazoles. The CREF values of **11a** and **11b** are 0.411 and 0.412 and therefore differ only marginally from the values of 1,3-dimethylimidazolium (0.413) for the formation of imidazol-2-ylidenes. The substitution pattern is therefore not reflected in the CREF values here. The value of the abnormal N-heterocyclic carbene for the latter is 0.442. The ^1^*J*_CH_ heteronuclear coupling constant of azolium salts was considered as a measure of the σ-donicities of the underlying N-heterocyclic carbene and weaker donors should result in larger coupling constants.^25,26a^ We determined the corresponding coupling constant of **11a** to be 226.4 Hz in CDCl_3_ and 226.7 Hz in DMSO-d_6_. The values of the isopropyl derivative **11b** are 226.5 Hz (CDCl_3_) and 226.6 Hz (DMSO-d_6_), and these correspond approximately to those of the 1,3-DIPP imidazolium salt IPr·HBF_4_ (^1^*J*_CH_ = 225 Hz in DMSO-d_6_) or the 1,3-Mes-imidazolium salt IMes·HCl (^1^*J*_CH_ = 225 in CDCl_3_).^26a^ The thiones and selenourea derivatives **12a**–**d** were synthesized by treatment of the salts **11a,b** with the corresponding elements in anhydrous acetonitrile in the presence of cesium carbonate in very good to quantitative yields. The N-heterocylic carbene can be detected by NMR spectroscopy; its ^13^C{^1^H} NMR signal appears at δ = 189.7 ppm in CD_3_CN. Methyl trifluoromethylsulfonate converted **12a**–**d** into the Se- and S-ethers after aqueous anion exchange to tetraphenylborate and hexafluorophosphate, respectively. The imidazolium salts **13a**–**f** possessing exocyclic selenoether and thioether groups were thus obtained in medium to very good yields.

**Scheme 4:**
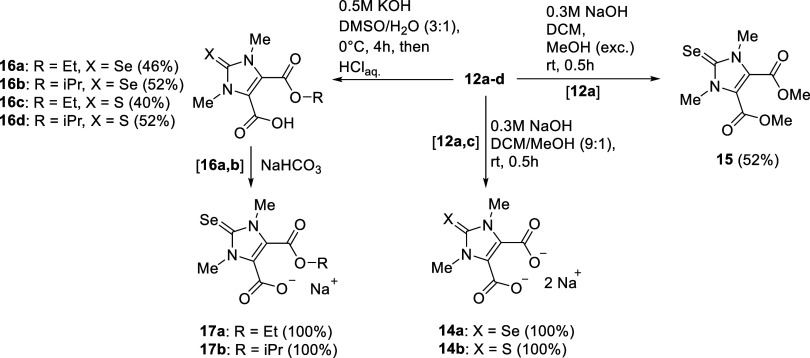
Transesterification and Saponification Reactions

The saponification of the ester functions of the selenium and sulfur adducts **12a**–**d** proved to be a challenge and required careful exploration of the reaction conditions in order to achieve any saponification at all without decomposing the molecule. Finally, the disodium dicarboxylates **14a,b** were formed from **12a,b** with 0.3 M sodium hydroxide solution in a mixture of dichloromethane (DCM) and methanol in a ratio of 9:1 at rt within only 30 min (Scheme 4). A concentrated aqueous solution of the disodium dicarboxylate **14a** has pH 7.5 (10 g/L) in agreement with its structure and in accordance with pH values of other dicarboxylates such as sodium oxalate (8.0; 30 g/L) or sodium succinate (8.6; 100 g/L). Higher proportions of methanol in the solvent mixture led to transesterification of the diethyl ester to the dimethyl ester **15** in medium yields. The saponification of only one of the two ester groups of **12a**–**d** was achieved with 0.5 M potassium hydroxide solution in a mixture of DMSO and water (3:1) under ice cooling. These reaction conditions yielded the imidazole-4,5-carboxylic acid monoesters **16a**–**d** after adjusting the pH to 5–6 by dilute hydrochloric acid. The proton of the carboxylic acid is visible between δ = 14.18 and 14.36 ppm in the ^1^H NMR spectra measured in DMSO-d_6_. The imidazole-4,5-carboxylic acid monoesters can be converted in quantitative yields into the corresponding mono sodium salts **17a,b** by adding aqueous NaHCO_3_ solution.

**Figure 3:**
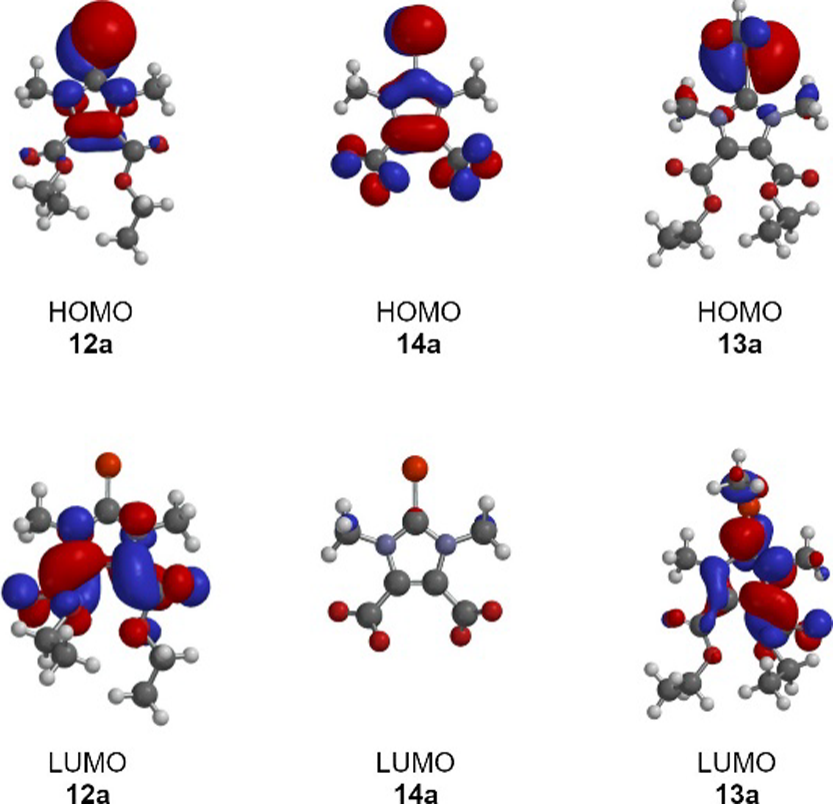
Selected HOMO/LUMO profiles of selected selenourea derivatives and a selenenyl.

DFT calculations [B3LYP 6-311++G**] were performed to visualize the highest occupied (HOMO) and lowest unoccupied molecular orbitals (LUMO) and calculate their energies. The frontier orbital profiles of **12a**, **13a**, and **14a** are shown in Figure 3, those of **12b**, **13b**, **16a,b**, and **17a,b** as well as of the NHCs of **12b**, **16a,b**, and **17a,b** are presented in the Supporting Information. As expected, the frontier orbitals of the diesters and monoesters differ architecturally only in the backbone of the selenourea derivatives. As an example, the frontier orbital profile of diester **12a** is shown in Figure 3. As a consequence of the negative charges, the dianion **14a** has considerable atomic orbital coefficients at the oxygen atoms of the carboxylate groups whose carbon atom is a nodal position. This is typical for cross-conjugated mesomeric betaines with these building blocks;^5c,7,16^ however, considerable atomic orbital coefficients are also found in the imidazole ring and at the selenium atom, where the selenium carbonyl carbon atom, in contrast to the HOMO of **12a**, is also an active position. This is also the case for all carbenes we have calculated (c.f. Supporting Information). When interpreting the ^77^Se-NMR shifts as a measure of the π-character of N-heterocyclic carbenes, orbitals with a nodal position, *i.e*. so-called inactive positions, at the carbon atom of the C=Se group are often not taken into account.^35^ The largest atomic orbital coefficients of the LUMO of **12a** are found in the maleate moiety, indicating strong electron-withdrawing properties, while the LUMO of the dianion **14a** shows only small atomic orbital coefficients. Indeed, the selenium atom with its large atomic orbital coefficients of the HOMO seems susceptible to electrophiles such as Me^+^. The HOMO of the cationic selenenyl **13** (R = Et) displays a different geometry of atomic orbital coefficients at the selenium atom, as expected. Its LUMO includes the carbonyl groups. A large atomic orbital coefficient of the LUMO is localized at the selenium atom. These unavoidable differences in the π-architectures are the caveat of our investigations, which aim to elucidate the influence of charges on the ^77^Se NMR shifts in the light of their interpretation as probes for the π-properties of the underlying NHCs, while excluding or minimizing the many parameters mentioned at the beginning.

### ^77^Se NMR Spectroscopy

**Figure 4:**
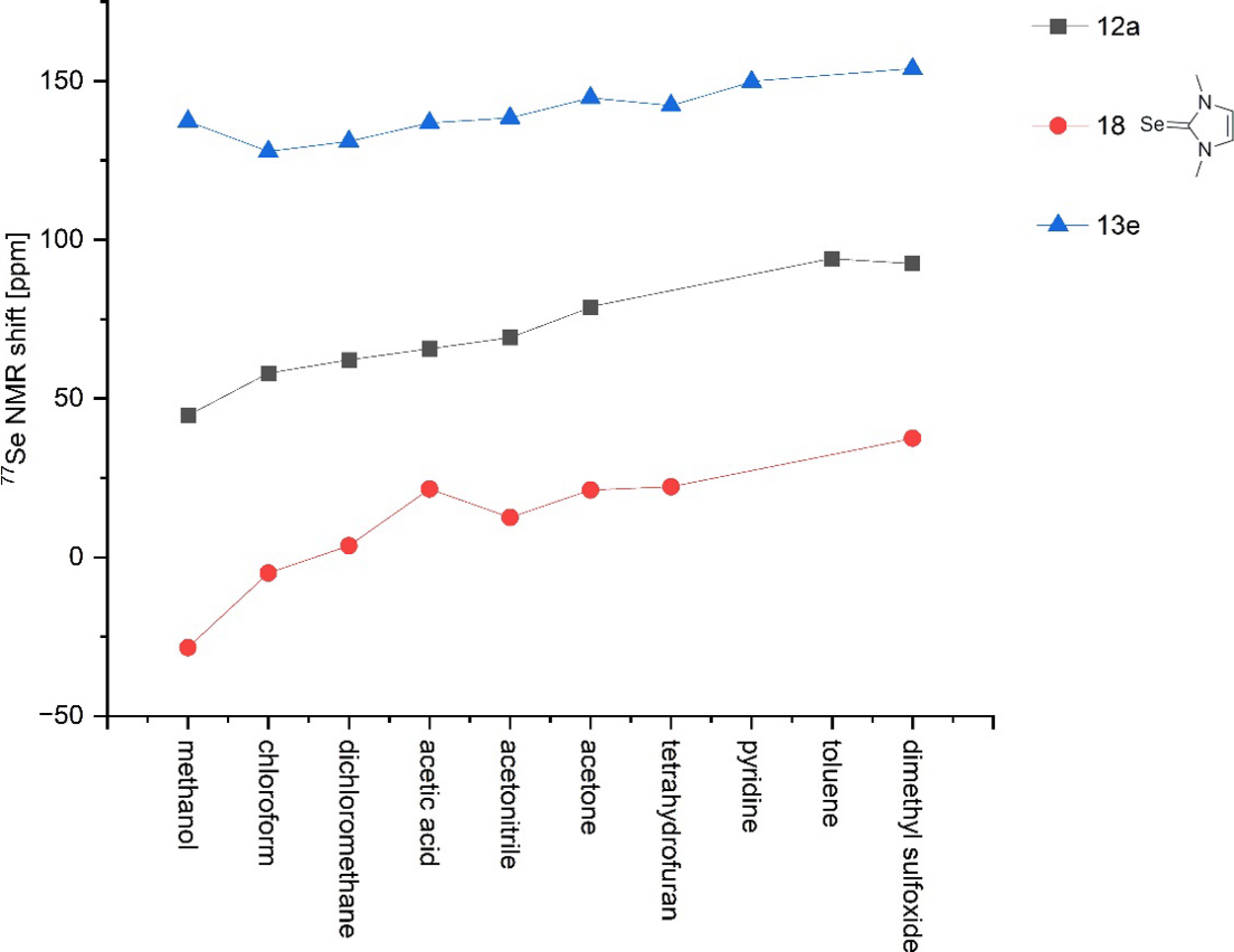
^77^Se NMR chemical shift changes in different solvents.

First, we examined the solvent dependence of the ^77^Se NMR shifts of selenone **12a**, selenenyl **13e** and, as comparison, 1,3-dimethylimidazole-2-selenone **18** under identical conditions. In all measurements, we used diphenyldiselenide as an external reference reagent at room temperature. Characteristic properties of this reagent as a reference substance are summarized in the literature.^35^ The results are shown in Figure 4 and the data are presented in Table 1. They show that the resonance frequencies for compound **12a** lie in a range of Δδ = 49.3 ppm depending on the NMR solvent. The solvent-dependent change in the ^77^Se shift of **18** is slightly more pronounced and covers a shift range of Δδ = 66.0 ppm, while the solvent dependence is less pronounced for the selenenyl compound **13e**, where the values vary over a range of Δδ = 26 ppm. The approximate parallelism of the shifts is striking, although the ranking of the solvents does not follow simple solvent parameters. This ranking does not correlate with empirical polarity scales for solvents, dielectric constants, or other scales,^48−50^ and differs from the ranking for acetic acid in the case of related pyrrolidines,^36^ which, in contrast to the cross-conjugated compounds studied here, are conjugated.

**Figure 5:**
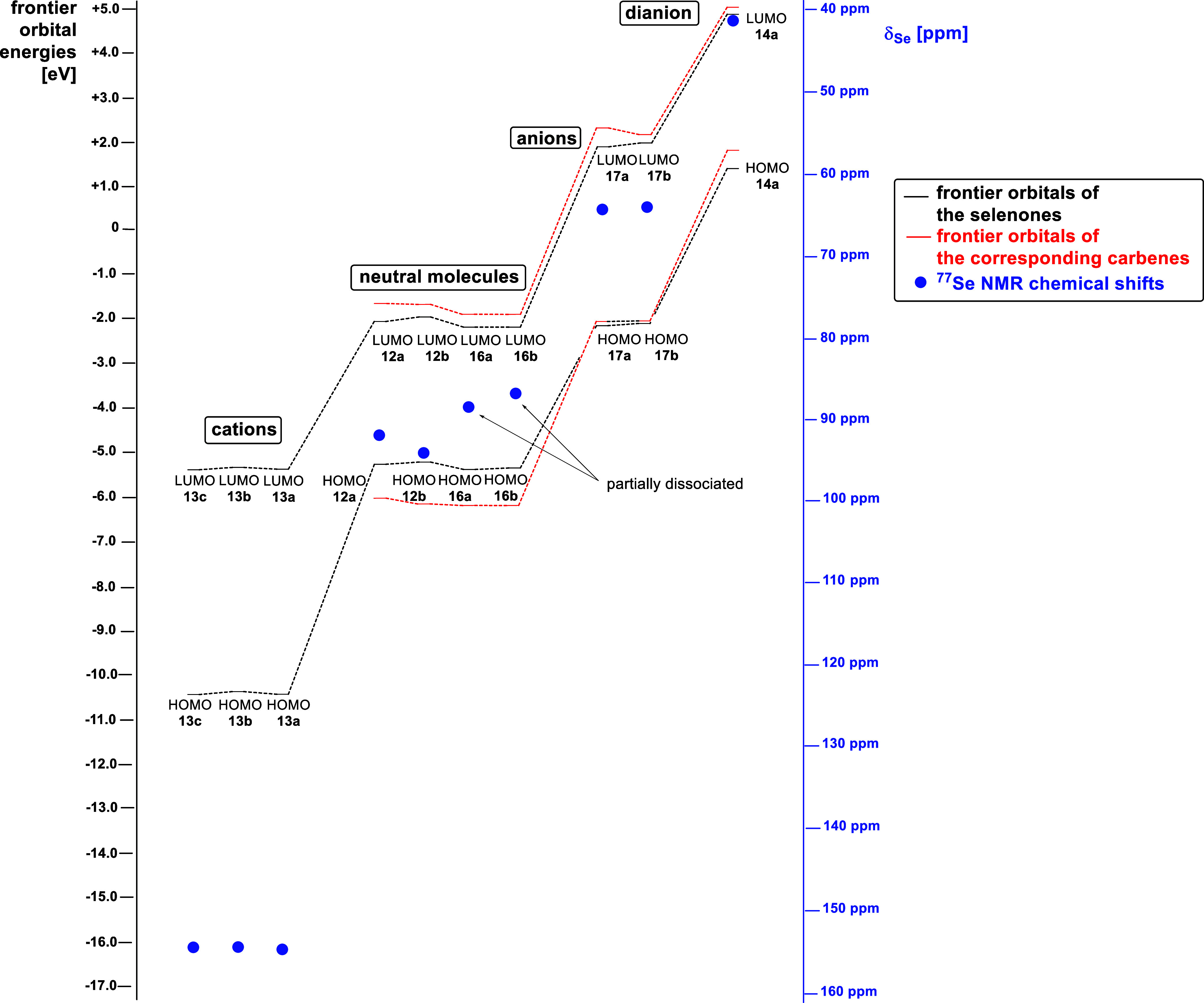
HOMO/LUMO energies and ^77^Se NMR shifts.

**Table 1:** NMR Shifts of **12a**, **13e**, **18** in Different NMR Solvents

NMR solvent	^77^Se NMR shift^a^ of 12a [ppm]	^77^Se NMR shift^a^ of 13e [ppm]	^77^Se NMR shift^a^ of 18 [ppm]
MeOD	44.7	137.3	–28.5
CDCl_3_	58.0	127.8	–5.0
CD_2_Cl_2_	62.2	131.0	3.6
CD_3_CN	69.2	138.4	12.5
acetone-d_6_	78.7	144.7	21.2
THF-d_8_		142.3	22.2
pyridine-d_5_		149.7	
toluene-d_8_	94.0		
DMSO-d_6_	92.5	153.8	37.5
AcOD	65.7	136.8	21.5

a External referenced to Ph–Se–Se–Ph @ 461.0 ppm rel. to SeMe_2_ @ 0.0 ppm.^38^

As mentioned before, our compounds allow the investigation of the influence of charges on ^77^Se NMR shifts because, by minimizing structural changes, most of the numerous other influencing factors mentioned above are ruled out. We therefore continued our investigations with the cationic, neutral, anionic and dianionic derivatives of imidazole-4,5-dicarboxylic acid derivatives described above. Their ^77^Se NMR shift values measured in DMSO-d_6_ are summarized in Table 2. Thus, the ^77^Se NMR shifts of the monoesters **16a,b** are at δ = 88.8 and 87.1 ppm, respectively, and are therefore slightly highfield shifted compared to the diesters **12a,b** (δ_Se_ = 92.5/90.6 ppm). The slight shift can be logically explained by a partial dissociation of the carboxylic acid group in DMSO-d_6_ with the formation of negatively charged carboxylate groups, especially since DMSO is a basic solvent with a value of Δδ_∞_ = 1.32.^41^ The signals of the acidic proton of the carboxylic acids **16a,b** appear at δ = 14.6 ppm in the ^1^H NMR spectra taken in DMSO-d_6_ and show the integral 0.8. These signals disappear after treatment with base, whereupon the monoanions **17a,b** form as monosodium salts, whose ^77^Se NMR signals appear at 64.8 and 65.0 ppm, respectively, i.e. shifted upfield by Δδ = 23.8 and 22.3 ppm in comparison to the partially dissociated carboxylic acids. Complete saponification of **12a,b** and deprotonation yields the dianionic dicarboxylate **14a**, whose ^77^Se NMR resonance frequencies then appear even further highfield by Δδ = 50.4/48.5 ppm at δ = 42.1 ppm. The resonance frequencies of the dianion **14a** are also solvent-dependent; they occur at δ = −15.5 ppm in MeOD and at δ = −31.8 ppm in CDCl_3_. As for the spectroscopic consequences of Se methylation, the ^77^Se NMR resonance frequencies of selenone **12a** and **12b** shift by about 61 to 153.9 ppm (**13a**) and 152.7 ppm (**13b**) when converted to the selenoethers in DMSO-d_6_. The anion of the cationic selenoethers (BPh_4_^–^ vs PF_6_^–^) apparently has no significant influence on the ^77^Se NMR shift under the measurement conditions applied. Although the manipulation of selenium by methylation is certainly more significant than the deprotonation of substituents at positions 4 and 5, this change in the chemical shift from about 60 ppm to just over 150 ppm can therefore be attributed primarily to the installation of a positive charge; as mentioned at the beginning, however, statements like these should be taken *cum grano salis* due to the sensitivity of the ^77^Se resonance frequencies to structural and external parameters.^35^

**Table 2:** ^77^Se NMR Chemical Shifts in DMSO-d_6_, Sorted by Charge and Referenced to PhSe-SePh @ 461.0 ppm Relative to SeMe_2_ @ 0.0 ppm

charge	cationic			neutral	
compound	13a	13b	13c	12a	12b
δ_Se_ DMSO-*d*_6_ [ppm]	153.9	152.7	153.8	92.5	90.6

charge	partially dissociated		anionic		dianionic
compound	16a	16b	17a	17b	14a
δ_Se_ DMSO-*d*_6_ [ppm]	88.8	87.1	65.0	64.8	42.1

As expected, there is also a pH dependence that confirms the structure of the dianionic molecule **14a**. While, as expected, the values do not change significantly under alkaline conditions, protonation of the carboxylate groups is observable beginning at pH 5.5 and resulted in a ^77^Se NMR value of +16.1 ppm at pH 3.5 in D_2_O (Table 3). Unfortunately, solubility problems prevented further investigations at lower pH values.

**Table 3:** pH Dependence of the ^77^Se Shift of the Disodium Salt **14a**^a^

solvent	D_2_O + NaOD	D_2_O + NaOD	D_2_O	D_2_O + DCl	D_2_O + DCl
pH value	14	11	7.5	5.5	3.5
δ_Se_ DMSO-*d*_6_[ppm]	–16.0	–17.0	–16.5	–10.4	16.1

a Spectra were referenced to PhSe-SePh @ 461.0 ppm relative to SeMe_2_ @ 0.0 ppm in DMSO-*d*_6_.

**Scheme 5:**
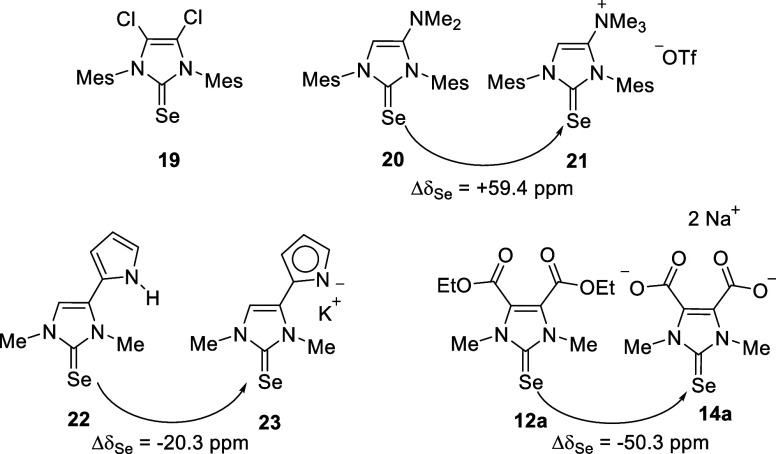
^77^Se NMR Chemical Shift Changes on Installing Charges

The following are comparisons with examples from the literature (Scheme 5): The selenium atoms of the adducts of **3** and **4** are detectable at δ_Se_ (**19**) = 114.0 ppm (acetone-d_6_)^39^ and δ_Se_ (**20**) = 42.8 (acetone-d_6_).^40^ Installing a positive charge by methylation of the exocyclic dimethylamino group results in a chemical shift δ_Se_ (**21**) = 102.2 ppm (acetone-d_6_), i.e., a downfield shift of +59.4 ppm is observed.^12^ As a comparison, the ^77^selenium resonance frequency of pyrrol-2-yl-imidazol-2-selenone **22** shifts from δ_Se_ = 52.1 ppm (DMSO-*d*_6_) to δ_Se_ = 31.8 ppm (DMSO-*d*_6_) (**23**) which corresponds to an upfield shift of Δδ = −20.3 ppm.^36^ The selenone **12a** described here undergoes an upfield shift of Δδ = −50.3 ppm on dianion formation (**14a**), when the spectra were taken in DMSO-*d*_6_. The *conditio sine qua non* for a serious comparison is, as already mentioned several times, to keep the structural differences as small as possible in order to be able to make statements about the influence of charges on the ^77^Se NMR shifts.^35^

Motivated by our interest in the correlations between the electronic properties of various NHCs and the ^77^Se NMR shifts of their selenium adducts, we have calculated the HOMO and LUMO energies of various imidazolium-4,5-dicarboxylate selenium derivatives, as well as their carbene precursors and selenoethers (Table 4). We analyzed derivatives of imidazolium-4,5-dicarboxylates that are neutral molecules, such as **12a,b** and **16a,b**, as well as anionic species such as **17a,b** and dianionic molecules such as **14a** and compared them to the cationic selenoethers. As expected, the cations have the lowest frontier orbital energies, while the dianion has the highest energies. It is also shown that the energies of the selenones and their carbene precursors are of a similar order of magnitude. The energies and HOMO/LUMO gaps can be found in Table 4 and a graphical representation is shown in Figure 5. Methylation of selenium lowers the HOMO energies to about −10.5 eV and the LUMO energies to about −5.4 eV. The negative charge in the imidazolium compounds’ backbones results in a significant increase in both the HOMO and LUMO energies of the compounds compared to the neutral molecules. For example, the HOMO and LUMO energies of the monoanionic sodium salts **17a,b** are about −2.2 and −1.9 eV, respectively. For the dianion **14a**, a further increase in energy can be observed, with the HOMO at 1.3 eV and the LUMO at 4.7 eV. The ^77^Se NMR resonance frequencies, which follow the trend of the HOMO/LUMO energies, are shown as blue dots in Figure 5. The most highly shifted value is that of the dianion **14a** in DMSO-*d*_6_ with a value of 41.1 ppm. A downfield shift of the ^77^Se NMR signal is observed as the negative charges in the molecular backbones are reduced. The shifts of the monoanions are at 65.0 and 64.8 ppm, respectively, and those of the neutral compounds are between 87.1 and 92.5 ppm. The slight highfield shift of **16a** and **16b** can be logically explained by the deprotonation of the acidic protons in the basic solvent DMSO-*d*_6_, as mentioned above.

**Table 4:** Frontier Orbital Energies of the Selenourea Derivatives and Selenenyls As Well As Carbene Precursors

selenone	12a	12b	16a	16b	14a	17a	17b
HOMO [eV]	–5.37	–5.33	–5.51	–5.47	1.28	–2.24	–2.25
LUMO [eV]	–2.18	–2.06	–2.30	–2.31	4.67	1.82	1.93
HOMO/LUMO gap [eV]	–3.19	–3.03	–3.21	–3.16	–3.39	4.06	4.18
carbene							
HOMO [eV]	–6.25	–6.20	–6.37	–6.35	1.77	–2.19	–2.22
LUMO [eV]	–1.78	–1.73	–2.02	–2.00	4.74	2.13	2.07
HOMO/LUMO gap [eV]	–4.47	–4.47	–4.35	–4.35	–2.97	4.32	4.29
δ_Se_ DMSO-*d*_6_ [ppm]	92.5	90.9	88.8	87.1	42.1	65.0	64.8

selenoether	13a	13b	13c
HOMO [eV]	–10.53	–10.46	–10.53
LUMO [eV]	–5.41	–5.38	–5.40
HOMO/LUMO gap [eV]	–5.12	–5.08	–5.13
δ_Se_ DMSO-*d*_6_ [ppm]	153.9	152.7	153.8

**Figure 6:**
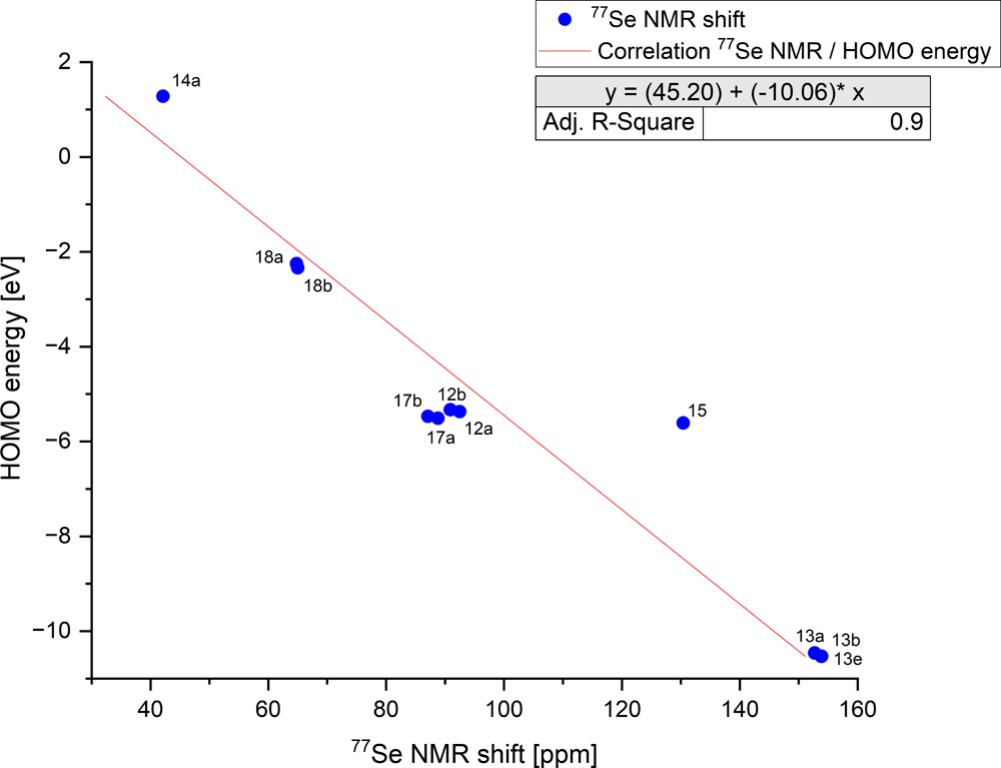
Correlation between the HOMO energies of the selenones and selenoether and their ^77^Se NMR shifts.

**Figure 7:**
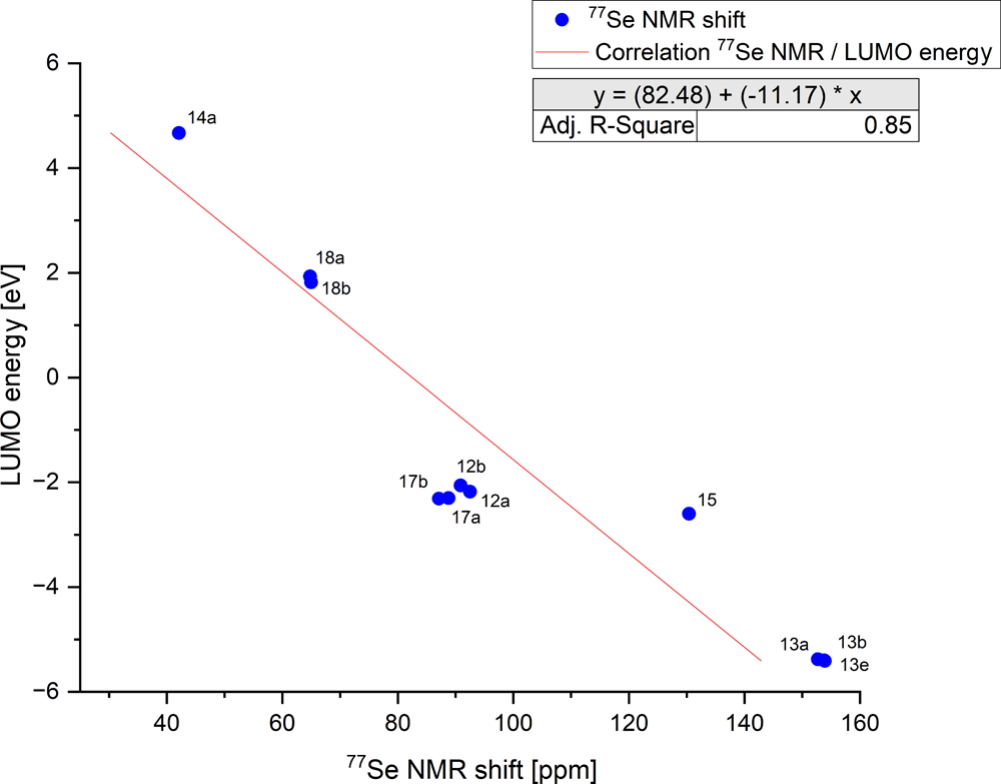
Correlation between the LUMO energies of the selenones and selenoether and their ^77^Se NMR shifts.

The HOMO/LUMO energies and the ^77^Se NMR shifts of both the selenones described here and the selenoethers are linearly related. The correlation coefficient *R*^2^ obtained for the relationship between the HOMO energies is 0.90 (Figure 6), while that between the LUMO energies and the ^77^Se resonance frequencies is 0.85 (Figure 7). Linear relationships between LUMO energies and ^77^Se NMR shifts have been reported in the literature,^25,27^ but their broad applicability has been repeatedly questioned due to the sensitivity of these resonance frequencies and often nonintuitive values.^26a,34^ The compounds examined here show a very high structural similarity, so that there are hardly any other external parameters that can massively influence the values. This is because ^77^Se NMR shifts are the result of many influences, often nonadditive, so that comparability is not given.

## Conclusions

In summary, we were able to show that the selenium adducts of 1,3-imidazole-4,5-dicarboxylic acid derivatives are suitable model compounds for studying the influence of charges on the much-discussed ^77^Se NMR resonance frequencies. These are very sensitive to a myriad of influences, which makes interpreting the ^77^Se NMR shifts as a measure of the π-electronic properties of N-heterocyclic carbenes difficult when too many parameters are changed simultaneously, making the overall effect often unpredictable. The selenium adducts described here were prepared as diesters (charge = 0) of imidazole-4,5-dicarboxylic acid, as the sodium salts of the monoesters (charge = −1), as the fully hydrolyzed dicarboxylate (charge = −2) and as selenenyls (charge = +1). The ^77^Se NMR shifts of these derivatives are in apparently characteristic ranges when the measurement conditions are kept constant, because structural changes that occurred in the molecule’s hinterland, with the exception of Se methylation, were reduced to a minimum. This makes it possible to determine relatively unadulterated charge parameters. As expected, the signals also show a significant solvent dependence and pH dependence in D_2_O. Linear relationships were found between the HOMO energies (*R*^2^ = 0.90) and the LUMO energies (*R*^2^ = 0.85) which both govern the π-architecture and the chemical shifts of the ^77^Se NMR spectroscopy. Despite the sensitivity mentioned above, the ^77^Se NMR shifts of the selenium adducts are considered important parameters for characterizing mainly the π-electronic properties of the underlying N-heterocyclic carbenes. In our model systems, the expected increase in π-donicity from neutral molecules to dianions can be relatively reliably quantified spectroscopically. Therefore, our results may be helpful for all those working in this current field of chemistry.

## Experimental Section

Commercially available reagents and solvents were purchased and used without further purification unless otherwise stated. For thin layer chromatography (TLC), 60 F254 silica-coated aluminum plates from Merck were used and evaluated with UV light. The melting points are uncorrected and were determined in an apparatus according to Dr. Tottoli (Büchi). We used oil baths as heating sources. The ^1^H NMR, ^13^C NMR and ^77^Se NMR spectra were recorded with a Bruker Avance III 600 MHz spectrometer (at 600.35 MHz for ^1^H NMR, 114.50 MHz for ^77^Se NMR, 150.97 MHz for ^13^C NMR) and a Bruker Avance 400 MHz spectrometer (at 400.18 MHz for ^1^H NMR, 76.32 for ^77^Se NMR, 100.63 MHz for ^13^C NMR). Variable temperature (VT) NMR measurements were performed on the above-mentioned 600 MHz AVANCE III spectrometer with thermometer upgrade and the Bruker Smart Variable Temperature System (BSVT) using a BBFO standard probe head. Prior to VT measurement the implemented fully automated self-tune was performed. Each target temperature was successively reached with a gradient of 2 K/min. Temperature stabilization was controlled by BRUKER′s PID system and was achieved within an equilibration time of 15 min. Subsequent gradient shimming was performed at the targeted temperature until no shim changes were reported. The VT gas flow was 600 L/min in order to avoid an internal temperature gradient. Wilmad 528 PP tubes were used in a Kel-F spinner. The shim coil temperature was kept below 55 °C by means of an appropriate flush gas flow. The multiplicities are described with the following abbreviations: s = singlet, d = doublet, t = triplet, q = quartet and m = multiplet. Structural assignments were made with additional information from ^13^C DEPT135, gs-^1^H, ^13^C-HSQC, and gs-^1^H, ^13^C-HMBC measurements. Signal orientations in ^13^C-DEPT135 experiments are described here as follows: o = no signal C_quart_; + = up (CH, CH_3_); - = down (CH_2_). The ^77^Se NMR spectra were referenced to PhSe-SePh @461.0 ppm relative to SeMe_2_ @ 0.0 ppm.^26,35^ For numberings, c.f. Supporting Information. The ATR-IR spectra were measured with a Bruker Alpha in the range from 400 to 4000 cm^–1^. Electrospray ionization mass spectra were measured using a Bruker Impact II TOF mass spectrometer with samples sprayed from MeCN. Some abreviations: PE, petroleum ether; EE, ethyl acetate; DCM, dichloromethane.

### Diisopropyl-1-methyl-1*H*-imidazole-4,5-dicarboxylate **10b**

The 1-methylimidazole-4,5-dicarboxylic acid (1.000 g, 5.878 mmol) was weighed into a round-bottom flask and mixed with a mixture of alcohol and conc. sulfuric acid in a ratio of 10:1. The reaction mixture was then stirred under a protective gas atmosphere for 48 h under reflux temperature. After this reaction time, the reaction mixture was cooled down to room temperature and concentrated *in vacuo*. The reaction mixture was then neutralized with saturated, aqueous sodium hydrogen carbonate solution and extracted with dichloromethane (3 × 100 mL). The organic phases were then combined and washed with an aqueous sodium chloride solution. The organic phase was dried over magnesium sulfate, filtered and concentrated *in vacuo*. Column chromatography was carried out for further purification. A solvent mixture of PE:EE in a mixing ratio of 1:1 was used as eluent. Yield: 1.0746 g (72%), yellowish oil. ^1^H NMR (600 MHz, chloroform-d_1_): δ = 5.25 (sept., ^3^*J*_H–H_ = 6.2 Hz, 1 H, H-9), 5.24 (sept., ^3^*J*_H–H_ = 6.2 Hz, 1 H, H-13), 3.84 (s, 3 H, H-6), 1.38 (d, ^3^*J*_H–H_ = 6.2 Hz, 6 H, H-14/H-14’), 1.37 (d, ^3^*J*_H–H_ = 6.2 Hz, 6 H, H-10/H-10’) ppm. ^13^C{^1^H} NMR (150 MHz, chloroform-d_1_): δ = 162.3 (o, 1C, C-7), 159.6 (o, 1C, C-11), 139.8 (+, 1 C, C-2), 138.0 (o, 1 C, C-4), 125.3 (o, 1 C, C-5), 69.8 (+, 1 C, C-9), 69.0 (+, 1 C, C-13), 34.2 (+, 1 C, C-6), 21.9 (+, 2 C, C-14/C-14’), 21.8 (+, 2 C, C-10/C-10’) ppm. IR (ATR): *ṽ* = 3113, 2981, 2936, 2878, 1709, 1223, 1099 cm^–1^. HRMS (ESI): *m*/*z* [M + Na] Calcd for C_12_H_18_N_2_O_4_ 277.1159; Found: 277.1154.

### 4,5-Bis(isopropoxycarbonyl)-1,3-dimethyl-1*H*-imidazolium Iodide **11b**

The ester **10b** (2.413 g, 9.489 mmol) was placed in a heated and evacuated 25 mL Schlenk tube. Then 10 mL of anhydrous acetonitrile was added. Subsequently, 3.5 equiv of iodomethane (2.07 mL, 33.212 mmol) were added dropwise and the reaction mixture was refluxed for 6 h with vigorous stirring. After this reaction time, the reaction mixture was allowed to cool to rt and the solvent was removed *in vacuo*. The residue was washed with an ice-cooled solvent mixture of PE:EE in a mixing ratio of 2:3. Yield: 3.257 g (87%), yellowish solid. mp.: 168 °C.^1^H NMR (600 MHz, DMSO-d_6_): δ = 9.43 (s, 1 H, H-2), 5.21 (sept., ^3^*J*_H–H_ = 6.2 Hz, 2 H, H-10/H-14), 3.93 (s, 6 H, H-6/H-7), 1.34 (d, ^3^*J*_H–H_ = 6.2 Hz, 12 H, H-11/H-11’/H-15/H-15’) ppm. ^13^C{^1^H} NMR (150 MHz, DMSO-d_6_): δ = 156.1 (o, 2 C, C-8/C-12), 140.8 (o, 1 C, C-2), 126.8 (o, 2 C, C-4/C-5), 71.7 (+, 2 C, C-10/C-14), 36.1 (+, 2 C, C-6/C-7), 21.2 (+, 4 C, C-11/C-11’/C-15/C-15’) ppm. IR (ATR): *ṽ* = 3156, 2995, 2965, 2884, 2793, 1711, 1096 cm^–1^. HRMS (ESI): *m*/*z*: [M] Calcd for C_13_H_21_N_2_O_4_^+^ 269.1496; Found: 269.1494.

### General Procedure for the Syntheses of the Selenones and Thiones **12a–d**

The reactant and elemental selenium (1.2 equiv) or elemental sulfur (1.4 equiv) were placed in a heated and evacuated 25 mL Schlenk tube. Then anhyd. acetonitrile was added. Under vigorous stirring, 2.0 equiv of cesium carbonate was added and the reaction mixture was stirred at room temperature for 2 h. After this reaction time, the reaction mixture was filtered through Celite and the solvent was removed *in vacuo*. Column chromatography was carried out for further purification. A solvent mixture of PE:EE in a mixing ratio of 2:1 was used as eluent.

#### Diethyl-1,3-dimethyl-2-selenoxo-2,3-dihydro-1H-imidazole-4,5-dicarboxylate **12a**

1.500 g (4.074 mmol) of **11a** and 0.386 g (4.889 mmol) of elemental selenium were used. Yield: 1.174 g (90%), apricot-colored solid. mp.: 51 °C. ^1^H NMR (600 MHz, DMSO-d_6_): δ = 4.33 (q, ^3^*J*_H,H_ = 7.1 Hz, 4 H, H-10/H-14), 3.75 (s, 6 H, H-6/H-7), 1.28 (t, ^3^*J*_H,H_ = 7.1 Hz, 6 H, H-11/H-15) ppm. ^13^C{^1^H} NMR (150 MHz, DMSO-d_6_): δ = 161.6 (o, 1 C, C-2), 158.1 (o, 2 C, C-8/C-12), 125.3 (o, 2 C, C-4/C-5), 62.3 (-, 2 C, C10/C-14), 35.8 (+, 2 C, C-6/C-7), 13.7 (+, 2 C, C-11/C-15) ppm. ^77^Se NMR (114.0 MHz, chloroform-d_1_): δ = 92.61 ppm. ^77^Se NMR (114.0 MHz, DMSO-d_6_): δ = 92.5 ppm. IR (ATR): *ṽ* = 2978, 2935, 2902, 2870, 1708, 1337, 1245, 1074 cm^–1^. HRMS (ESI): *m*/*z* [M + Na]^+^ Calcd for C_11_H_16_N_2_O_4_Se 343.0167; Found.: 343.0171.

#### Diisopropyl-1,3-dimethyl-2-selenoxo-2,3-dihydro-1H-imidazole-4,5-dicarboxylate **12b**

0.250 g (0.631 mmol) of **11b** and 0.060 g (0.757 mmol) of elemental selenium were used. Yield: 0.205 g (94%), apricot-colored solid. mp.: 72 °C. ^1^H NMR (600 MHz, DMSO-d_6_): δ = 5.14 (sept., ^3^*J*_H–H_ = 6.3 Hz, 2 H, H-10/H-14), 3.74 (s, 6 H, H-6/H-7), 1.30 (d, ^3^*J*_H–H_ = 6.3 Hz, 12 H, H-11/H-11’/H-15/H-15’) ppm. ^13^C{^1^H} NMR (150 MHz, DMSO-d_6_): δ = 161.3 (o, 1 C, C-2), 157.5 (o, 2 C, C-8/C-12), 125.4 (o, 2 C, C-4/C-5), 70.6 (+, 2 C, C-10/C-14), 35.8 (+, 2 C, C-6/C-7), 21.2 (+, 4 C, C-11/C-11’/C-15/C-15’) ppm. ^77^Se NMR (114.0 MHz, DMSO-d_6_): δ = 90.6 ppm. ^77^Se NMR (114.0 MHz, chloroform-d_1_): δ = 55.3 ppm. IR (ATR): *ṽ* = 2980, 2952, 2934, 1723, 1599, 1350, 1241, 1073, 895 cm^–1^. HRMS (ESI): *m*/*z* [M + Na]^+^ Calcd for C_13_H_20_N_2_O_4_Se 371.0481; Found: 371.0481.

#### Diethyl-1,3-dimethyl-2-thioxo-2,3-dihydro-1H-imidazole-4,5-dicarboxylate **12c**

0.400 g (1.087 mmol) of **11a** and 0.049 g (1.521 mmol) of elemental sulfur were used. Yield: 0.3357 g (98%), apricot-colored solid. mp.: 36 °C ^1^H NMR (600 MHz, DMSO-d_6_): δ = 4.32 (q, ^3^*J*_H,H_ = 7.1 Hz, 4 H, H-10/H-14), 3.65 (s, 6 H, H-6/H-7), 1.28 (t, ^3^*J*_H,H_ = 7.1 Hz, 6 H, H-11/H-15) ppm. ^13^C{^1^H} NMR (150 MHz, DMSO-d_6_): δ = 165.7 (o, 1 C, C-2), 158.3 (o, 2 C, C-8/C-12), 123.4 (o, 2 C, C-4/C-5), 62.2 (-, 2 C, C10/C-14), 33.7 (+, 2 C, C-6/C-7), 13.7 (+, 2 C, C-11/C-15) ppm. IR (ATR): *ṽ* = 2981, 2939, 2906, 2874, 1715, 1336, 1243, 1082 cm^–1^. HRMS (ESI): *m*/*z* [M + Na]^+^ Calcd for C_11_H_16_N_2_O_4_S 295.0723; Found 295.0735.

#### Diisopropyl-1,3-dimethyl-2-thioxo-2,3-dihydro-1H-imidazole-4,5-dicarboxylate **12d**

0.250 g (0.631 mmol) of **11b** and 0.028 g (0.883 mmol) of elemental sulfur were used. Yield: 0.187 (99%), yellowish solid. mp.: 44 °C. ^1^H NMR (600 MHz, DMSO-d_6_): δ = 5.13 (sept., ^3^*J*_H–H_ = 6.2 Hz, 2 H, H-10/H-14), 3.64 (s, 6 H, H-6/H-7), 1.30 (d, ^3^*J*_H–H_ = 6.2 Hz, 12 H, H-11/H-11’/H-15/H-15’) ppm. ^13^C{^1^H} NMR (150 MHz, DMSO-d_6_): δ = 165.5 (o, 1 C, C-2), 157.6 (o, 2 C, C-8/C-12), 123.5 (o, 2 C, C-4/C-5), 70.4 (+, 2 C, C-10/C-14), 33.6 (+, 2 C, C-6/C-7), 21.2 (+, 4 C, C-11/C-11’/C-15/C-15’) ppm. IR (ATR): *ṽ* = 2983, 2937, 2878, 1704, 1584, 1350, 1255, 1071, 905 cm^–1^. HRMS (ESI): *m*/*z* [M + Na]^+^ Calcd. for C_13_H_20_N_2_O_4_S 323.1036; Found: 323.1034.

### General Procedure for the Syntheses of the Selenenyls and Thioethers **13a–f**

The selenone or thione was placed in a heated and evacuated 25 mL Schlenk tube. Then anhydrous acetonitrile was added. While stirring vigorously, 1.5 equiv of methyl trifluoromethyl sulfonate was added. Then, the reaction mixture was stirred at room temperature for 2 h (for the selenoether) or 1 h (for the thioether). After this reaction time, the solvent was removed *in vacuo* and the residue was mixed with 20 mL of pure water. Water-insoluble residues were filtered off via a 0.2 μm cellulose acetate syringe prefilter. An aqueous sodium tetraphenylborate solution was then added dropwise to the filtrate. (The sodium tetraphenylborate solution consists of 1.1 equiv sodium tetraphenylborate and 2 mL of pure water). The resulting precipitate was filtered off, washed with ethanol and dried *in vacuo*.

#### 4,5-Bis(ethoxycarbonyl)-1,3-dimethyl-2-methylselanyl-1H-imidazolium Tetraphenylborate **13a**

0.100 g (0.313 mmol) of **12a** and 0.05 mL (0.470 mmol) of methyl trifluoromethyl sulfonate were used. Yield: 0.1869 g (91%), colorless solid. mp.: 138 °C.^1^H NMR (600 MHz, DMSO-d_6_): δ = 7.21–7.18 (m, 8 H, *o*-PhH), 6.95–6.92 (m, 8 H, *m*-PhH), 6.81–6.79 (m, 4 H, *p*-PhH), 4.43 (q, ^3^*J*_H,H_ = 7.1 Hz, 4 H, H-10/H-14), 4.01 (s, 6 H, H-6/H-7), 2.43 (s, 3 H, H-16), 1.32 (t, ^3^*J*_H,H_ = 7.1 Hz, 6 H, H-11/H-15) ppm. ^13^C{^1^H} NMR (150 MHz, DMSO-d_6_): δ = 163.4 (q, ^1^*J*_C–B_ = 48.9 Hz, 4 C, *ipso*-PhC), 157.0 (o, 2 C, C-8/C-12), 144.6 (o, 1 C, C-2), 135.5 (+, 8 C, *o*-PhC), 127.5 (o, 2 C, C-4/C-5), 125.3 (+, 8 C, *m*-PhC), 121.5 (+, 4 C, *p*-PhC), 63.4 (−, 2 C, C-10/C-14), 37.3 (+, 2 C, C-6/C-7), 13.6 (+, 2 C, C-11/C-15), 10.2 (+, 1 C, C-16) ppm. ^77^Se NMR (114 MHz, DMSO-d_6_): δ = 153.9 ppm. IR (ATR): *ṽ* = 3054, 3000, 2987, 1732, 1251, 734, 707, 611 cm^–1^. HRMS (ESI): *m*/*z* [M]^+^ Calcd for C_12_H_19_N_2_O_4_Se 335.0505; Found: 335.0505. HRMS (ESI): *m*/*z* [M]^−^ Calcd for C_24_H_20_B 319.1664; Found: 319.1669.

#### 4,5-Bis(isopropoxycarbonyl)-1,3-dimethyl-2-methylselanyl-1H-imidazolium Tetraphenylborate **13b**

0.100 g (0.288 mmol) of **12b** and 0.05 mL (0.432 mmol) of methyl trifluoromethyl sulfonate were used. Yield: 0.097 g (50%), colorless solid. mp.: 128 °C.^1^H NMR (600 MHz, DMSO-d_6_): δ = 7.21–7.18 (m, 8 H, *o*-PhH), 6.94–6.92 (m, 8 H, *m*-PhH), 6.81–6.78 (m, 4 H, *p*-PhH), 5.25 (sept., ^3^*J*_H–H_ = 6.2 Hz, 2 H, H-10/H-14), 3.99 (s, 6 H, H-6/H-7), 2.43 (s, 3 H, H-16), 1.35 (d, ^3^*J*_H–H_ = 6.2 Hz, 12 H, H-11/H-11’/H-15/H-15’) ppm. ^13^C{^1^H} NMR (150 MHz, DMSO-d_6_): δ = 163.4 (q, ^1^*J*_C–B_ = 48.9 Hz, 4 C, *ipso*-PhC), 156.3 (o, 2 C, C-8/C-12), 144.3 (o, 1 C, C-2), 135.5 (+, 8 C, *o*-PhC), 128.1 (o, 2 C, C-4/C-5), 125.3 (+, 8 C, *m*-PhC), 121.5 (+, 4 C, *p*-PhC), 72.0 (+, 2 C, C-10/C-14), 37.2 (+, 2 C, C-6/C-7), 21.2 (+, 4 C, C-11/C-11’/C-15/C-15’), 10.2 (+, 1 C, C-16) ppm. ^77^Se NMR (114 MHz, DMSO-d_6_): δ = 152.7 ppm. IR (ATR): *ṽ* = 3053, 3033, 2983, 1728, 1251, 1085, 733, 705, 611 cm^–1^. HRMS (ESI): *m*/*z* [M]^+^ Calcd for C_14_H_23_N_2_O_4_Se 363.0818; Found: 363.0818. HRMS (ESI): *m*/*z* [M]^−^ Calcd for C_24_H_20_B 319.1664; Found: 319.1669.

#### 4,5-Bis(ethoxycarbonyl)-1,3-dimethyl-2-methylthionyl-1H-imidazolium Tetraphenylborate **13c**

0.100 g (0.367 mmol) of **12c** and 0.06 mL (0.551 mmol) of methyl trifluoromethyl sulfonate were used. Yield: 0.174 g (78%), colorless solid. mp.: 157 °C. ^1^H NMR (600 MHz, DMSO-d_6_): δ = 7.21–7.18 (m, 8 H, *o*-PhH), 6.95–6.92 (m, 8 H, *m*-PhH), 6.81–6.79 (m, 4 H, *p*-PhH), 4.44 (q, ^3^*J*_H–H_ = 7.1 Hz, 4 H, H-10/H-14), 3.99 (s, 6 H, H-6/H-7), 2.58 (s, 3 H, H-16), 1.32 (t, ^3^*J*_H–H_ = 7.1 Hz, 6 H, H-11/H-15) ppm. ^13^C{^1^H} NMR (150 MHz, DMSO-d_6_): δ = 163.4 (q, ^1^*J*_C–B_ = 49.0 Hz, 4 C, *ipso*-PhC), 156.8 (o, 2 C, C-8/C-12), 147.3 (o, 1 C, C-2), 135.5 (+, 8 C, *o*-PhC), 127.5 (o, 2 C, C-4/C-5), 125.3 (+, 8 C, *m*-PhC), 121.5 (+, 4 C, *p*-PhC), 63.5 (−, 2 C, C-10/C-14), 36.0 (+, 2 C, C-6/C-7), 16.9 (+, 1 C, C-16), 13.6 (+, 2 C, C-11/C-15) ppm. IR (ATR): *ṽ* = 3053, 3001, 2986, 1735, 1251, 735, 708, 611 cm^–1^. HRMS (ESI): *m*/*z* [M]^+^ Calcd for C_12_H_19_N_2_O_4_S^+^: 287.1060; Found: 287.1060. HRMS (ESI): *m*/*z* [M]^−^ Calcd for C_24_H_20_B 319.1664; Found: 319.1664.

#### 4,5-Bis(isopropoxycarbonyl)-1,3-dimethyl-2-methylthionyl-1H-imidazolium Tetraphenylborate **13d**

0.100 g (0.333 mmol) of **12d** and 0.06 mL (0.500 mmol) of methyl trifluoromethyl sulfonate were used. Yield: 0.120 g (57%), colorless solid. mp.: 135 °C. ^1^H NMR (600 MHz, DMSO-d_6_): δ = 7.21–7.18 (m, 8 H, *o*-PhH), 6.95–6.92 (m, 8 H, *m*-PhH), 6.81–6.79 (m, 4 H, *p*-PhH), 5.25 (sept., ^3^*J*_H–H_ = 6.2 Hz, 2 H, H-10/H-14), 3.98 (s, 6 H, H-6/H-7), 2.57 (s, 3 H, H-16), 1.35 (d, ^3^*J*_H–H_ = 6.2 Hz, 12 H, H-11/H-11’/H-15/H-15’) ppm. ^13^C{^1^H} NMR (150 MHz, DMSO-d_6_): δ = 163.4 (q, ^1^*J*_C–B_ = 48.9 Hz, 4 C, *ipso*-PhC), 156.2 (o, 2 C, C-8/C-12), 147.0 (o, 1 C, C-2), 135.5 (+, 8 C, *o*-PhC), 127.7 (o, 2 C, C-4/C-5), 125.3 (+, 8 C, *m*-PhC), 121.5 (+, 4 C, *p*-PhC), 72.1 (+, 2 C, C-10/C-14), 36.0 (+, 2 C, C-6/C-7), 21.2 (+, 4 C, C-11/C-11’/C-15/C-15’), 16.9 (+, 1 C, C-16) ppm. IR (ATR): *ṽ* = 3053, 3032, 2984, 1728, 1254, 1087, 734, 706, 612 cm^–1^. HRMS (ESI): *m*/*z* [M]^+^ Calcd for C_14_H_23_N_2_O_4_S^+^ 315.1373; Found: 315.1367. HRMS (ESI): *m*/*z* [M]^−^ Calcd for C_24_H_20_B^–^ 319.1664; Found: 319.1667.

#### 4,5-Bis(ethoxycarbonyl)-1,3-dimethyl-2-methylselanyl-1H-imidazolium Hexafluorophosphate **13e**

0.500 g (1.566 mmol) of **12a** and 0.27 mL (2.349 mmol) of methyl trifluoromethyl sulfonate were used. Yield: 0.556 g (74%), colorless solid. mp.: 100 °C. ^1^H NMR (600 MHz, DMSO-d_6_): δ = 4.43 (q, ^3^*J*_H,H_ = 7.1 Hz, 4 H, H-10/H-14), 4.02 (s, 6 H, H-6/H-7), 2.45 (s, 3 H, H-16), 1.32 (t, ^3^*J*_H,H_ = 7.1 Hz, 6 H, H-11/H-15) ppm. ^13^C{^1^H} NMR (150 MHz, DMSO-d_6_): δ = 157.0 (o, 2 C, C-8/C-12), 144.7 (o, 1 C, C-2), 127.9 (o, 2 C, C-4/C-5), 63.4 (−, 2 C, C10/C-14), 37.3 (+, 2 C, C-6/C-7), 13.6 (+, 2 C, C-11/C-15), 10.2 (+, 1 C, C-16) ppm. ^77^Se NMR (114 MHz, DMSO-d_6_): δ = 153.8 ppm. ^77^Se NMR (114 MHz, chloroform-d_1_): δ = 127.4 ppm. IR (ATR): *ṽ* = 2997, 2970, 2953, 2907, 1723, 1256, 827, 556 cm^–1^. HRMS: *m*/*z* [M]^+^ Calcd for C_12_H_19_N_2_O_4_Se 335.0505; Found: 335.0506.

#### 4,5-Bis(ethoxycarbonyl)-1,3-dimethyl-2-methylthionyl-1H-imidazolium Hexafluorophosphate **13f**

0.200 g (0.734 mmol) of **12c** and 0.13 mL (1.102 mmol) of methyl trifluoromethyl sulfonate were used. Yield: 0.177 g (56%), colorless solid. mp.: 111 °C. ^1^H NMR (600 MHz, DMSO-d_6_): δ = 4.44 (q, ^3^*J*_H,H_ = 7.1 Hz, 4 H, H-10/H-14), 4.01 (s, 6 H, H-6/H-7), 2.60 (s, 3 H, H-16), 1.32 (t, ^3^*J*_H,H_ = 7.1 Hz, 6 H, H-11/H-15) ppm. ^13^C{^1^H} NMR (150 MHz, DMSO-d_6_): δ = 156.8 (o, 2 C, C-8/C-12), 147.4 (o, 1 C, C-2), 127.5 (o, 2 C, C-4/C-5), 63.5 (−, 2 C, C10/C-14), 36.0 (+, 2 C, C-6/C-7), 16.9 (+, 1 C, C-16), 13.6 (+, 2 C, C-11/C-15) ppm. IR (ATR): *ṽ* = 2994, 2945, 2911, 1729, 1251, 828, 556 cm^–1^. HRMS (ESI): *m*/*z* [M]^+^ Calcd for C_12_H_19_N_2_O_4_S 287.1066; Found: 287.1065.

### General Procedure for the Syntheses of the Disodium Dicarboxylates **14a,b**

The selenium adduct or the sulfur adduct was placed in a 50 mL round-bottom flask. DCM was then added as a solvent. A 0.3 M methanolic sodium hydroxide solution was then added dropwise. The DCM:MeOH solvent ratio was 9:1 and the reaction mixture was stirred vigorously until it reaches room temperature. The resulting precipitate was filtered off, washed with cold DCM and dried *in vacuo*.

#### Disodium 1,3-Dimethyl-2-selenoxo-2,3-dihydro-1H-imidazole-4,5-dicarboxylate **14a**

0.100 g (0.313 mmol) of **12a** were used. Yield: 0.169 g (100%), beige-colored solid. mp.: 125 °C. ^1^H NMR (600 MHz, DMSO-d_6_): δ = 3.59 (s, 6 H, H-6/H-7) ppm. ^13^C NMR (150 MHz, DMSO-d_6_): δ = 164.9 (o, 2 C, C-8/C-10), 154.4 (o, 1 C, C-2), 129.5 (o, 2 C, C-4/C-5), 35.0 (+, 2 C, C-6/C-7) ppm. ^77^Se NMR (114.0 MHz, DMSO-d_6_): δ = 42.05 ppm. IR (ATR): *ṽ* = 3451, 2947, 1660, 1594, 1395, 1332 cm^–1^. ^1^H NMR (600 MHz, water-d_2_): δ = 3.76 (s, 6 H, H-6/H-7) ppm. ^13^C{^1^H} NMR (150 MHz, water-d_2_): δ = 165.8 (o, 2 C, C-8/C-10), 151.6 (o, 1 C, C-2), 129.9 (o, 2 C, C-4/C-5), 35.5 (+, 2 C, C-6/C-7) ppm. ^77^Se NMR (114.0 MHz, chloroform-d_1_): δ = −31.84 ppm. ^77^Se NMR (114.0 Hz, DMSO-d_6_): 42.1 ppm. (IR (ATR): *ṽ* = 3489, 3405, 2948, 1621, 1602, 1383, 1363, 912 cm^–1^. HRMS (ESI): *m*/*z* [M + H]^−^ Calcd for C_7_H_8_N_2_O_4_Se 262.9571; Found: 262.9572.

#### Disodium 1,3-Dimethyl-2-thioxo-2,3-dihydro-1H-imidazole-4,5-dicarboxylic Acid **14b**

0.100 g (0.367 mmol) of **12c** were used. Yield: 0.079 g (100%), colorless soid. dec 294.4 °C. ^1^H NMR (600 MHz, water-d_2_): δ = 3.68 (s, 6 H, H-6/H-7) ppm. ^13^C{^1^H} NMR (150 MHz, water-d_2_): δ = 168.8 (o, 2 C, C-8/C-10), 161.1 (o, 1 C, C-2), 131.0 (o, 2 C, C-4/C-5), 36.3 (+, 2 C, C-6/C-7) ppm. IR (ATR): *ṽ* = 3422, 2945, 1611, 1568, 1384, 1335, 545 cm^–1^. HRMS (ESI): *m*/*z* [M + H]^−^ Calcd for C_7_H_8_N_2_O_4_S 215.0127; Found 215.0124.

#### Dimethyl-1,3-dimethyl-2-selenoxo-2,3-dihydro-1H-imidazole-4,5-dicarboxylate **15**

A mixture of DCM:methanol (1:1) was used, same procedure as for **14a,b**. Yield: 0.048 g (52%), yellowish solid. mp.: 146 °C. ^1^H NMR (600 MHz, DMSO-d_6_): δ = 3.87 (s, 6 H, H-10/H-13), 3.75 (s, 6 H, H-6/H-7) ppm. ^13^C{^1^H} NMR (150 MHz, DMSO-d_6_): δ = 161.8 (o, 1 C, C-2), 158.6 (o, 2 C, C-8/C-11), 125.3 (o, 2 C, C-4/C-5), 53.2 (+, 2 C, C-10/C-13), 35.9 (+, 2 C, C-6/C-7) ppm. ^77^Se NMR (114.0 MHz, DMSO-d_6_): δ = 94.55 ppm. IR (ATR): *ṽ* = 2949, 1716, 1346, 1256, 1079, 901 cm^–1^. HRMS (ESI): *m*/*z* [M + Na]+ Calcd for C_9_H_12_N_2_O_4_Se 314.9854; Found: 314.9858.

### General Experimental Procedure for the Syntheses of **16a–d**

The selenium adduct or the sulfur adduct was placed in a 50 mL round-bottom flask, mixed with a solvent mixture of DMSO: ultrapure water in a mixing ratio of 3:1 and cooled to 0 °C in an ice bath. An aqueous 0.5 M potassium hydroxide solution (0.2 mL solution every 15 min) was then added in portions over a reaction period of 4 h until the reactant spot on the tlc has completely disappeared. After the 4h reaction time, an aqueous 1 M hydrochloric acid solution was added dropwise until the pH value of the reaction mixture was pH = 5–6. The reaction mixture was then extracted with diethyl ether (3 × 100 mL). The organic phases were combined, washed with a saturated sodium chloride solution, dried over magnesium sulfate and filtered. The filtrate was concentrated *in vacuo*. The residue was then washed again with *n*-hexane and dried *in vacuo*.

#### 5-(Ethoxycarbonyl)-1,3-dimethyl-2-selenoxo-2,3-dihydro-1H-imidazole-4-carboxylic Acid **16a**

0.100 g (0.313 mmol) of **12a** was used. Yield: 0.042 (46%), orange-colored solid. mp.: 127 °C. ^1^H NMR (600 MHz, DMSO-d_6_): δ = 14.34 (s broad, 1 H, H-13), 4.32 (q, ^3^*J*_H–H_ = 7.1 Hz, 2 H, H-10), 3.75 (s, 3 H, H-7), 3.73 (s, 3 H, H-6), 1.27 (t, ^3^*J*_H–H_ = 7.1 Hz, 3 H, H-11) ppm. ^13^C{^1^H} NMR (150 MHz, DMSO-d_6_): δ = 161.1 (o, 1 C, C-2), 159.6 (o, 1 C, C-12), 158.4 (o, 1 C, C-8), 126.7 (o, 1 C, C-4), 124.8 (o, 1 C, C-5), 62.2 (-, 1 C, C-10), 35.8 (+, 1 C, C-7), 35.7 (+, 1 C, C-6), 13.6 (+, 1 C, C-11) ppm. ^77^Se NMR (114.0 MHz, DMSO-d_6_): δ = 88.8 ppm. IR (ATR): *ṽ* = 2981, 2941, 1744, 1695, 1322, 1260 cm^–1^. HRMS (ESI): *m*/*z* [M – H]^−^ Calcd for C_9_H_12_N_2_O_4_Se 290.9890; Found: 290.9889.

#### 5-(Isopropoxycarbonyl)-1,3-dimethyl-2-selenoxo-2,3-dihydro-1H-imidazole-4-carboxylic Acid **16b**

0.100 g (0.288 mmol) of **12b** was used. Yield: 0.046 g (52%), yellow-orange-colored solid. mp.: 128 °C. ^1^H NMR (600 MHz, DMSO-d_6_): δ = 14.36 (s broad, 1 H, H-13), 5.13 (sept., ^3^*J*_H–H_ = 6.3 Hz, 1 H, H-10), 3.75 (s, 3 H, H-7), 3.73 (s, 3 H, H-6), 1.28 (d, ^3^*J*_H–H_ = 6.3 Hz, 6 H, H-11/H-11’) ppm. ^13^C{^1^H} NMR (150 MHz, DMSO-d_6_): δ = 161.0 (o, 1 C, C-2), 159.6 (o, 1 C, C-12), 157.9 (o, 1 C, C-8), 126.5 (o, 1 C, C-4), 125.1 (o, 1 C, C-5), 70.3 (+, 1 C, C-10), 35.8 (+, 1 C, C-7), 35.6 (+, 1 C, C-6), 21.1 (+, 2 C, C-11/C-11’) ppm. ^77^Se NMR (114 MHz, DMSO-d_6_): δ = 87.1 ppm. IR (ATR): *ṽ* = 2982, 1714, 1370, 1273, 1219, 1082 cm^–1^. HRMS (ESI): *m*/*z* [M – H]^−^ Calcd for C_10_H_14_N_2_O_4_Se 305.0046; Found: 305.0038.

#### 5-(Ethoxycarbonyl)-1,3-dimethyl-2-thioxo-2,3-dihydro-1H-imidazole-4-carboxylic Acid **16c**

0.100 g (0.367 mmol) of **12c** was used. Yield: 0.036 (40%), yellow solid. mp.: 134 °C. ^1^H NMR (600 MHz, DMSO-d_6_): δ = 14.22 (s broad, 1 H, H-13), 4.30 (q, ^3^*J*_H–H_ = 7.1 Hz, 2 H, H-10), 3.65 (s, 3 H, H-7), 3.63 (s, 3 H, H-6), 1.26 (t, ^3^*J*_H–H_ = 7.1 Hz, 3 H, H-11) ppm. ^13^C{^1^H} NMR (150 MHz, DMSO-d_6_): δ = 165.4 (o, 1 C, C-2), 159.7 (o, 1 C, C-12), 158.5 (o, 1 C, C-8), 124.8 (o, 1 C, C-4), 122.8 (o, 1 C, C-5), 62.0 (-, 1 C, C-10), 33.7 (+, 1 C, C-7), 33.6 (+, 1 C, C-6), 13.6 (+, 1 C, C-11) ppm. IR (ATR): *ṽ* = 2918, 2850, 1742, 1689, 1257, 853 cm^–1^. HRMS (ESI): *m*/*z* [M – H]^−^ Calcd for C_9_H_12_N_2_O_4_S 243.0439; Found: 243.0438.

#### 5-(Isopropoxycarbonyl)-1,3-dimethyl-2-thioxo-2,3-dihydro-1H-imidazole-4-carboxylic Acid **16d**

0.100 g (0.333 mmol) of **12d** was used. Yield: 0.035 g (52%), yellow solid. mp.: 132 °C.^1^H NMR (600 MHz, DMSO-d_6_): δ = 14.18 (s broad, 1 H, H-13), 5.12 (sept, ^3^*J*_H–H_ = 6.2 Hz, 1 H, H-10), 3.65 (s, 3 H, H-7), 3.62 (s, 3 H, H-6), 1.27 (d, ^3^*J*_H–H_ = 6.2 Hz, 6 H, H-11/H-11’) ppm. ^13^C{^1^H} NMR (150 MHz, DMSO-d_6_): δ = 165.3 (o, 1 C, C-2), 159.7 (o, 1 C, C-12), 158.1 (o, 1 C, C-8), 124.6 (o, 1 C, C-4), 123.2 (o, 1 C, C-5), 70.1 (+, 1 C, C-10), 33.6 (+, 1 C, C-7), 33.5 (+, 1 C, C-6), 21.2 (+, 2 C, C-11/C-11’) ppm. IR (ATR): *ṽ* = 2985, 2951, 1738, 1712, 1374, 1080 cm^–1^. HRMS (ESI): *m*/*z* [M-H]^−^ Calcd for C_10_H_14_N_2_O_4_S 257.0602; Found: 257.0599.

### General Experimental Procedure for the Syntheses of **17a,b**

The monocarboxylic acid **16a** or **16b** was suspended in ultrapure water in a 5 mL round-bottom flask. To the suspension 1 equiv sodium carbonate was added, resulting in a direct decolorisation of the solution. After the solution was stirred for an additional 5 min, the solvent was removed using a rotary evaporator and the product was dried *in vacuo*.

#### Sodium 5-(Ethoxycarbonyl)-1,3-dimethyl-2-selenoxo-2,3-dihydro-1H-imidazole-4-carboxylate **17a**

0.025 g (0.087 mmol) of **16a** was used. Yield: 0.027 (quant.), colorless solid. mp.: >250 °C. ^1^H NMR (600 MHz, DMSO-d_6_): δ = 4.18 (q, ^3^*J*_H–H_ = 7.1 Hz, 2 H, H-10), 3.75 (s, 3 H, H-7), 3.56 (s, 3 H, H-6), 1.21 (t, ^3^*J*_H–H_ = 7.1 Hz, 3 H, H-11) ppm. ^13^C{^1^H} NMR (150 MHz, DMSO-d_6_): δ = 160.6 (o, 1 C, C-2), 159.7 (o, 1 C, C-12), 158.1 (o, 1 C, C-8), 141.5 (o, 1 C, C-4/C-5), 115.80 (o, 1 C, C-4/C-5), 60.9 (−, 1 C, C-10), 36.1 (+, 1 C, C-6/C-7), 35.4 (+, 1 C, C-6/C-7), 14.4 (+, 1 C, C-11) ppm. ^77^Se NMR (114.0 MHz, DMSO-d_6_): δ = 65.0 ppm. IR (ATR): *ṽ* = 3326, 2977, 2944, 1708, 1612, 1582, 1467, 1440, 1376, 1344, 1326, 1262, 1183, 1159, 1146, 1101, 1081, 1023, 930, 891, 856, 829, 800, 777, 743, 700, 667, 637, 578, 521, 422 cm^–1^. HRMS (ESI): *m*/*z* [M]^−^ Calcd for C_9_H_11_N_2_O_4_Se 290.9890; Found: 290.9892.

#### Sodium 5-(Isopropoxycarbonyl)-1,3-dimethyl-2-selenoxo-2,3-dihydro-1H-imidazole-4-carboxylate **17b**

0.025 g (0.082 mmol) of **16b** was used. Yield: 0.029 g (quant.), colorless solid. mp.: >250 °C.^1^H NMR (600 MHz, DMSO-d_6_): δ = 5.00 (sept., ^3^*J*_H–H_ = 6.3 Hz, 1 H, H-10), 3.74 (s, 3 H, H-7), 3.59 (s, 3 H, H-6), 1.23 (d, ^3^*J*_H–H_ = 6.3 Hz, 6 H, H-11/H-11’) ppm. ^13^C{^1^H} NMR (150 MHz, DMSO-d_6_): δ = 160.4 (o, 1 C, C-2), 158.8 (o, 1 C, C-12), 157.6 (o, 1 C, C-8), 140.4 (o, 1 C, C-4/C-5), 116.3 (o, 1 C, C-4/C-5), 68.3 (+, 1 C, C-10), 34.9 (+, 1 C, C-6/C-7), 35.6 (+, 1 C, C-6/C-7), 21.4 (+, 2 C, C-11/C-11’) ppm. ^77^Se NMR (114 MHz, DMSO-d_6_): δ = 64.8 ppm. IR (ATR): *ṽ* = 3321, 2977, 2930, 1707, 1629, 1580, 1467, 1439, 1375, 1344, 1324, 1260, 1183, 1161, 1145, 1101, 1071, 930, 891, 856, 828, 800, 777, 742, 700, 668, 636, 521, 413 cm^–1^. HRMS (ESI): *m*/*z* [M]^−^ Calcd for C_10_H_13_N_2_O_4_Se 305.0046; Found: 305.0050.

## Data Availability

The data underlying this study are available in the published article and its Supporting Information.

## Supporting Information Available

Numberings of all compounds; ^1^H NMR spectra of all synthesized components; ^13^C{^1^H} NMR spectra and 2D-NMR spectra (HSQC, HMBC) of all unknown compounds; ^77^Se NMR spectra of all compounds containing selenium; data from the DFT calculations; HOMO/LUMO profiles; and calculations for geometry optimizations (PDF)

## References

[ref1] aBiju A. T.; Breslow R. N-Heterocyclic Carbenes in Organocatalysis; Wiley/VCH: Weinheim, Germany, 2019.

[ref2] Borguet Y.; Zaragoza G.; Demonceau A.; Delaude L. Assessing the Ligand Properties of 1,3-Dimesitylbenzimidazol-2-ylidene in Ruthenium-Catalyzed Olefin Metathesis. Dalton Trans. 2013, 42, 7287–7296. 10.1039/C2DT31520C. 22990296

[ref3] aVanucci-Bacqué C.; Wolff M.; Delavaux-Nicot B.; Mosaad Abdallah A.; Mallet-Ladeira S.; Serpentini C.-L.; Bedos-Belval F.; Fong K. W.; Ng X. Y.; Low M. L.; Benoist E.; Fery-Forgues S. 1,2,3-Triazol-5-ylidene- vs. 1,2,3-Triazole-Based Tricarbonylrhenium(I) Complexes: Influence of a Mesoionic Carbene Ligand on the Electronic and Biological Properties. Dalton Trans. 2024, 53 (27), 11276–11294. 10.1039/d4dt00922c. 38776120

[ref4] aThie C.; Hitzel S.; Wallbaum L.; Bruhn C.; Siemeling U. Coinage Metal Complexes of the Carbenic Tautomer of Nitron. J. Organomet. Chem. 2016, 821, 112–121. 10.1016/j.jorganchem.2016.03.023.

[ref5] aLi W.; Tang J.; Li S.; Zheng X.; Yuan M.; Xu B.; Jiang W.; Fu H.; Li R.; Chen H. Stereodivergent Synthesis of Alkenylpyridines via Pd/Cu Catalyzed C–H Alkenylation of Pyridinium Salts with Alkynes. Org. Lett. 2020, 22 (20), 7814–7819. 10.1021/acs.orglett.0c02679. 33026228

[ref6] Ung G.; Mendoza-Espinosa D.; Bertrand G. Ynamides: Stable Ligand Equivalents of Unstable Oxazol-4-Ylidenes (Novel Mesoionic Carbenes). Chem. Commun. 2012, 48 (56), 7088–7090. 10.1039/c2cc33319h. 22684100

[ref7] Guan Z.; Nieger M.; Schmidt A. Organic Synthesis with N-Heterocyclic Carbenes of Indazole: Synthesis of Benzo(Thio)Imidates, Benzo[d][1,3]thiazines and Quinazoline-4-thiones. Eur. J. Org. Chem. 2015, 2015 (21), 4710–4719. 10.1002/ejoc.201500331.

[ref8] Schmidt A.; Mummel S.; Lederle F.; Hübner E. G.; Namyslo J. C.; Nieger M. Sydnone Methides — a Forgotten Class of Mesoionic Compounds for the Generation of Anionic N-Heterocyclic Carbenes. Angew. Chem., Int. Ed. 2021, 60 (34), 18882–18887. 10.1002/anie.202107495. PMC8456854 34153173

[ref9] Arduengo A. J.; Harlow R. L.; Kline M. A Stable Crystalline Carbene. J. Am. Chem. Soc. 1991, 113 (1), 361–363. 10.1021/ja00001a054.

[ref10] Schuster O.; Yang L.; Raubenheimer H. G.; Albrecht M. Beyond Conventional N-Heterocyclic Carbenes: Abnormal, Remote, and Other Classes of NHC Ligands with Reduced Heteroatom Stabilization. Chem. Rev. 2009, 109 (8), 3445–3478. 10.1021/cr8005087. 19331408

[ref11] Urbina-Blanco C. A.; Bantreil X.; Clavier H.; Slawin A. M. Z.; Nolan S. P. Backbone Tuning in Indenylidene–Ruthenium Complexes Bearing an Unsaturated *N*-Heterocyclic Carbene. Beilstein J. Org. Chem. 2010, 6, 1120–1126. 10.3762/bjoc.6.128. 21160916 PMC3002078

[ref12] Ruamps M.; Lugan N.; César V. A Cationic N-Heterocyclic Carbene Containing an Ammonium Moiety. Organometallics 2017, 36 (5), 1049–1055. 10.1021/acs.organomet.7b00017.

[ref13] aBenhamou L.; Vujkovic N.; César V.; Gornitzka H.; Lugan N.; Lavigne G. Facile Derivatization of a “Chemo-Active” NHC Incorporating an Enolate Backbone and Relevant Tuning of Its Electronic Properties. Organometallics 2010, 29 (11), 2616–2630. 10.1021/om1003607.

[ref14] aCésar V.; Tourneux J.-C.; Vujkovic N.; Brousses R.; Lugan N.; Lavigne G. Interplay between an Elusive 4-(Isopropylamino)Imidazol-2-ylidene and its Isolable Mesoionic Tautomer, and Associated Reactivities. Chem. Commun. 2012, 48 (17), 2349–2349. 10.1039/c2cc17870b. 22258227

[ref15] Phillips N.; Tirfoin R.; Aldridge S. Anionic N-Heterocyclic Carbenes (NHCs): A Versatile Route to Saturated NHCs Bearing Pendant Weakly Coordinating Anions. Dalton Trans. 2014, 43 (41), 15279–15282. 10.1039/C4DT02662D. 25198297

[ref16] Schmidt A.; Wiechmann S.; Freese T. Recent Advances in Neutral and Anionic N-Heterocyclic Carbene–Betaine Interconversions Synthesis, Characterization, and Applications. Arkivoc 2013, 2013 (1), 424–469. 10.3998/ark.5550190.p008.251.

[ref17] aCésar V.; Lugan N.; Lavigne G. A Stable Anionic N-Heterocyclic Carbene and Its Zwitterionic Complexes. J. Am. Chem. Soc. 2008, 130 (34), 11286–11287. 10.1021/ja804296t. 18680370

[ref18] Guo X.; Huang Z.; Xiong L.; Dong L.; Huang Y.; Wei L.; Tang R.; Wang Z.; Xu H. Azole Selenourea Disrupted the Midgut and Caused Malformed Development of Plutella Xylostella. J. Integr. Agricult. 2023, 22 (4), 1104–1116. 10.1016/j.jia.2022.09.001.

[ref19] aClarke M.; Taube H. Nitrogen-Bound and Carbon-Bound Xanthine Complexes of Ruthenium Ammines. J. Am. Chem. Soc. 1975, 97 (6), 1397–1403. 10.1021/ja00839a020.

[ref20] Dorta R.; Stevens E. D.; Scott N. M.; Costabile C.; Cavallo L.; Hoff C. D.; Nolan S. P. Steric and Electronic Properties of N-Heterocyclic Carbenes (NHC): A Detailed Study on Their Interaction with Ni(CO)_4_. J. Am. Chem. Soc. 2005, 127 (8), 2485–2495. 10.1021/ja0438821. 15725003

[ref21] aTolman C. A. Steric Effects of Phosphorus Ligands in Organometallic Chemistry and Homogeneous Catalysis. Chem. Rev. 1977, 77 (3), 313–348. 10.1021/cr60307a002.

[ref22] aSetiawan D.; Kalescky R.; Kraka E. Direct Measure of Metal–Ligand Bonding Replacing the Tolman Electronic Parameter. Inorg. Chem. 2016, 55 (5), 2332–2344. 10.1021/acs.inorgchem.5b02711. 26900632

[ref23] aHuynh H. V.; Han Y.; Jothibasu R.; Yang J. A. ^13^C NMR Spectroscopic Determination of Ligand Donor Strengths Using N-Heterocyclic Carbene Complexes of Palladium(II). Organometallics 2009, 28 (18), 5395–5404. 10.1021/om900667d.

[ref24] Back O.; Henry-Ellinger M.; Martin C. D.; Martin D.; Bertrand G. ^31^P NMR Chemical Shifts of Carbene-Phosphinidene Adducts as an Indicator of the π-Accepting Properties of Carbenes. Angew. Chem., Int. Ed. 2013, 52 (10), 2939–2943. 10.1002/anie.201209109. 23364832

[ref25] Verlinden K.; Buhl H.; Frank W.; Ganter C. Determining the Ligand Properties of N-Heterocyclic Carbenes From^77^Se NMR Parameters. Eur. J. Inorg. Chem. 2015, 2015 (14), 2416–2425. 10.1002/ejic.201500174.

[ref26] aHuynh H. V. Electronic Properties of N-Heterocyclic Carbenes and Their Experimental Determination. Chem. Rev. 2018, 118 (19), 9457–9492. 10.1021/acs.chemrev.8b00067. 29601194

[ref27] aLiske A.; Verlinden K.; Buhl H.; Schaper K.; Ganter C. Determining the π-Acceptor Properties of N-Heterocyclic Carbenes by Measuring the ^77^Se NMR Chemical Shifts of Their Selenium Adducts. Organometallics 2013, 32 (19), 5269–5272. 10.1021/om400858y.

[ref28] Hansch C.; Leo A.; Taft R. W. A Survey of Hammett Substituent Constants and Resonance and Field Parameters. Chem. Rev. 1991, 91 (2), 165–195. 10.1021/cr00002a004.

[ref29] Taft R. W. Linear Steric Energy Relationships. J. Am. Chem. Soc. 1953, 75 (18), 4538–4539. 10.1021/ja01114a044.

[ref30] Jamil M. S. S.; Endot N. A. Influence of Fluorine Substituents on the Electronic Properties of Selenium-N-Heterocyclic Carbene Compounds. Molecules 2020, 25 (21), 5161 10.3390/molecules25215161. 33171911 PMC7664287

[ref31] Junor G. P.; Lorkowski J.; Weinstein C. M.; Jazzar R.; Pietraszuk C.; Bertrand G. The Influence of C(sp^3^)H–Selenium Interactions on the ^77^Se NMR Quantification of the π-Accepting Properties of Carbenes. Angew. Chem., Int. Ed. 2020, 59 (49), 22028–22033. 10.1002/ange.202010744. 32822513

[ref32] Liu L. L.; Zhu D.; Cao L. L.; Stephan D. W. N-Heterocyclic Carbene Stabilized Parent Sulfenyl, Selenenyl, and Tellurenyl Cations (XH^+^, X = S, Se, Te). Dalton Trans. 2017, 46 (10), 3095–3099. 10.1039/C7DT00186J. 28145552

[ref33] Vummaleti S. V. C.; Nelson D. J.; Poater A.; Gomez-Suarez A.; Cordes D. B.; Slawin A. M. Z.; Nolan S. P.; Cavallo L. What can NMR spectroscopy of selenoureas and phosphinidenes teach us about the p-accepting abilities of N-heterocyclic carbenes?. Chem. Sci. 2015, 6, 1895–1904. 10.1039/C4SC03264K. 29449918 PMC5812343

[ref34] Barnett C.; Cole M. L.; Harper J. B. A Dual NMR Probe Approach to Understanding the Electronic Properties of *N*-Heterocyclic Carbenes. Chem. Methods 2021, 1 (8), 374–381. 10.1002/cmtd.202100043.

[ref35] Kahnert S. R.; Pruschinski L.; Schmidt A. ^77^Se NMR spectroscopy of selenium adducts of N-heterocyclic carbenes. Adv. Heterocycl. Chem. 2025, 145, in print 10.1016/bs.aihch.2024.10.004.

[ref36] Pruschinski L.; Namyslo J. C.; Schmidt A. Anionic N-Heterocyclic Carbenes from Mesoionic Imidazolium-4-pyrrolides: The Influence of Substituents, Solvents, and Charge on their ^77^Se NMR Chemical Shifts. J. Org. Chem. 2024, 89, 15003–15019. 10.1021/acs.joc.4c01732. 39360676 PMC11494649

[ref37] Luthra N. P.; Boccanfuso A. M.; Dunlap R. B.; Odom J. D. Studies in selenium-77 and tellurium-125 nuclear magnetic resonance. Substituent effects and polarizability. J. Organomet. Chem. 1988, 354, 51–62. 10.1016/0022-328X(88)80638-7.

[ref38] Prasad C. D.; Balkrishna S. J.; Kumar A.; Bhakuni B. S.; Shrimali K.; Biswas S.; Kumar S. Transition-metal-free synthesis of unsymmetrical diaryl chalcogenides from arenes and diaryl dichalcogenides. J. Org. Chem. 2013, 78, 1434–1443. 10.1021/jo302480j. 23327334

[ref39] Hellwig Bartz R.; dos Santos Hellwig P.; Perin G.; Beluzzo Iarocz L. E.; Mababeni A.; Orian L.; Santos Silva M. Solvent effect on the 77Se NMR chemical shifts of diphenyl diselenides. New J. Chem. 2024, 48, 2971–2978. 10.1039/D3NJ05149H.

[ref40] Sultane P. R.; Ahumada G.; Janssen-Müller D.; Bielwaski C. W. Cyclic (aryl)(Amido)Carbenes: NHCs with Triplet-like Reactivity. Angew. Chem., Int. Ed. 2019, 58, 16320–16325. 10.1002/anie.201910350. 31461555

[ref41] Wong T. C.; Guziec F. S. Jr.; Moustakis C. A. Oxygen-17 and selenium-77 nuclear magnetic resonance of carbonyl and selenocarbonyl compounds. Correlation of oxygen-17 and selenium-77 chemical shifts. J. Chem. Soc. Perkin Trans II 1983, 1471–1475. 10.1039/p29830001471.

[ref42] Hölzel T.; Otto M.; Buhl H.; Ganter C. An Extremely Electron Poor Cationic Triazoliumylidene N-Heterocyclic Carbene: Experimental and Computational Studies. Organometallics 2017, 36, 4443–4450. 10.1021/acs.organomet.7b00670.

[ref43] Mummel S.; Lederle F.; Hübner E. G.; Namyslo J. C.; Nieger M.; Schmidt A. Sydnone Methides: Intermediates between Mesoionic Compounds and Mesoionic N-Heterocyclic Olefins. Eur. J. Org. Chem. 2023, 26, e202300216 10.1002/ejoc.202300216.

[ref44] Kahnert S. R.; Namyslo J. C.; Rissanen K.; Nieger M.; Schmidt A. Imidazolium Dicyanomethylides as N-Ylide Precursors of Anionic N-Heterocyclic Carbenes. Eur. J. Org. Chem. 2024, 27, e202400163 10.1002/ejoc.202400163.

[ref45] Ostapiuk Y. V.; Ostapiuk M. Y.; Barabash O. V.; Kravets M.; Herzberger C.; Namyslo J. C.; Obushak M. D.; Schmidt A. One pot syntheses of substituted 2-aminothiazoles and 2-aminoselenazoles via Meerwein arylation of alkyl vinyl ketones. Synthesis 2022, 54, 3658–3666. 10.1055/s-0041-1738070.

[ref46] aAppel S.; Brüggemann P.; Ganter C. A Tropylium Annulated N-Heterocyclic Carbene. Chem. Commun. 2020, 56 (63), 9020–9023. 10.1039/D0CC04482B. 32639486

[ref47] aRamsden C. A.; Ozimiński W. P. Quantitative Index of the Relative Ease of Formation and σ-Bonding Strength of N-Heterocyclic Carbenes. J. Org. Chem. 2016, 81 (21), 10295–10301. 10.1021/acs.joc.6b01304. 27366937

[ref48] Reichardt C. L. Solvents and Solvent Effects in Organic Chemistry. Wiley 2002, 10.1002/3527601791.

[ref49] aMartin M.; Fauquignon C. Étude de Complexations Par Résonance Magnétique Nucléaire - Déplacement Chimique et Basicités. Ann. Phys. 1962, 13 (7), 35–55. 10.1051/anphys/196213070035.

[ref50] Grineva A. A.; Filippov O. A.; Nefedov S. E.; Lugan N.; César V.; Valyaev D. A. Direct Access to IMes^F^ and IMes^F^_2_ by Electrophilic Fluorination of Abnormal N-Heterocyclic Carbenes. Organometallics 2019, 38 (11), 2330–2337. 10.1021/acs.organomet.9b00151.

