# Supplementary material for: Studies on the Effect of Positive and Negative Charges on the 77Se NMR Shifts of Selenones and Selenenyls of N-heterocyclic Carbenes of Imidazolium-4,5-dicarboxylates
Source: J Org Chem. 2025 Feb 4;90(6):2201–13. doi: 10.1021/acs.joc.4c02581 (PMC11833865; doi:10.1021/acs.joc.4c02581)

Supporting Information

Studies on the effect of positive and negative charges on the ^{77}Se NMR shifts of selenones and selenenyls of N-heterocyclic carbenes of imidazolium-4,5-dicarboxylates.

Lucas Pruschinski, Sean Ray Kahnert, Colin Herzberger, Jan C. Namyslo, and Andreas Schmidt*

Clausthal University of Technology

Institute of Organic Chemistry

Leibnizstrasse 6

D-38678 Clausthal-Zellerfeld

Germany

schmidt@ioc.tu-clausthal.de

Phone: +49-5323-723861

Table of Contents

1. Numberings...S3-6

2. NMR spectra..S7-74

3. DFT calculations (HOMO/LUMO coefficients and orbital energies)..S75-141

<u>Numberings</u>

Diisopropyl-1-methyl-1*H*-imidazole-4,5-dicarboxylate 10b.

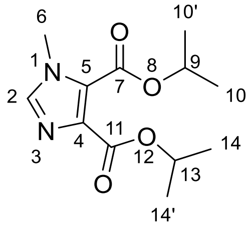

4,5-Bis(isopropoxycarbonyl)-1,3-dimethyl-1*H*-imidazolium iodide 11b.

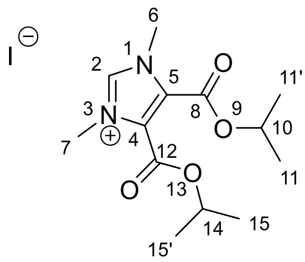

Diethyl-1,3-dimethyl-2-selenoxo-2,3-dihydro-1*H*-imidazole-4,5-dicarboxylate 12a.

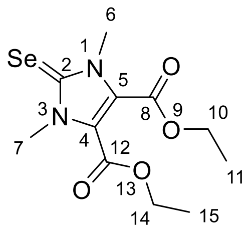

Diisopropyl-1,3-dimethyl-2-selenoxo-2,3-dihydro-1*H*-imidazole-4,5-dicarboxylate 12b.

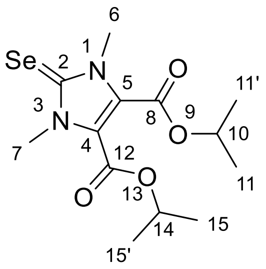

Diethyl-1,3-dimethyl-2-thioxo-2,3-dihydro-1*H*-imidazole-4,5-dicarboxylate 12c.

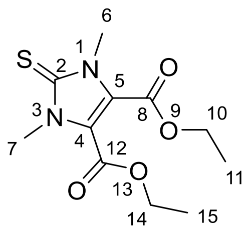

Diisopropyl-1,3-dimethyl-2-thioxo-2,3-dihydro-1*H*-imidazole-4,5-dicarboxylate 12d.

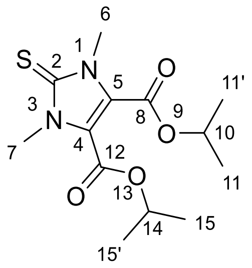

4,5-Bis(ethoxycarbonyl)-1,3-dimethyl-2-methylselanyl-1*H*-imidazolium tetraphenylborate 13a.

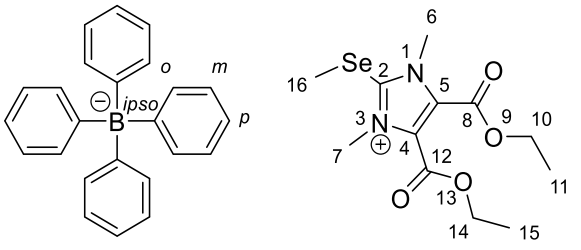

4,5-Bis(isopropoxycarbonyl)-1,3-dimethyl-2-methylselanyl-1*H*-imidazolium tetraphenylborate 13b.

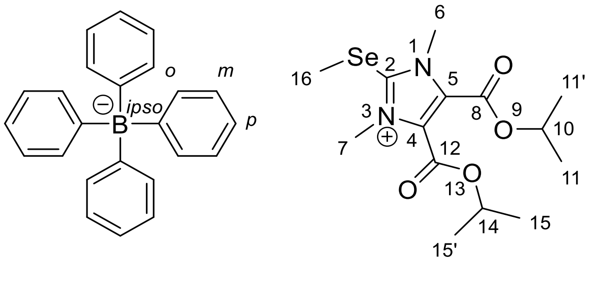

4,5-Bis(ethoxycarbonyl)-1,3-dimethyl-2-methylthionyl-1*H*-imidazolium tetraphenylborate 13c.

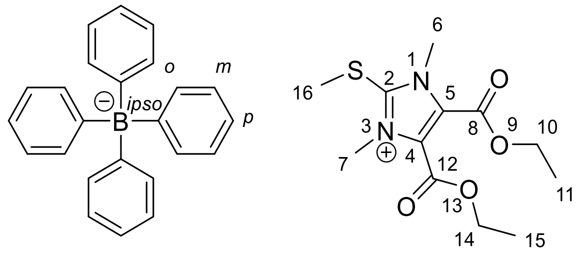

4,5-Bis(isopropoxycarbonyl)-1,3-dimethyl-2-methylthionyl-1*H*-imidazolium tetraphenylborate 13d.

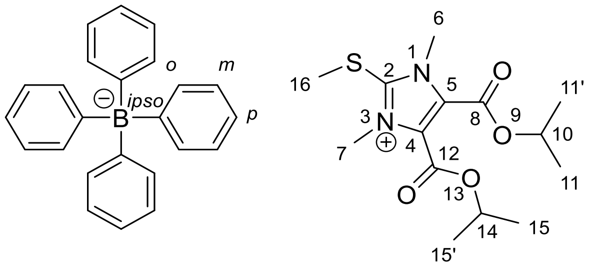

4,5-Bis(ethoxycarbonyl)-1,3-dimethyl-2-methylselanyl-1*H*-imidazolium hexafluorophosphate 13e.

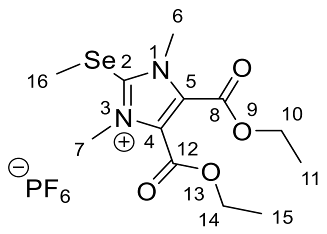

4,5-Bis(ethoxycarbonyl)-1,3-dimethyl-2-methylthionyl-1*H*-imidazolium hexafluorophosphate 13f.

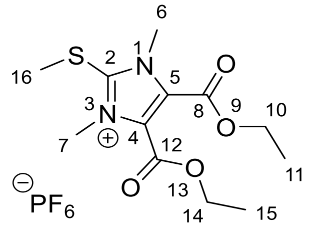

Disodium 1,3-dimethyl-2-selenoxo-2,3-dihydro-1*H*-imidazole-4,5-dicarboxylate 14a.

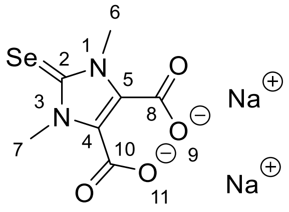

Disodium 1,3-dimethyl-2-thioxo-2,3-dihydro-1*H*-imidazole-4,5-dicarboxylic acid 14b.

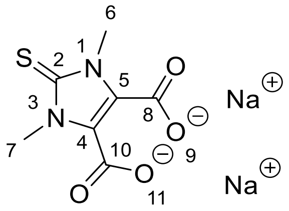

Dimethyl-1,3-dimethyl-2-selenoxo-2,3-dihydro-1*H*-imidazole-4,5-dicarboxylate 15.

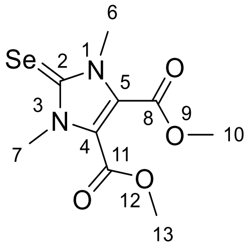

5-(Ethoxycarbonyl)-1,3-dimethyl-2-selenoxo-2,3-dihydro-1*H*-imidazole-4-carboxylic acid 16a.

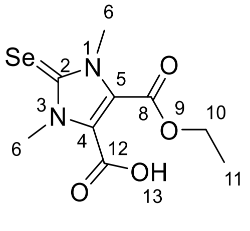

5-(Isopropoxycarbonyl)-1,3-dimethyl-2-selenoxo-2,3-dihydro-1*H*-imidazole-4-carboxylic acid 16b.

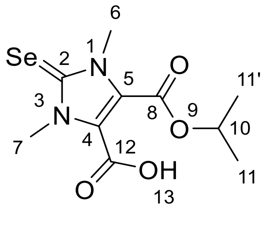

5-(Ethoxycarbonyl)-1,3-dimethyl-2-thioxo-2,3-dihydro-1*H*-imidazole-4-carboxylic acid 16c.

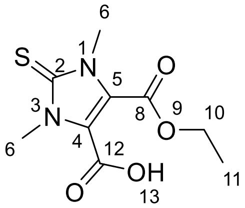

5-(Isopropoxycarbonyl)-1,3-dimethyl-2-thioxo-2,3-dihydro-1*H*-imidazole-4-carboxylic acid 16d.

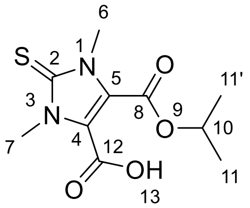

Sodium 5-(Ethoxycarbonyl)-1,3-dimethyl-2-selenoxo-2,3-dihydro-1*H*-imidazole-4-carboxylate 17a.

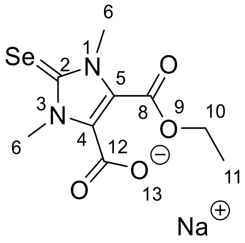

Sodium 5-(Isopropoxycarbonyl)-1,3-dimethyl-2-selenoxo-2,3-dihydro-*1H*-imidazole-4-carboxylate 17b

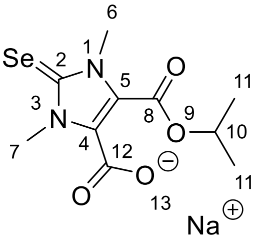

NMR Spectra

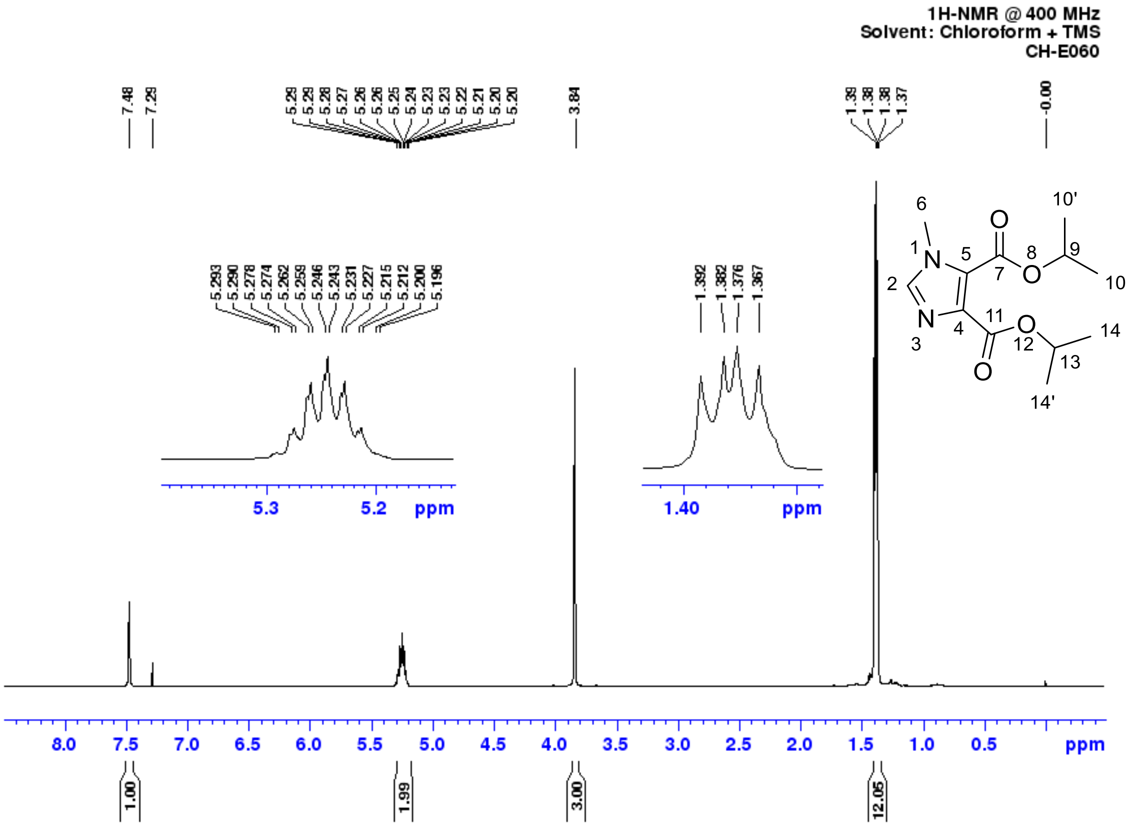

Figure S1: ^{1}H NMR spectrum of **10b** in $CDCl_3$.

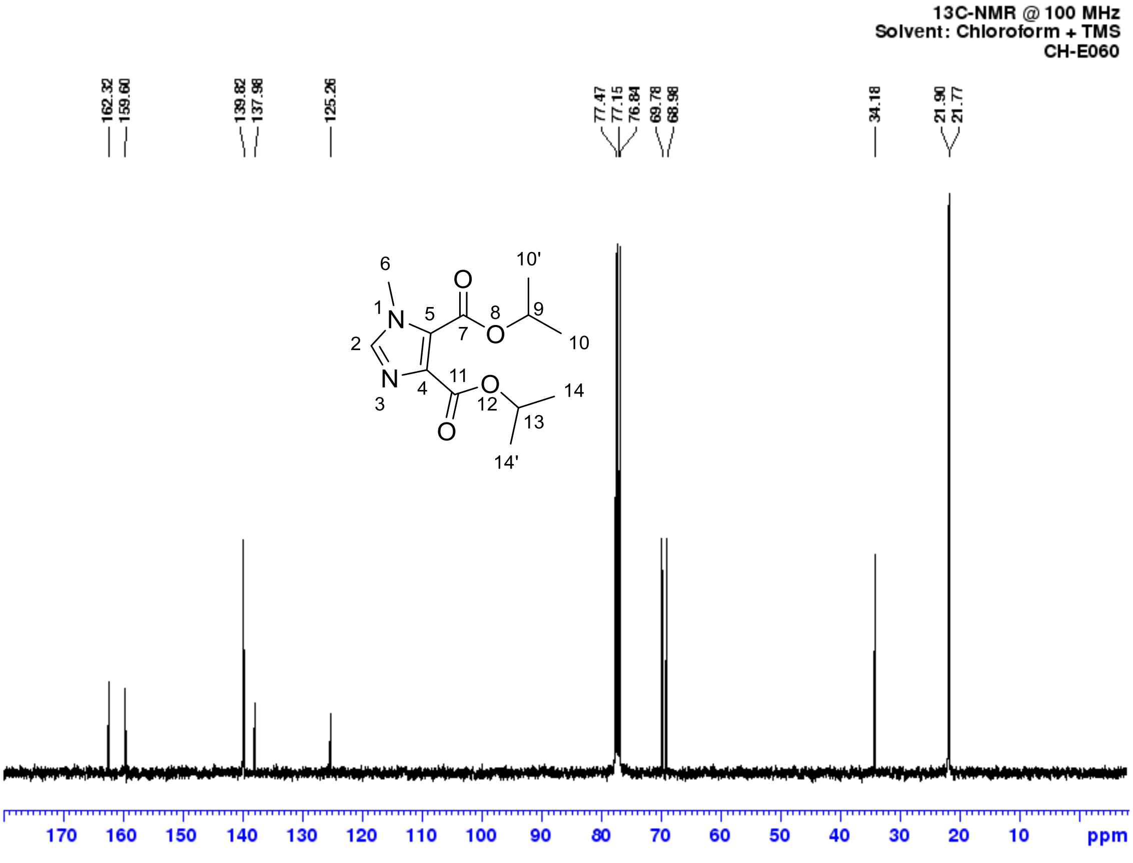

Figure S2: $^{13}C\{^{1}H\}$ NMR spectrum of **10b** in $CDCl_3$.

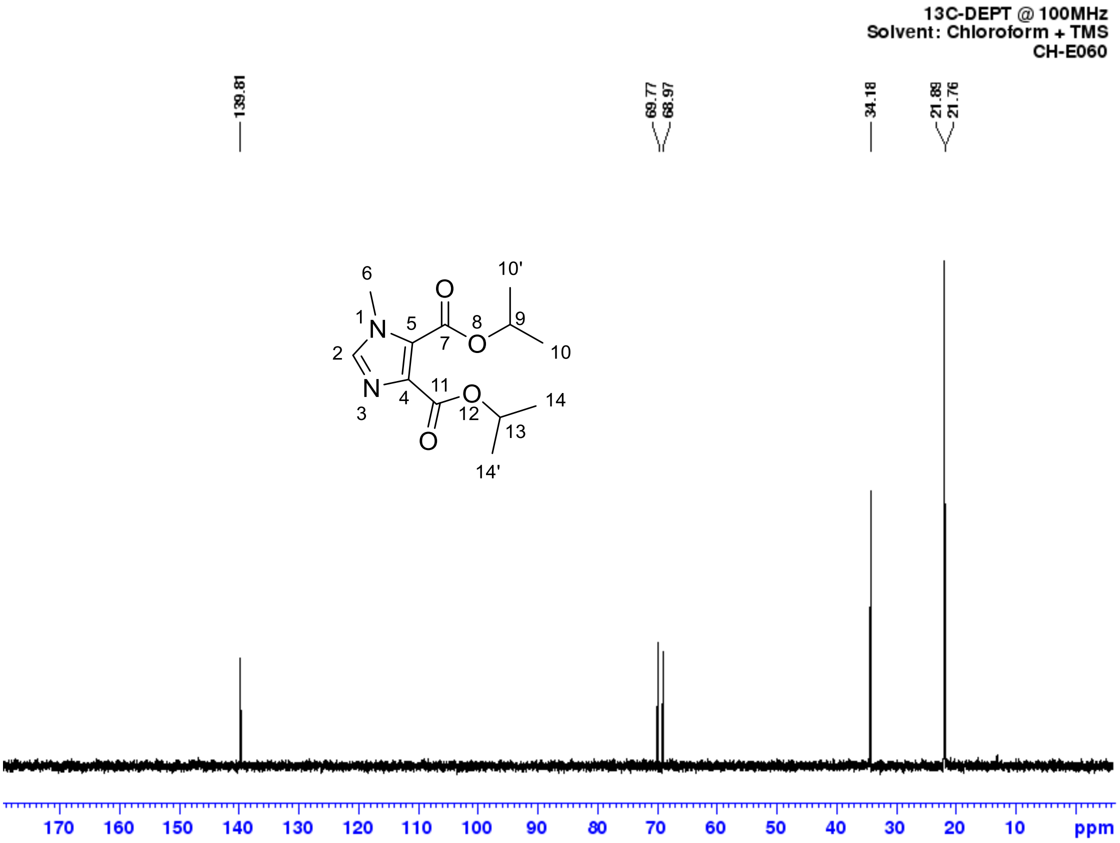

Figure S3: $^{13}C\{^1H\}$ DEPT spectrum of **10b** in $CDCl_3$.

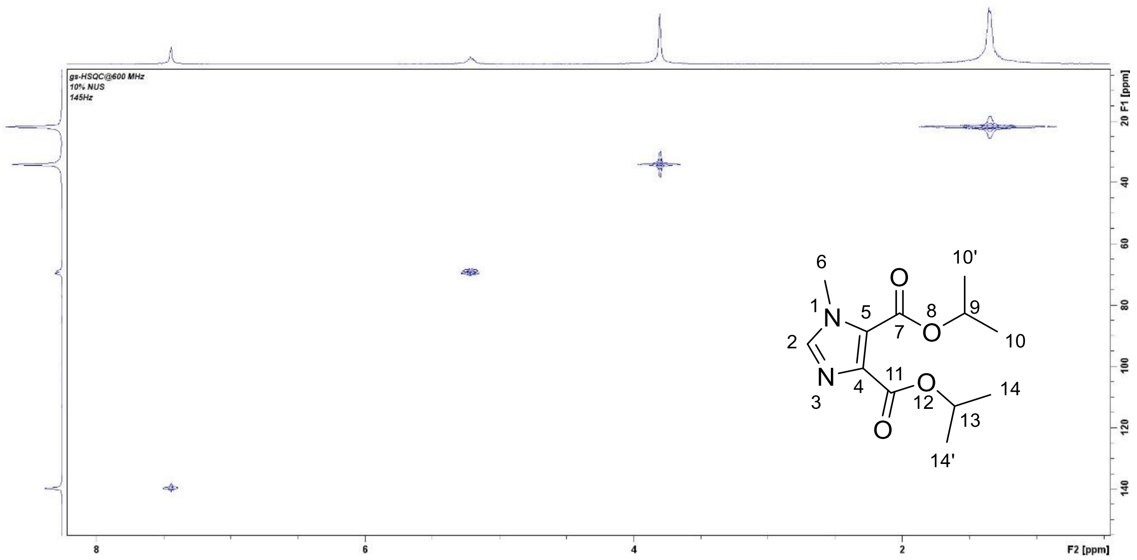

Figure S4: HSQC NMR spectrum of **10b** in $CDCl_3$.

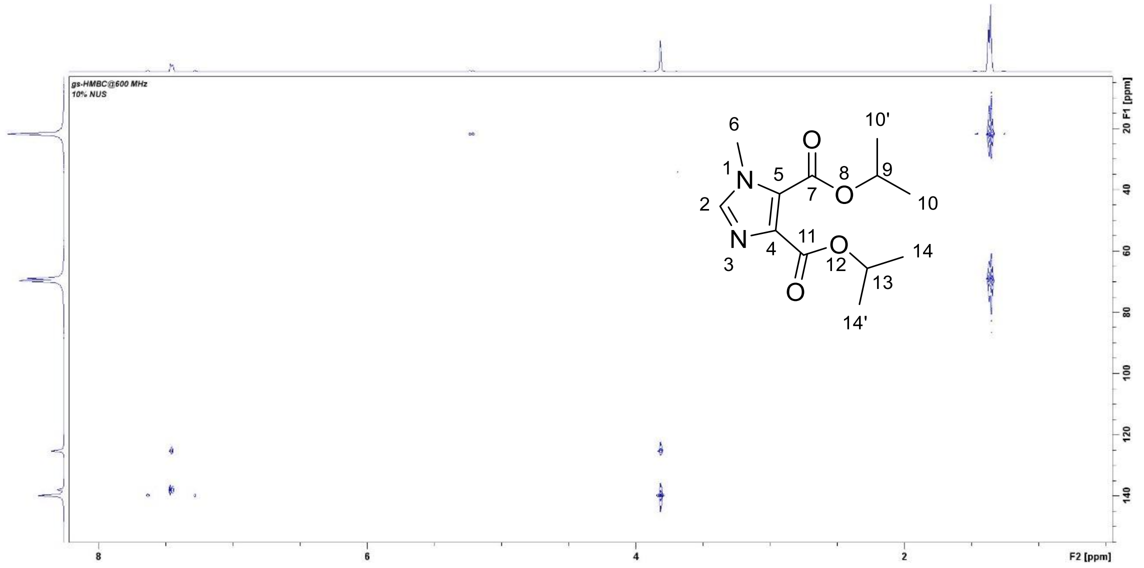

Figure S5: HMBC NMR spectrum of **10b** in $CDCl_3$.

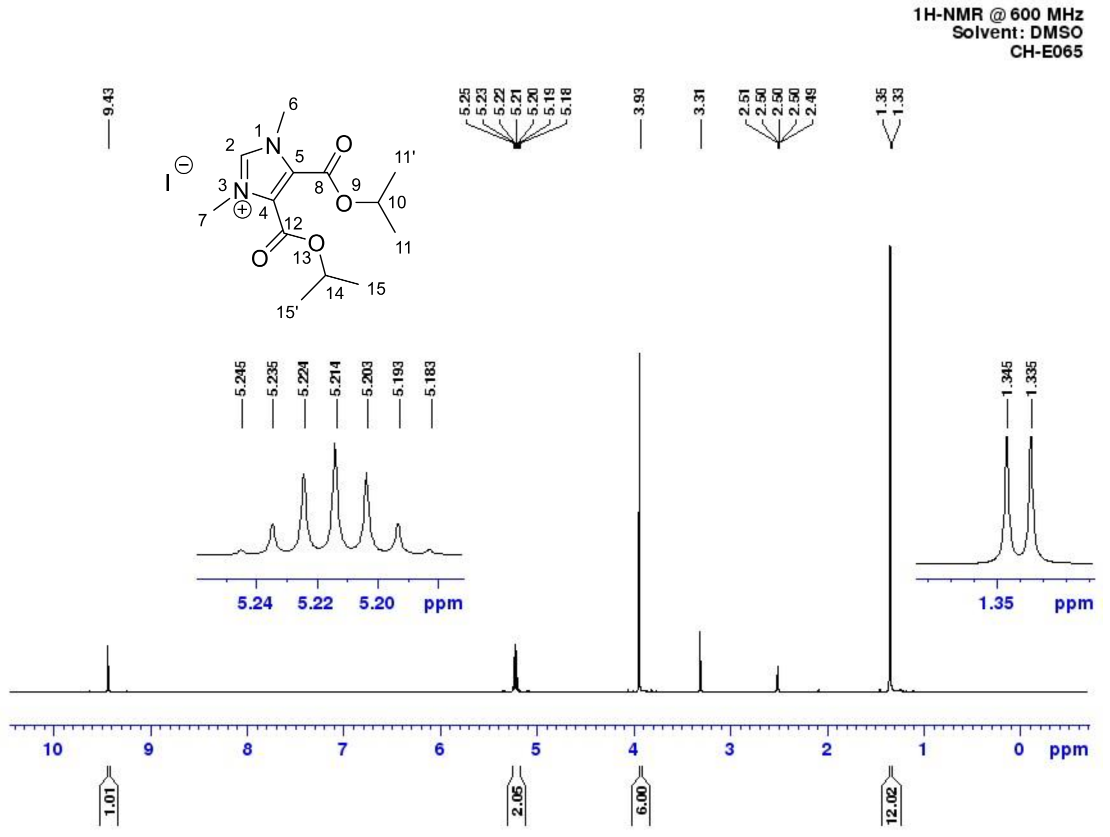

Figure S6: ^{1}H NMR spectrum of **11b** in DMSO-d6.

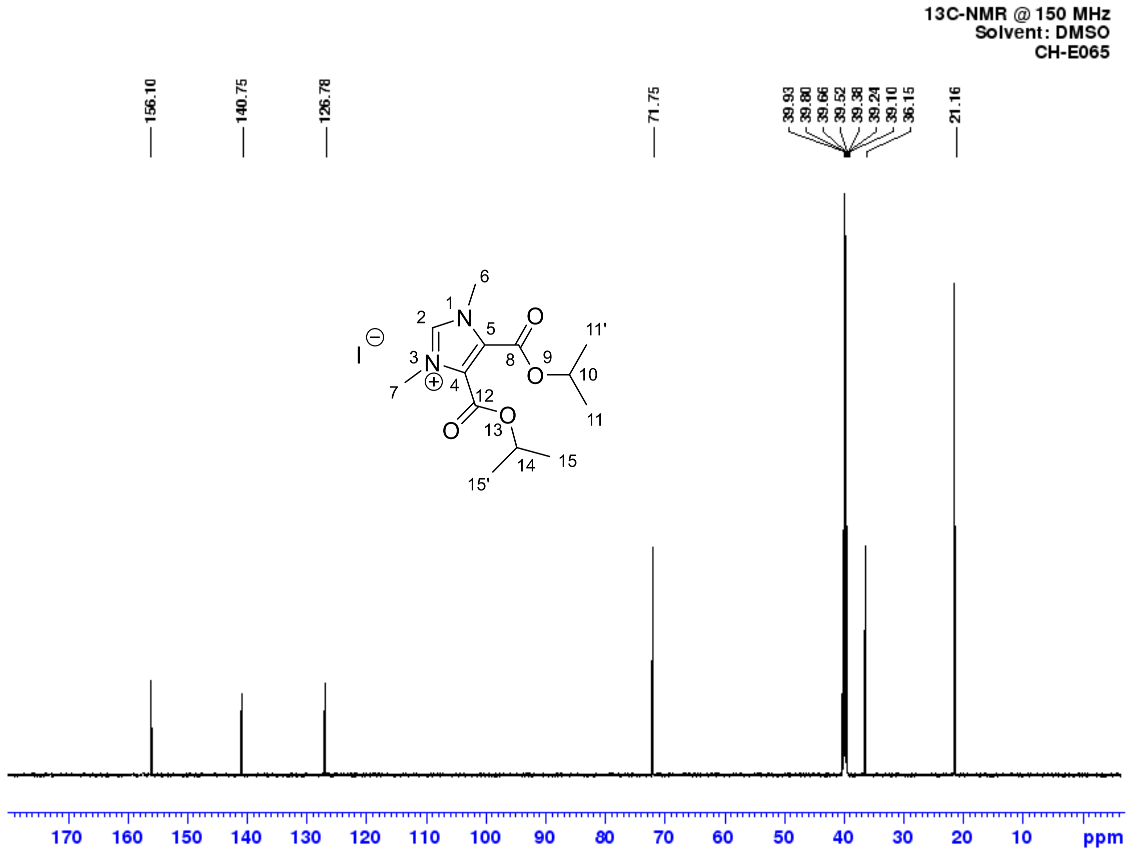

Figure S7: $^{13}C\{^{1}H\}$ NMR spectrum of **11b** in DMSO-d$_6$.

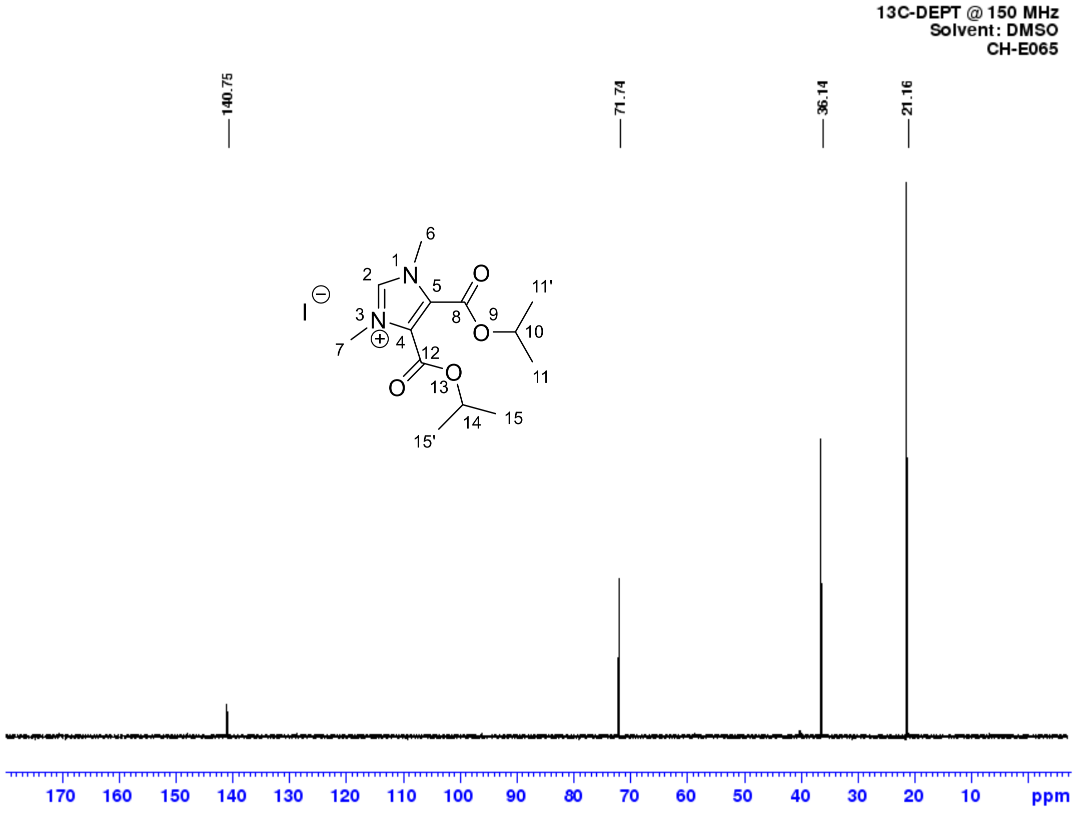

Figure S8: $^{13}C\{^{1}H\}$ DEPT NMR spectrum of **11b** in DMSO-d$_6$.

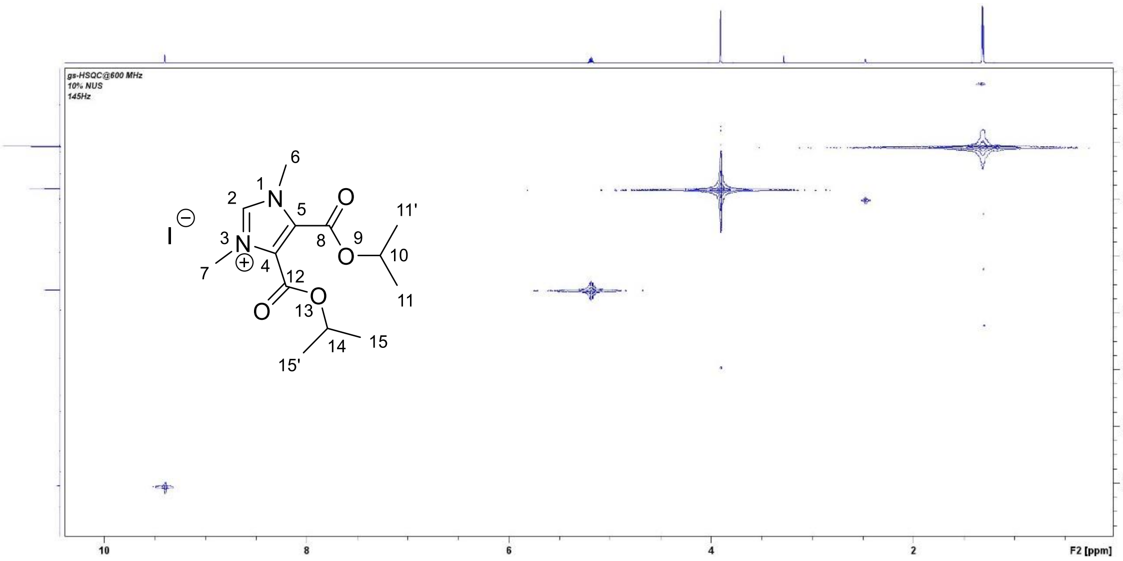

Figure S9: HSQC NMR spectrum of **11b** in DMSO-d$_6$**.**

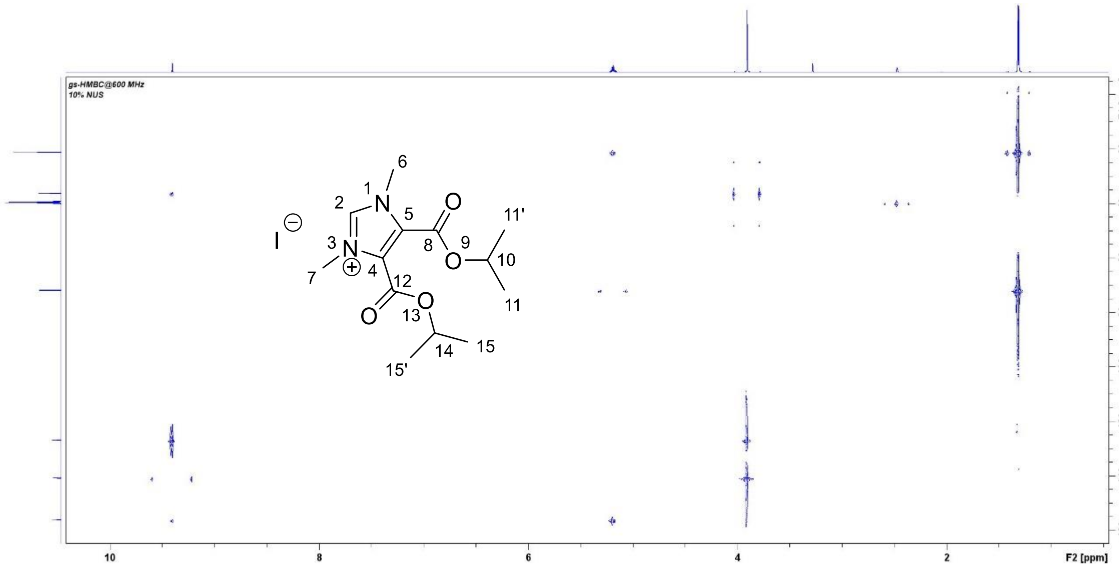

Figure S10: HMBC NMR spectrum of **11b** in DMSO-d$_6$.

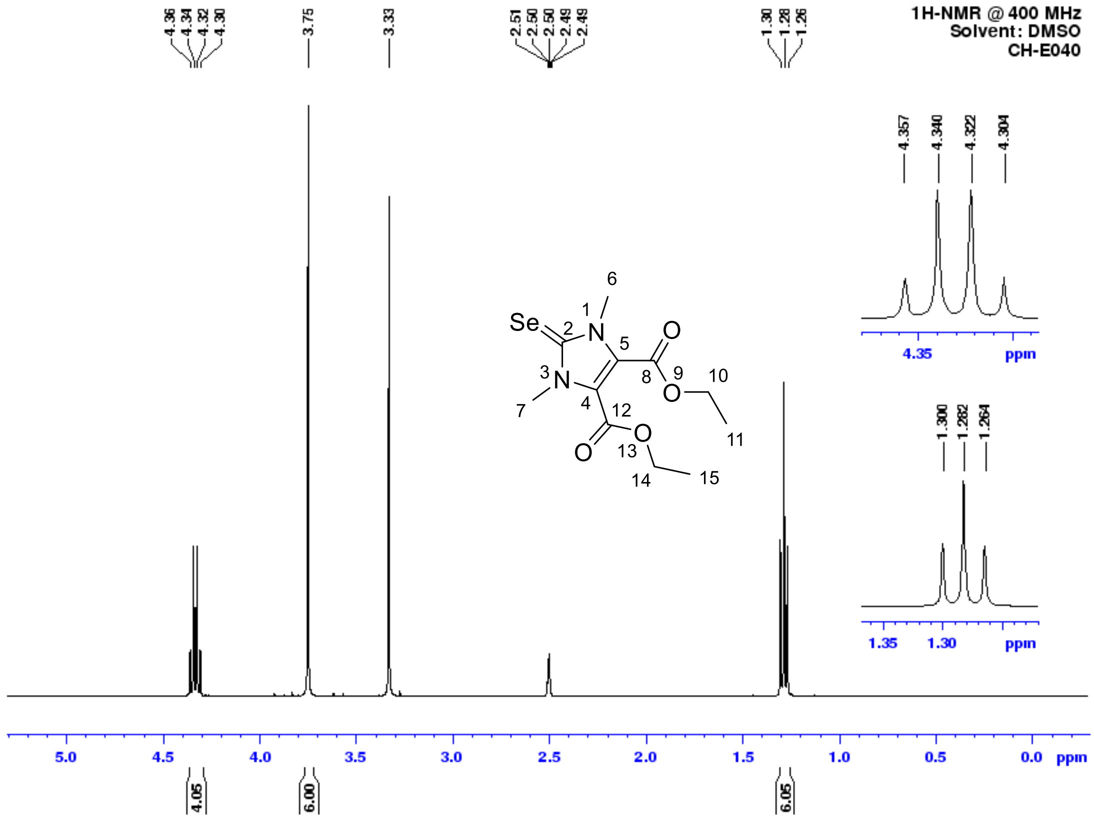

Figure S11: ^{1}H NMR spectrum of **12a** in DMSO-d_6.

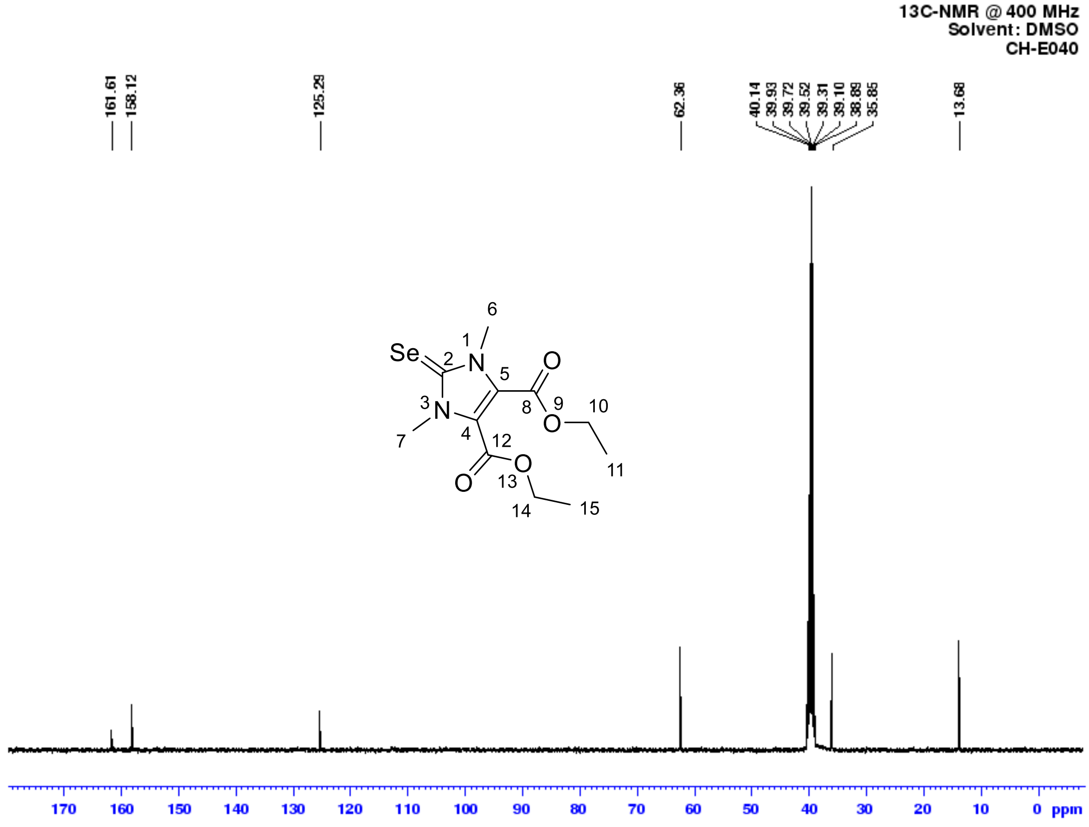

Figure S12: $^{13}C\{^{1}H\}$ NMR spectrum of **12a** in DMSO-d_6.

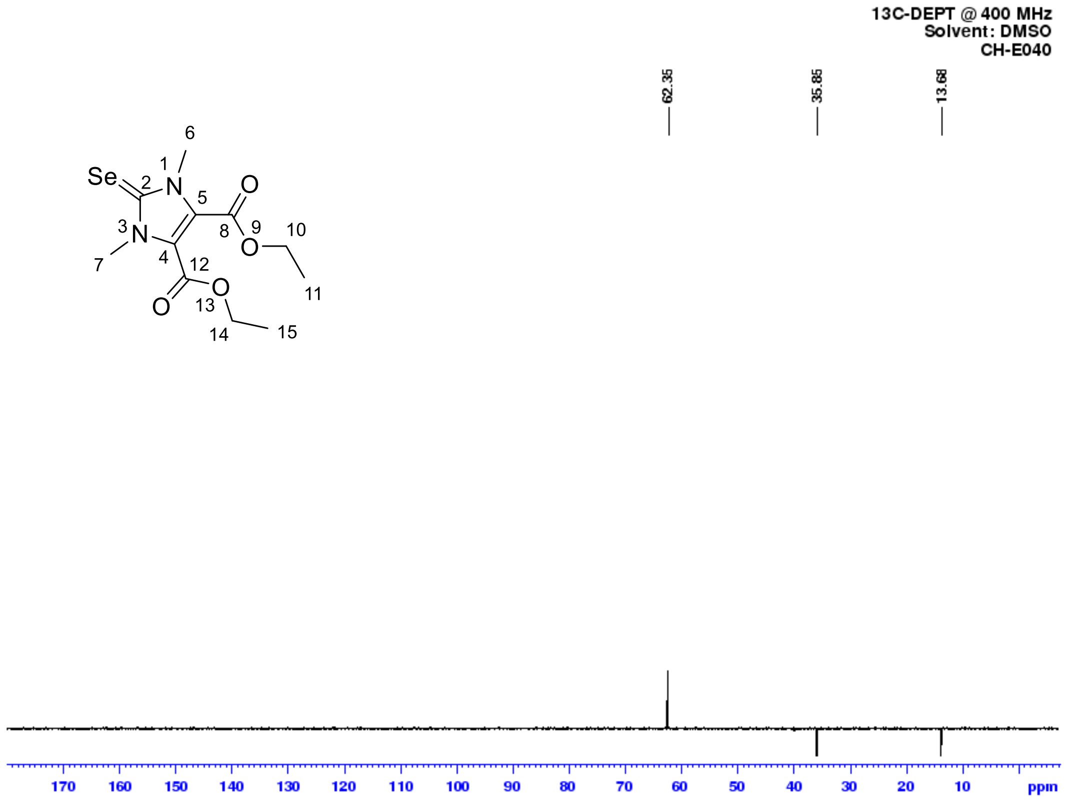

Figure S13: $^{13}C\{^{1}H\}$ DEPT NMR spectrum of **12a** in DMSO-d_6.

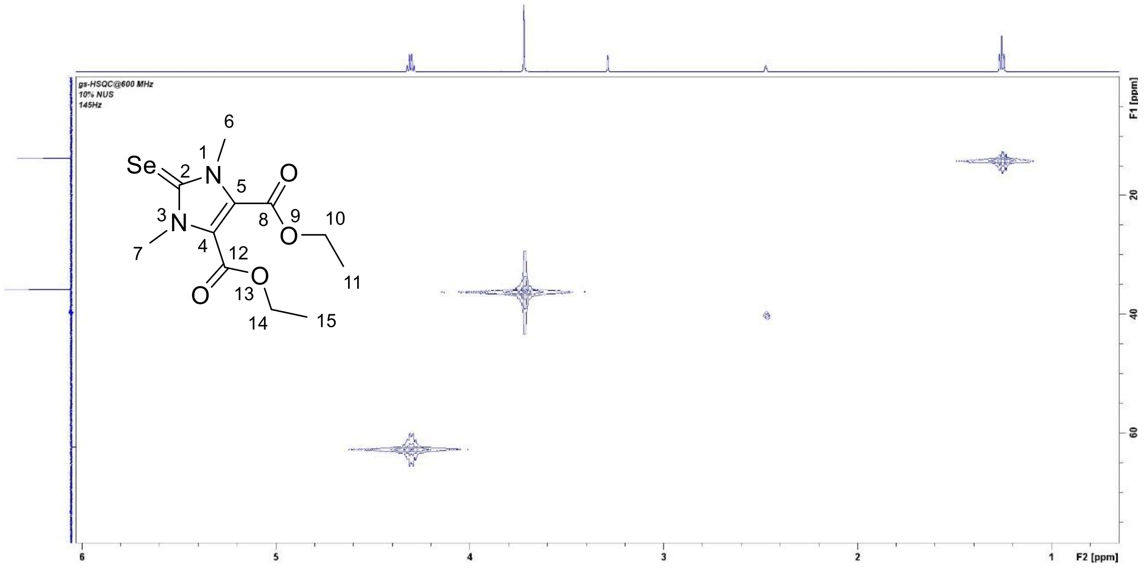

Figure S14: HSQC NMR spectrum of **12a** in DMSO-d_6.

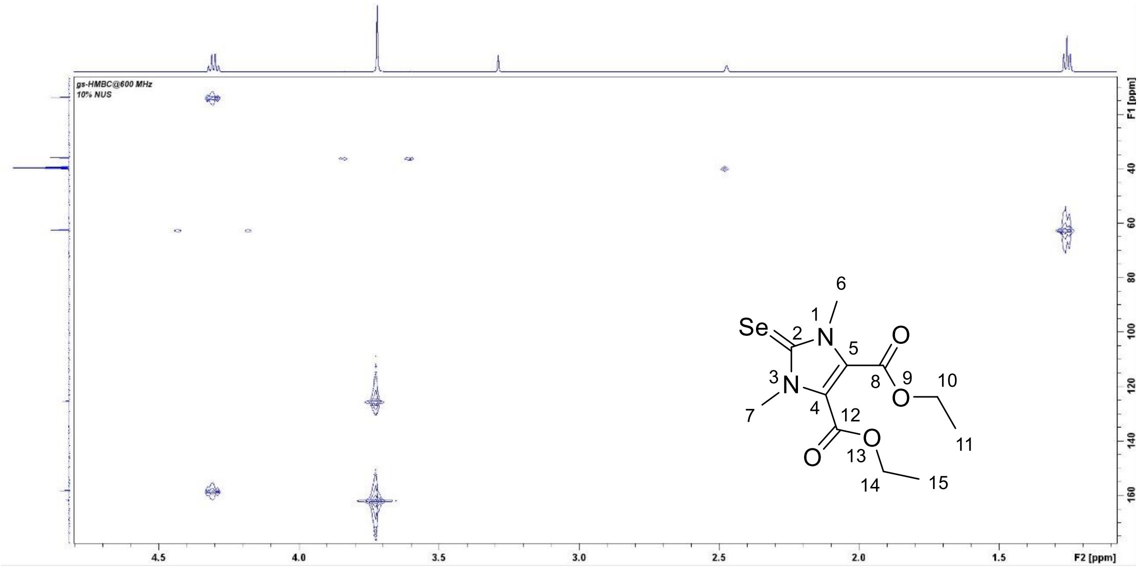

Figure S15: HMBC NMR spectrum of **12a** in DMSO-d$_6$.

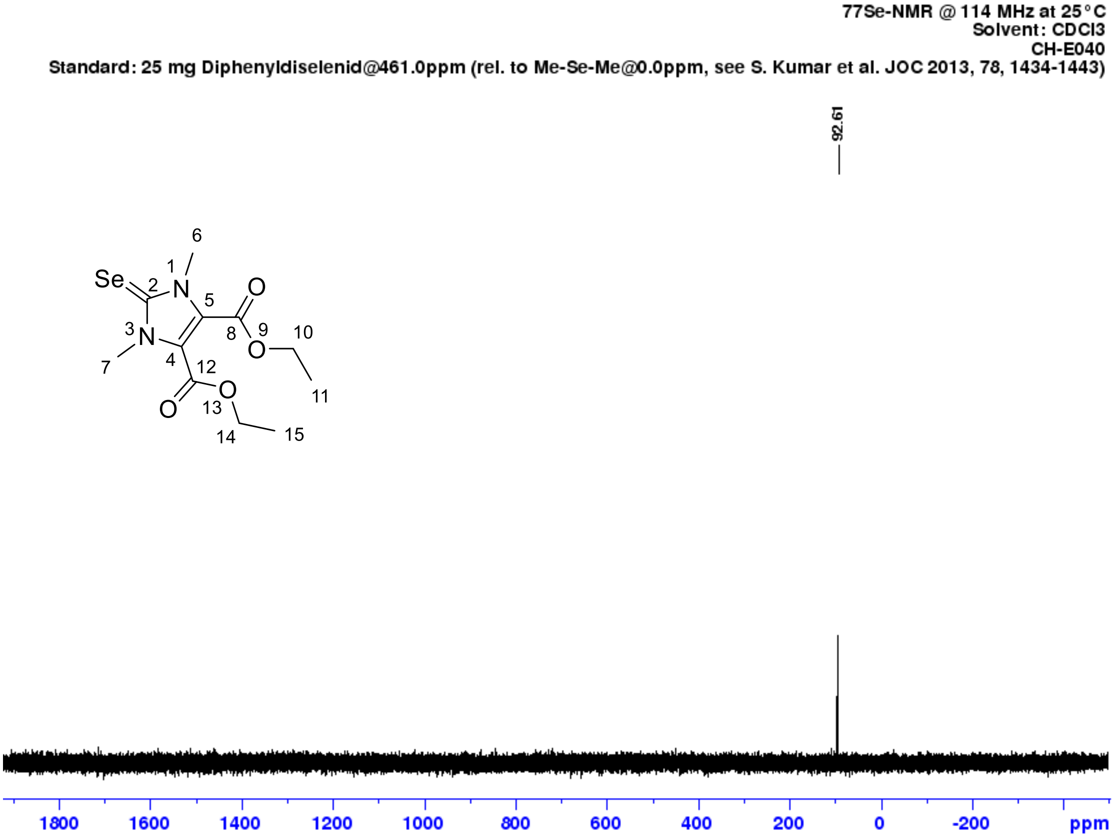

Figure S16: ^{77}Se NMR spectrum of **12a** in $CDCl_3$.

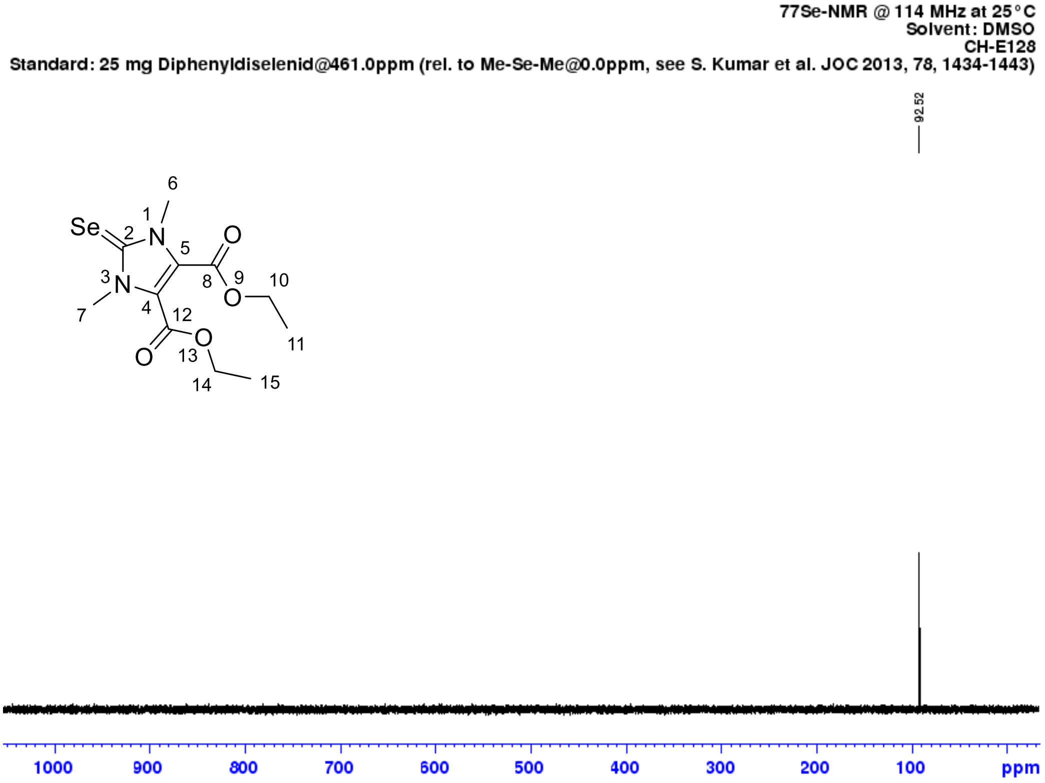

Figure S17: ^{77}Se NMR spectrum of **12a** in DMSO-d_6.

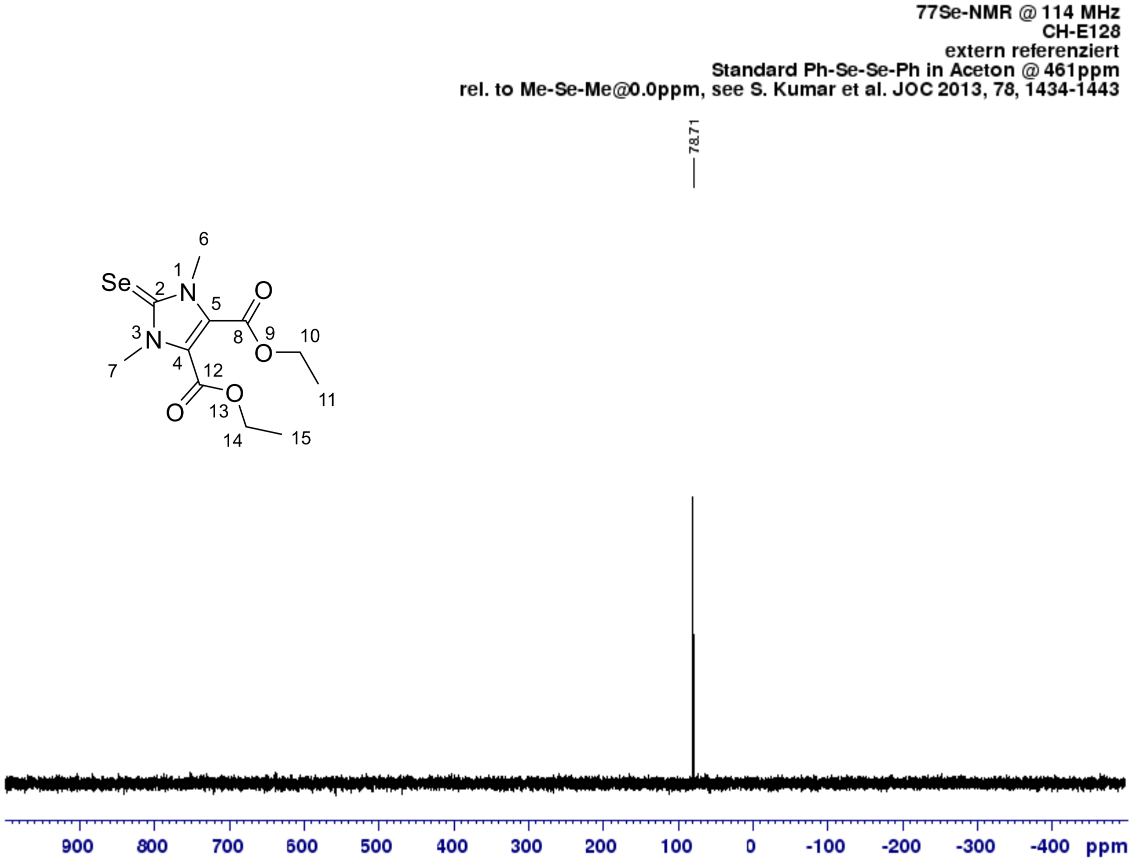

Figure S18: ^{77}Se NMR spectrum of **12a** in acetone-d_6.

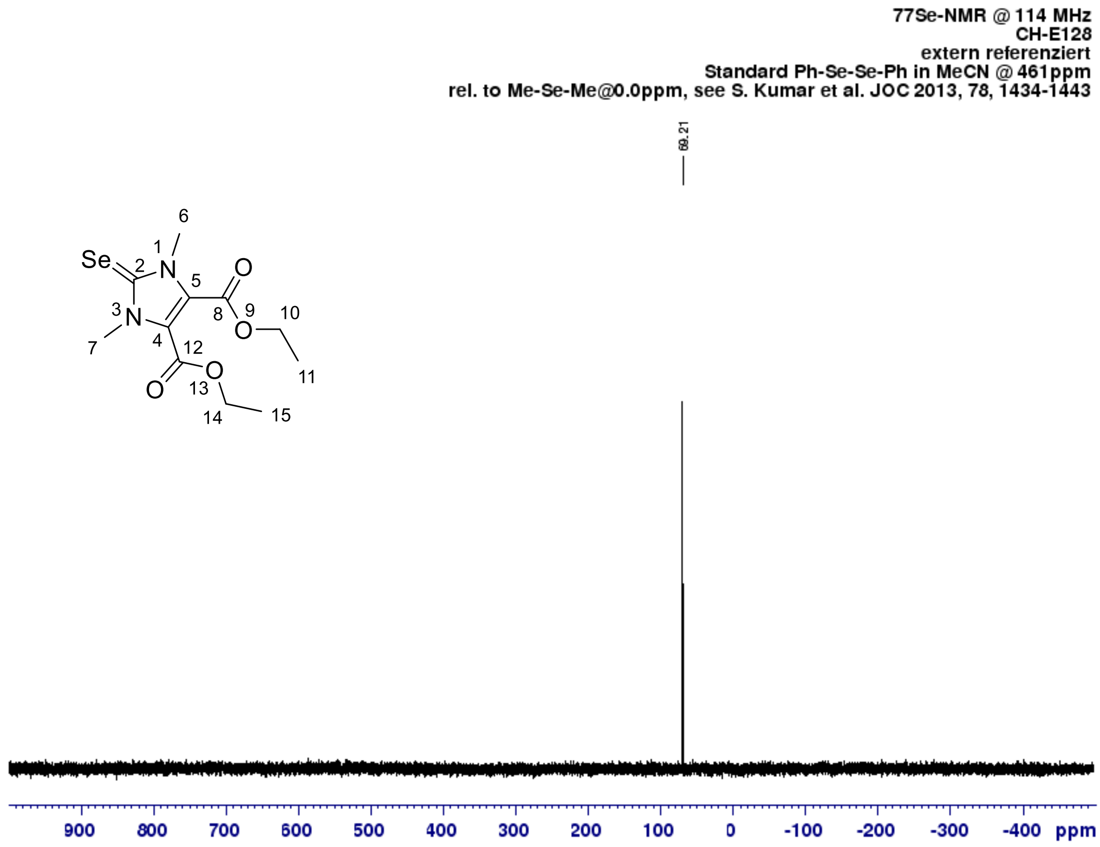

Figure S19: ^{77}Se NMR spectrum of **12a** in CD_3CN.

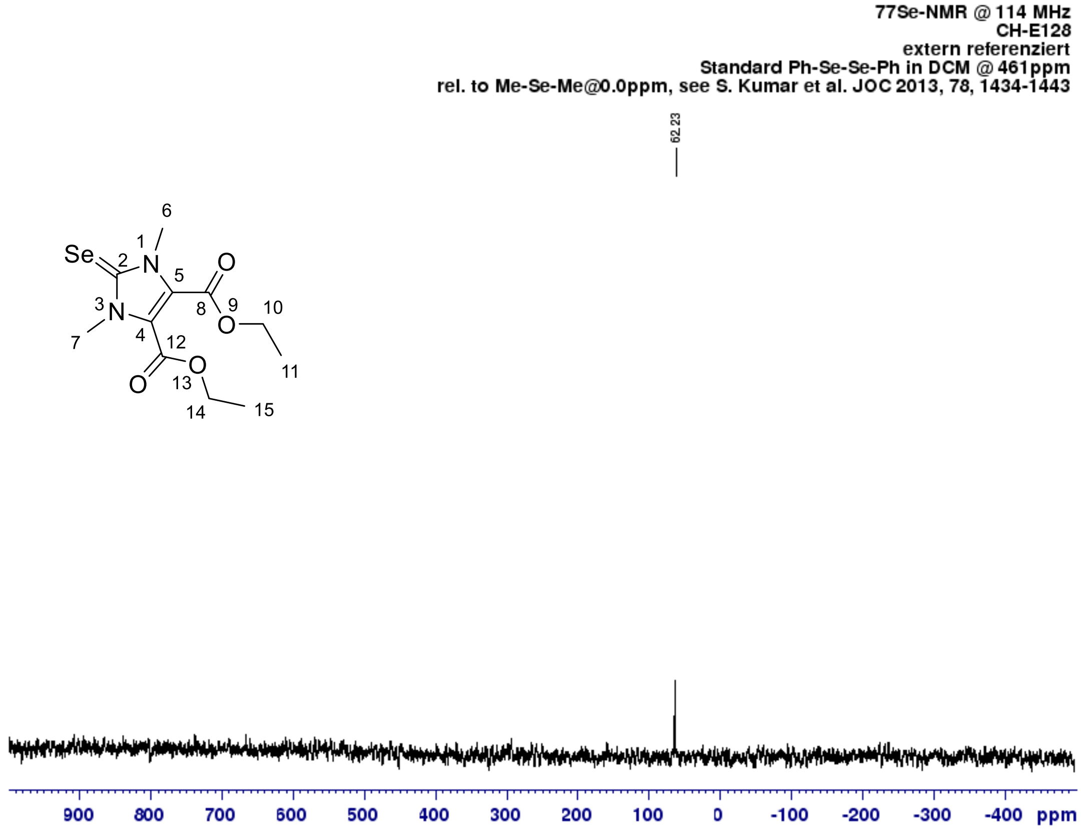

Figure S20: ^{77}Se NMR spectrum of **12a** in CD_2Cl_2.

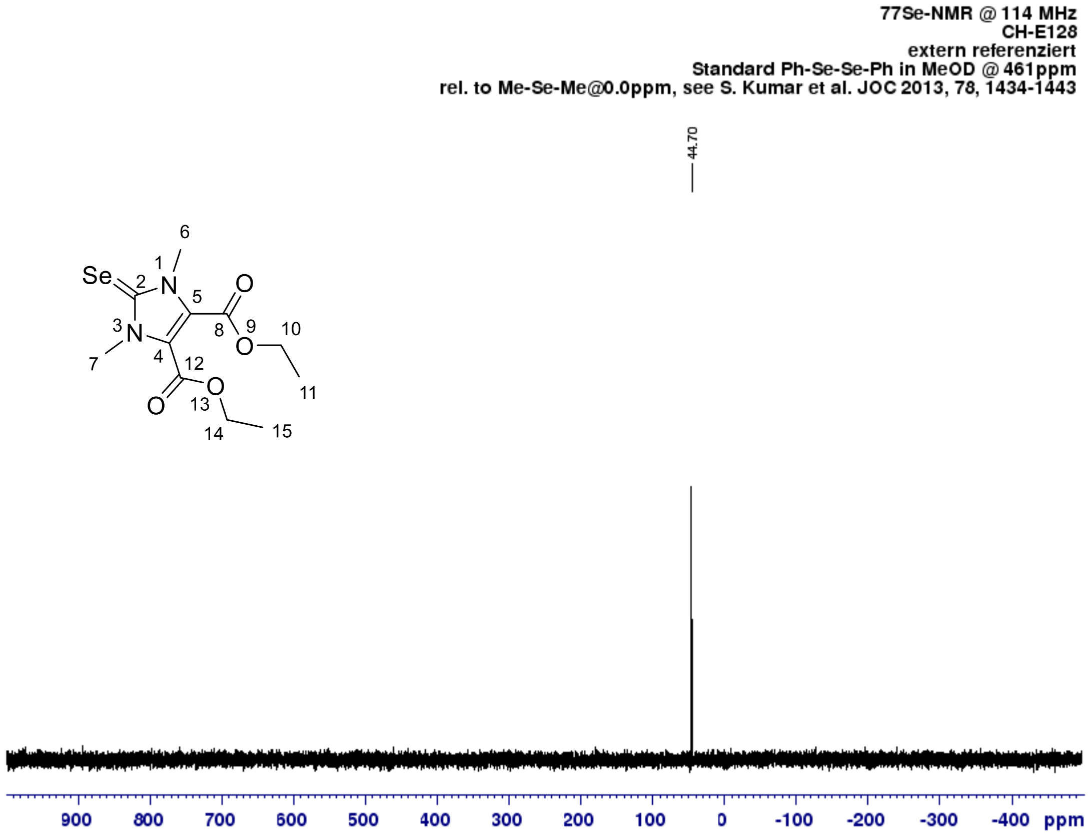

Figure S21: ^{77}Se NMR spectrum of **12a** in MeOD.

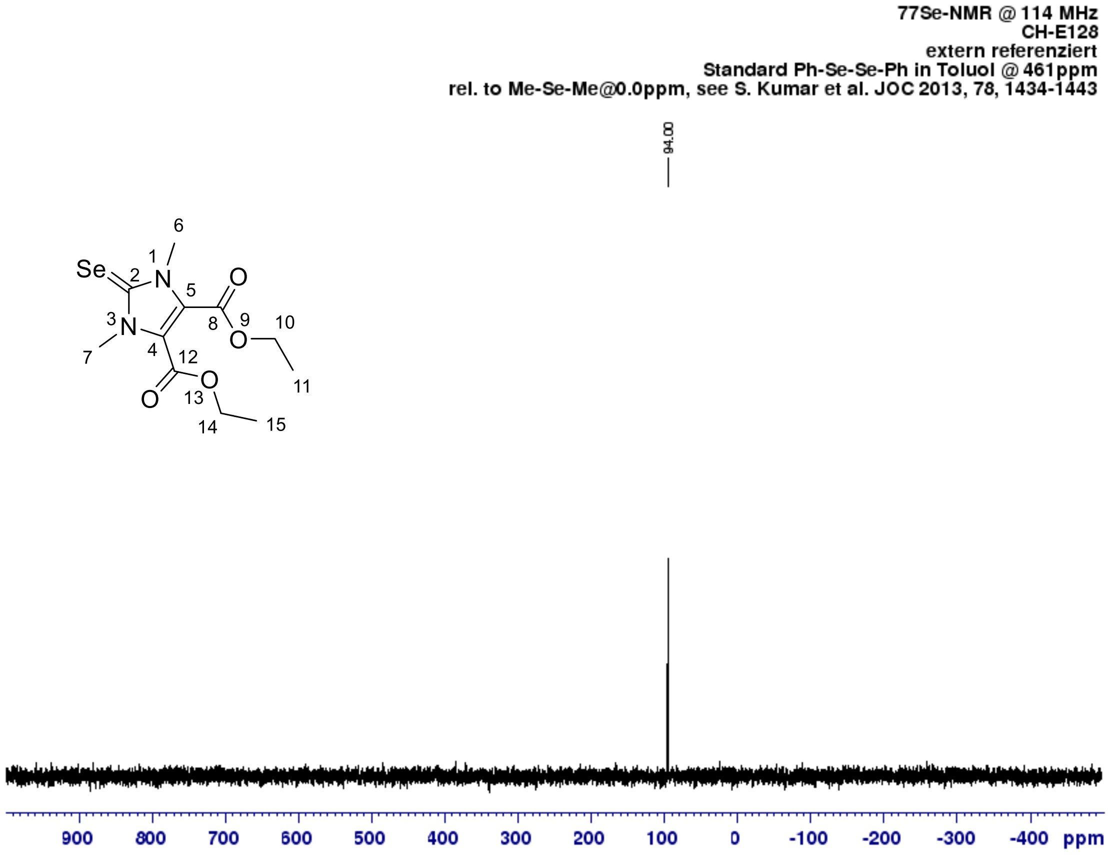

Figure S22: ^{77}Se NMR spectrum of **12a** in toluene-d_8.

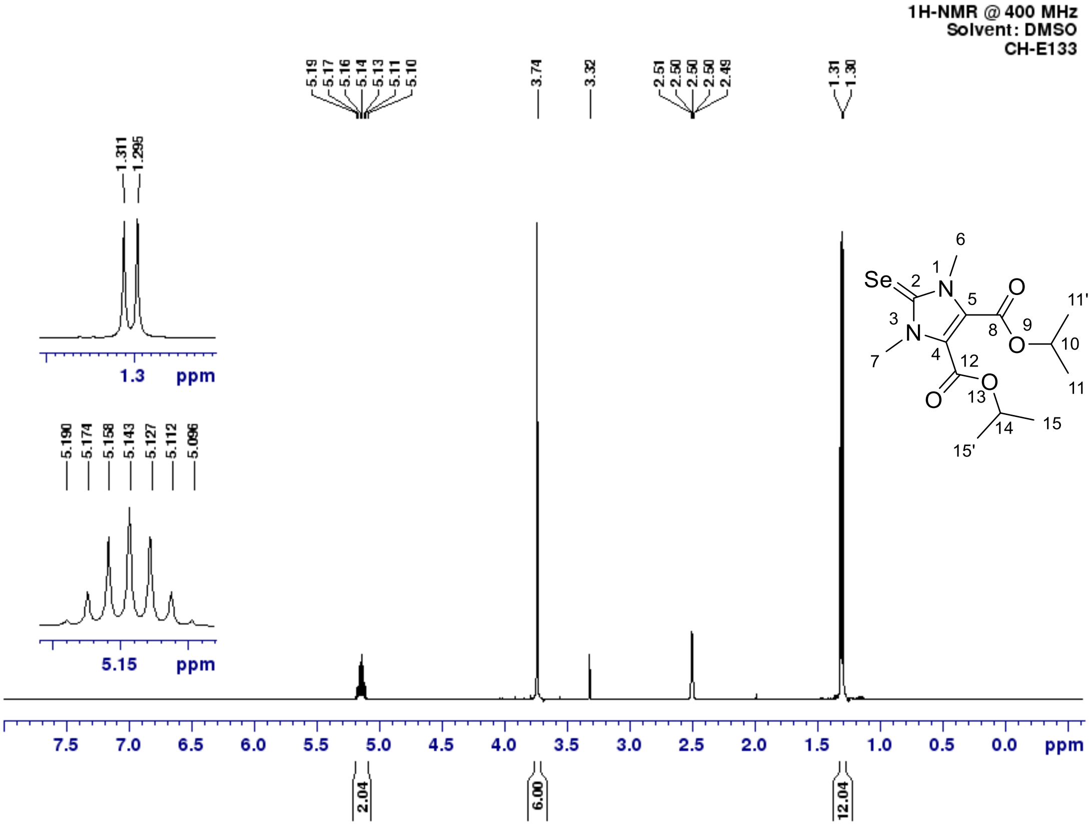

Figure S23: ^{1}H NMR spectrum of **12b** in DMSO-d$_6$.

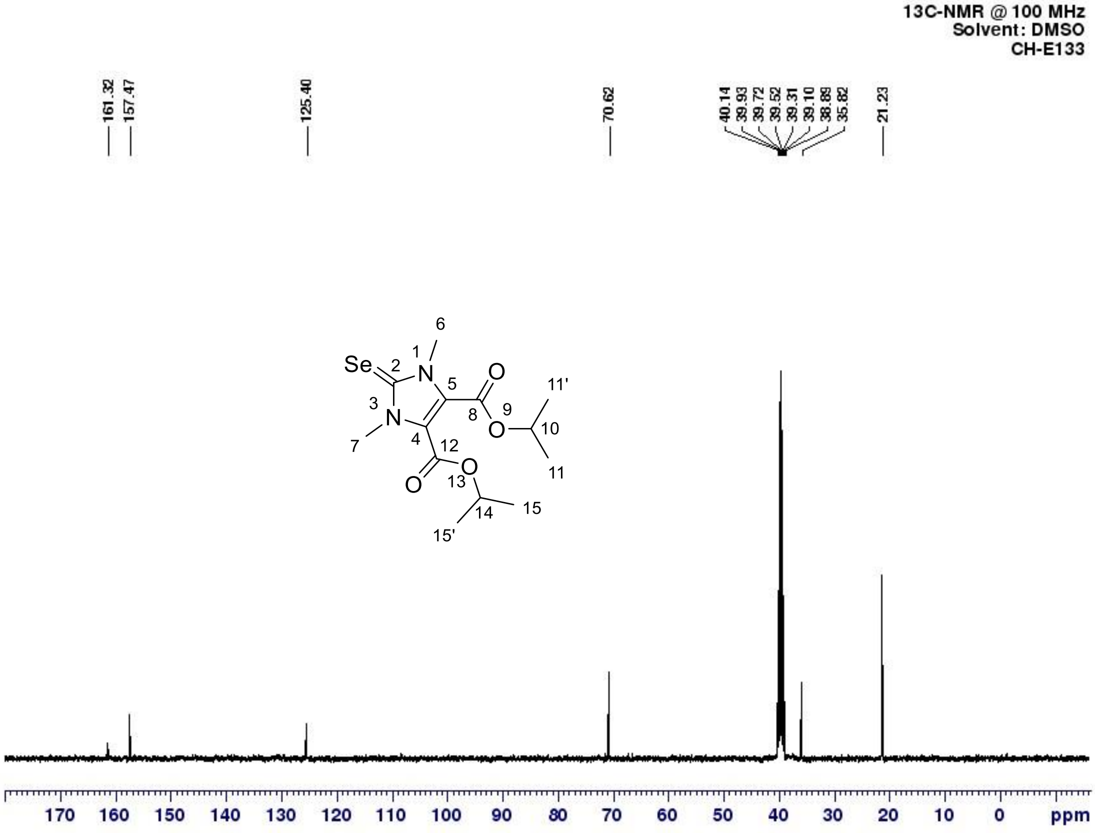

Figure S24: ^{13}C{^{1}H} NMR spectrum of **12b** in DMSO-d$_6$.

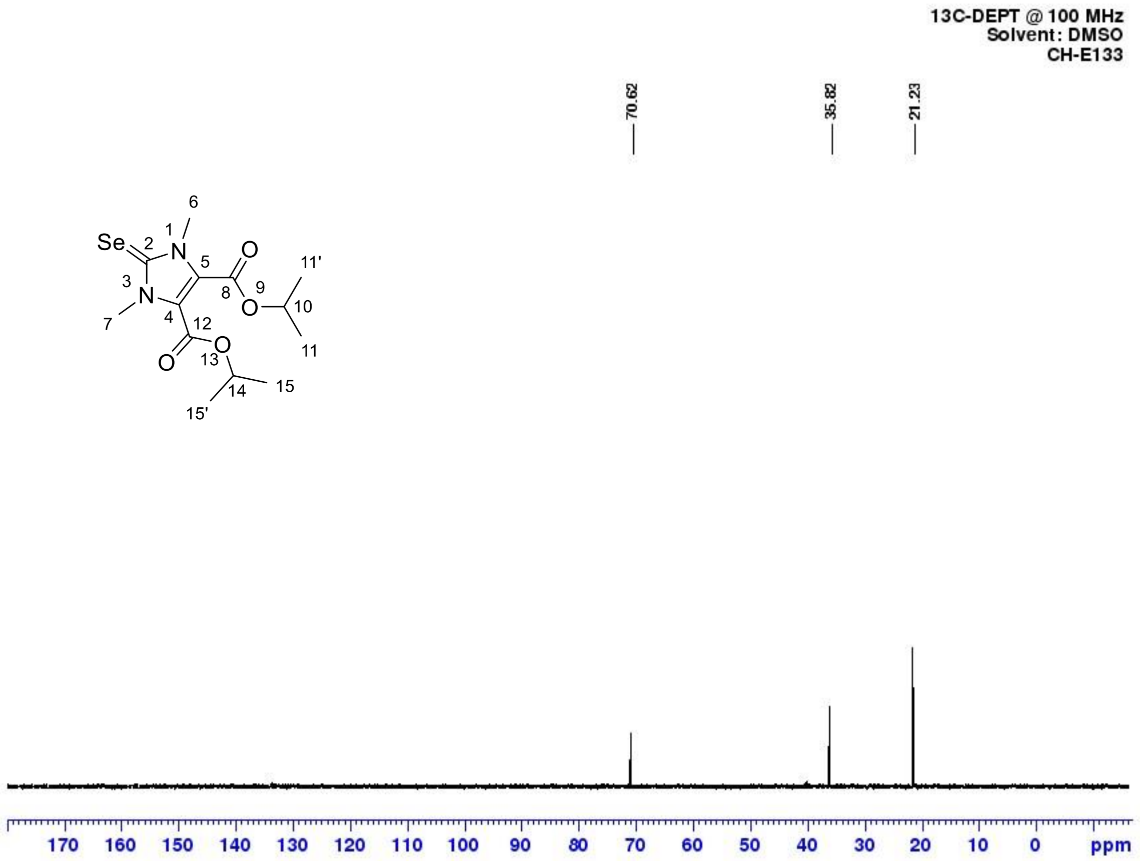

Figure S25: $^{13}C\{^{1}H\}$ DEPT NMR spectrum of **12b** in DMSO-d$_6$.

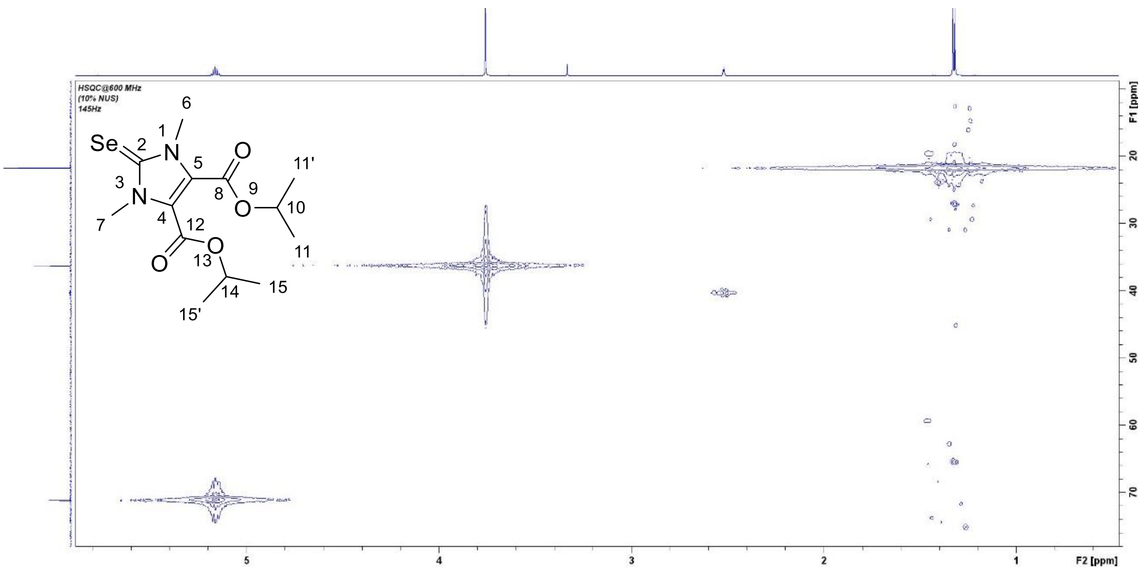

Figure S26: HSQC NMR spectrum of **12b** in DMSO-d$_6$.

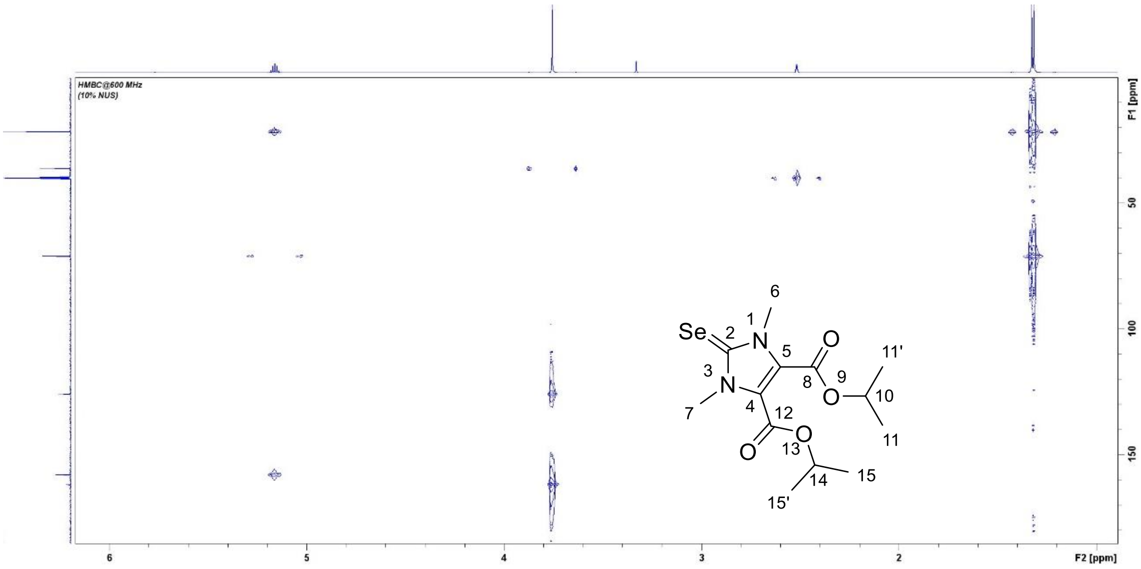

Figure S27: HMBC NMR spectrum of **12b** in DMSO-d$_6$.

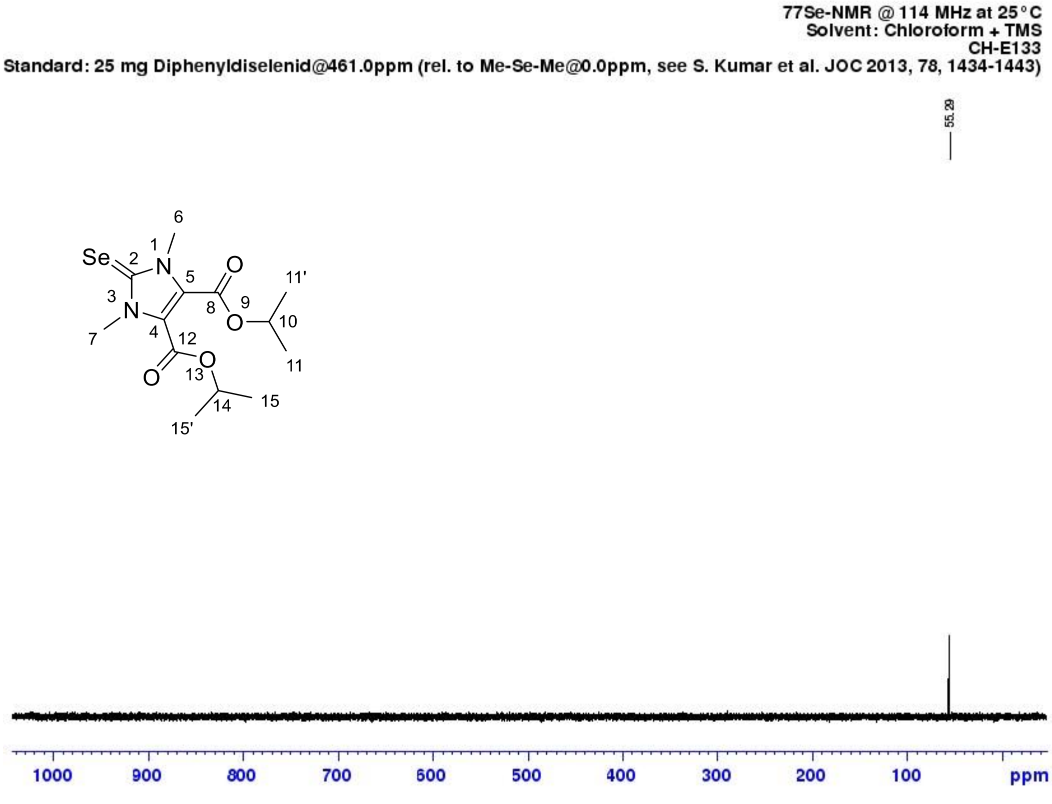

Figure S28: ^{77}Se NMR spectrum of **12b** in $CDCl_3$.

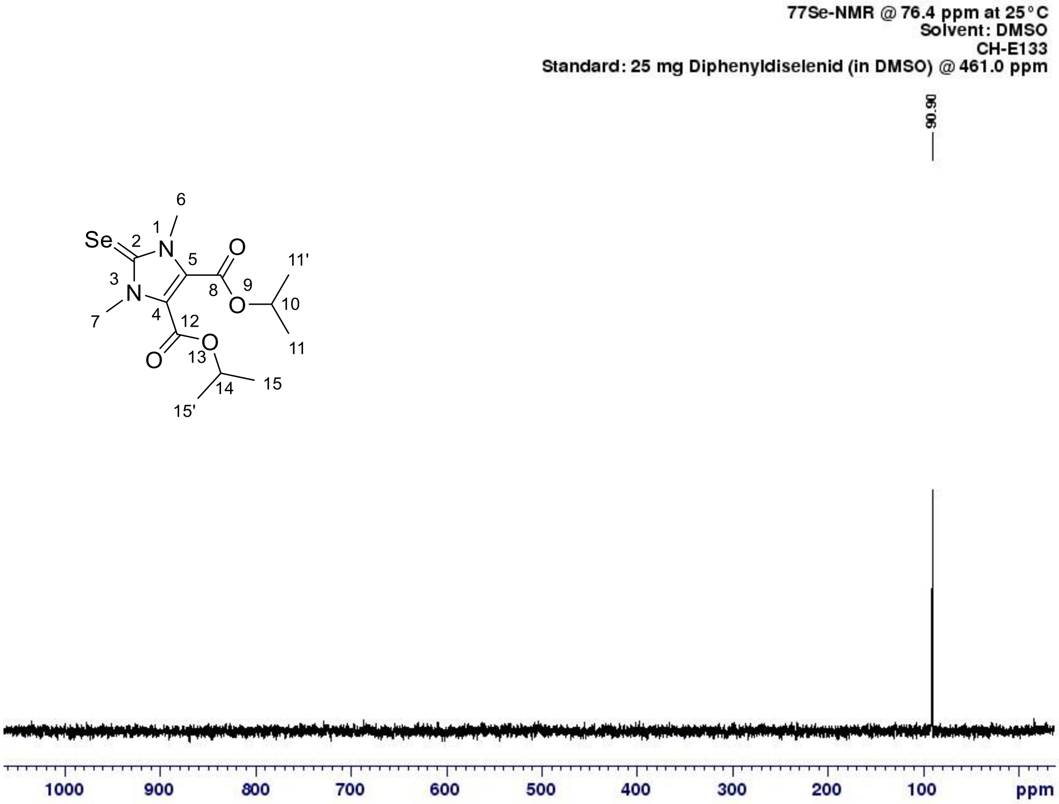

Figure S29: ^{77}Se NMR spectrum of **12b** in DMSO-d$_6$.

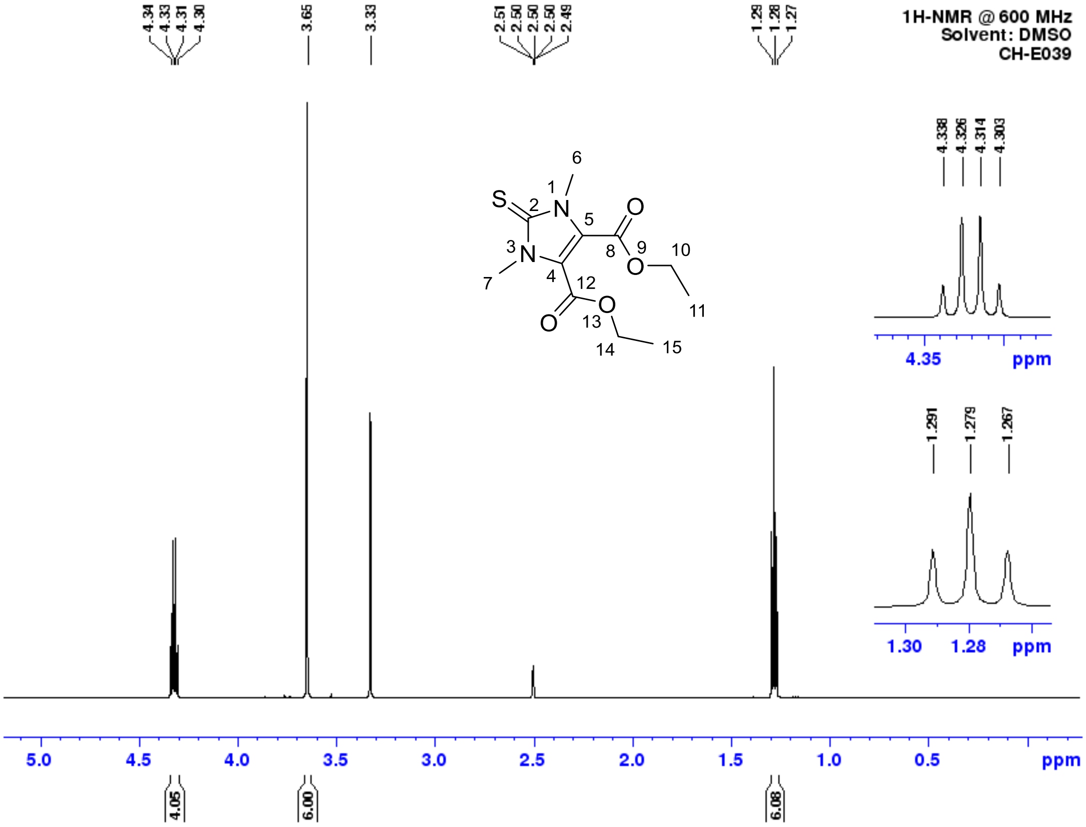

Figure S30: ^{1}H NMR spectrum of **12c** in DMSO-d$_6$.

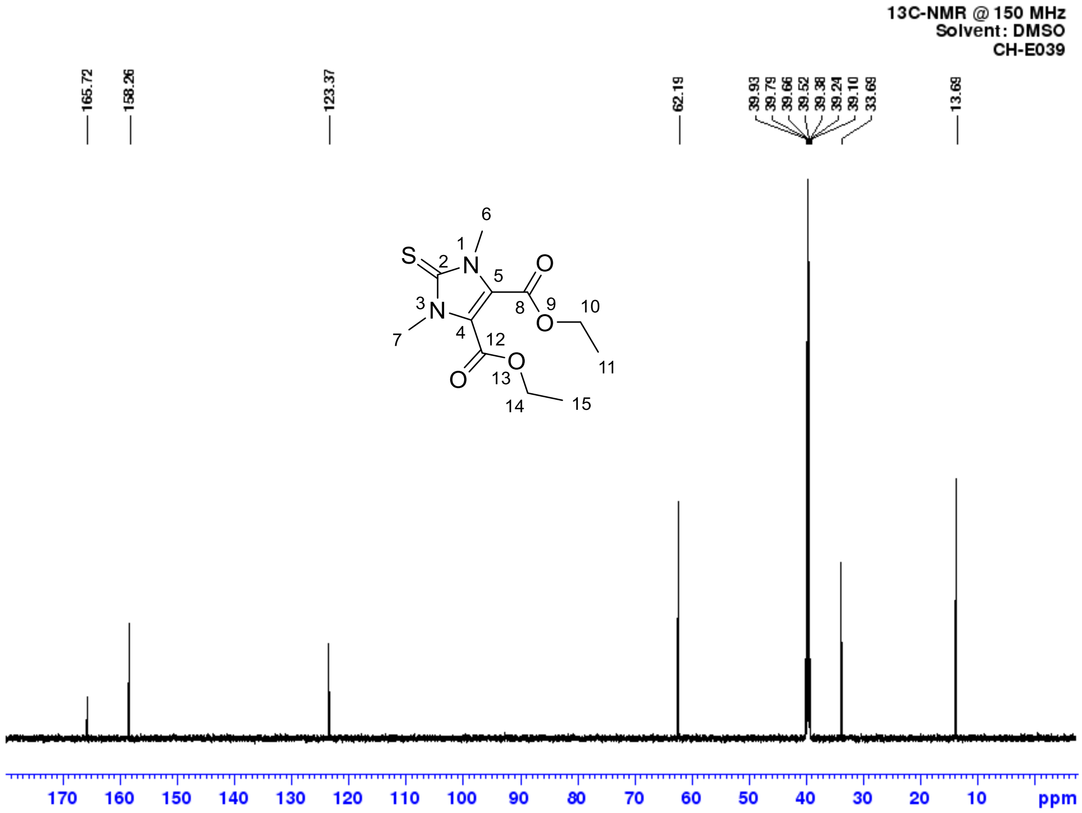

Figure S31: $^{13}C\{^{1}H\}$ NMR spectrum of **12c** in DMSO-d_6.

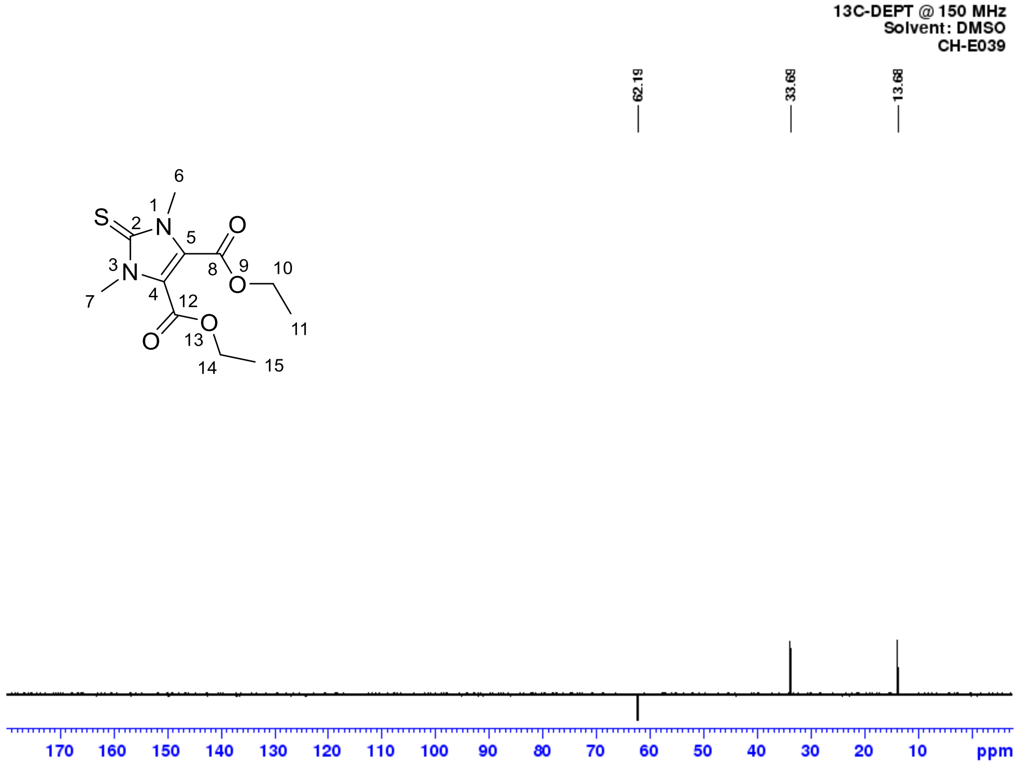

Figure S32: $^{13}C\{^{1}H\}$ DEPT NMR spectrum of **12c** in DMSO-d_6.

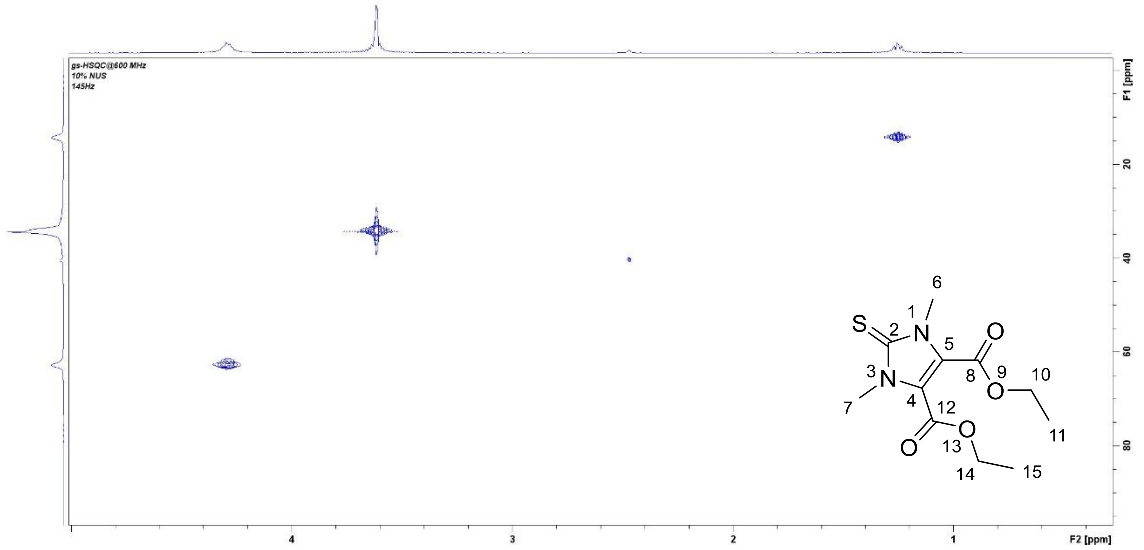

Figure S33: HSQC NMR spectrum of **12c** in DMSO-d_6.

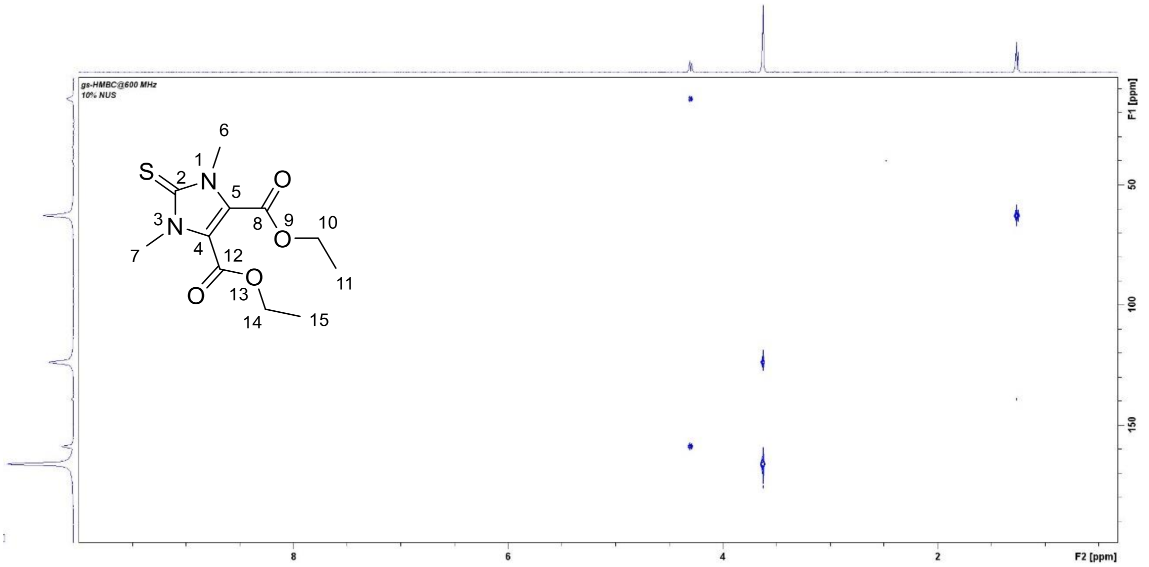

Figure S34: HMBC NMR spectrum of **12c** in DMSO-d_6.

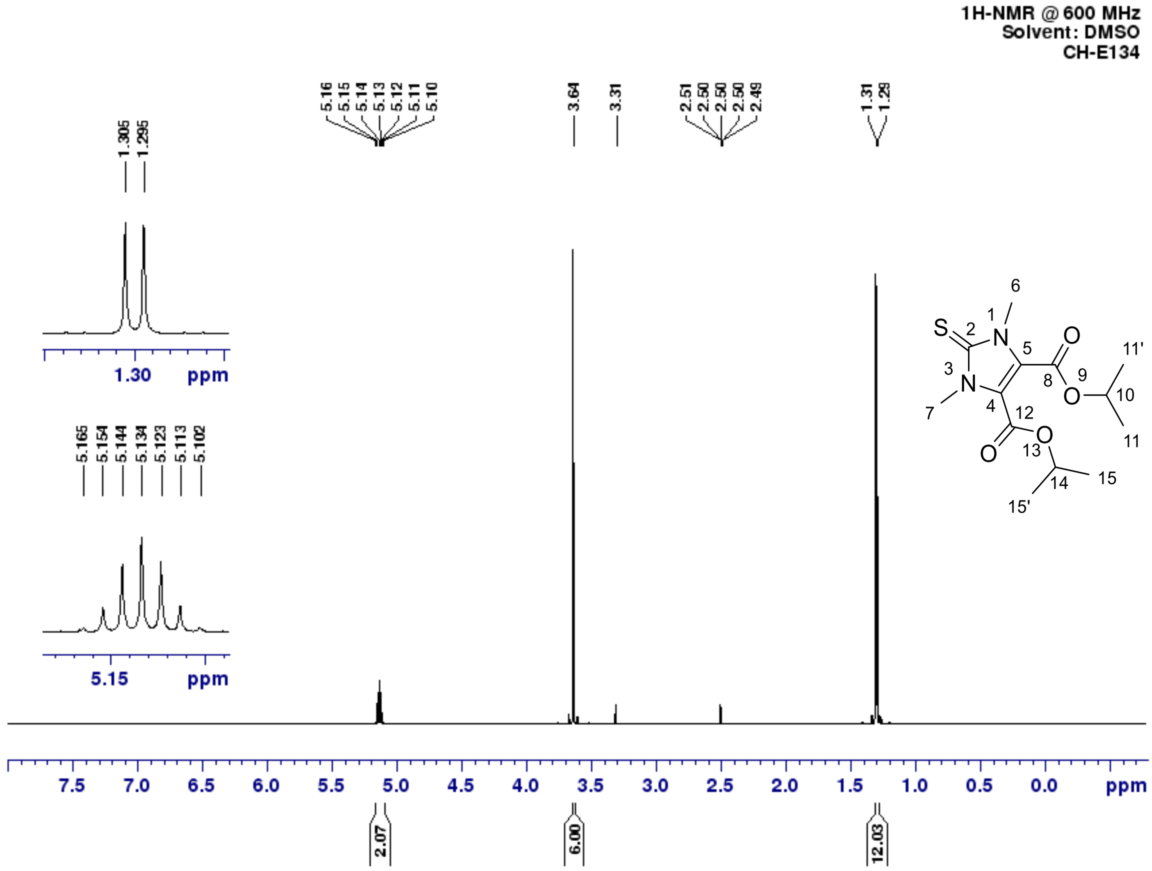

Figure S35: ^{1}H NMR spectrum of **12d** in DMSO-d_6.

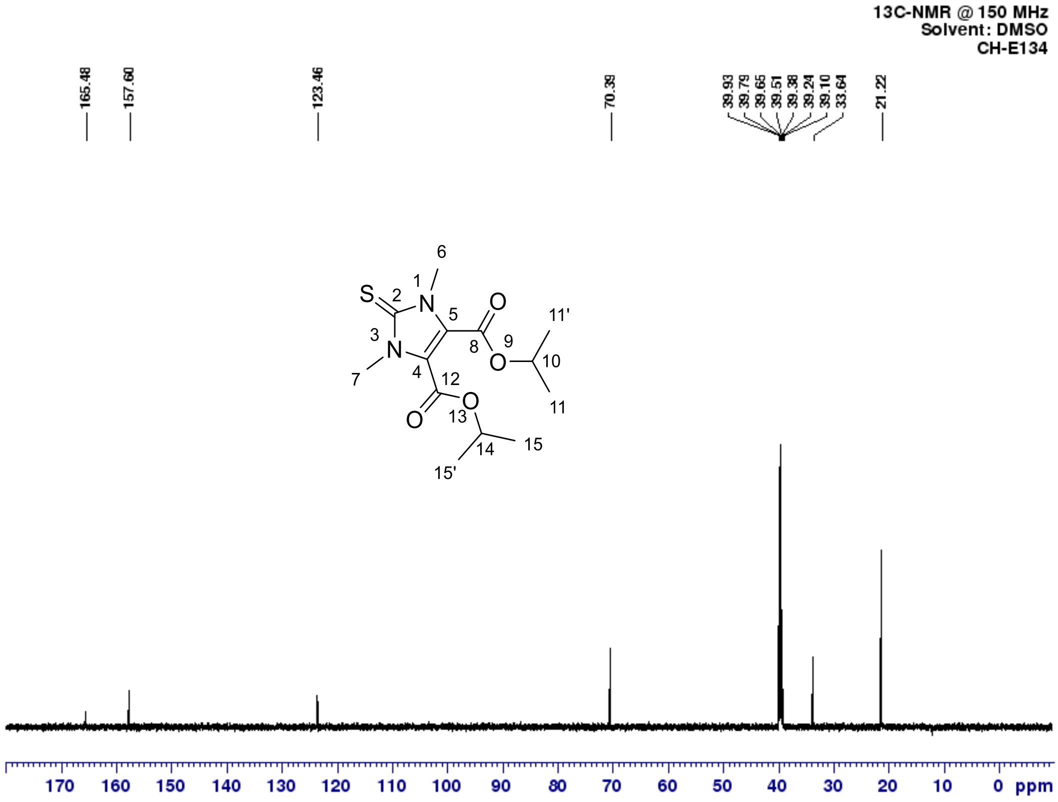

Figure S36: $^{13}C\{^1H\}$ NMR spectrum of **12d** in DMSO-d_6.

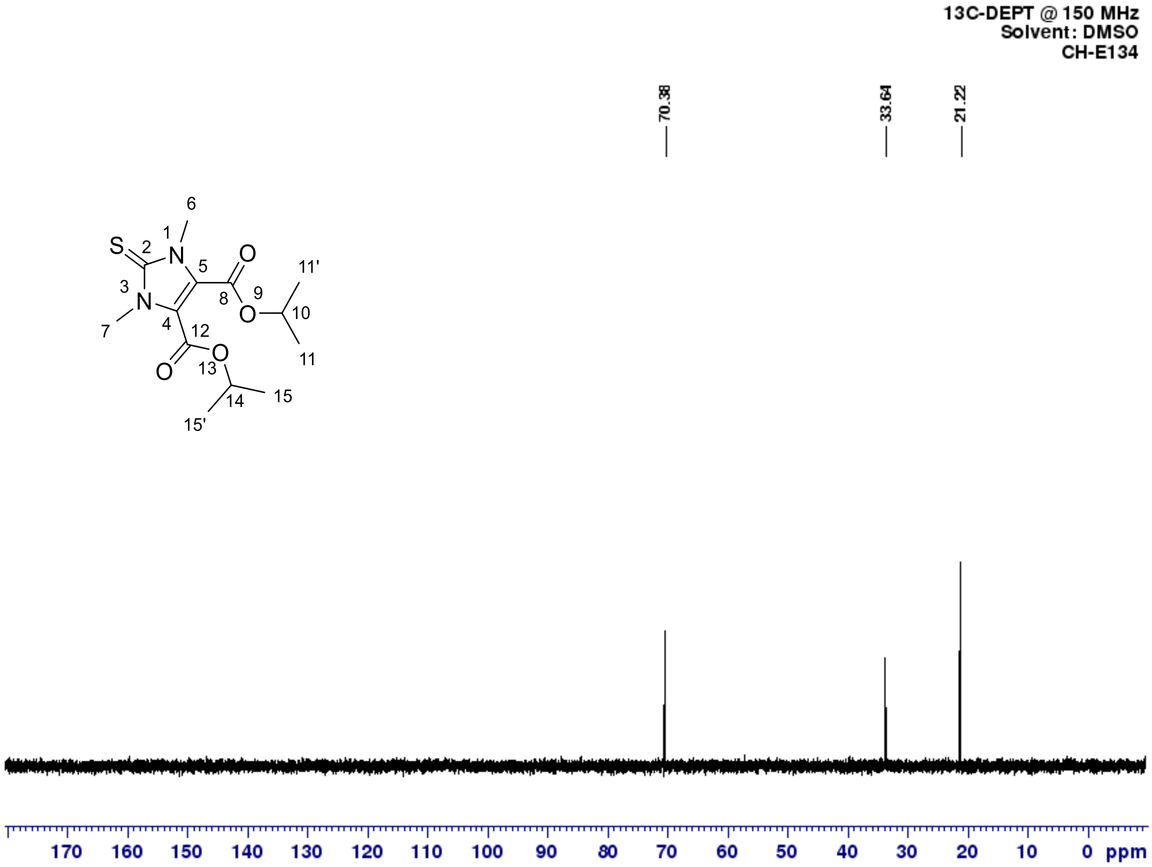

Figure S37: $^{13}C\{^{1}H\}$ DEPT NMR spectrum of **12d** in DMSO-d_6.

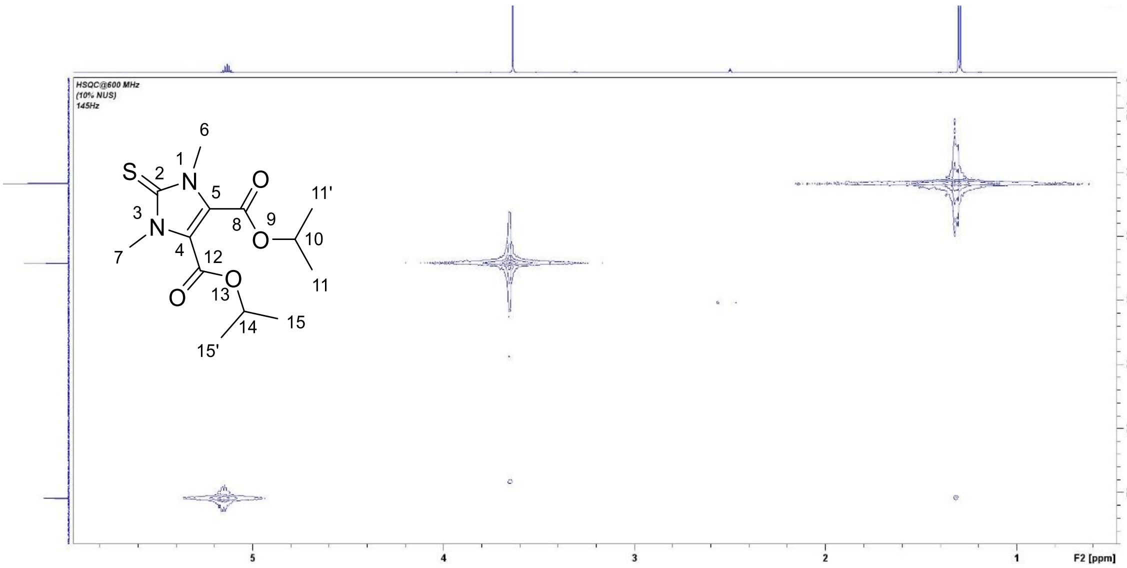

Figure

Figure S38: HSQC NMR spectrum of **12d** in DMSO-d_6.

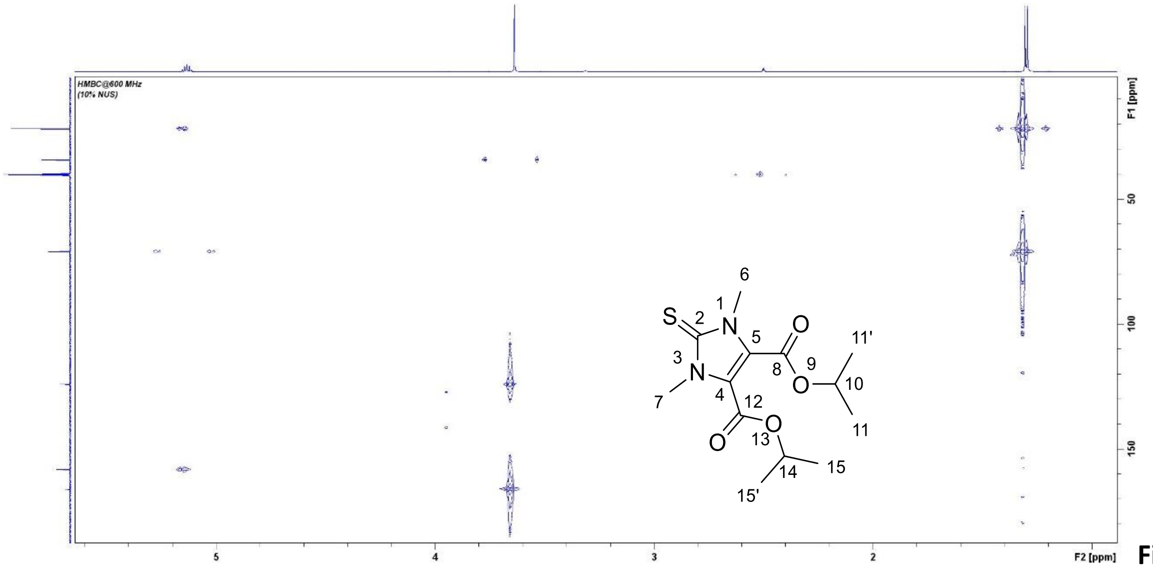

Figure

Figure S39: HMBC NMR spectrum of **12d** in DMSO-d$_6$.

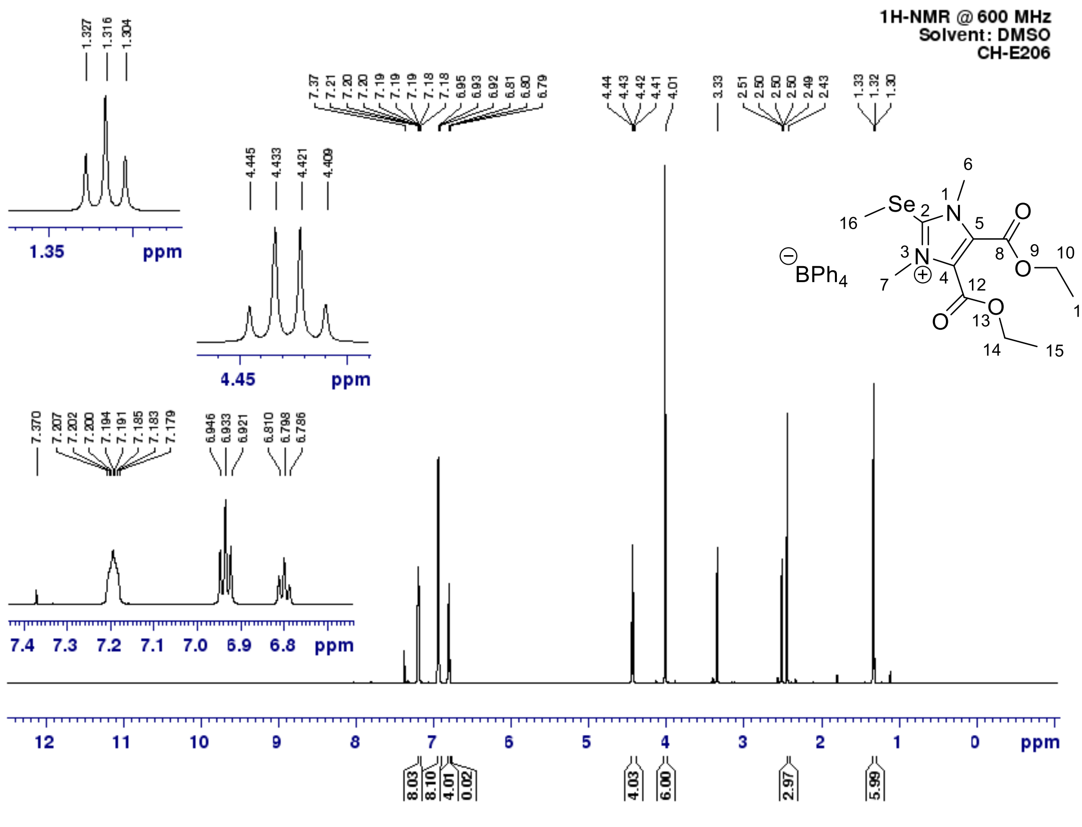

Figure S40: ^{1}H NMR spectrum of **13a** in DMSO-d$_6$.

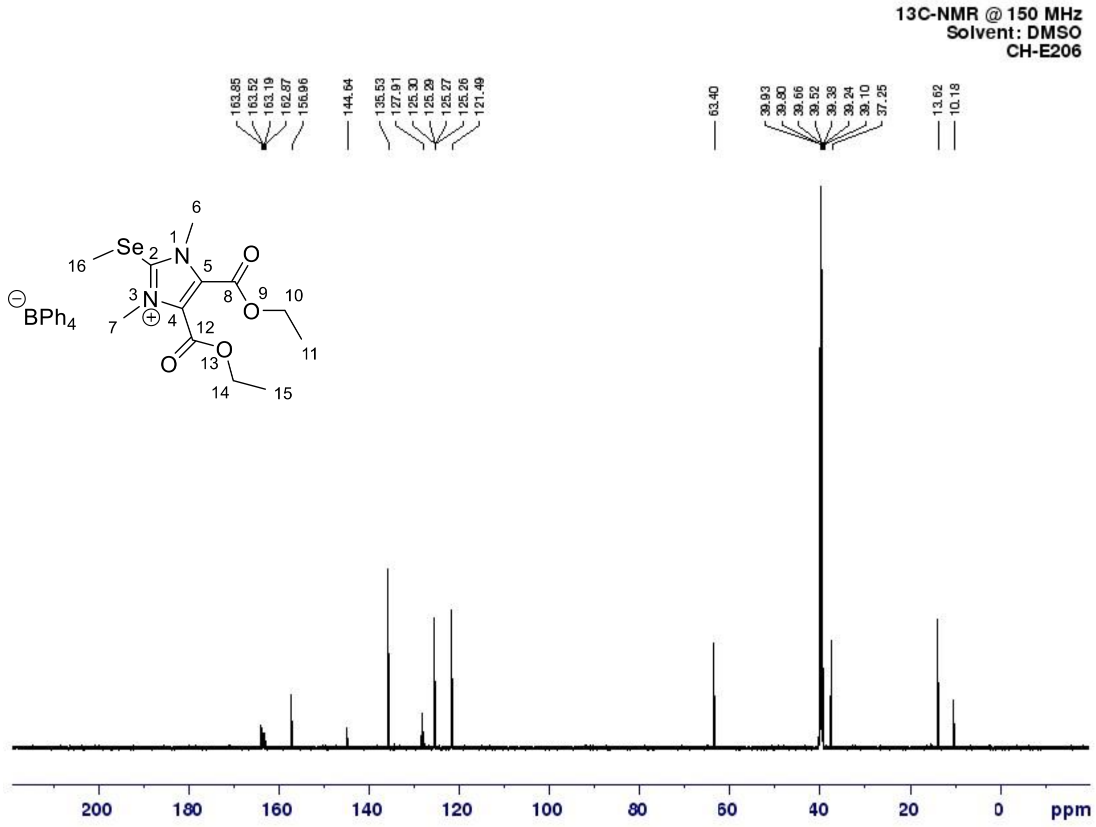

Figure S41: $^{13}C\{^{1}H\}$ NMR spectrum of **13a** in DMSO-d$_6$.

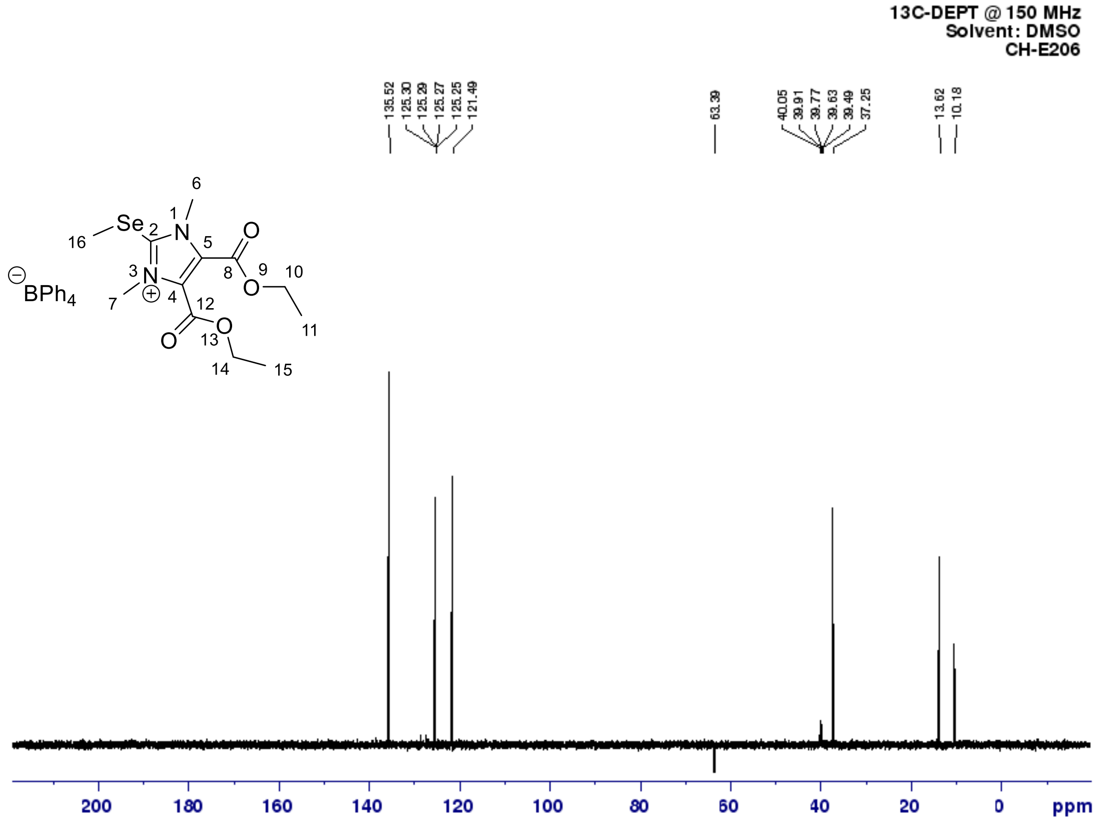

Figure S42: $^{13}C\{^{1}H\}$ DEPT NMR spectrum of **13a** in DMSO-d$_6$.

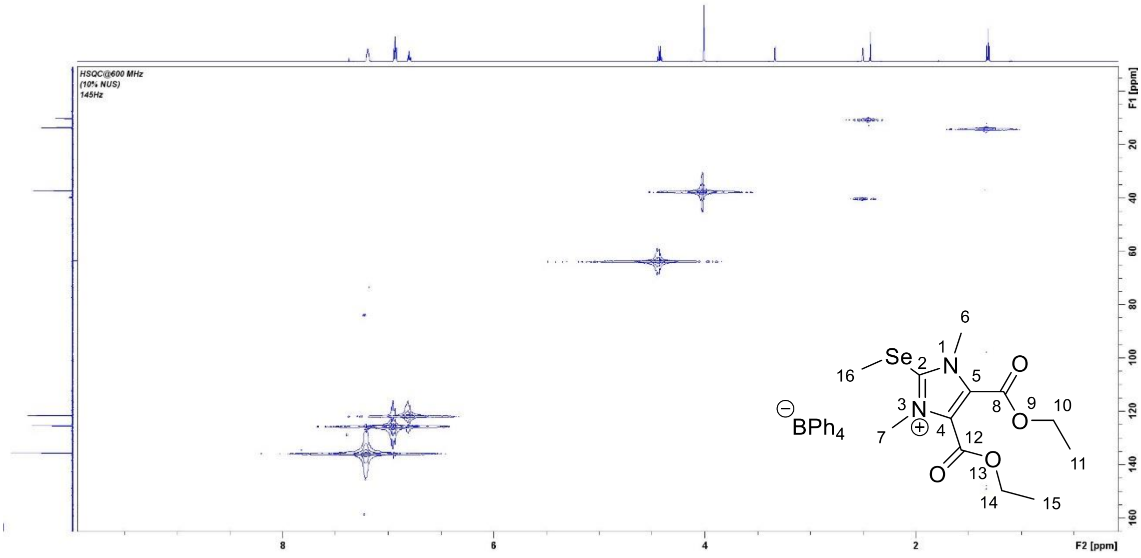

Figure S43: HSQC NMR spectrum of **13a** in DMSO-d$_6$.

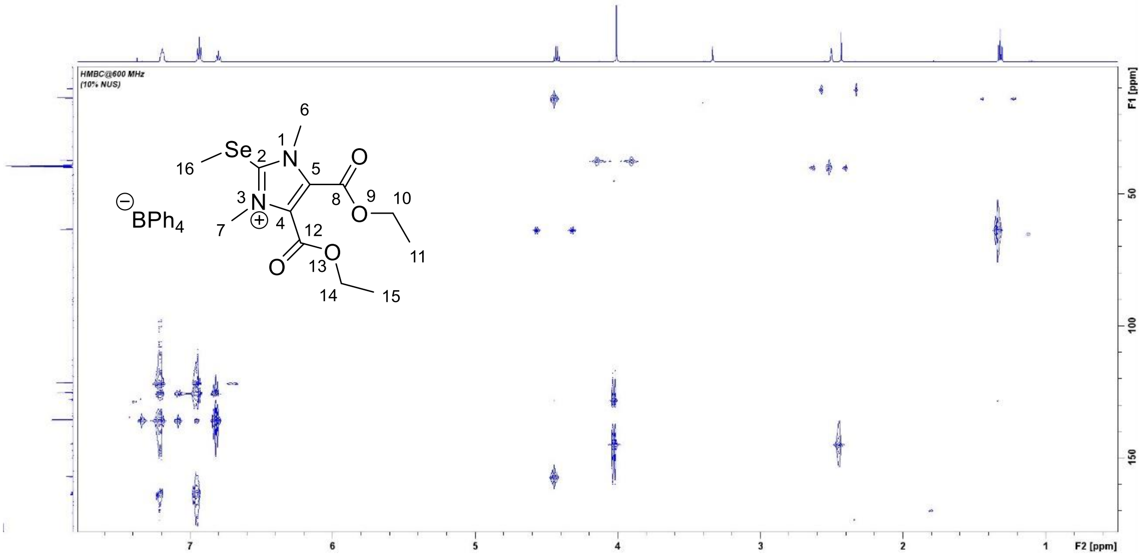

Figure S44: HMBC NMR spectrum of **13a** in DMSO-d$_6$.

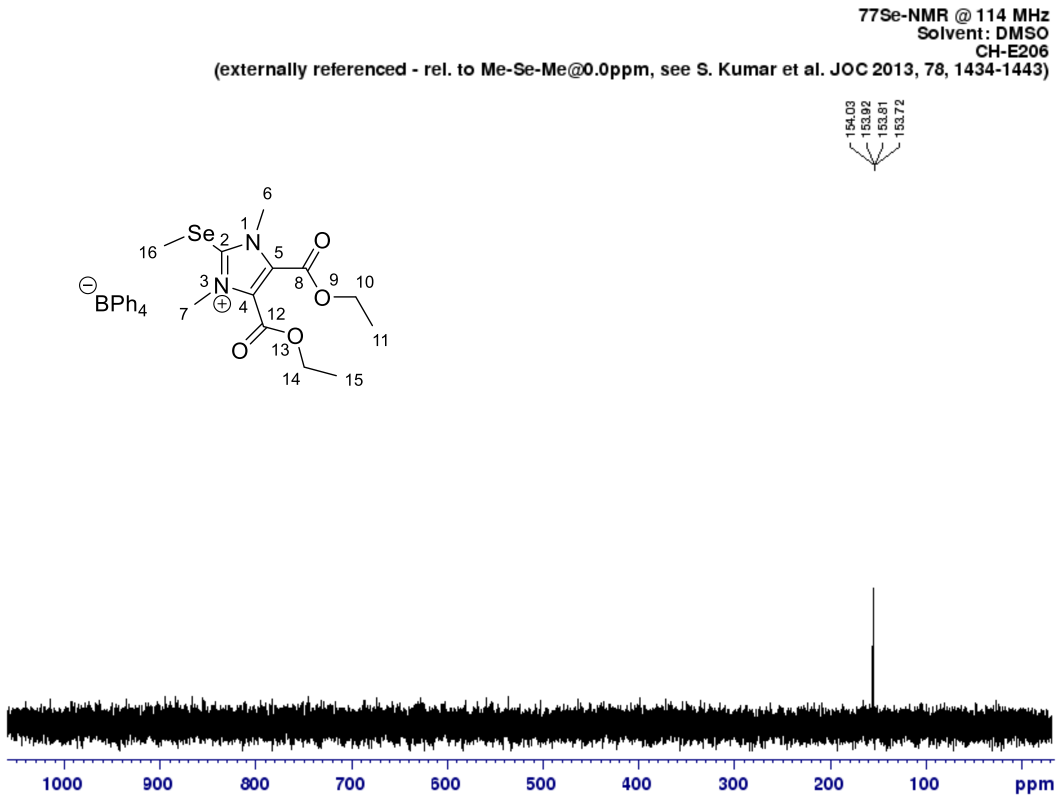

Figure S45: ^{77}Se NMR spectrum of **13a** in DMSO-d$_6$.

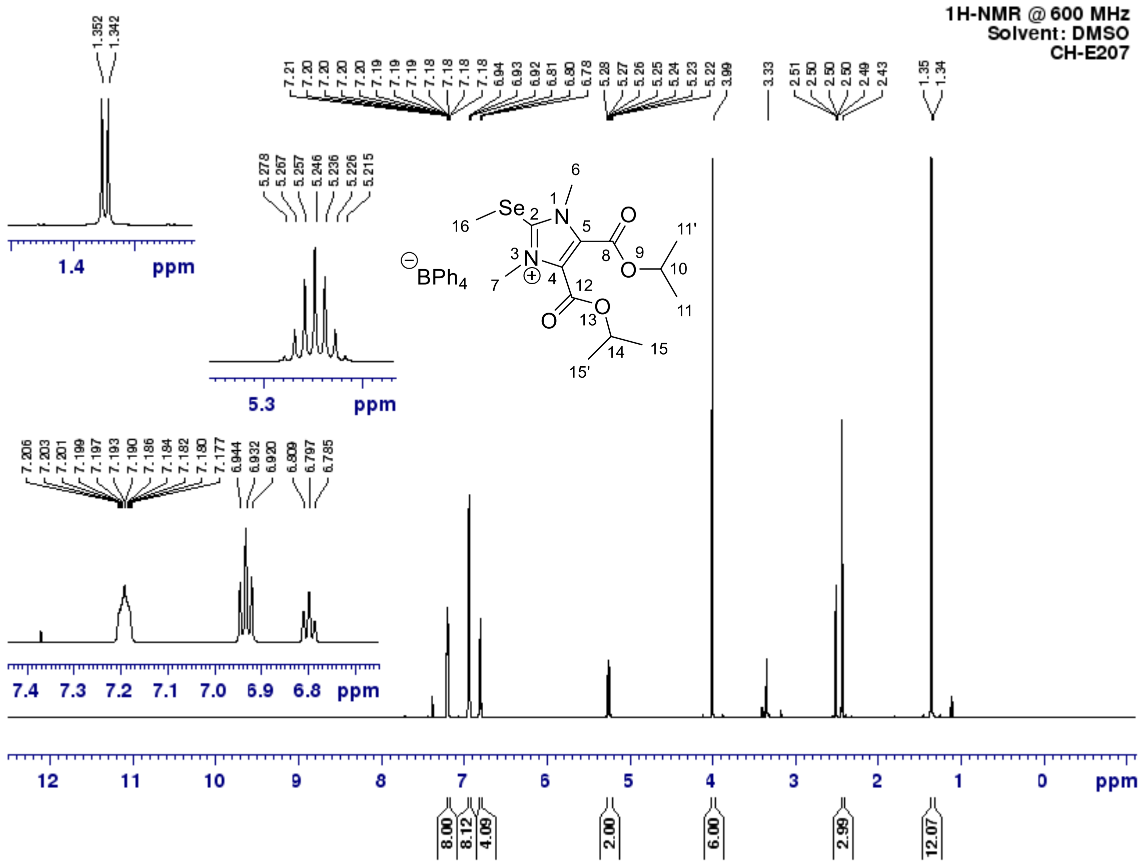

Figure S46: ^{1}H NMR spectrum of **13b** in DMSO-d$_6$.

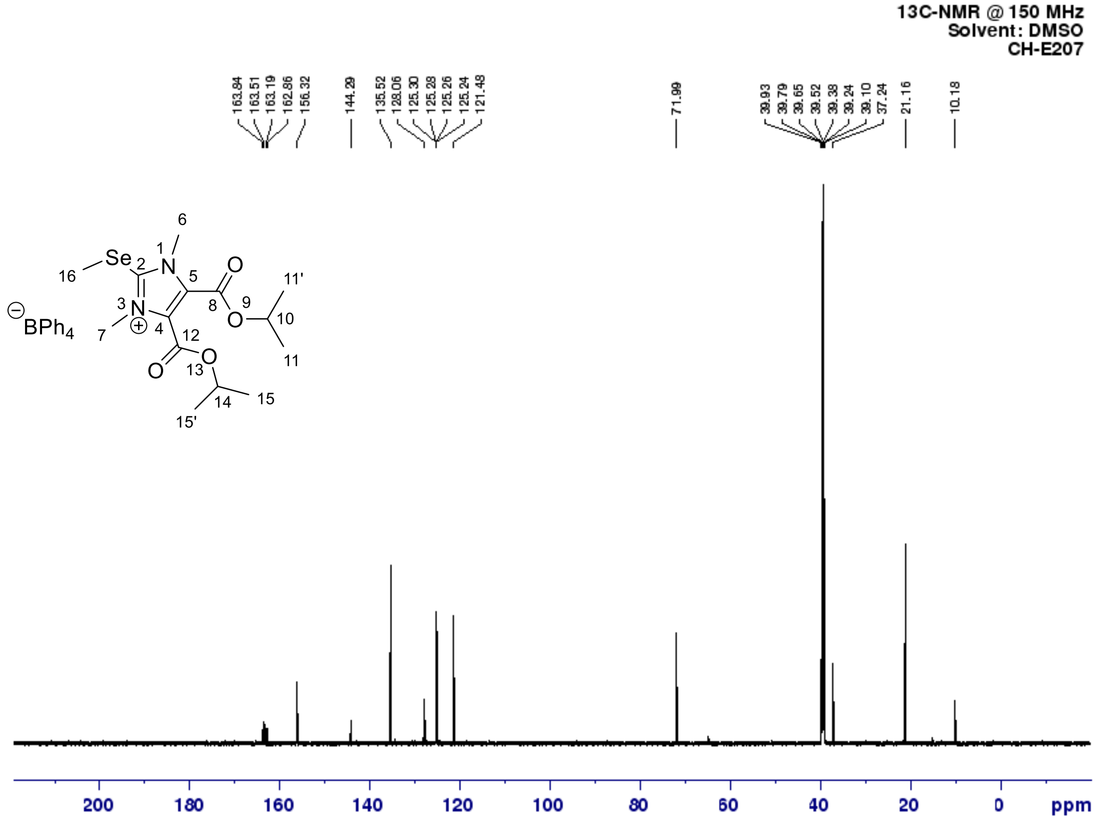

Figure S47: $^{13}C\{^{1}H\}$ NMR spectrum of **13b** in DMSO-d$_6$.

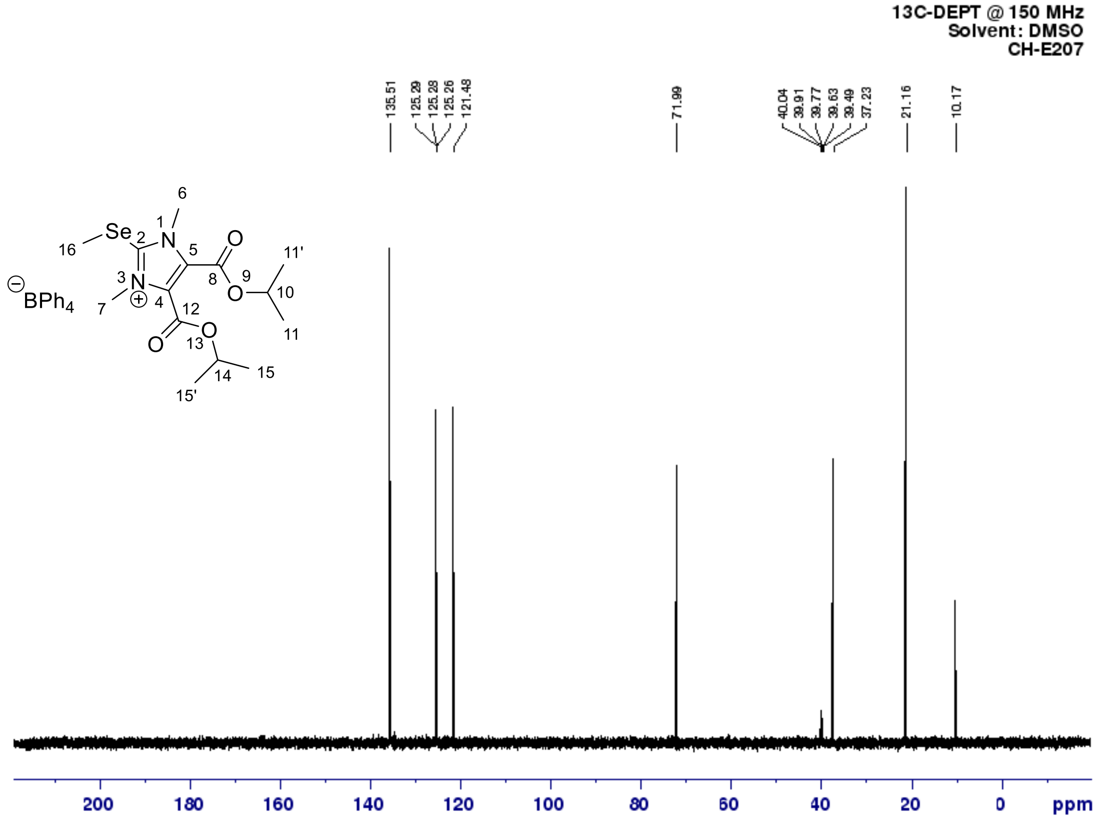

Figure S48: $^{13}C\{^{1}H\}$ DEPT NMR spectrum of **13b** in DMSO-d$_6$.

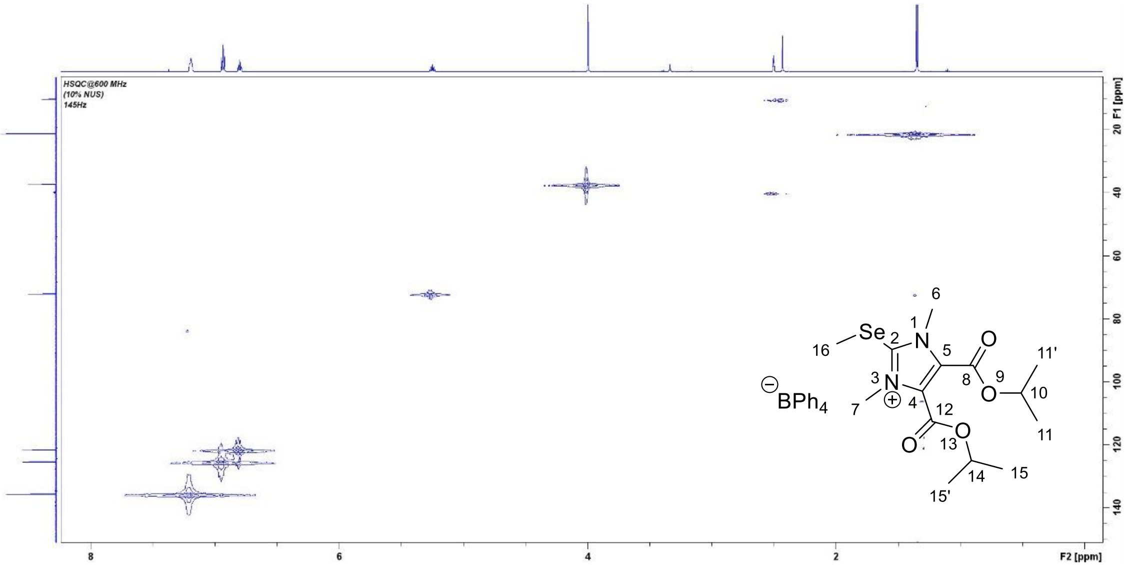

Figure S49: HSQC NMR spectrum of **13b** in DMSO-d$_6$.

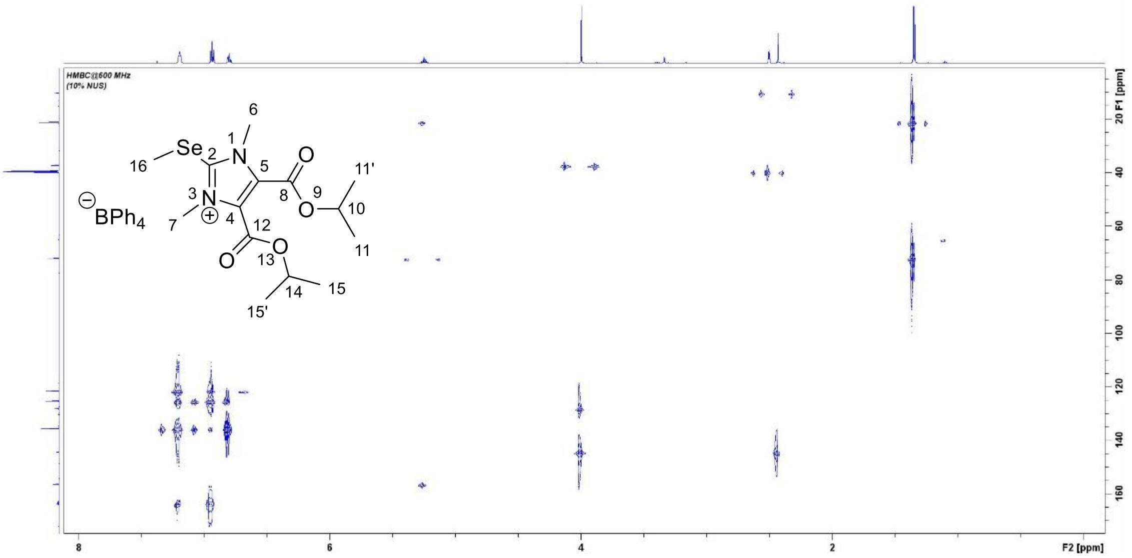

Figure S50: HMBC NMR spectrum of **13b** in DMSO-d$_6$.

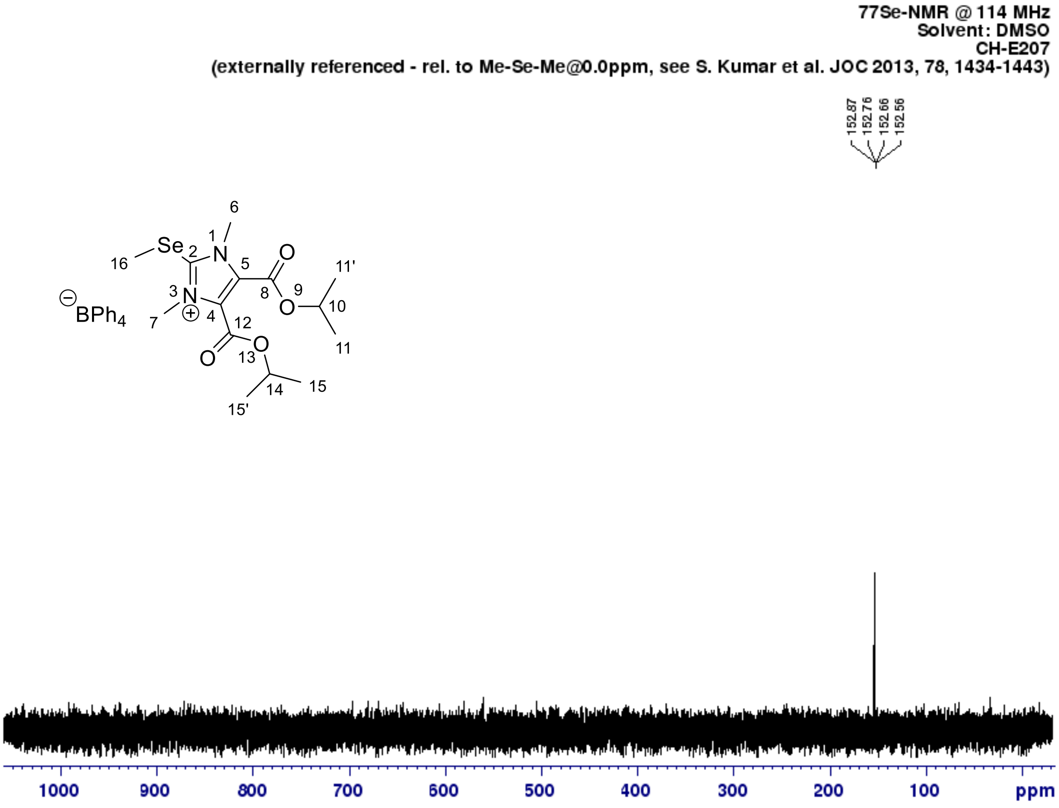

Figure S51: ^{77}Se NMR spectrum of **13b** in DMSO-d$_6$.

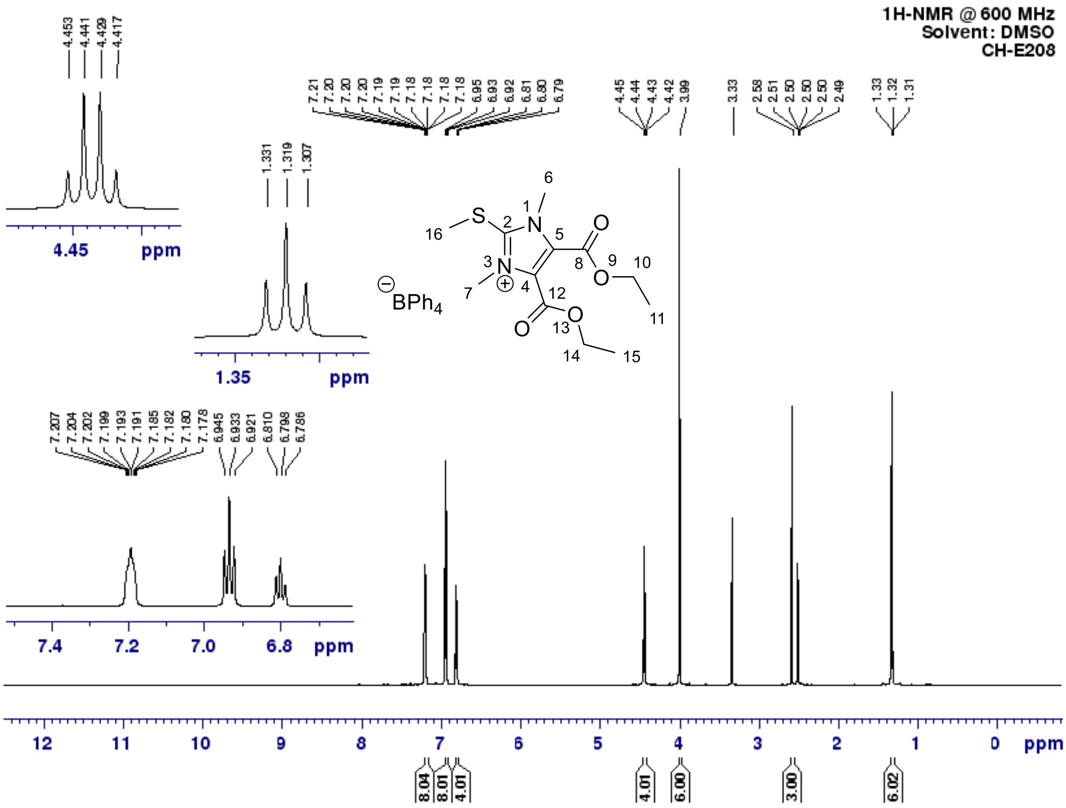

Figure S52: ^{1}H NMR spectrum of **13c** in DMSO-d$_6$.

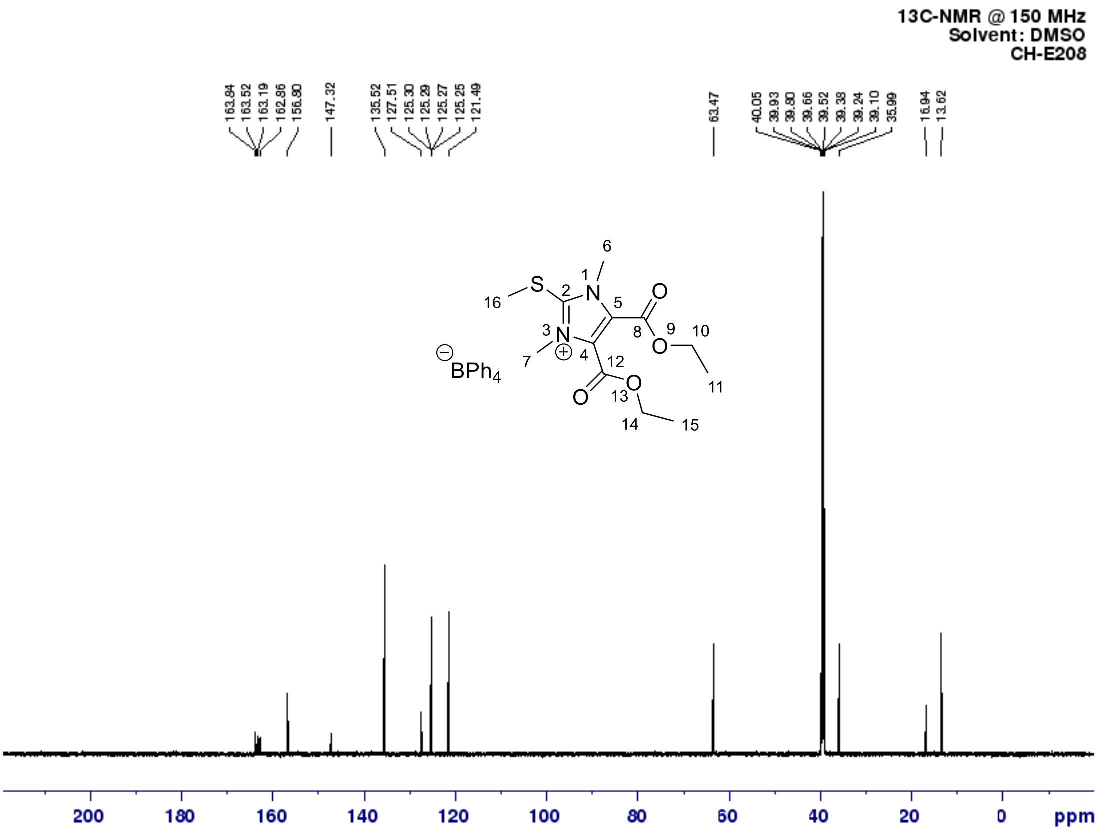

Figure S53: $^{13}C\{^{1}H\}$ NMR spectrum of **13c** in DMSO-d$_6$.

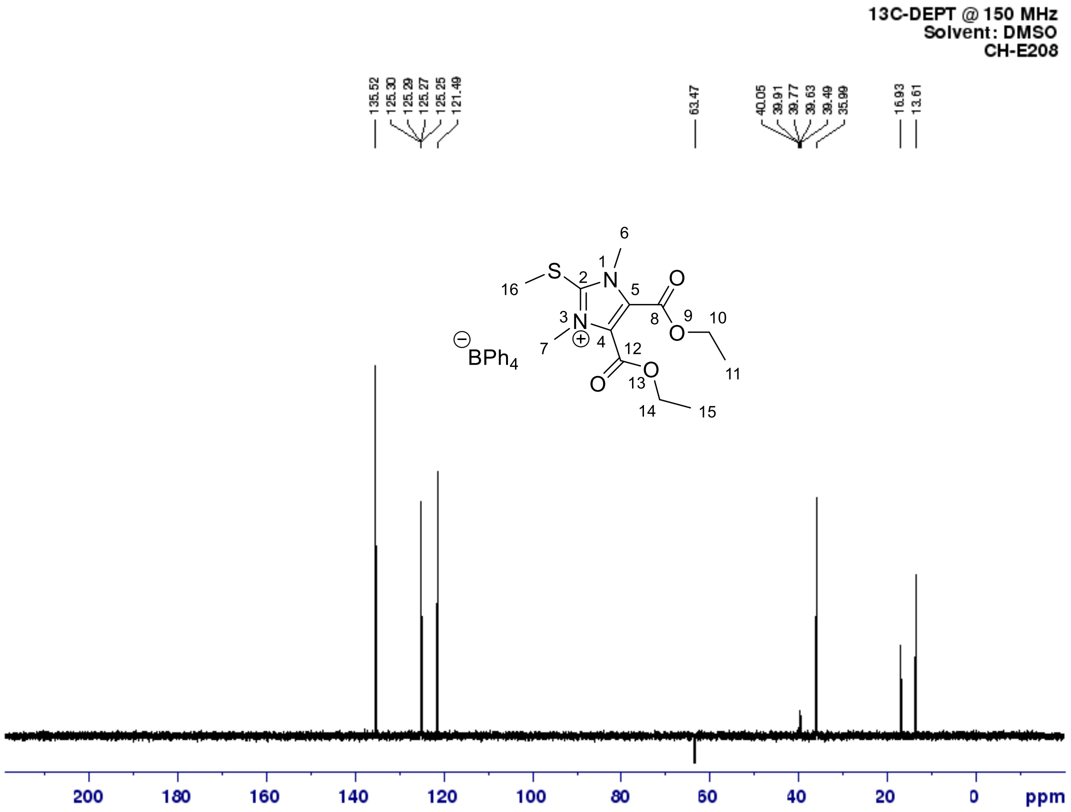

Figure S54: $^{13}C\{^{1}H\}$ DEPT NMR spectrum of **13c** in DMSO-d$_6$.

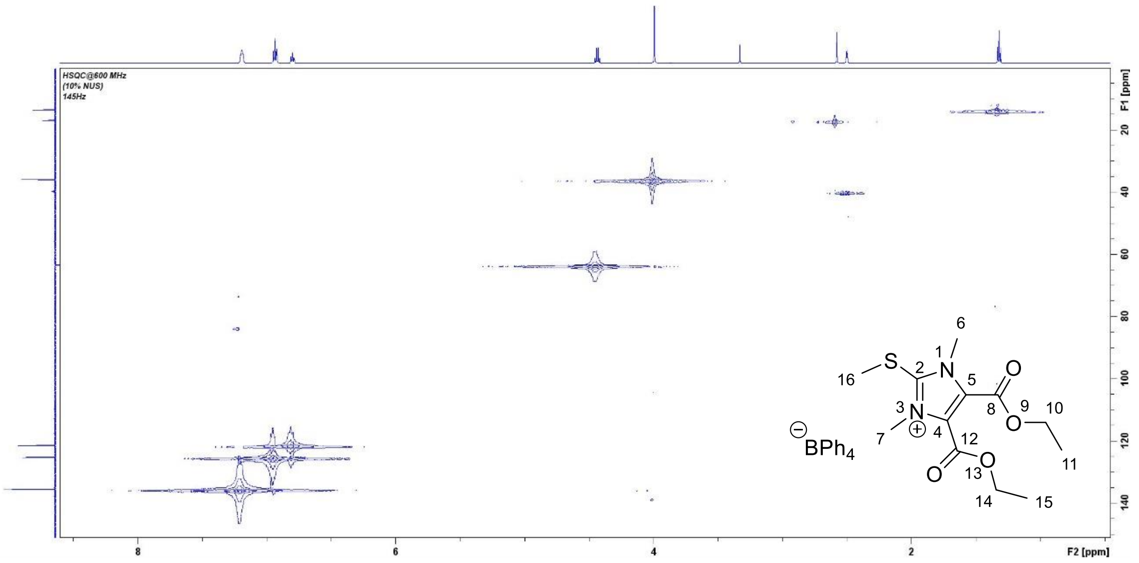

Figure S55: HSQC NMR spectrum of **13c** in DMSO-d$_6$.

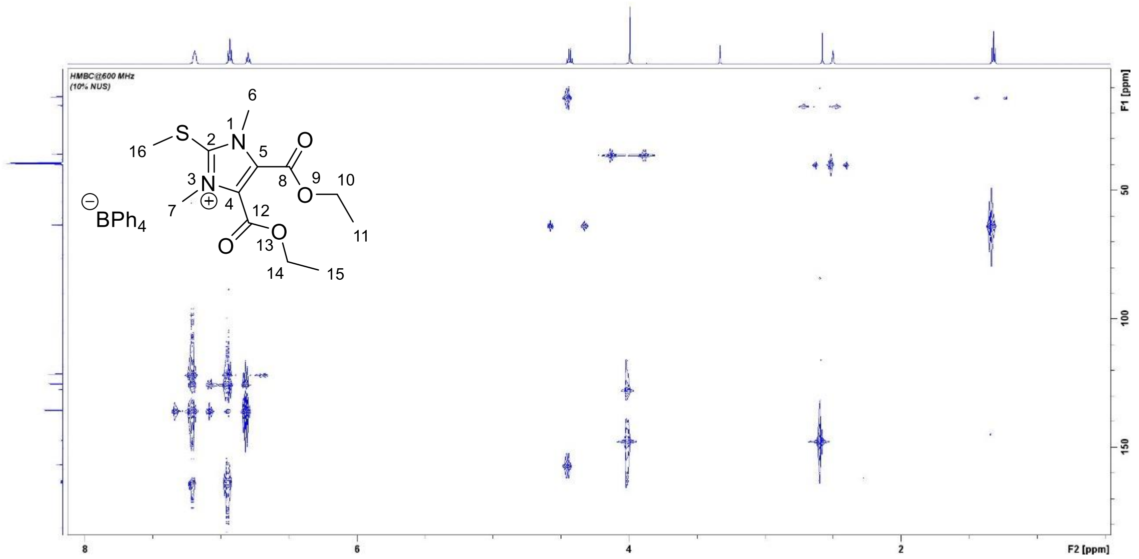

Figure S56: HMBC NMR spectrum of **13c** in DMSO-d$_6$.

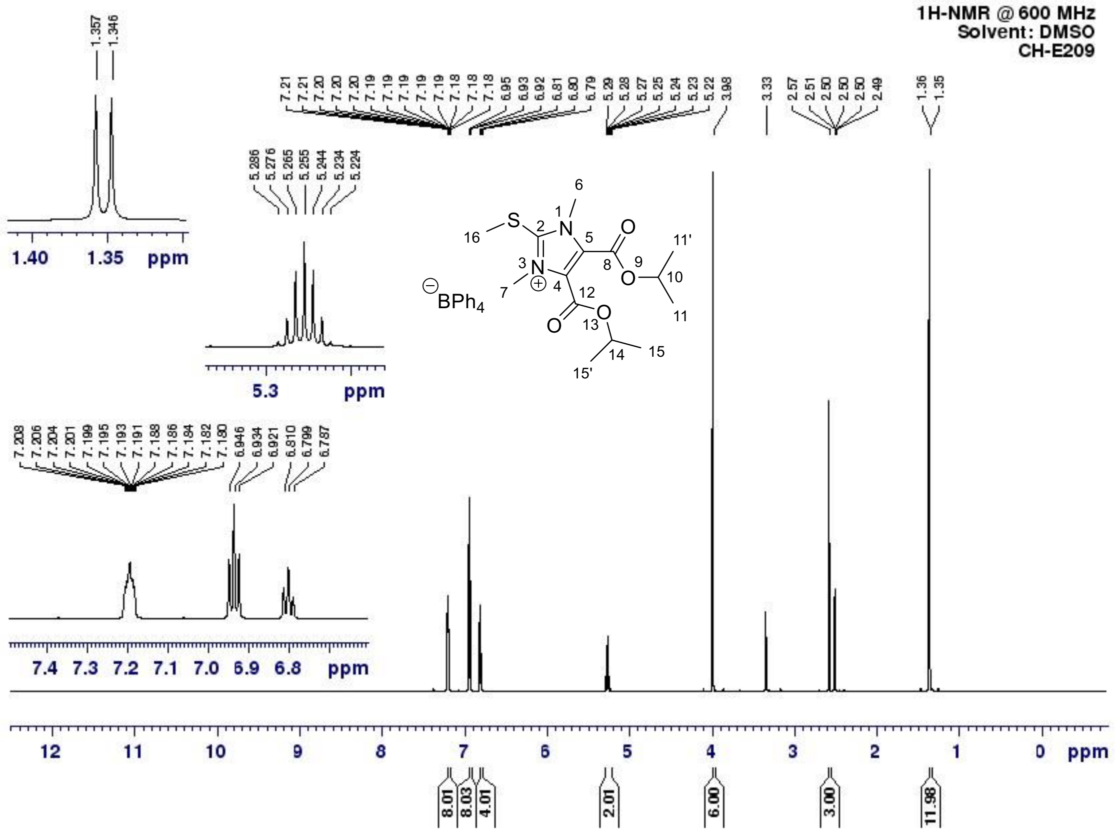

Figure S57: [1] H NMR spectrum of **13d** in DMSO-d$_6$.

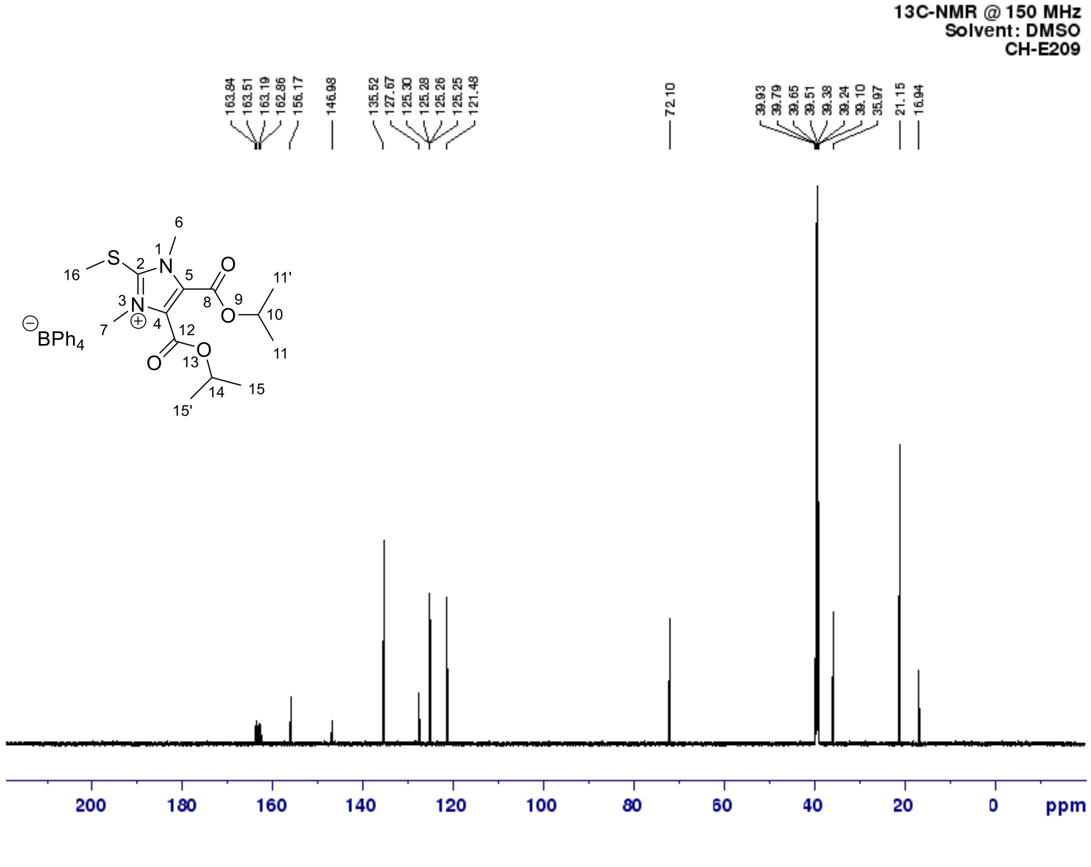

Figure S58: $^{13}C\{^1H\}$ NMR spectrum of **13d** in DMSO-d$_6$.

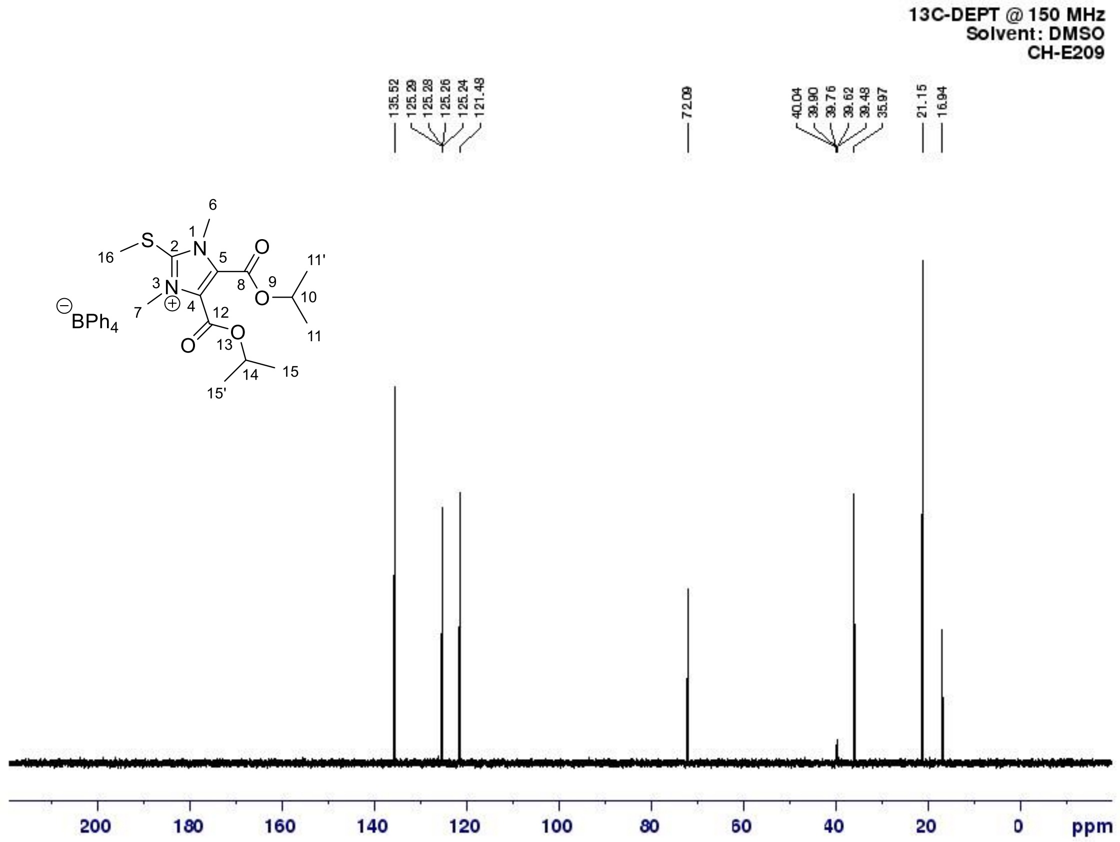

Figure S59: $^{13}C\{^{1}H\}$ DEPT NMR spectrum of **13d** in DMSO-d$_6$.

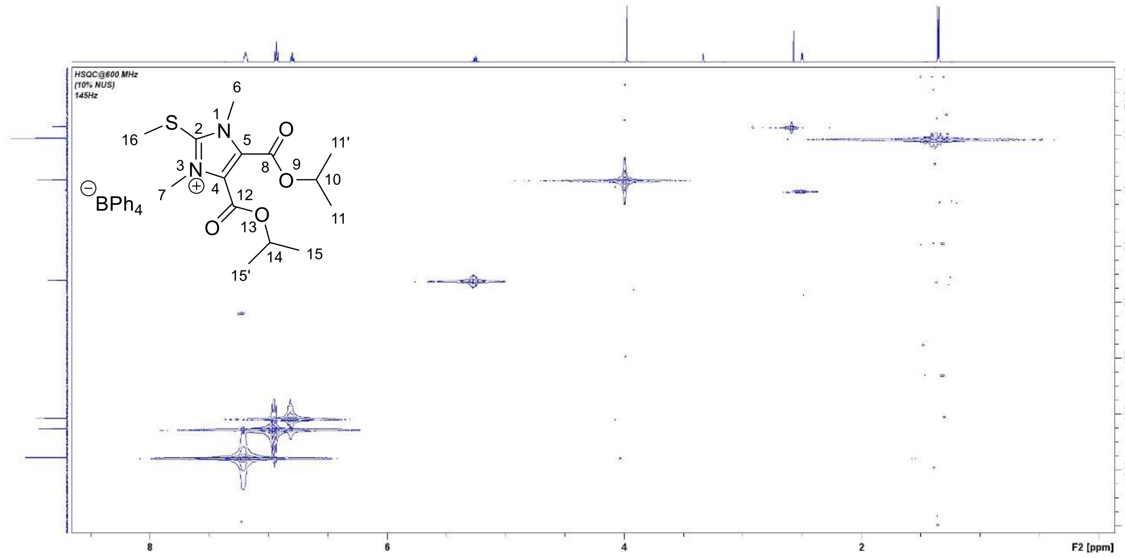

Figure S60: HSQC NMR spectrum of **13d** in DMSO-d$_6$.

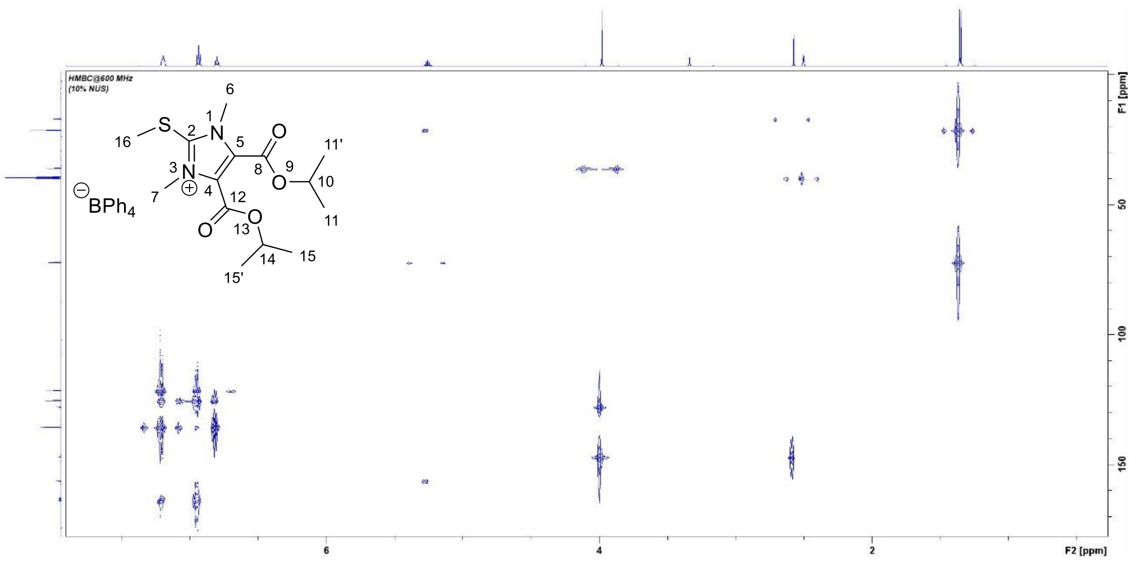

Figure S61: HMBC NMR spectrum of **13d** in DMSO-d$_6$.

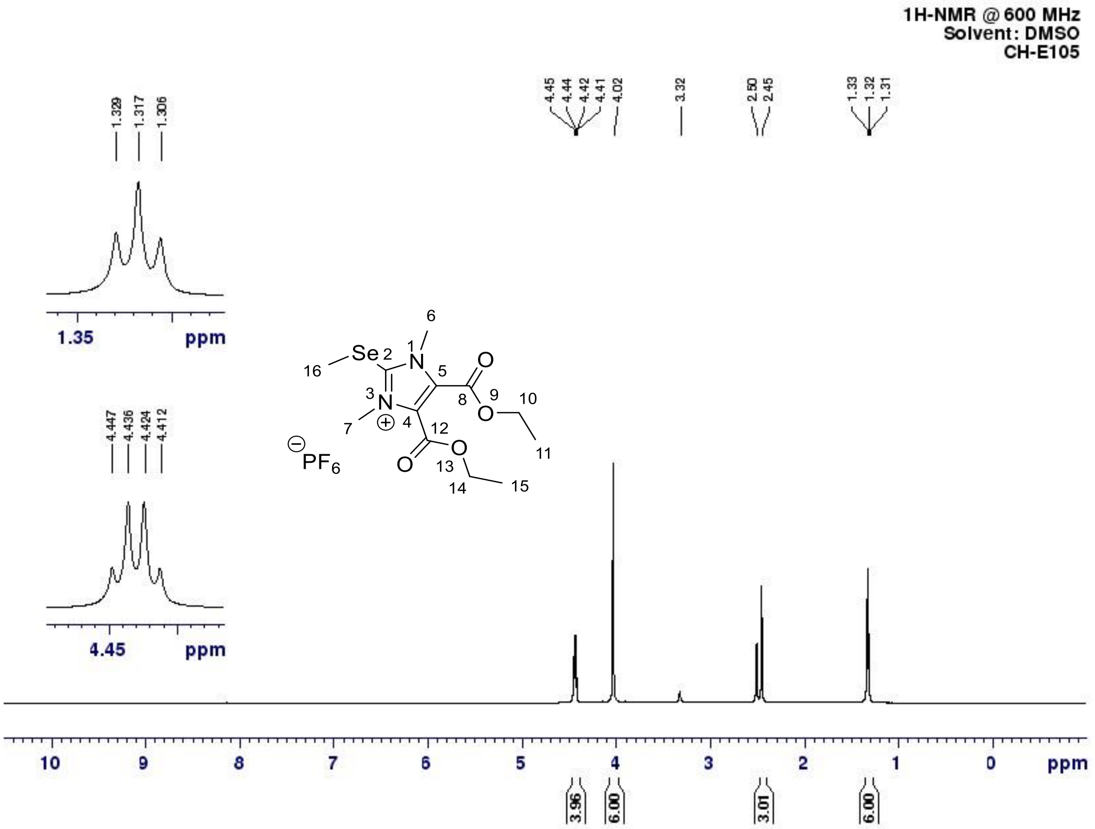

Figure S62: ^{1}H NMR spectrum of **13e** in DMSO-d$_6$.

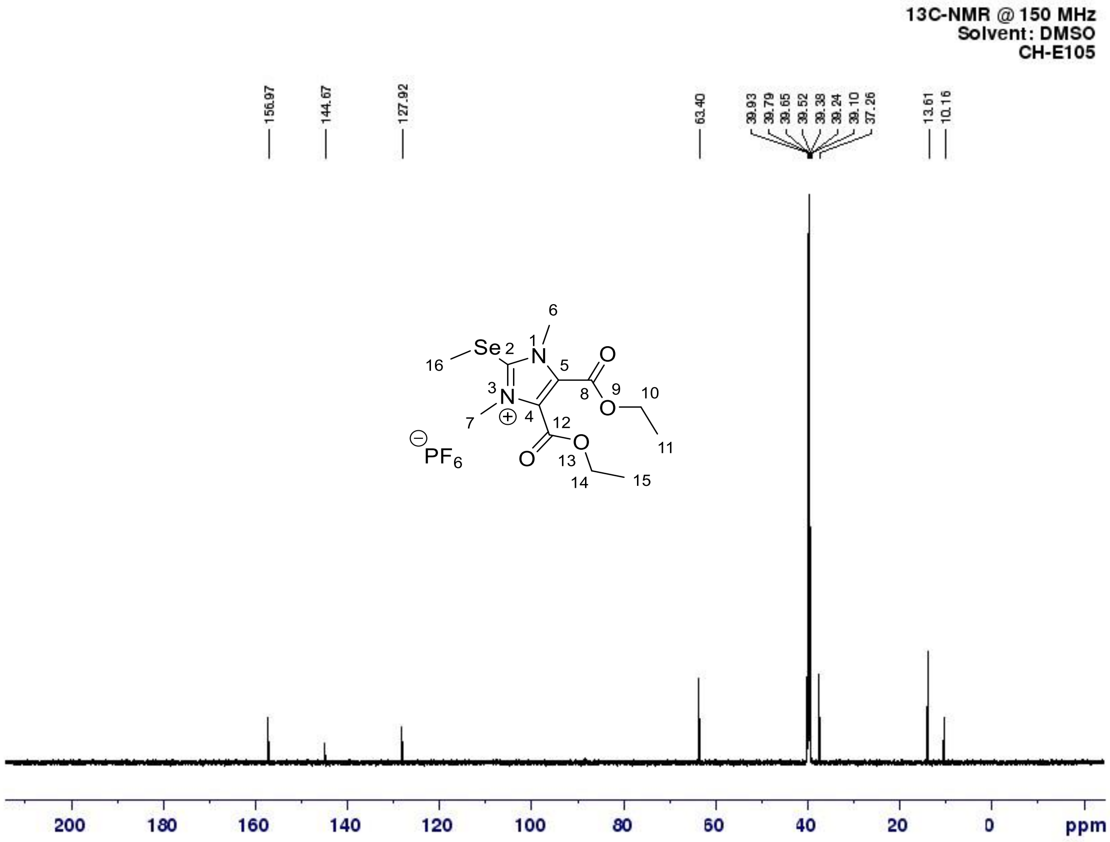

Figure S63: $^{13}C\{^1H\}$ NMR spectrum of **13e** in DMSO-d$_6$.

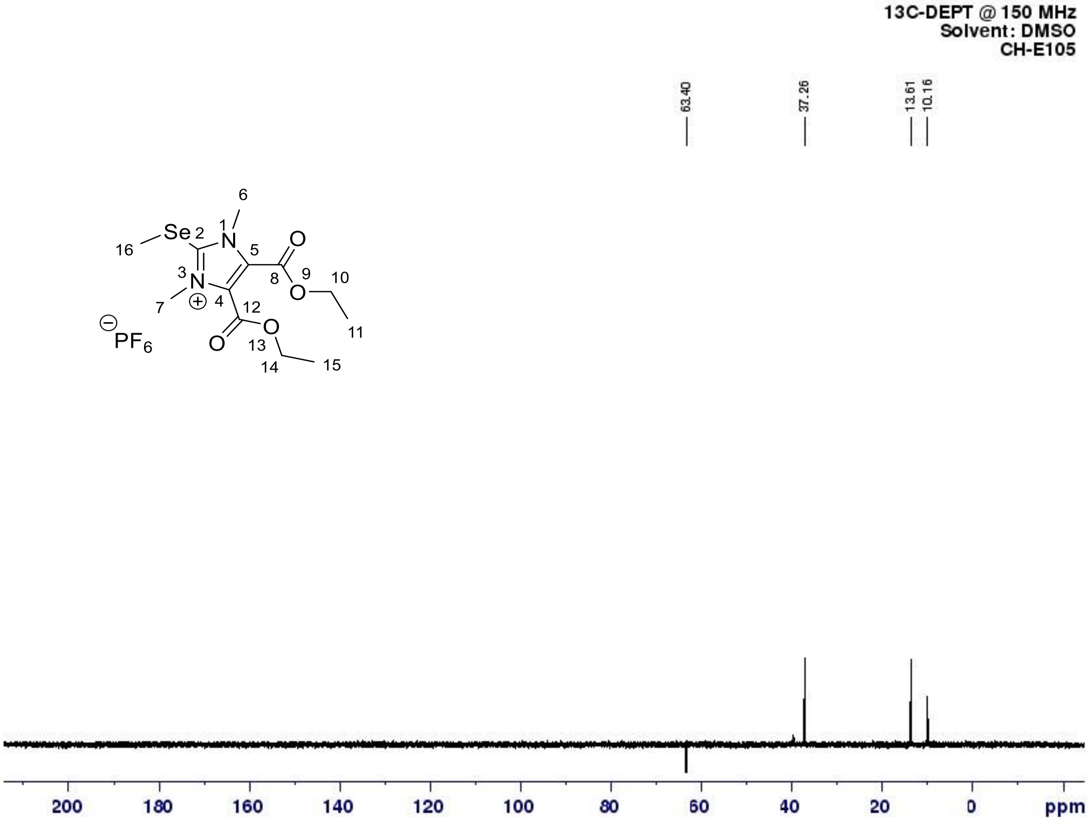

Figure S64: $^{13}C\{^1H\}$ DEPT NMR spectrum of **13e** in DMSO-d$_6$.

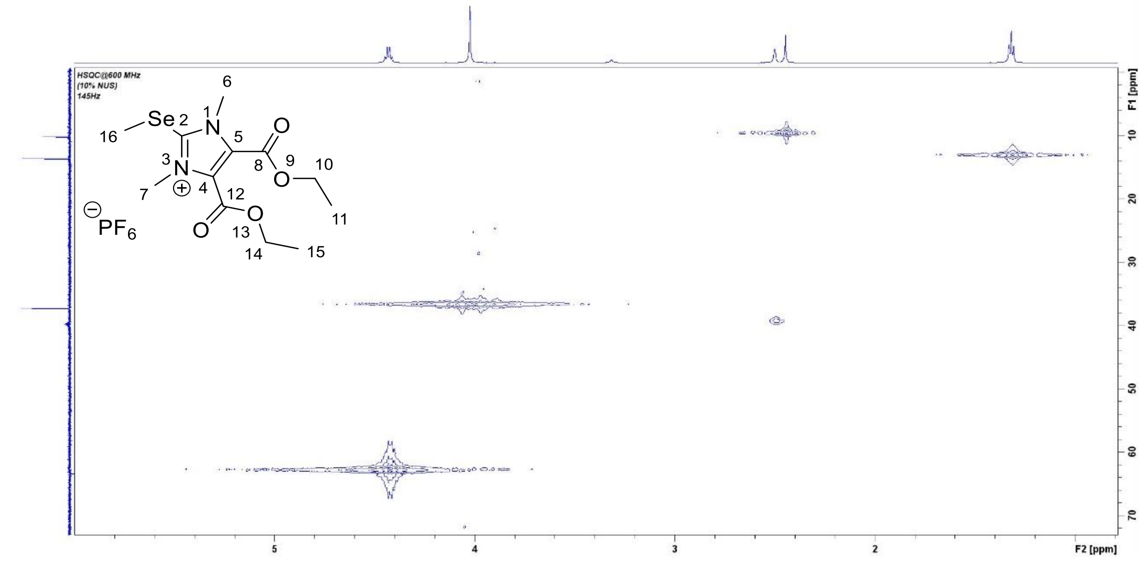

Figure S65: HSQC NMR spectrum of **13e** in DMSO-d_6.

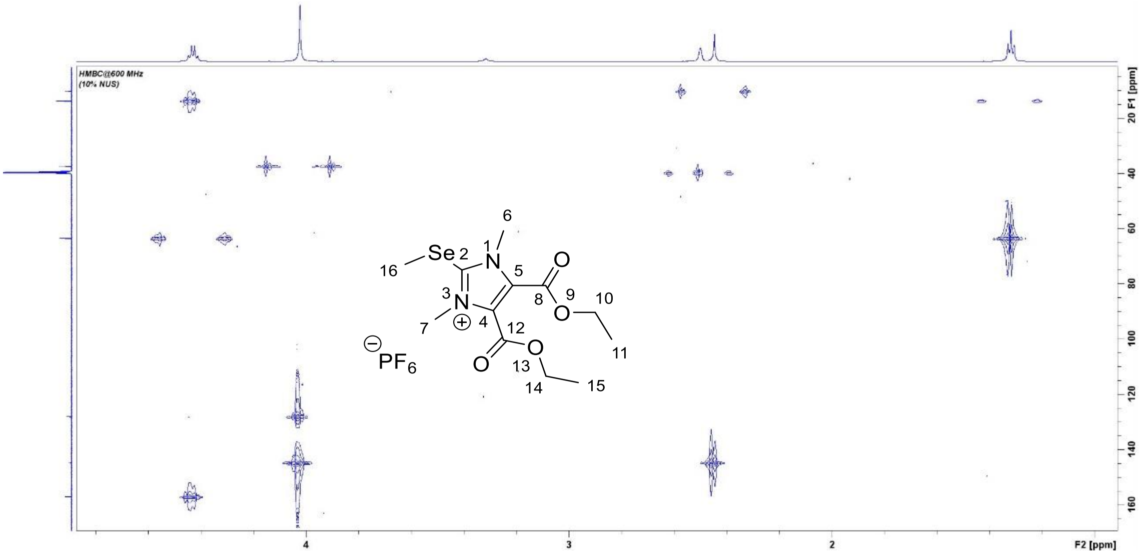

Figure S66: HMBC NMR spectrum of **13e** in DMSO-d_6.

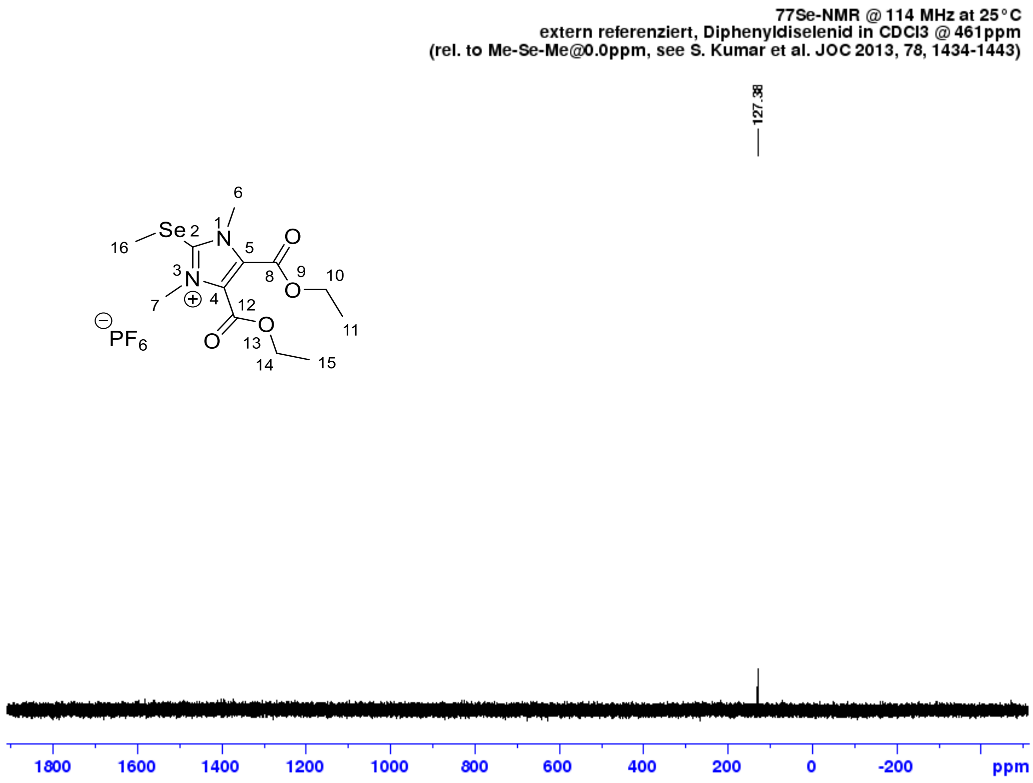

Figure S67: ^{77}Se NMR spectrum of **13e** in $CDCl_3$.

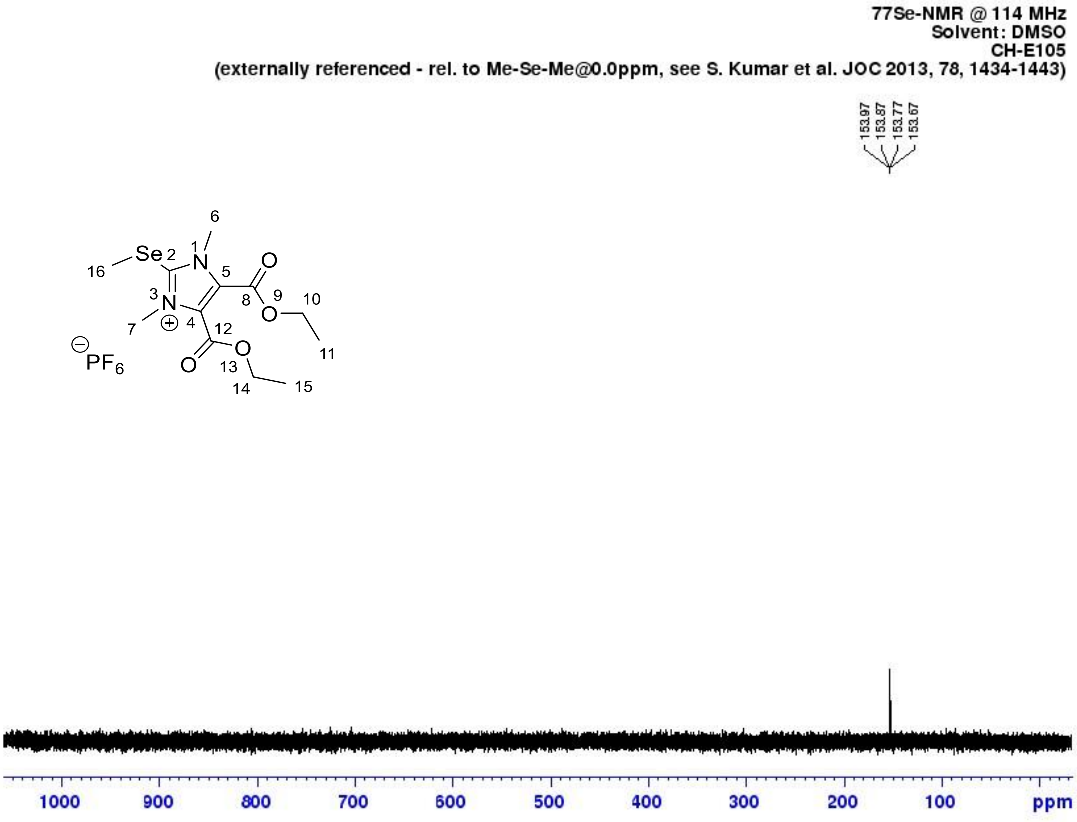

Figure S68: ^{77}Se NMR spectrum of **13e** in DMSO-d_6.

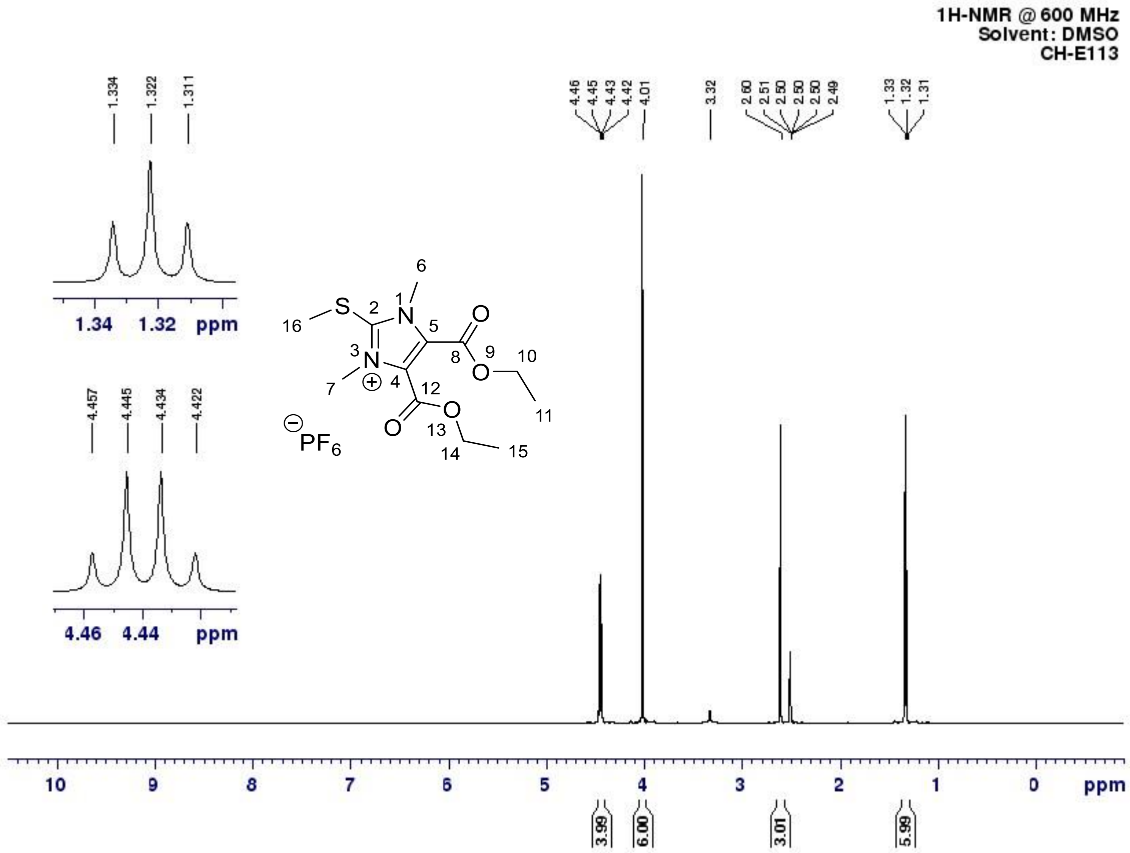

Figure S69: ^{1}H NMR spectrum of **13f** in DMSO-d$_6$.

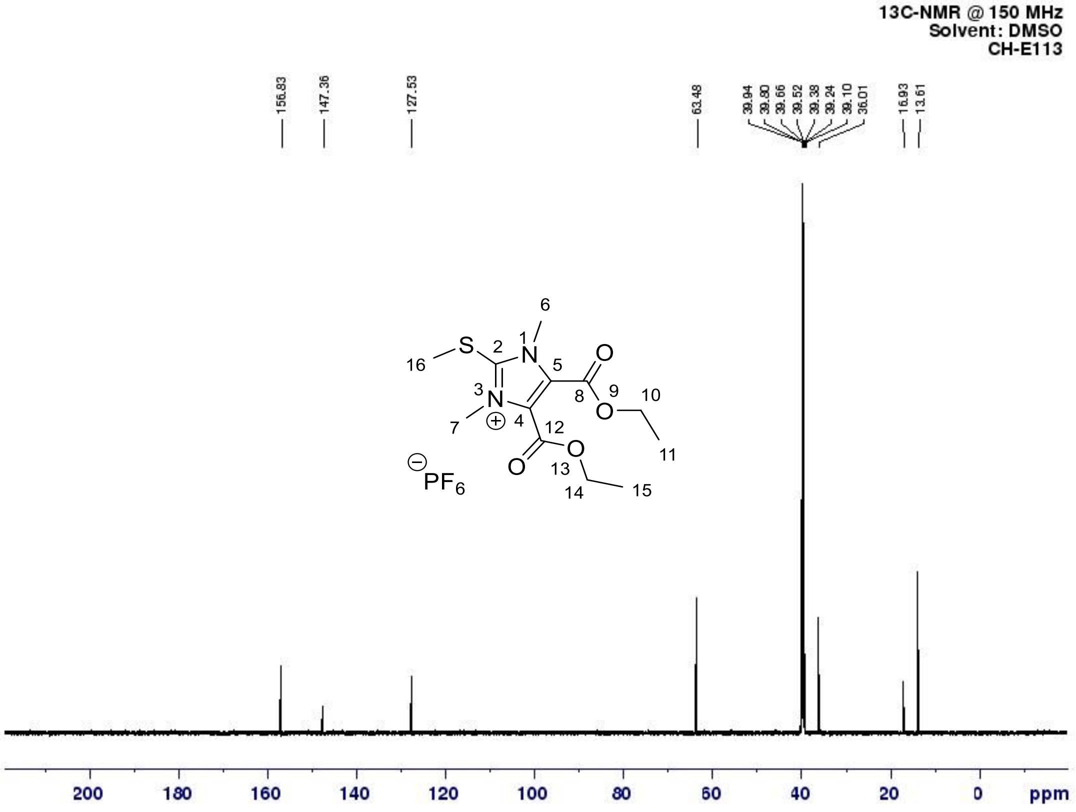

Figure S70: $^{13}C\{^{1}H\}$ NMR spectrum of **13f** in DMSO-d$_6$.

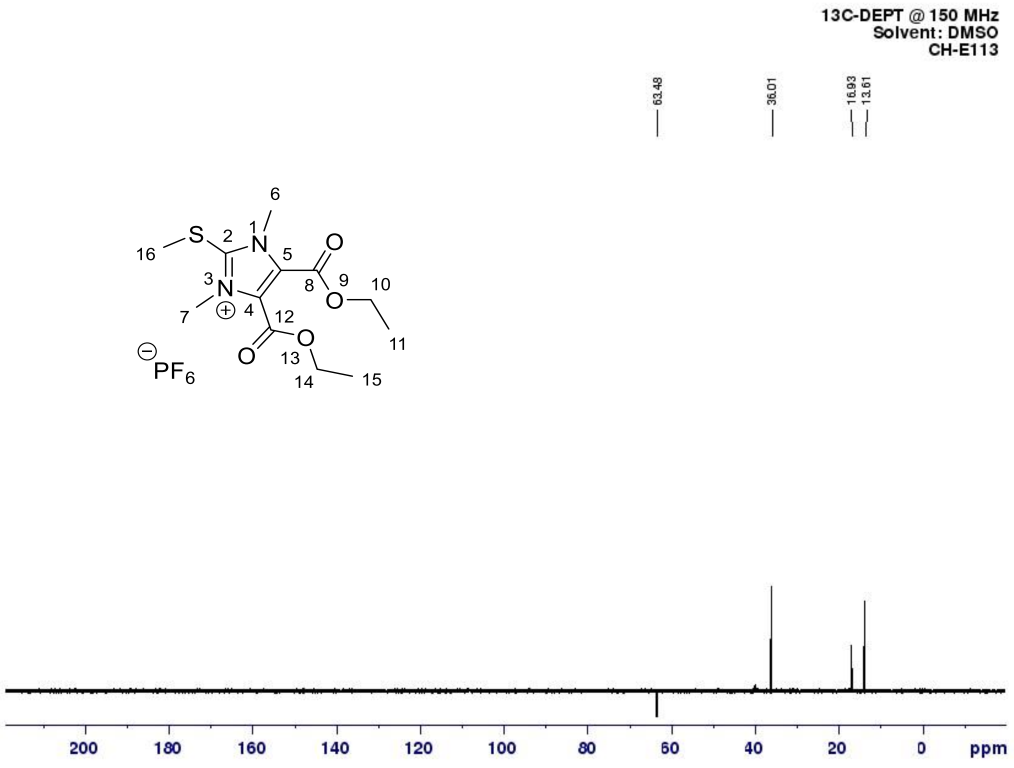

Figure S71: $^{13}C\{^{1}H\}$ DEPT NMR spectrum of **13f** in DMSO-d$_6$.

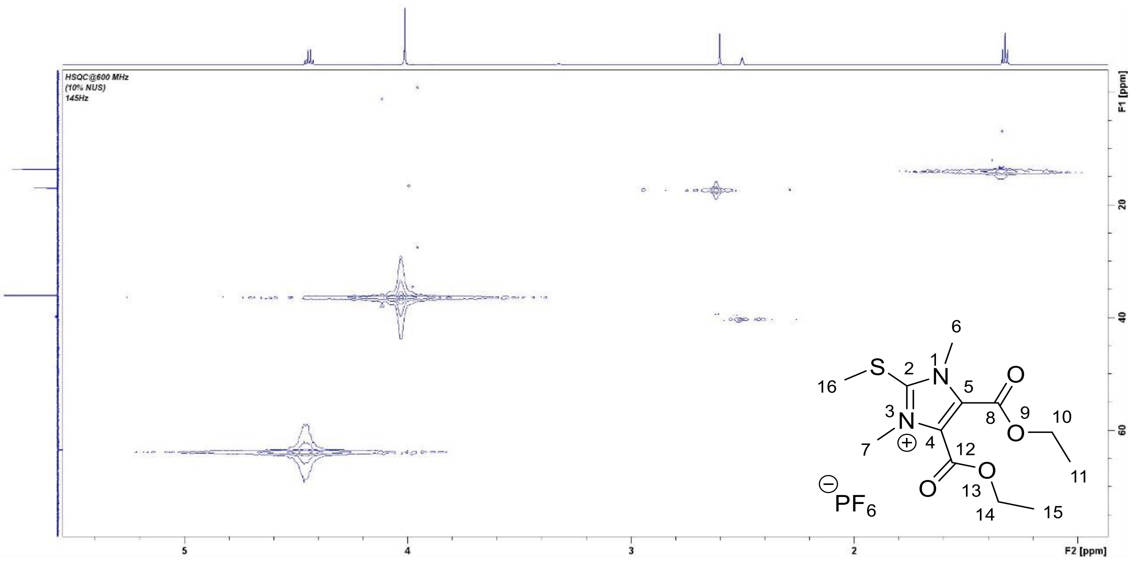

Figure 72: HSQC NMR spectrum of **13f** in DMSO-d$_6$.

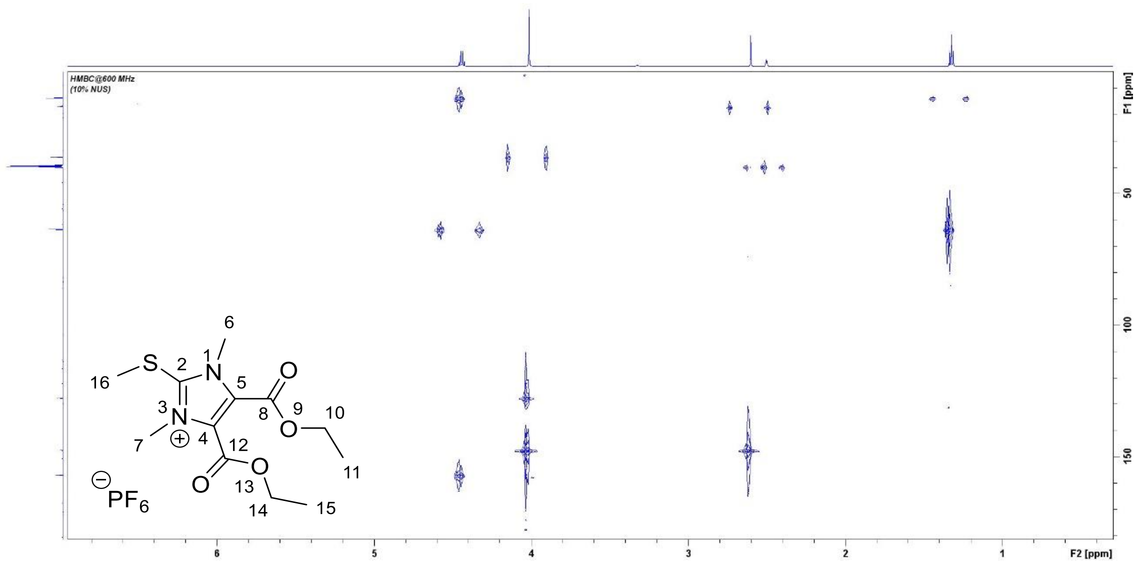

Figure 73: HMBC NMR spectrum of **13f** in DMSO-d$_6$.

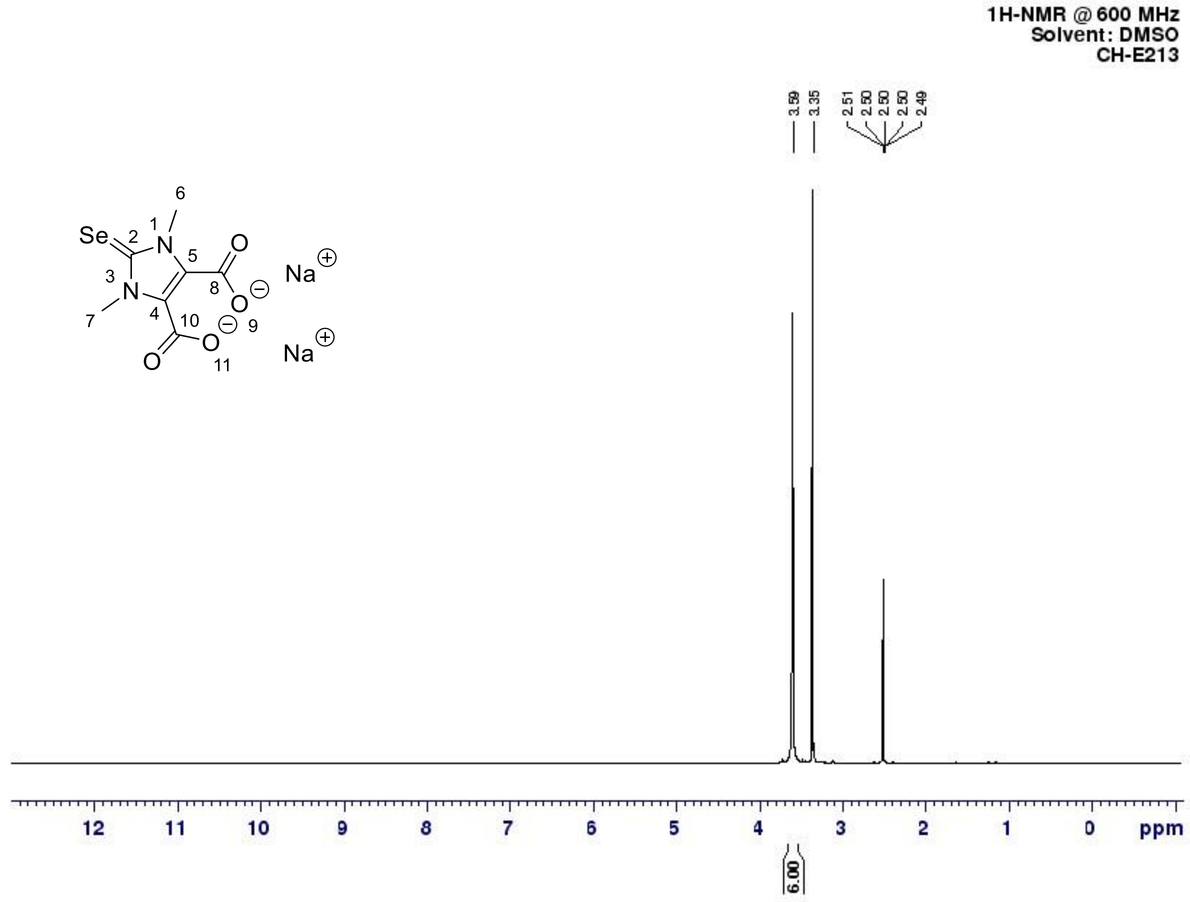

Figure S74: ^{1}H NMR spectrum of **14a** in DMSO-d$_6$.

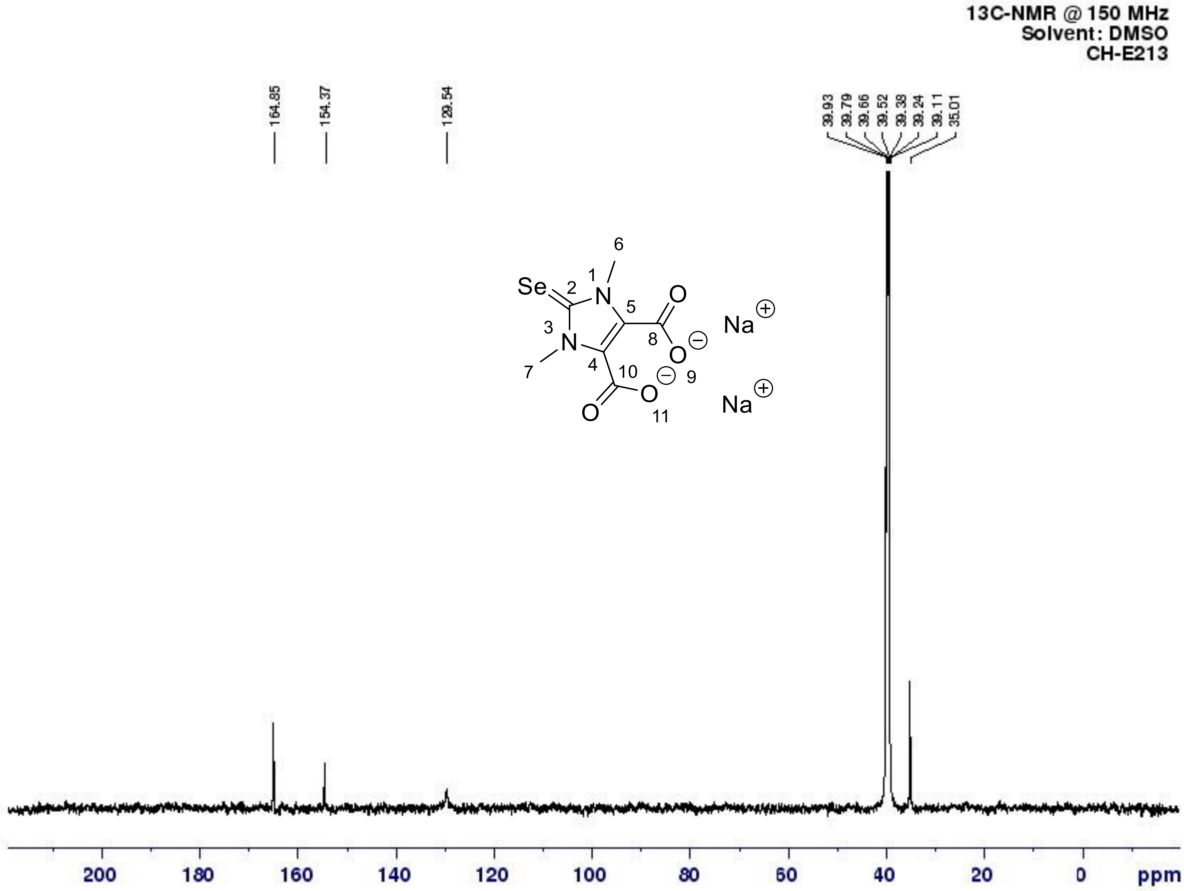

Figure S75: $^{13}C\{^{1}H\}$ NMR spectrum of **14a** in DMSO-d$_6$.

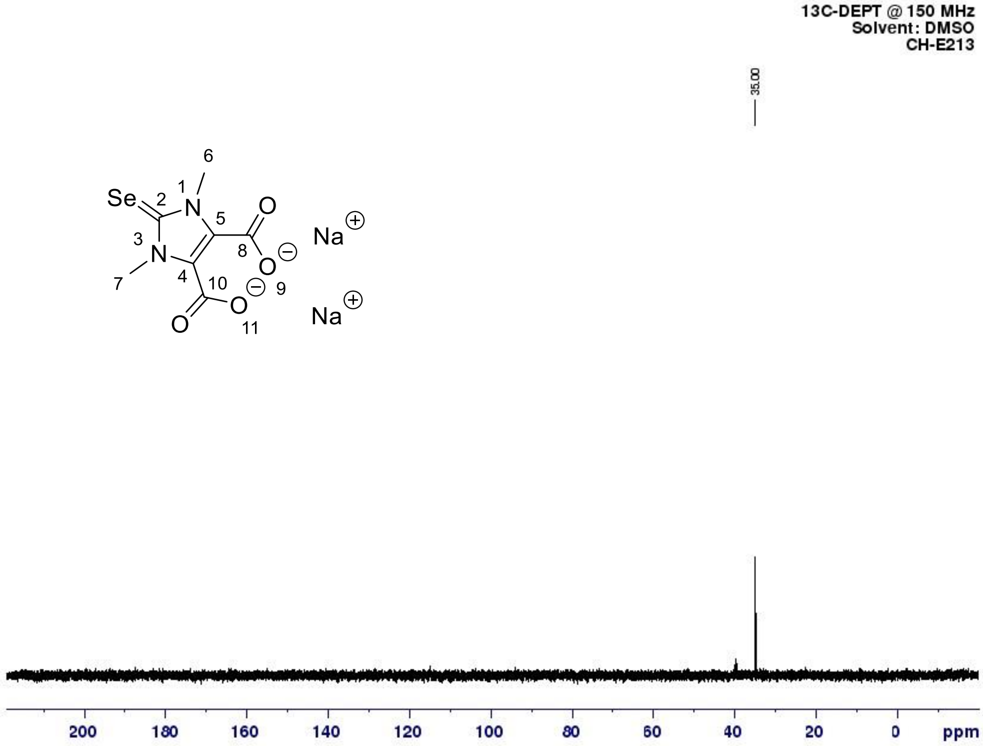

Figure S76: $^{13}C\{^{1}H\}$ DEPT NMR spectrum of **14a** in DMSO-d$_6$.

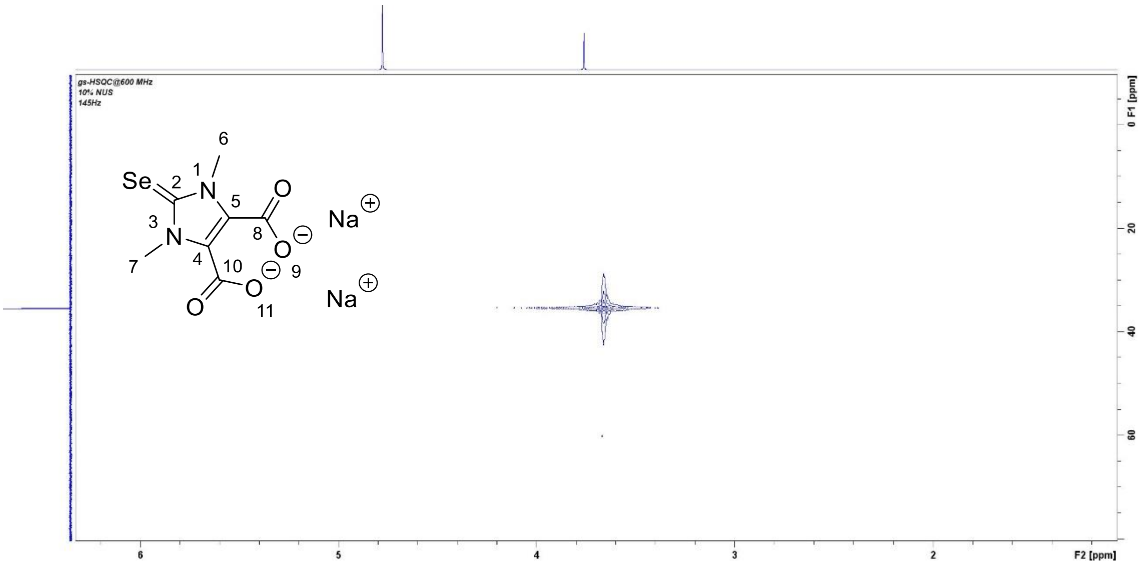

Figure S77: HSQC NMR spectrum of **14a** in DMSO-d_6.

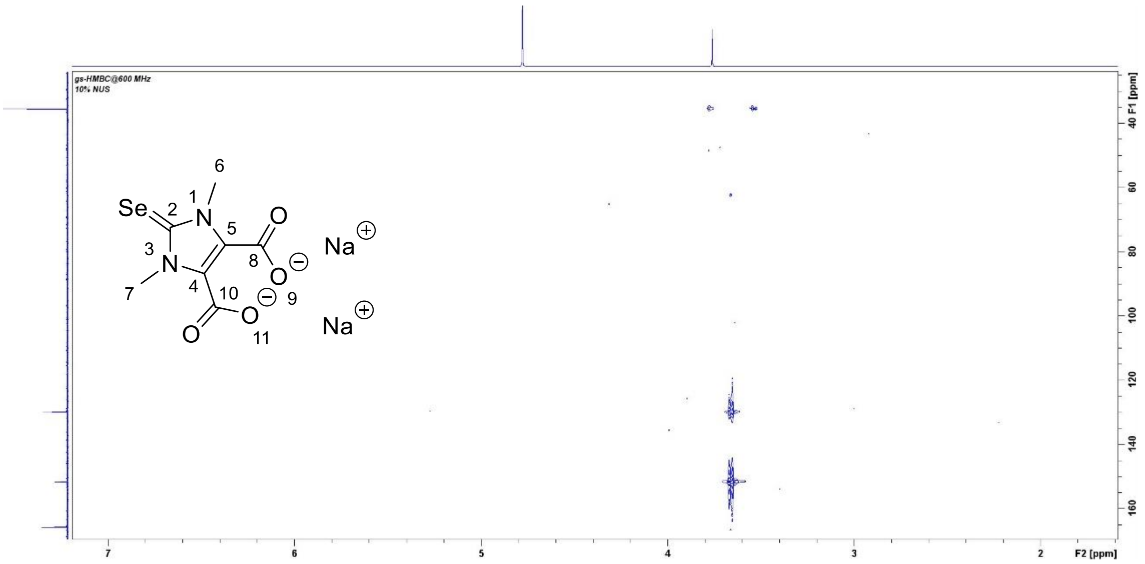

Figure S78: HMBC NMR spectrum of **14a** in DMSO-d_6.

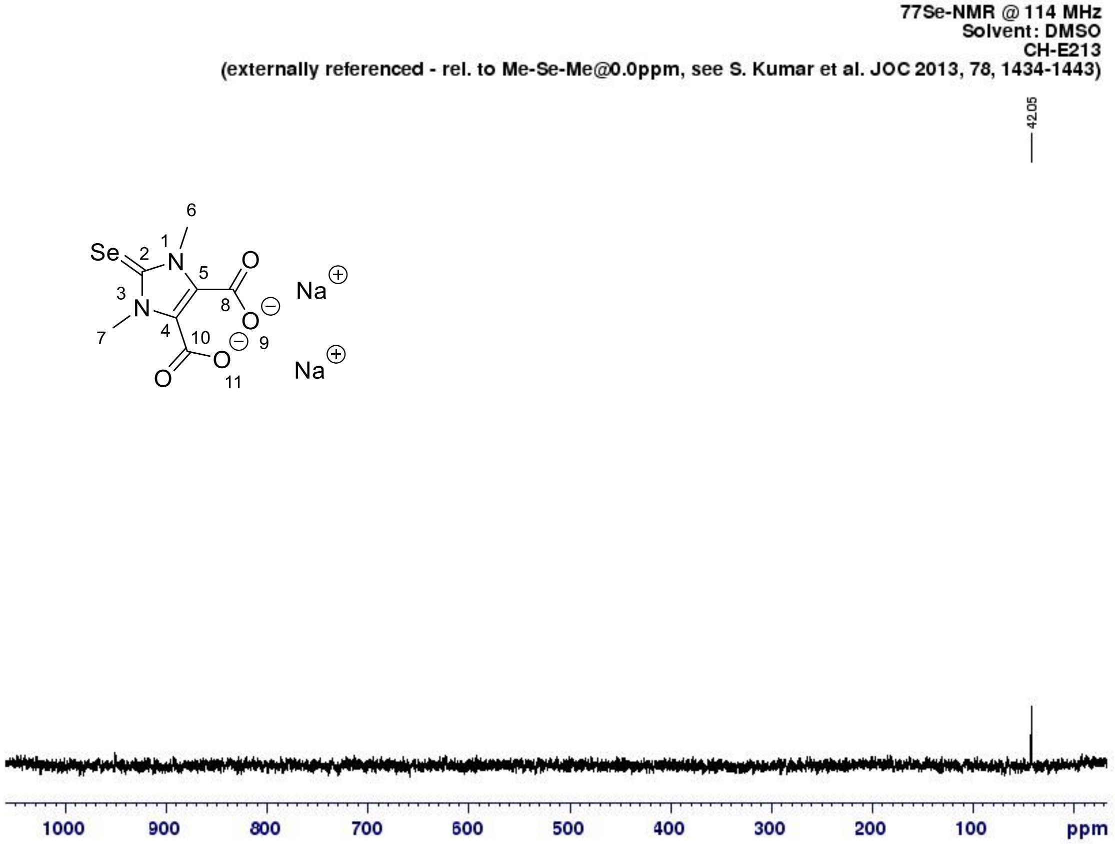

Figure S79: ^{77}Se NMR spectrum of **14a** in DMSO-d$_6$.

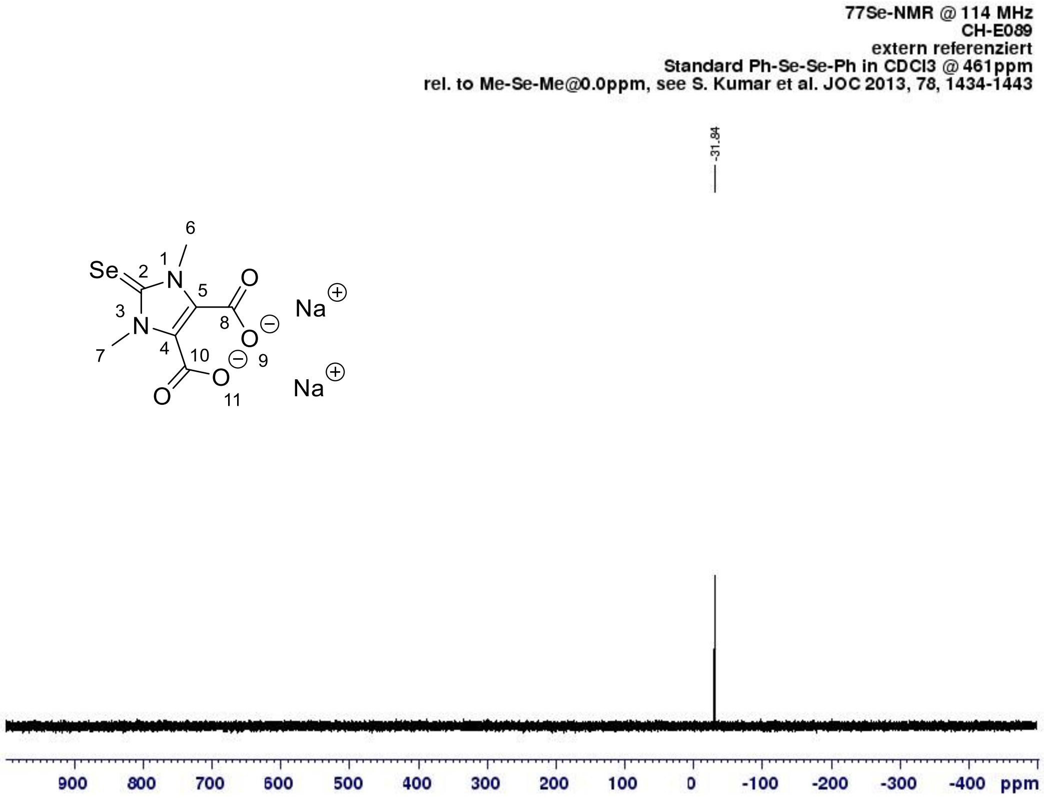

Figure S80: ^{77}Se NMR spectrum of **14a** in $CDCl_3$.

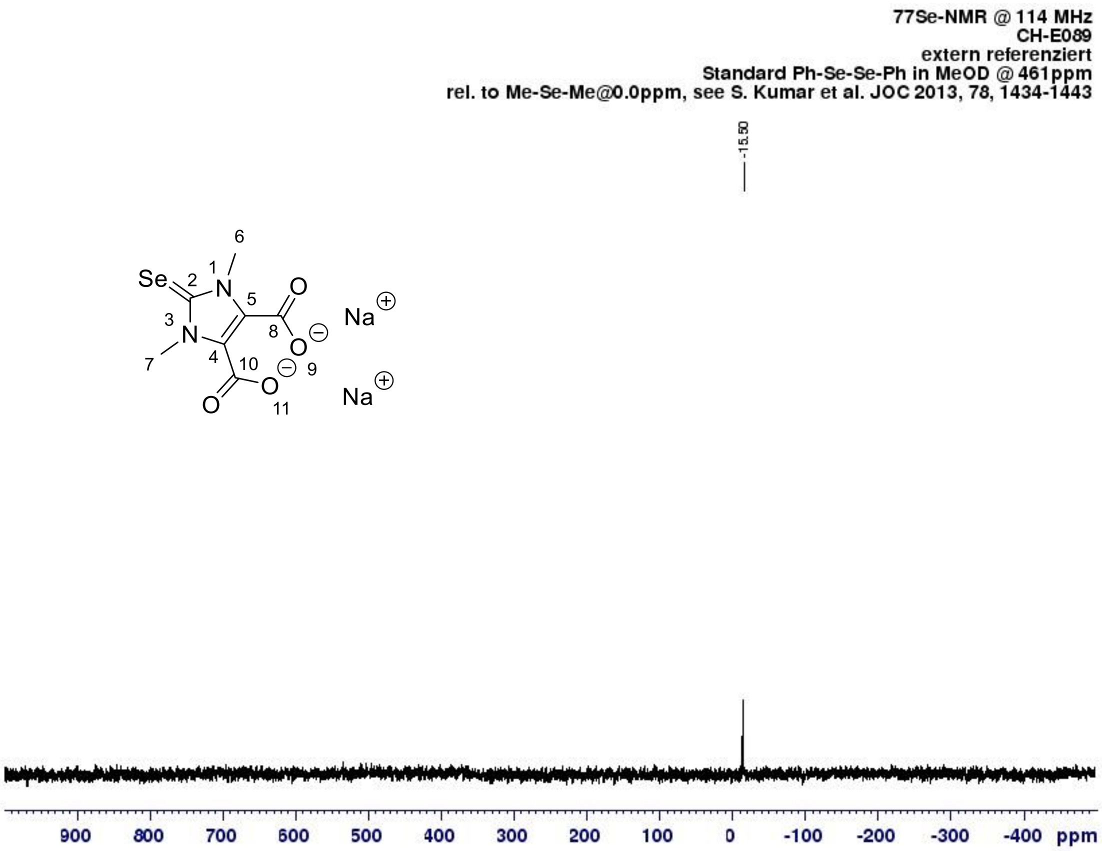

Figure S81: ^{77}Se NMR spectrum of **14a** in MeOD.

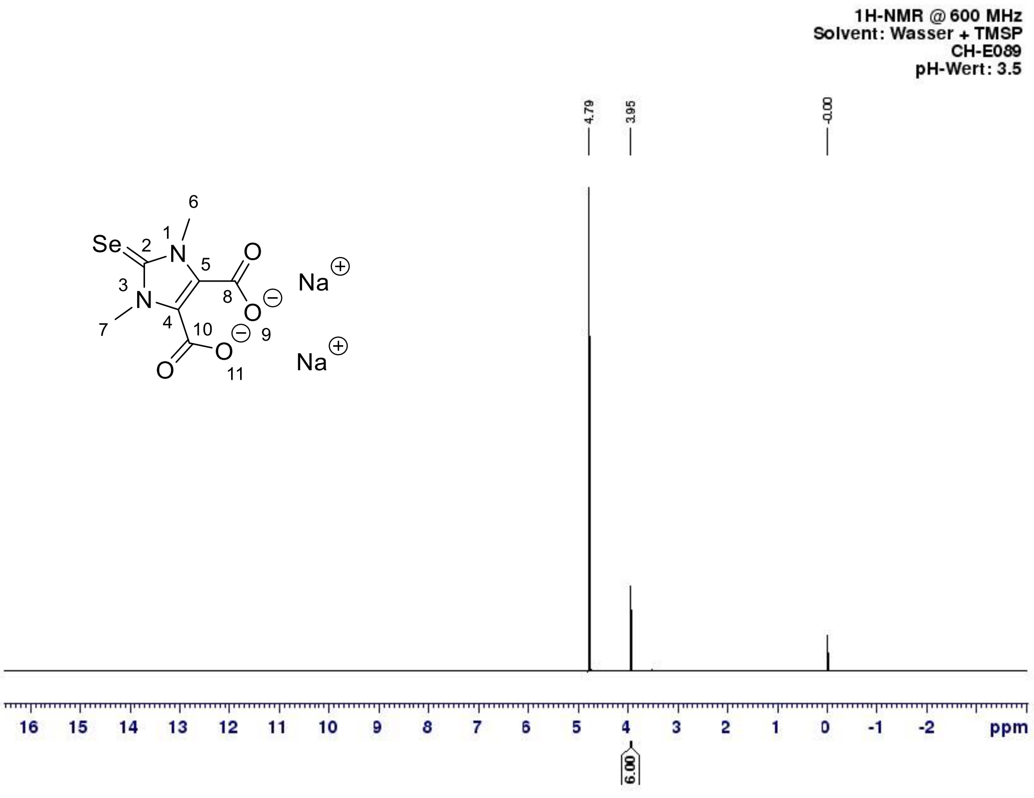

Figure S82: ^{1}H NMR spectrum of **14a** in D2O, pH 3.5.

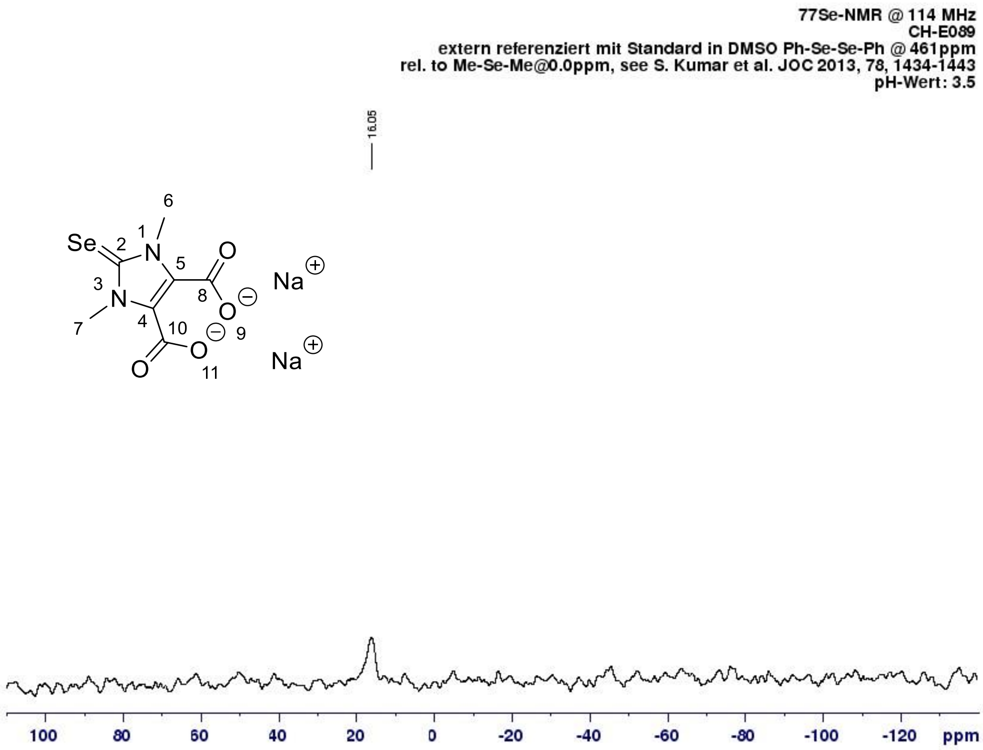

Figure S83: ^{77}Se NMR spectrum of **14a** in D_2O, pH 3.5.

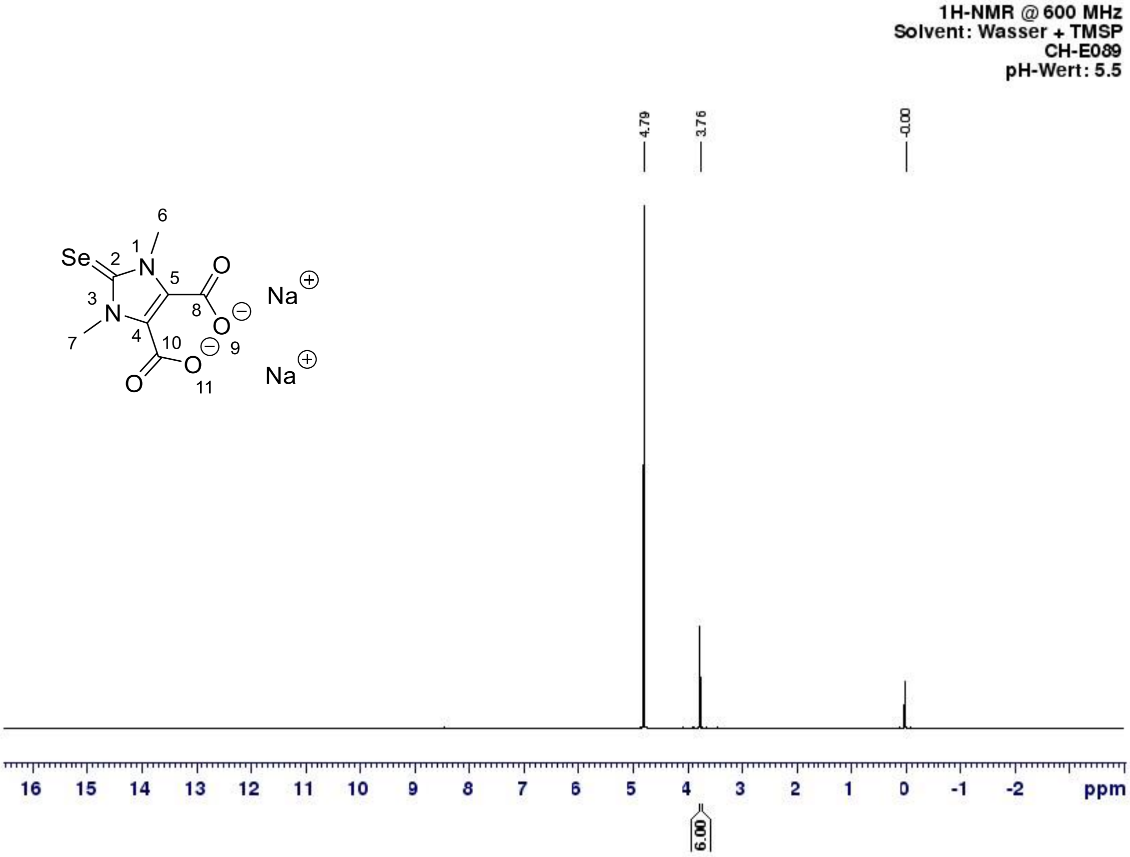

Figure S84: ^{1}H NMR spectrum of **14a** in D_2O, pH 5.5.

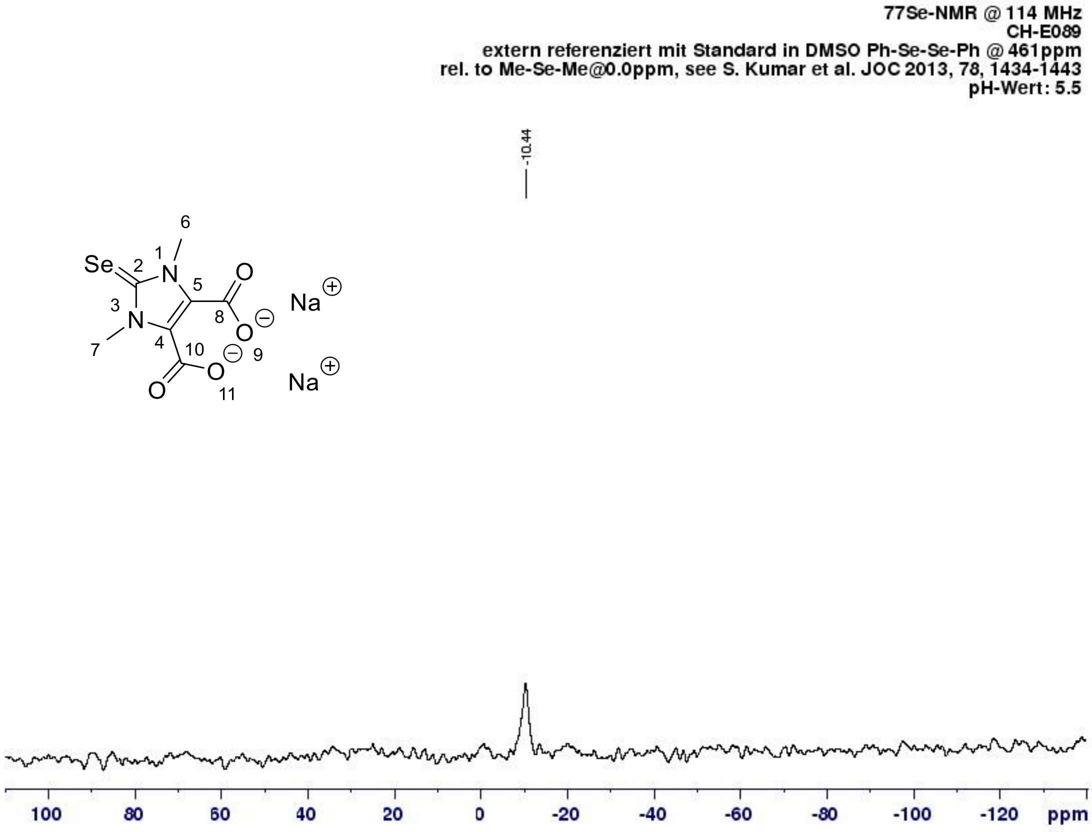

Figure S85: ^{77}Se NMR spectrum of **14a** in D_2O, pH 5.5.

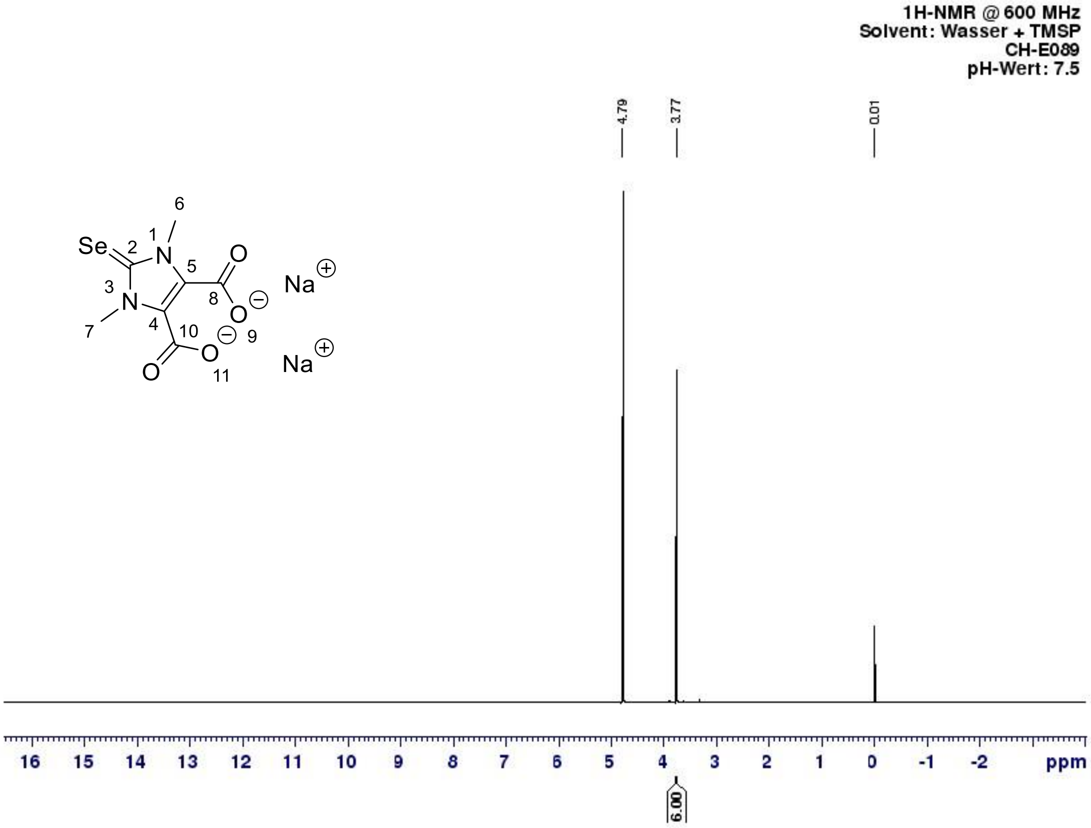

Figure S86: ^{1}H NMR spectrum of **14a** in D_2O, pH 7.5.

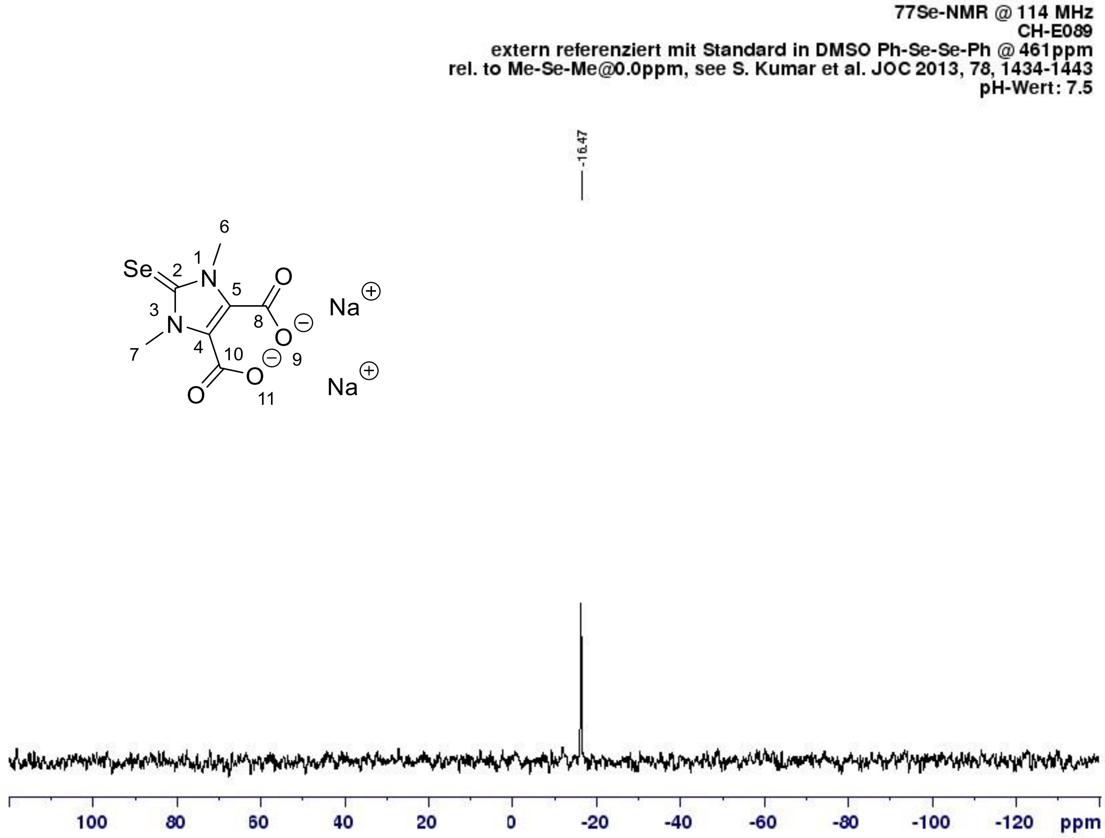

Figure S87: ^{77}Se NMR spectrum of **14a** in D_2O, pH 7.5.

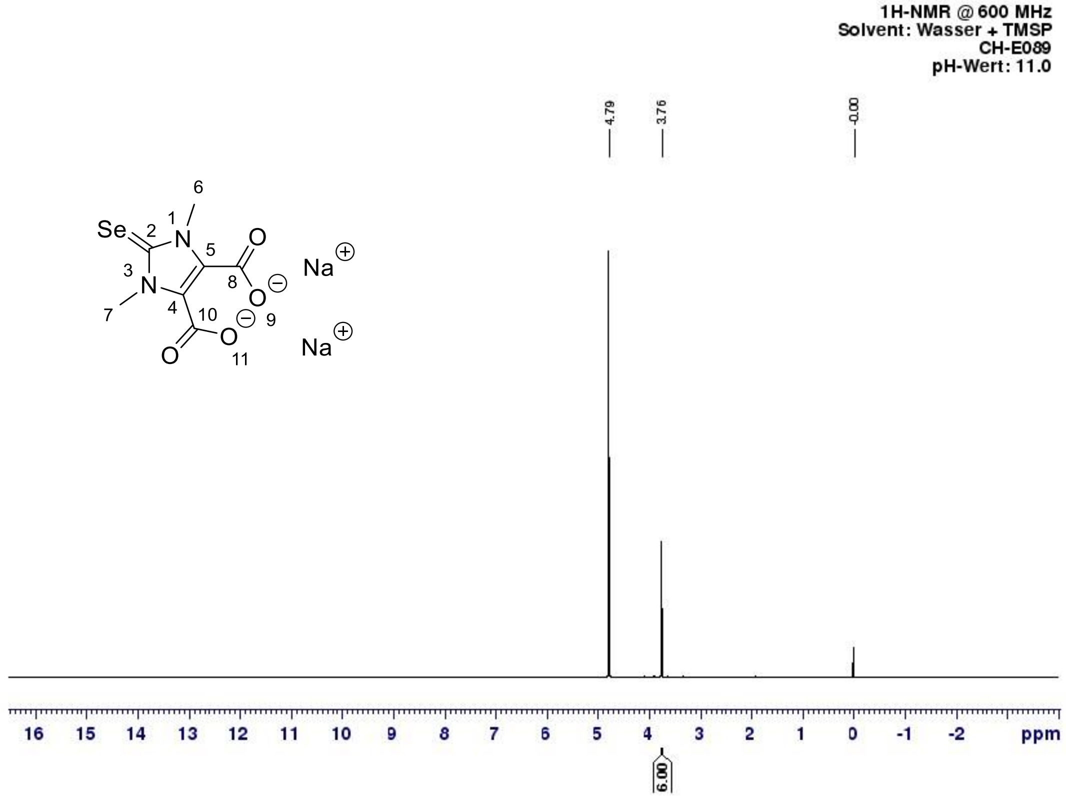

Figure S88: ^{1}H NMR spectrum of **14a** in D_2O, pH 11.0.

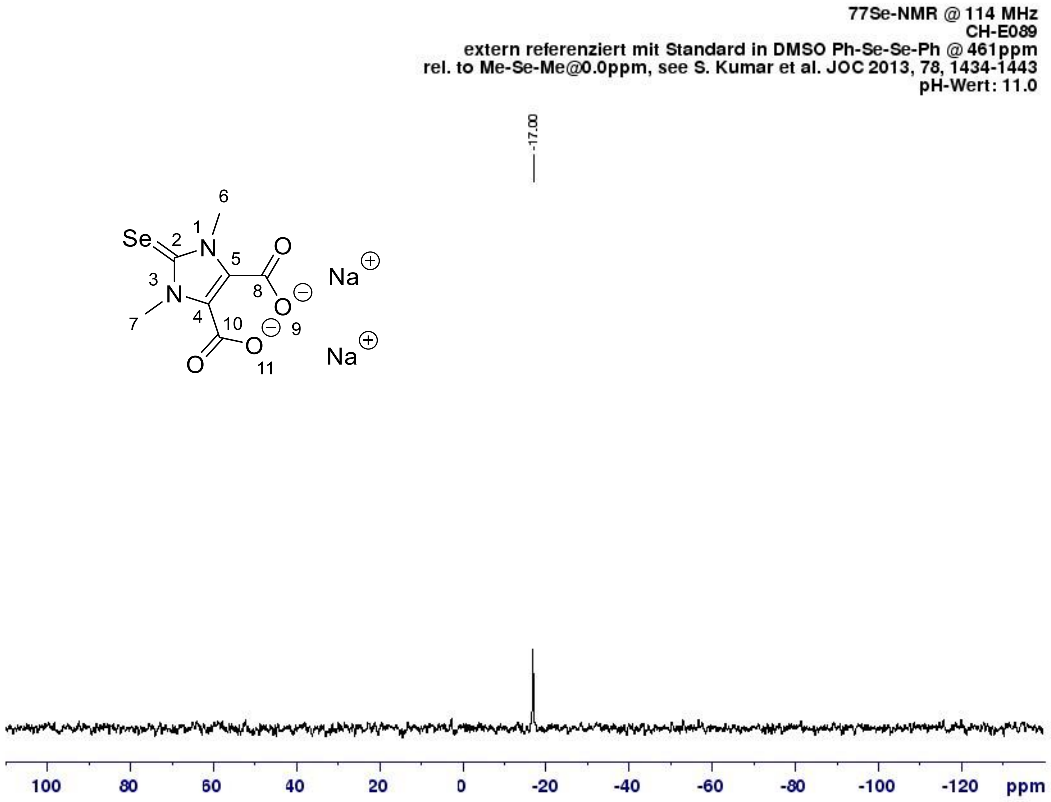

Figure S89: ^{77}Se NMR spectrum of **14a** in D_2O, pH 11.0.

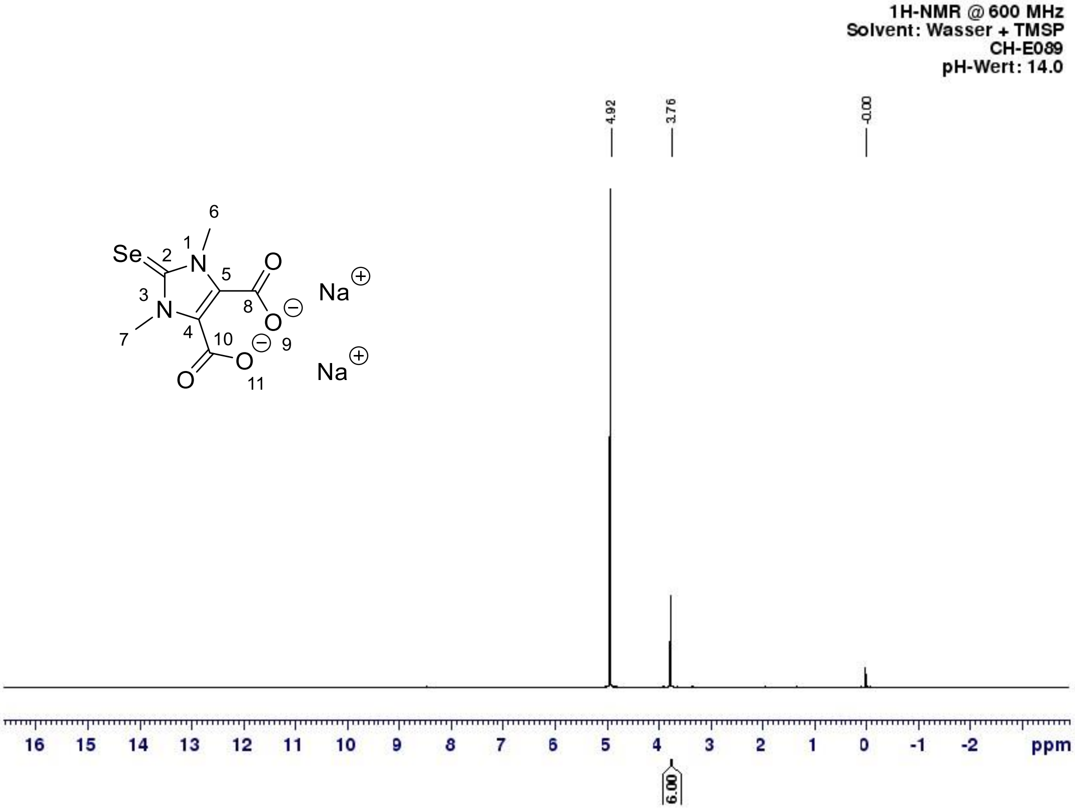

Figure S90: ^{1}H NMR spectrum of **14a** in D_2O, pH 14.0.

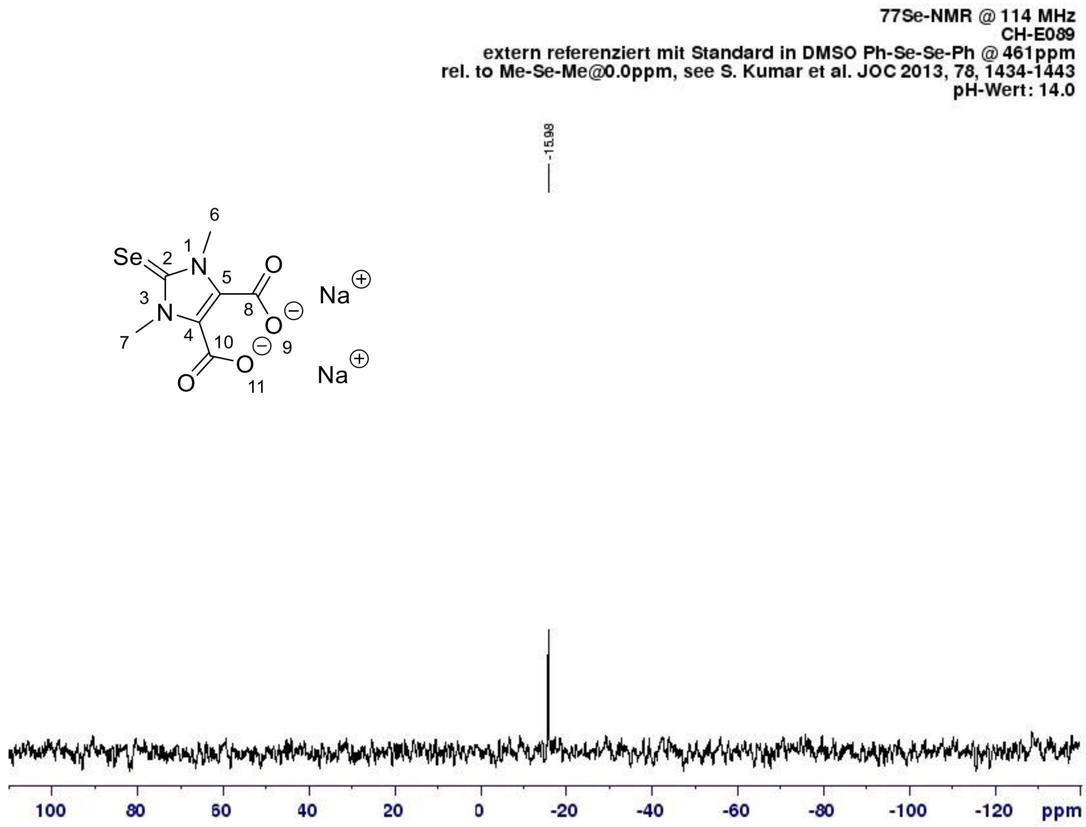

Figure S91: ^{77}Se NMR spectrum of **14a** in D_2O, pH 14.0.

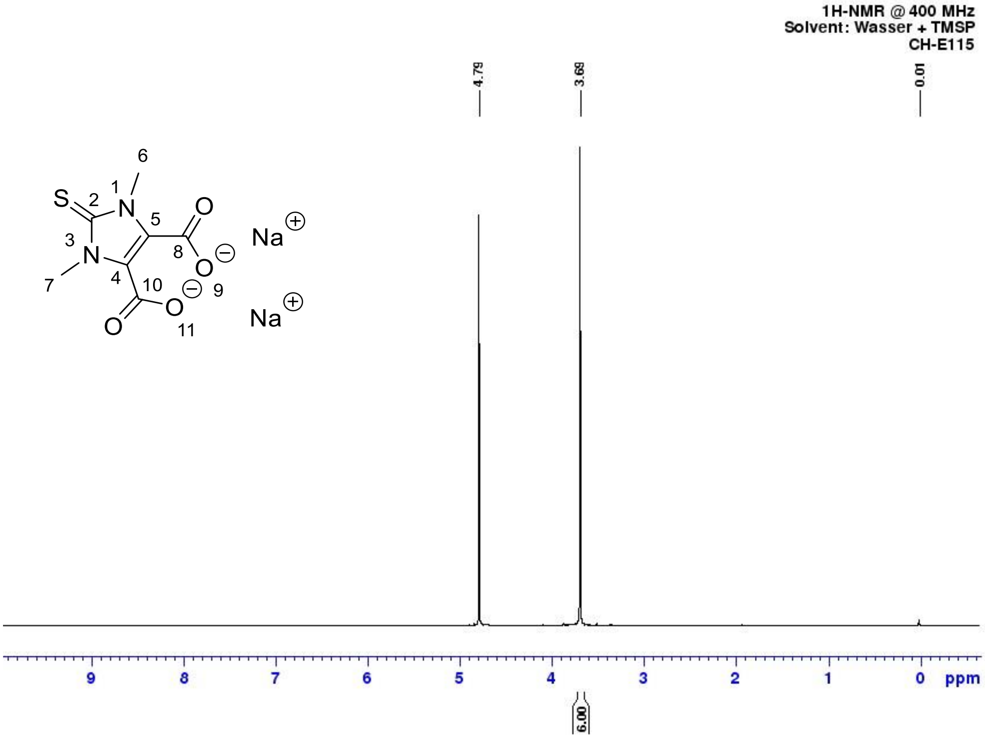

Figure S92: ^{1}H NMR spectrum of **14b** in D_2O.

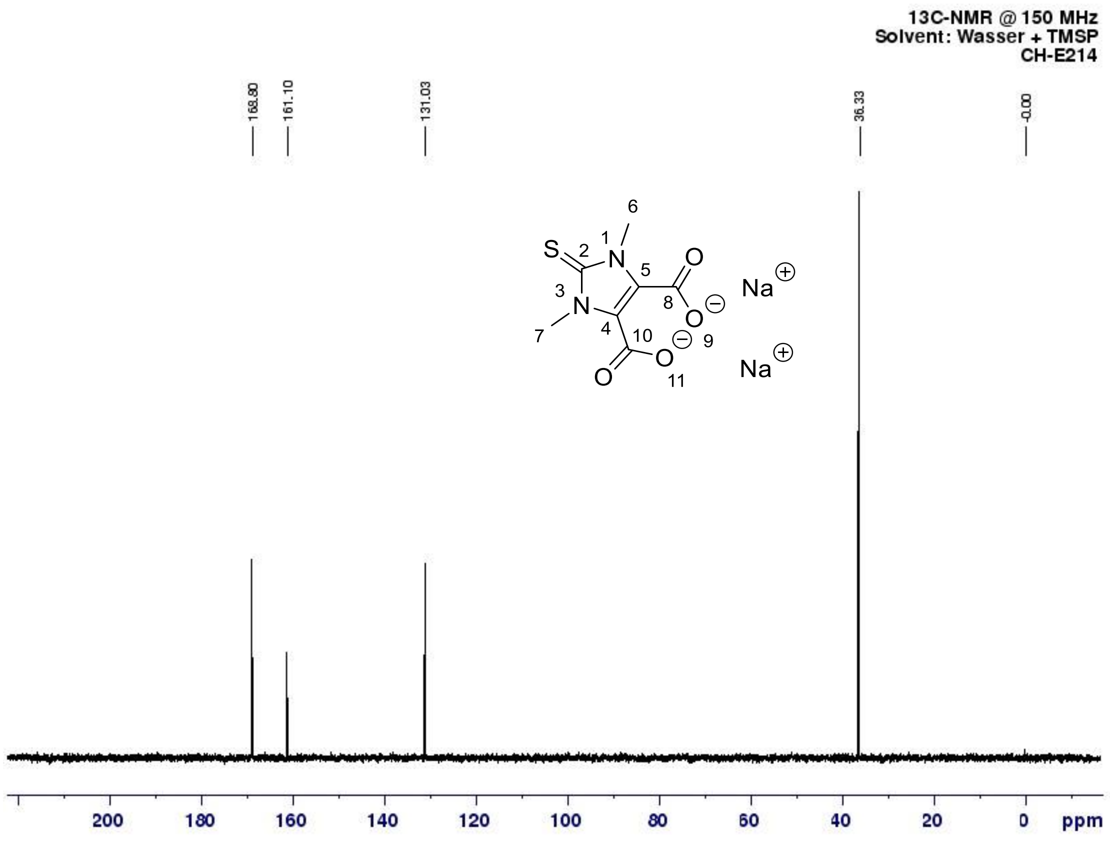

Figure S93: $^{13}C\{^{1}H\}$ NMR spectrum of **14b** in D_2O.

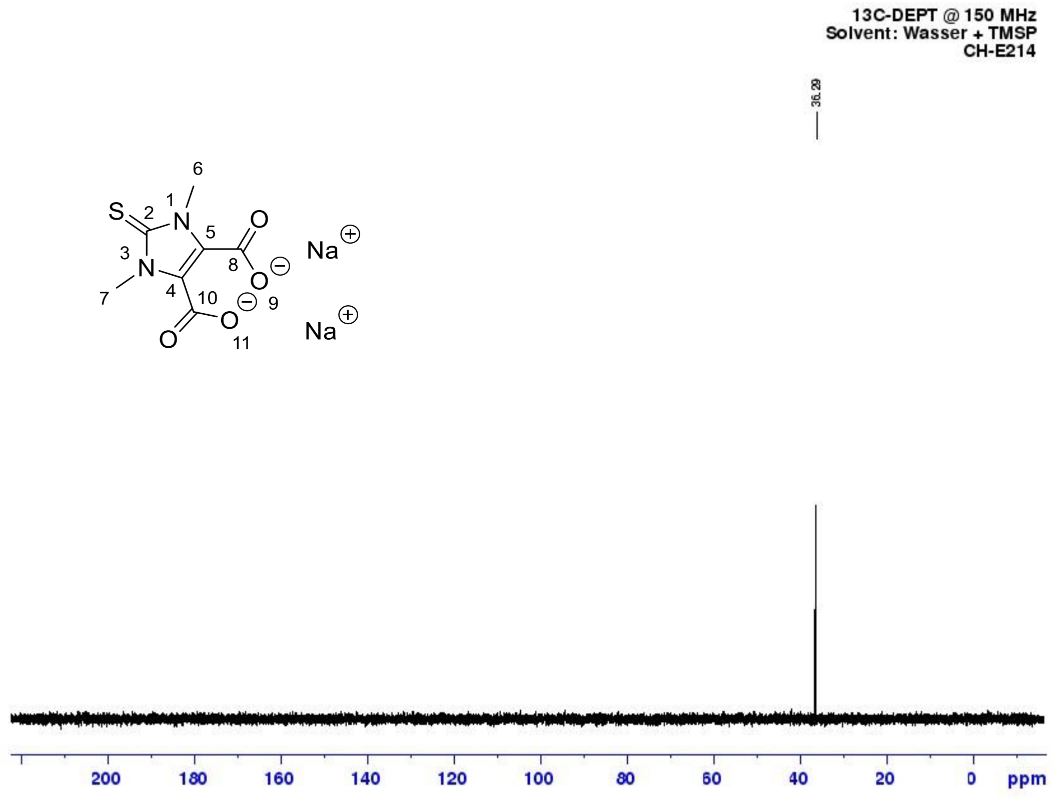

Figure S94: $^{13}C\{^{1}H\}$ DEPT NMR spectrum of **14b** in D_2O.

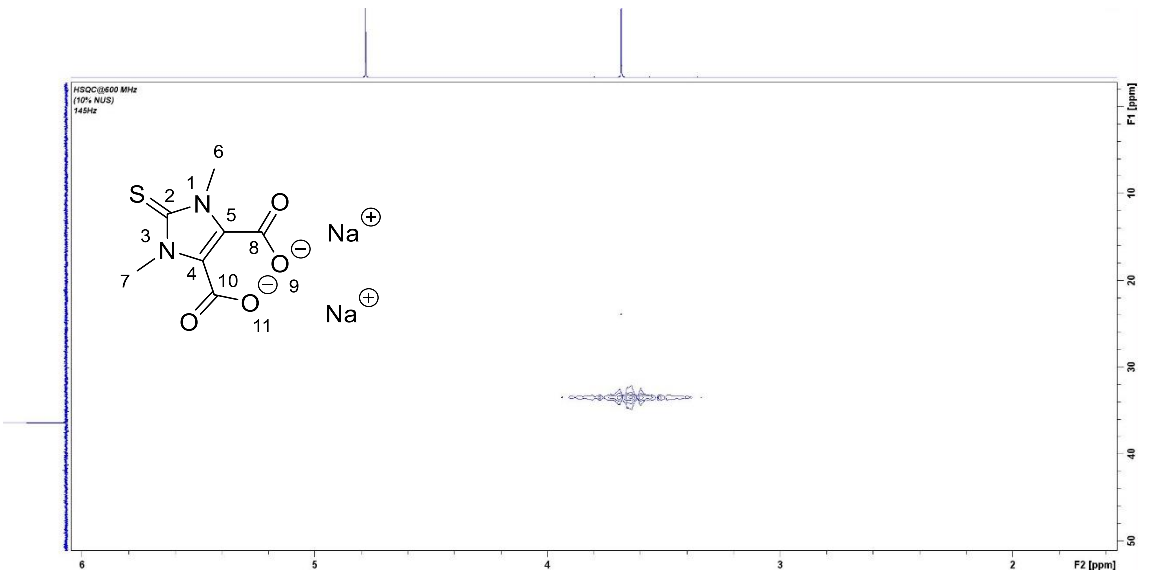

Figure S95: HSQC NMR spectrum of **14b** in D_2O.

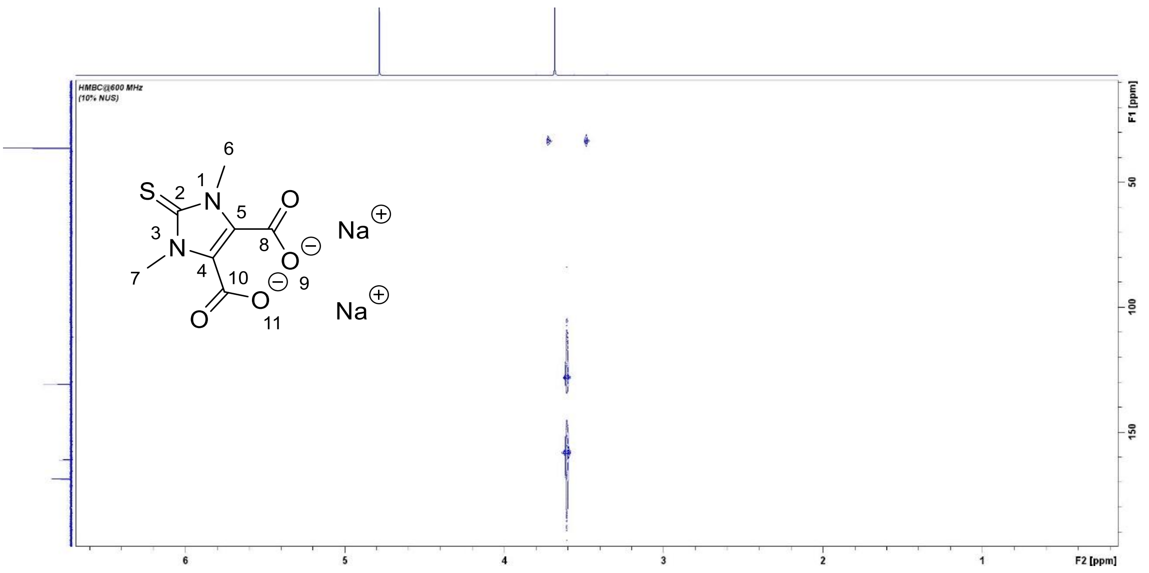

Figure S96: HMBC NMR spectrum of **14b** in D_2O.

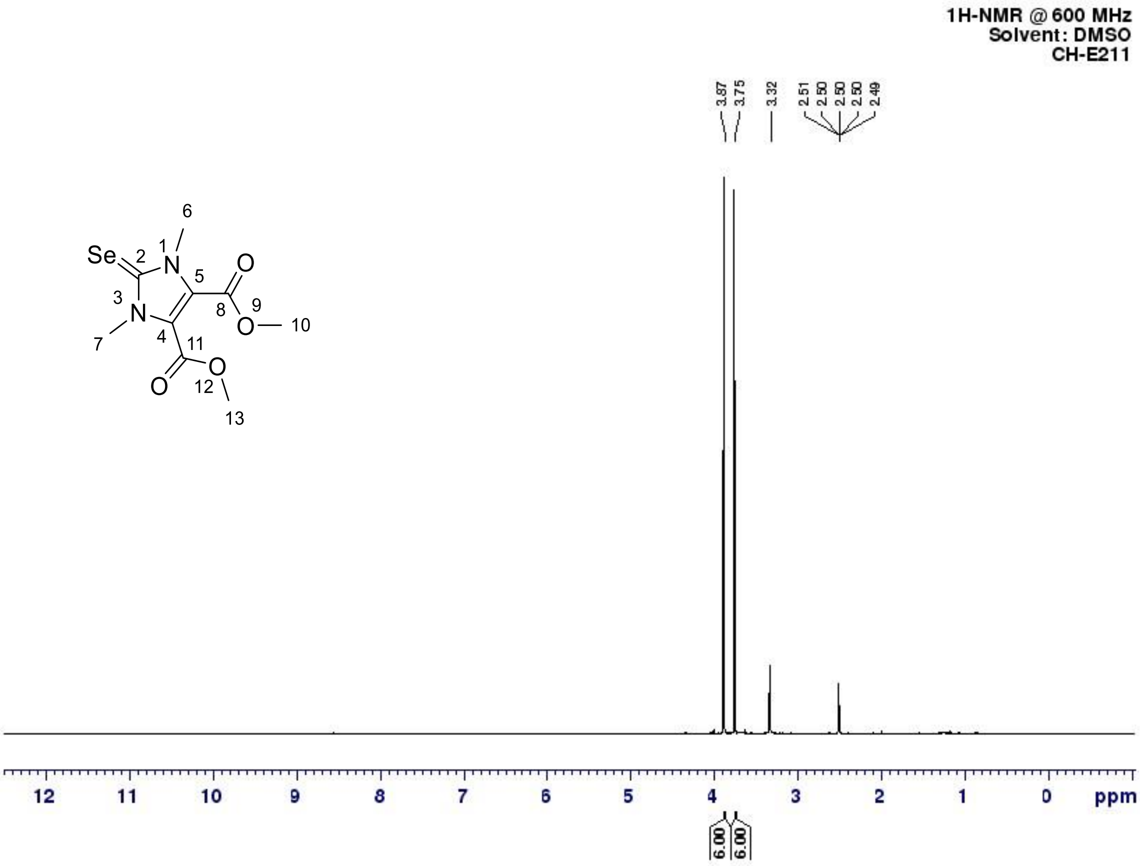

Figure S97: ^{1}H NMR spectrum of **15** in DMSO-d$_6$.

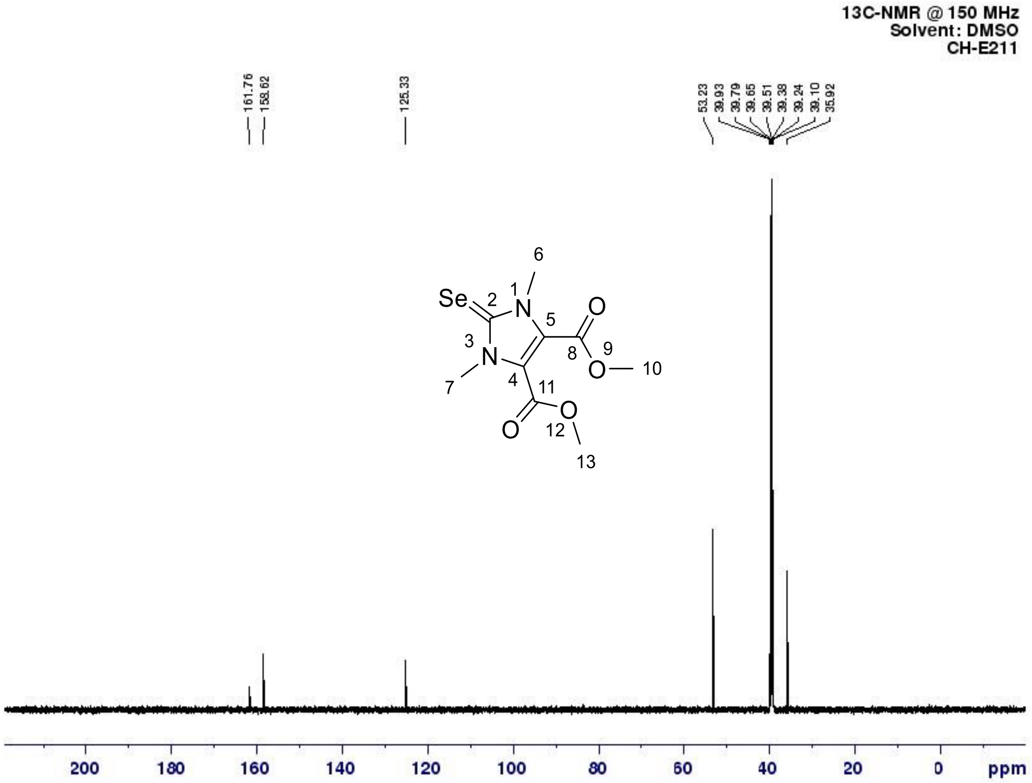

Figure S98: ^{13}C{^{1}H} NMR spectrum of **15** in DMSO-d$_6$.

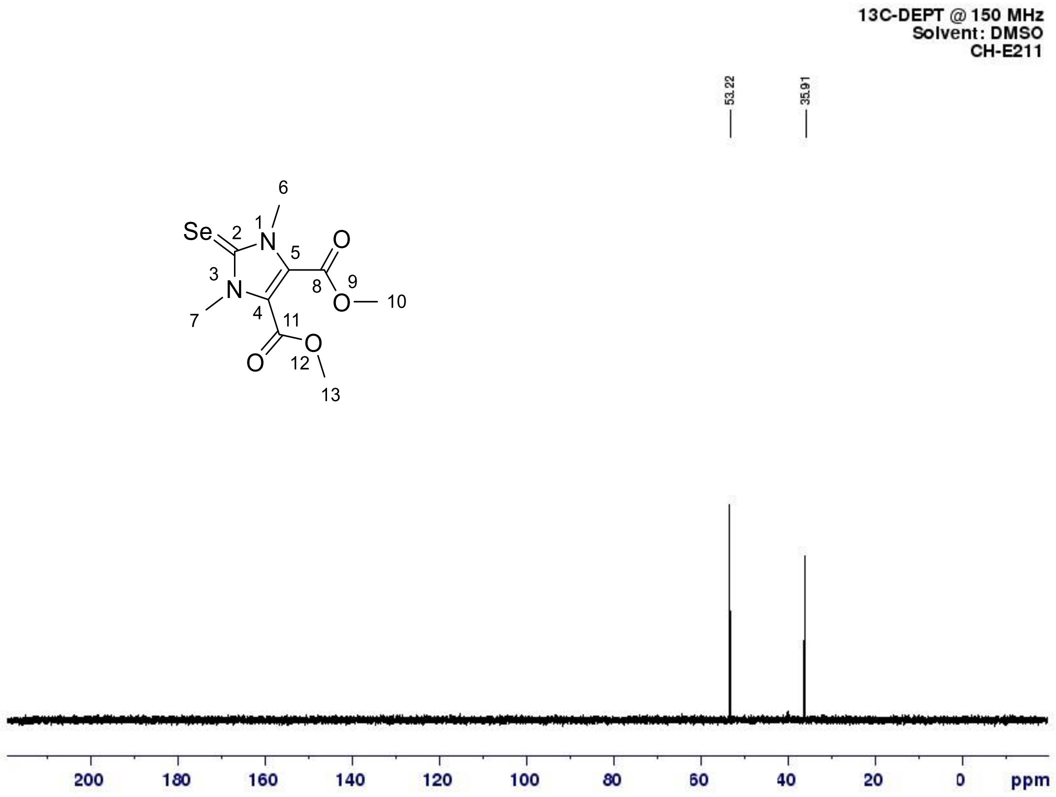

Figure S99: $^{13}C\{^{1}H\}$ DEPT NMR spectrum of **15** in DMSO-d$_6$.

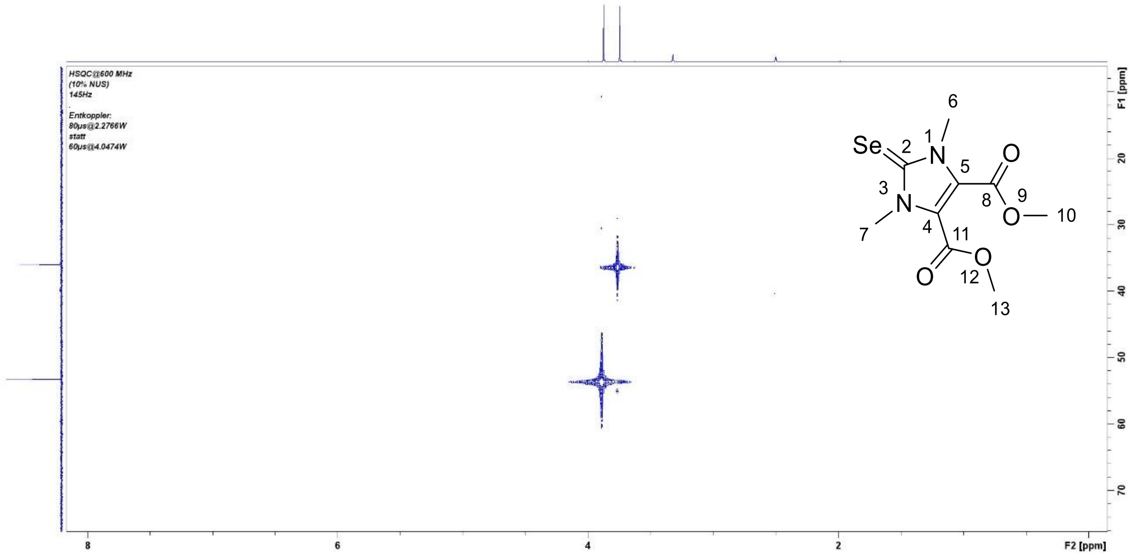

Figure S100: HSQC NMR spectrum of **15** in DMSO-d$_6$.

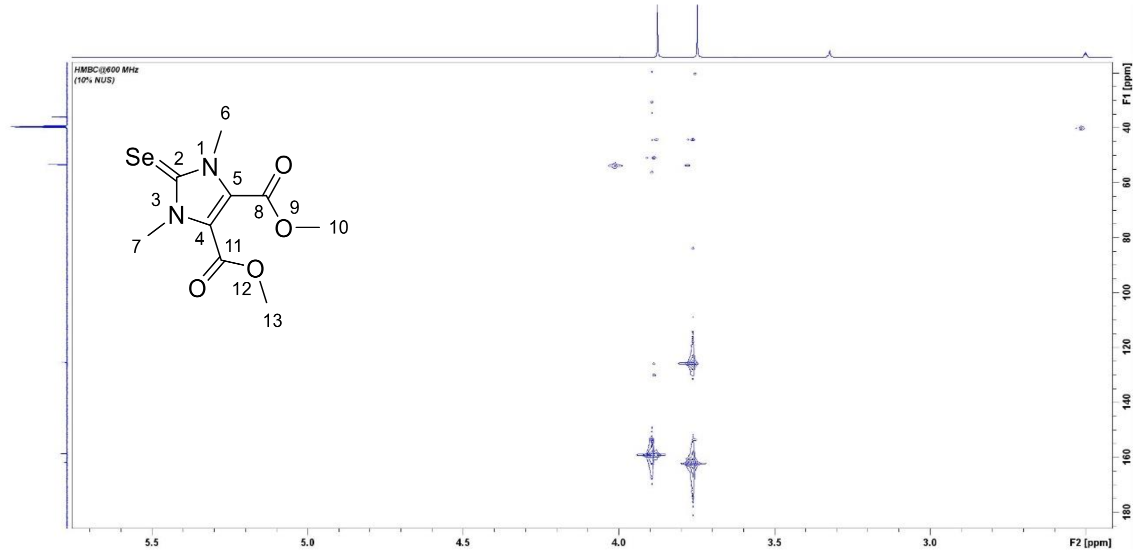

Figure S101: HMBC NMR spectrum of **15** in DMSO-d$_6$.

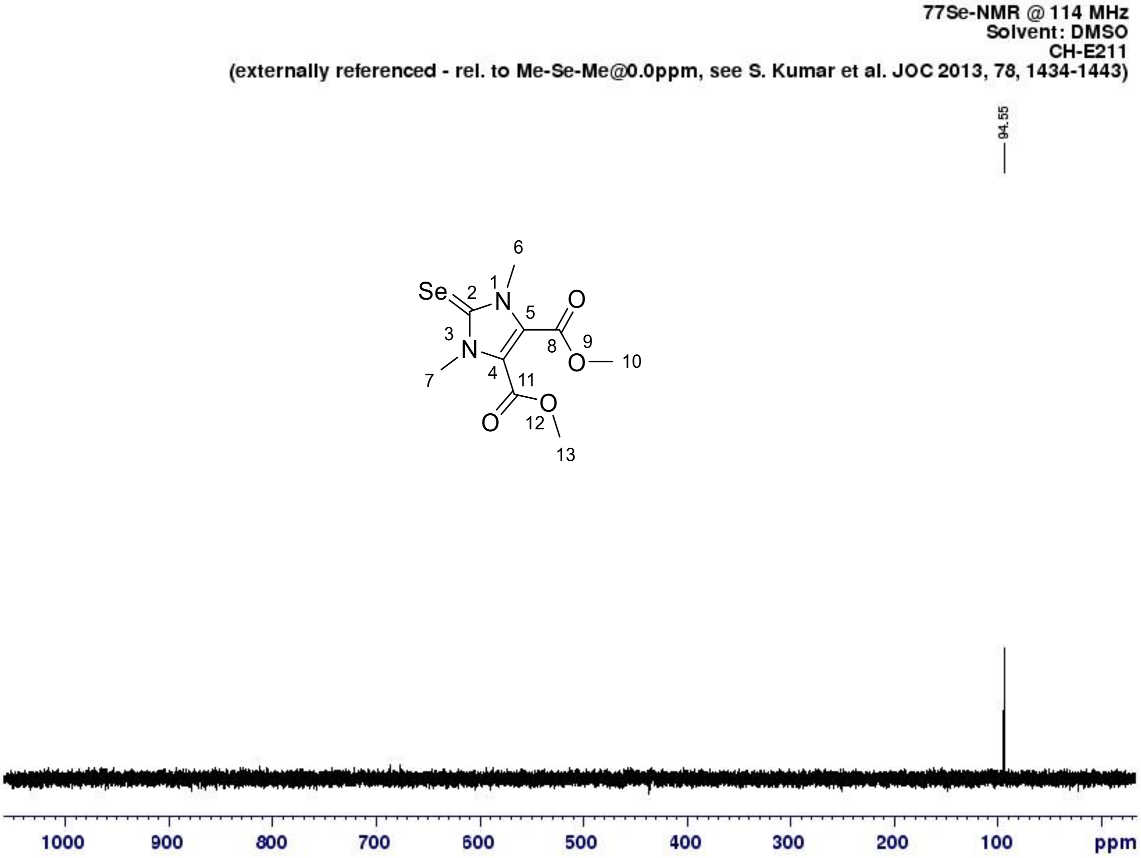

Figure S102: ^{77}Se NMR spectrum of **15** in DMSO-d$_6$.

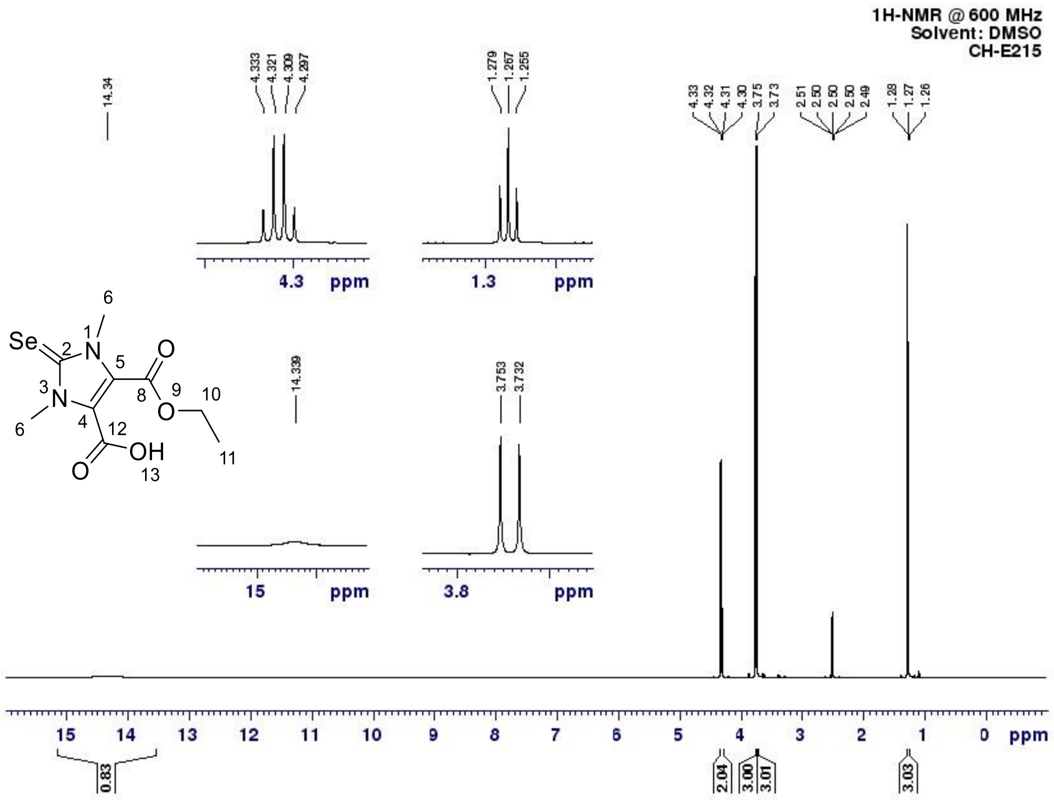

Figure S103: ^{1}H NMR spectrum of **16a** in DMSO-d_6.

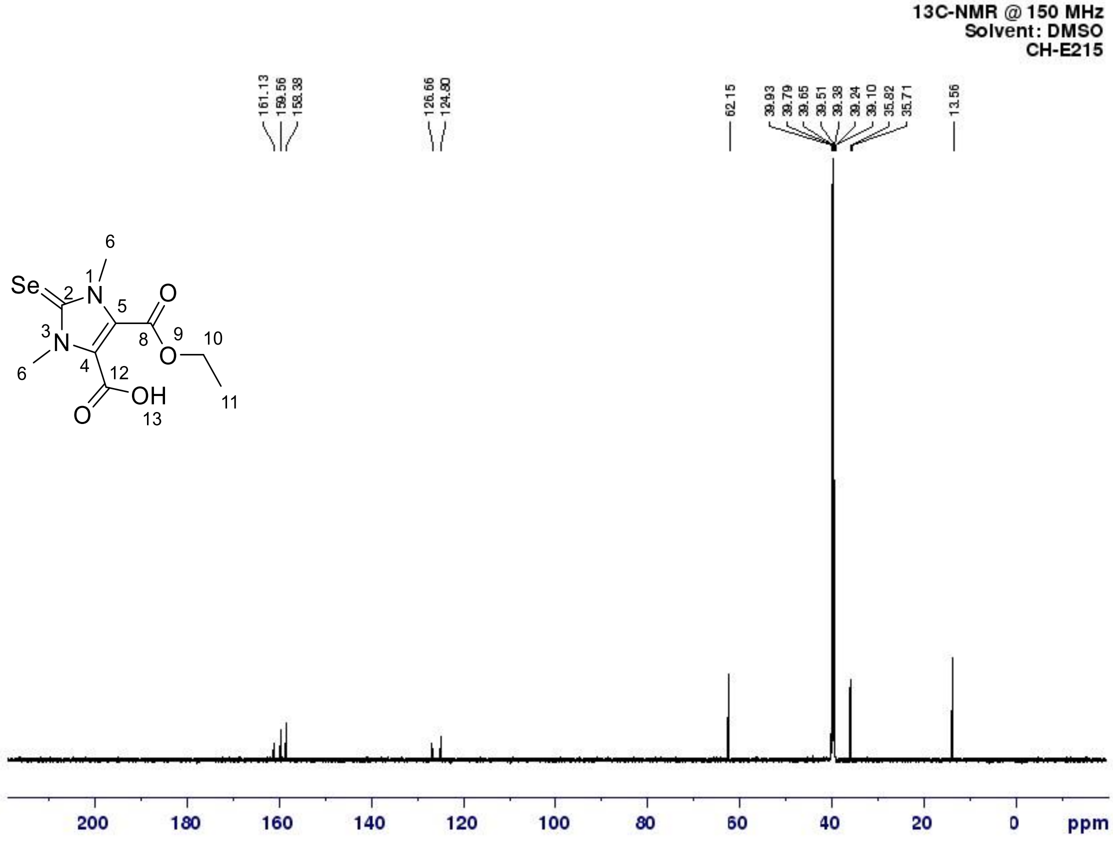

Figure S104: $^{13}C\{^{1}H\}$ NMR spectrum of **16a** in DMSO-d_6.

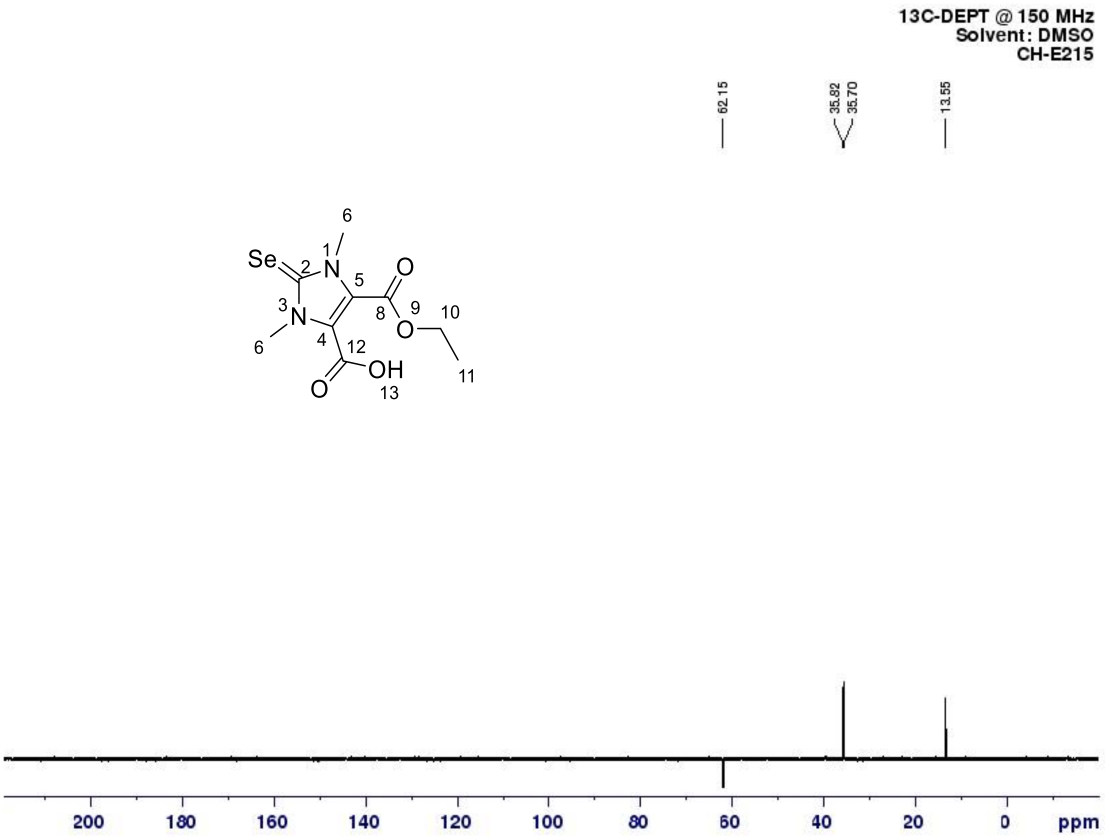

Figure S105: $^{13}C\{^{1}H\}$ DEPT NMR spectrum of **16a** in DMSO-d$_6$.

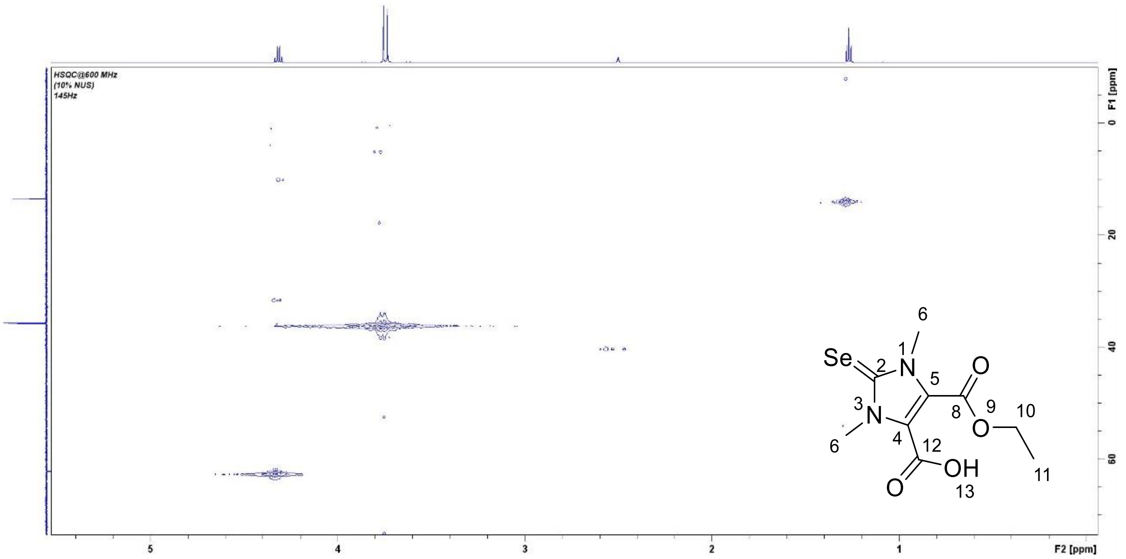

Figure S106: HSQC NMR spectrum of **16a** in DMSO-d$_6$.

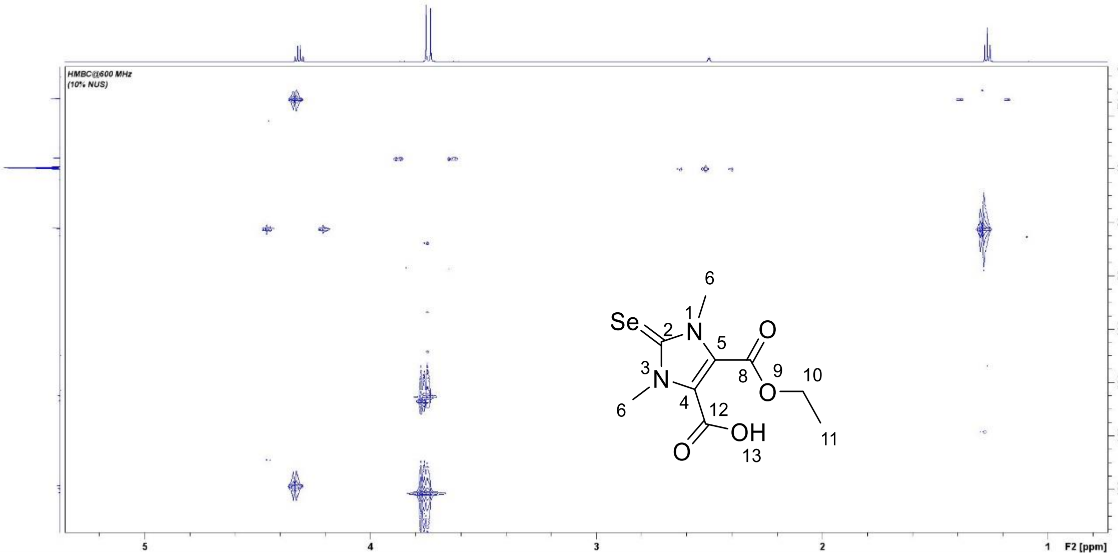

Figure S107: HMBC NMR spectrum of **16a** in DMSO-d$_6$.

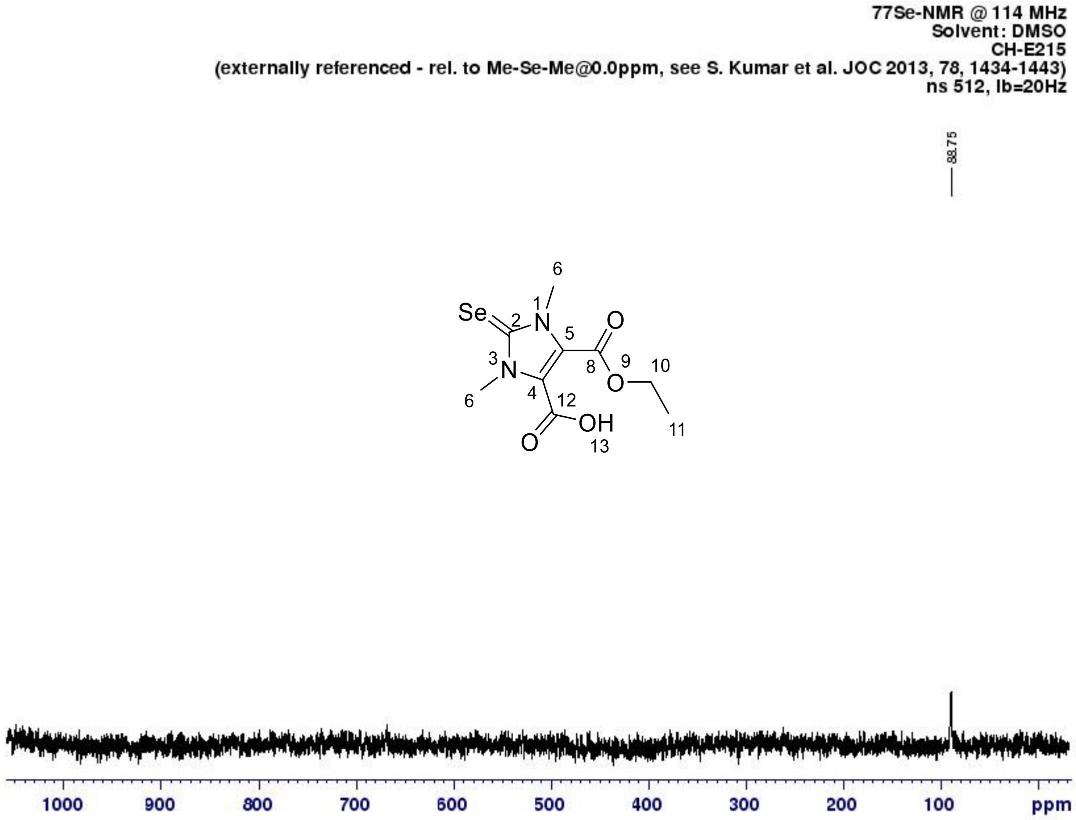

Figure S108: ^{77}Se NMR spectrum of **16a** in DMSO-d$_6$.

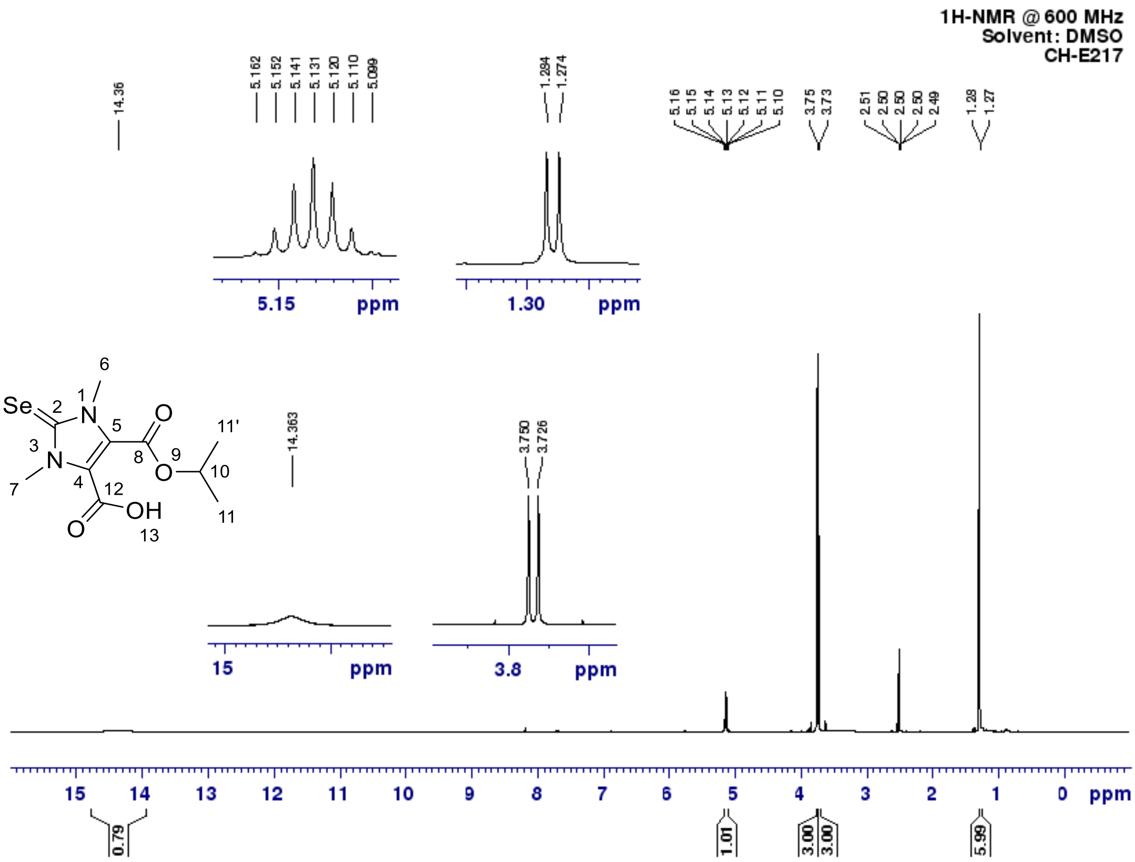

Figure S109: ^{1}H NMR spectrum of **16b** in DMSO-d$_6$.

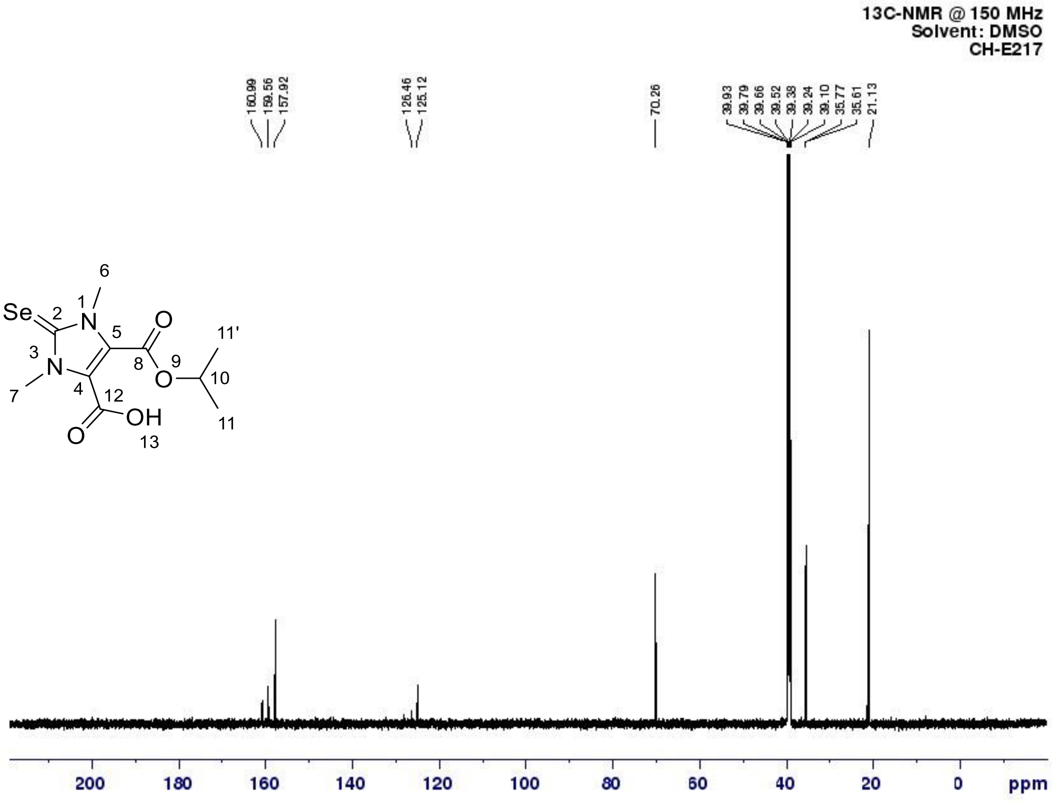

Figure S110: ^{13}C{^{1}H} NMR spectrum of **16b** in DMSO-d$_6$.

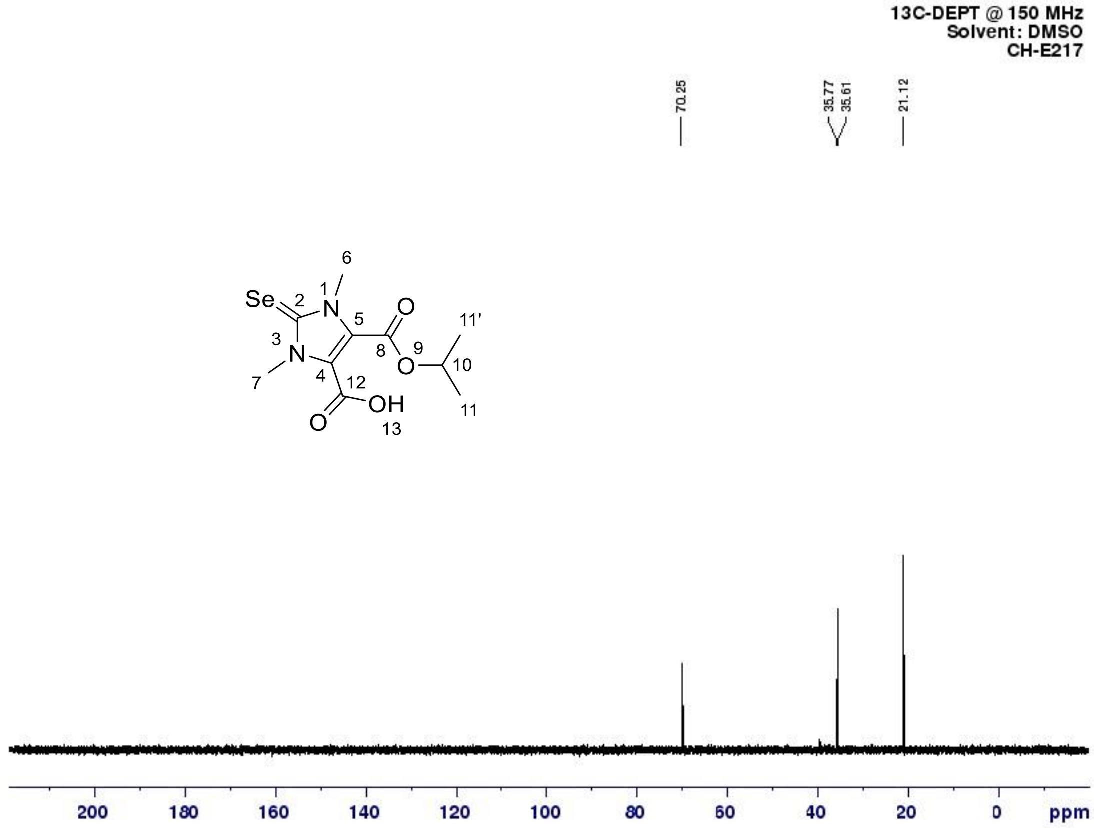

Figure S111: $^{13}C\{^{1}H\}$ DEPT NMR spectrum of **16b** in DMSO-d$_6$.

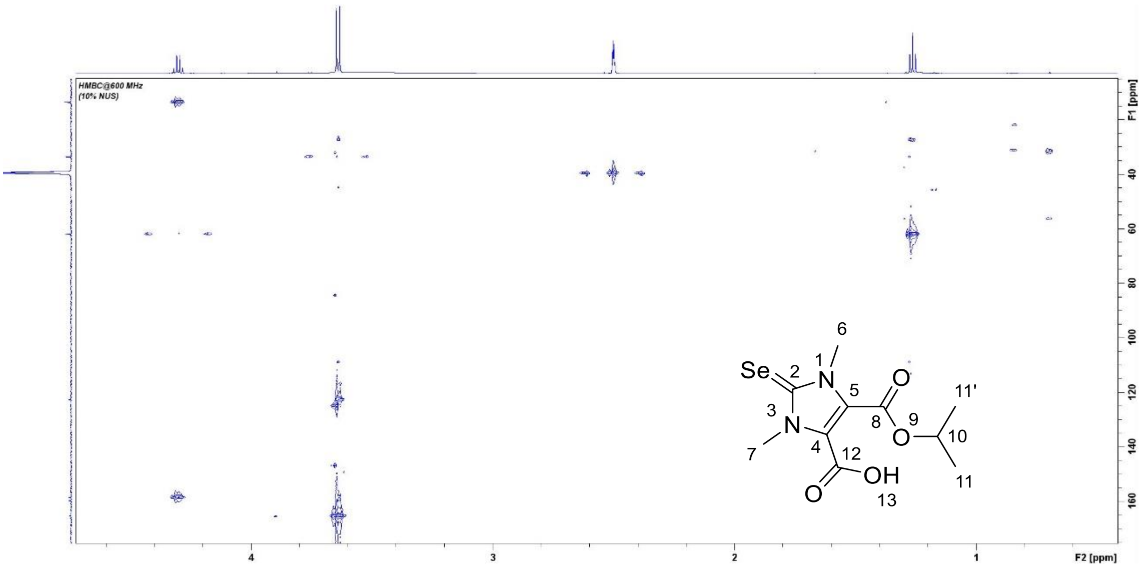

Figure S112: HSQC NMR spectrum of **16b** in DMSO-d$_6$.

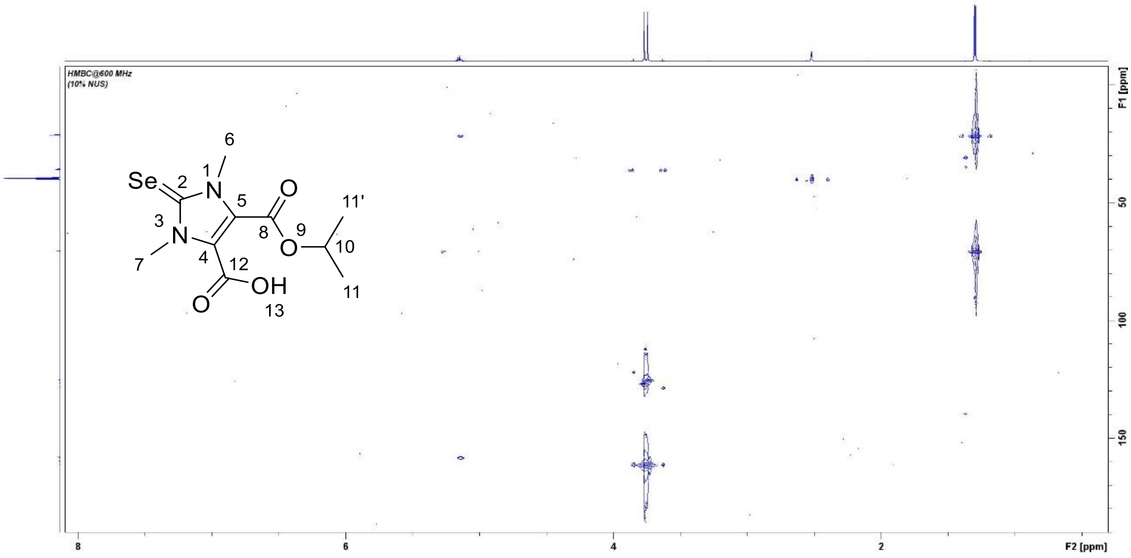

Figure S113: HMBC NMR spectrum of **16b** in DMSO-d_6.

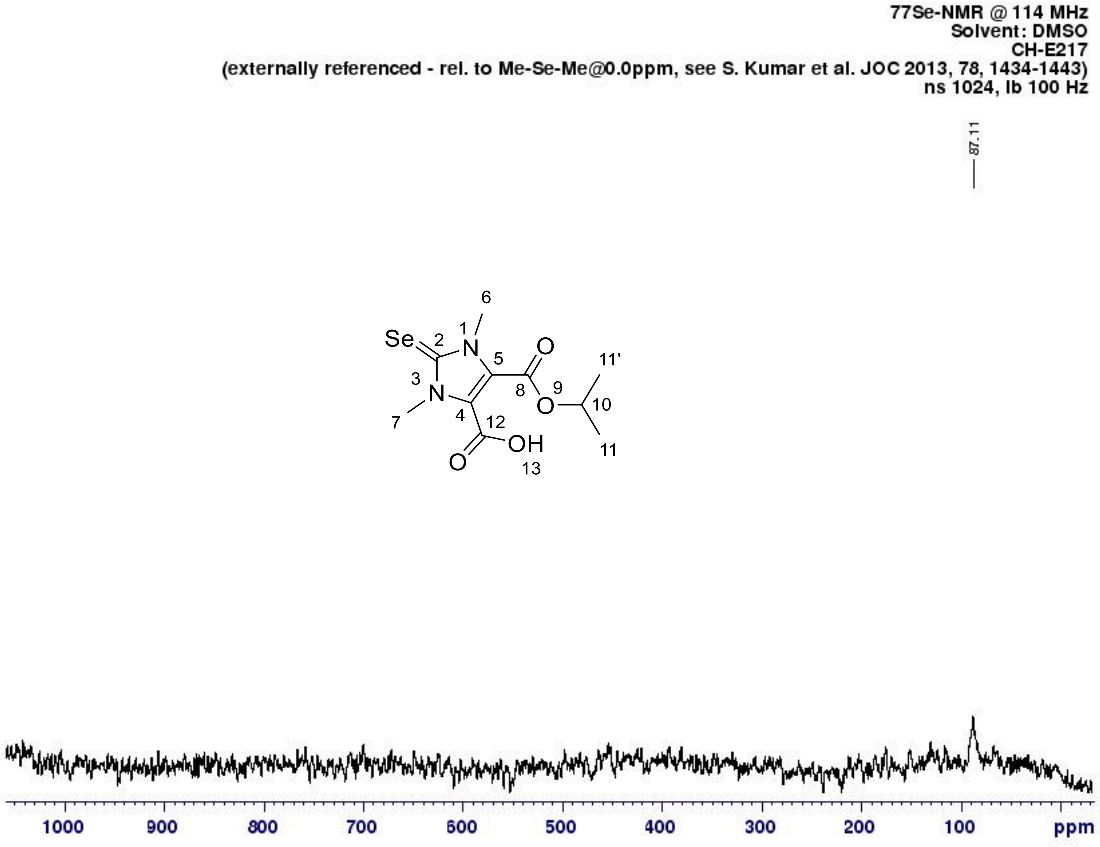

Figure S114: ^{77}Se NMR spectrum of **16b** in DMSO-d_6.

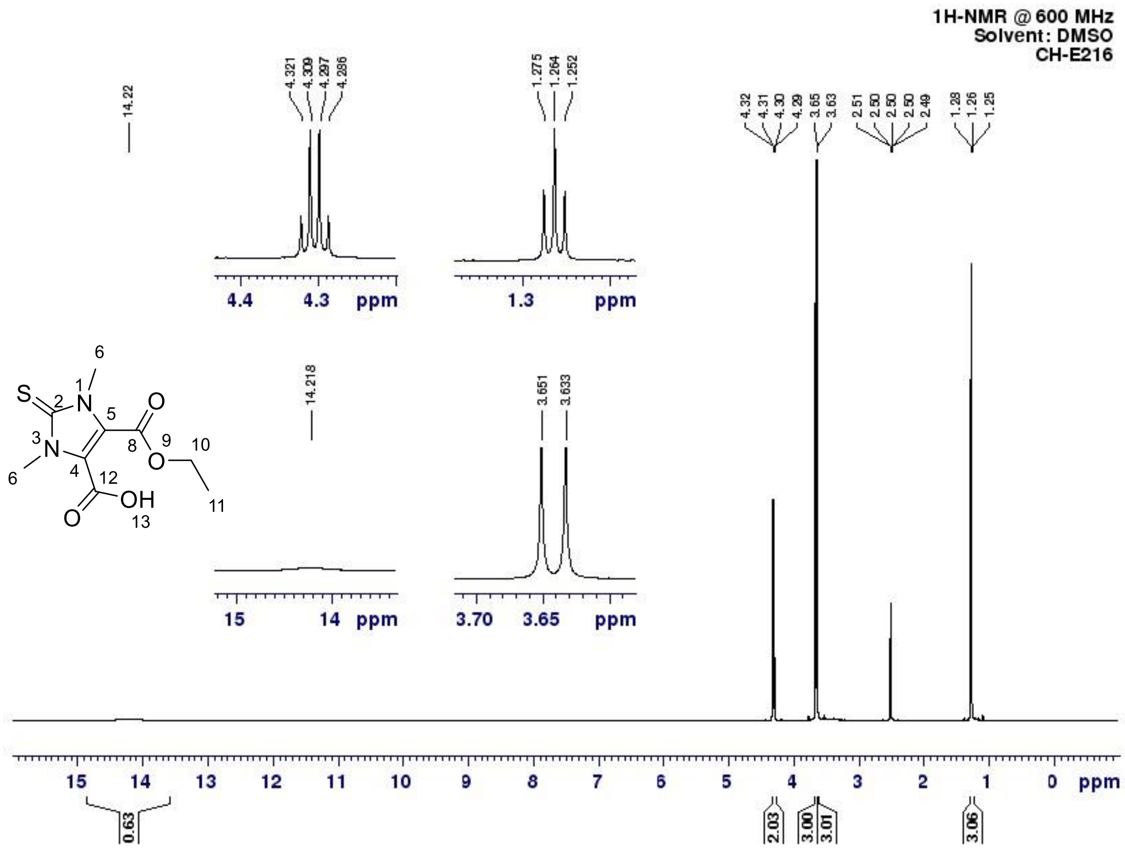

Figure S115: ^{1}H NMR spectrum of **16c** in DMSO-d$_6$.

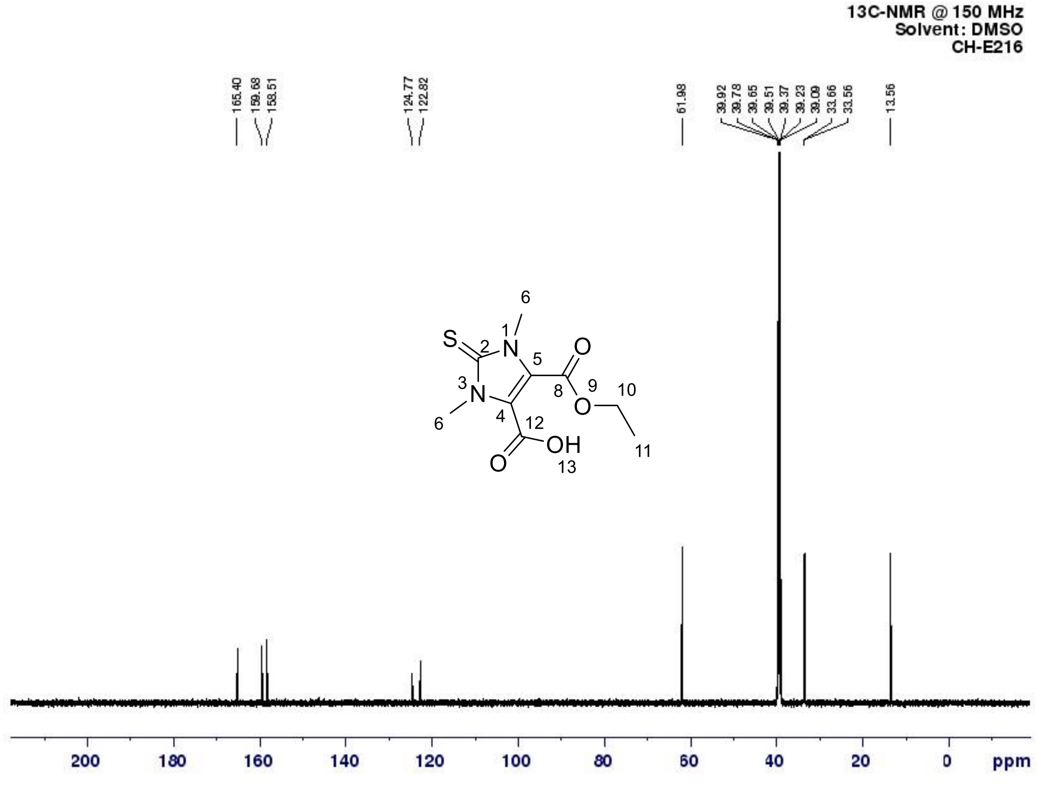

Figure S116: $^{13}C\{^{1}H\}$ NMR spectrum of **16c** in DMSO-d$_6$.

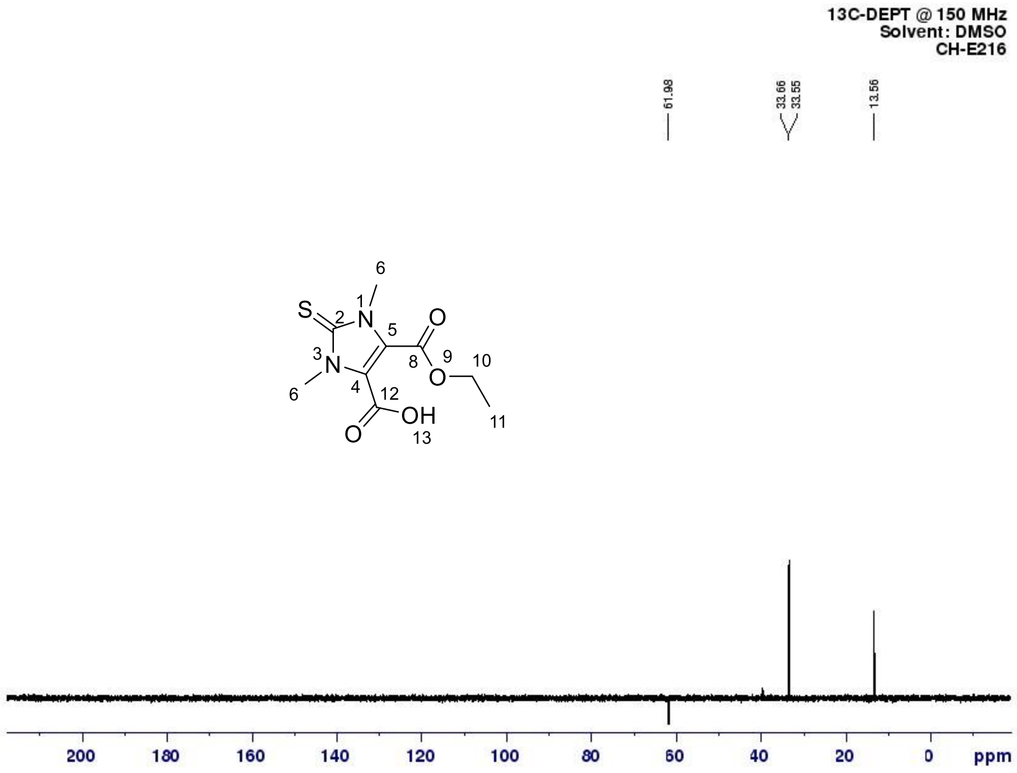

Figure S117: $^{13}C\{^{1}H\}$ DEPT NMR spectrum of **16c** in DMSO-d$_6$.

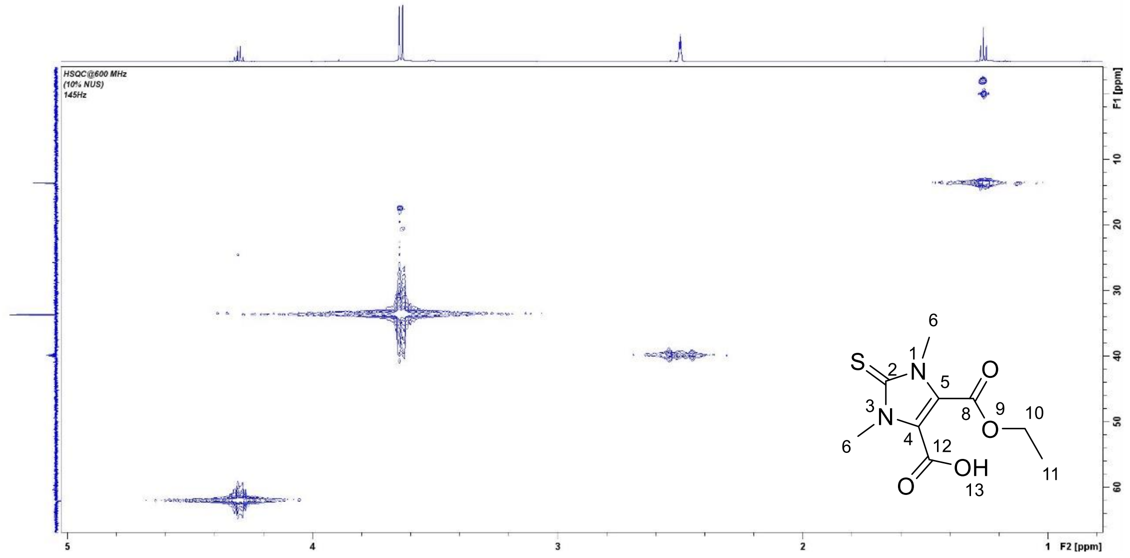

Figure S118: HSQC NMR spectrum of **16c** in DMSO-d$_6$.

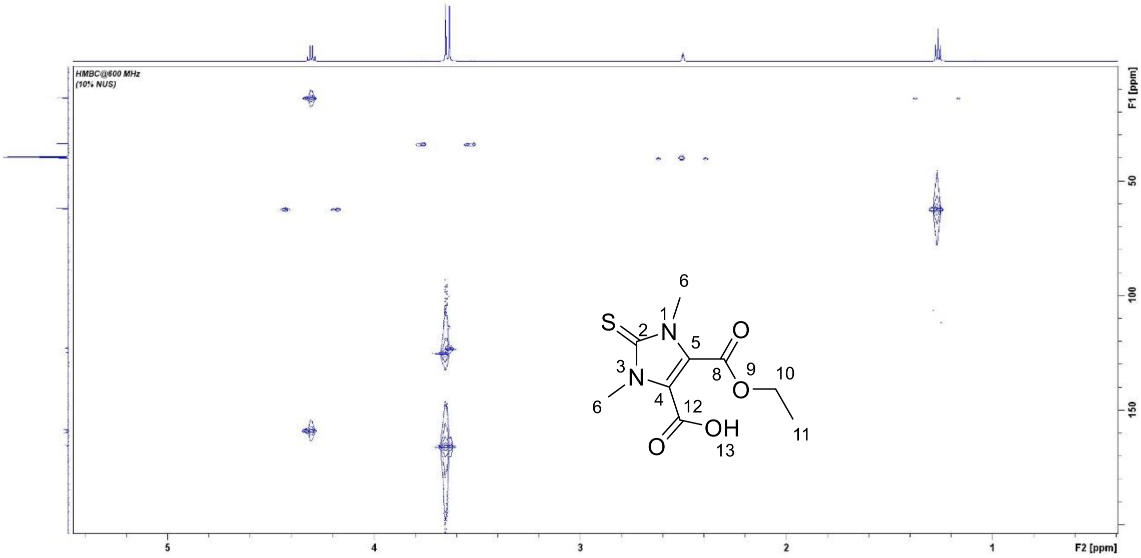

Figure S119: HMBC NMR spectrum of **16c** in DMSO-d6.

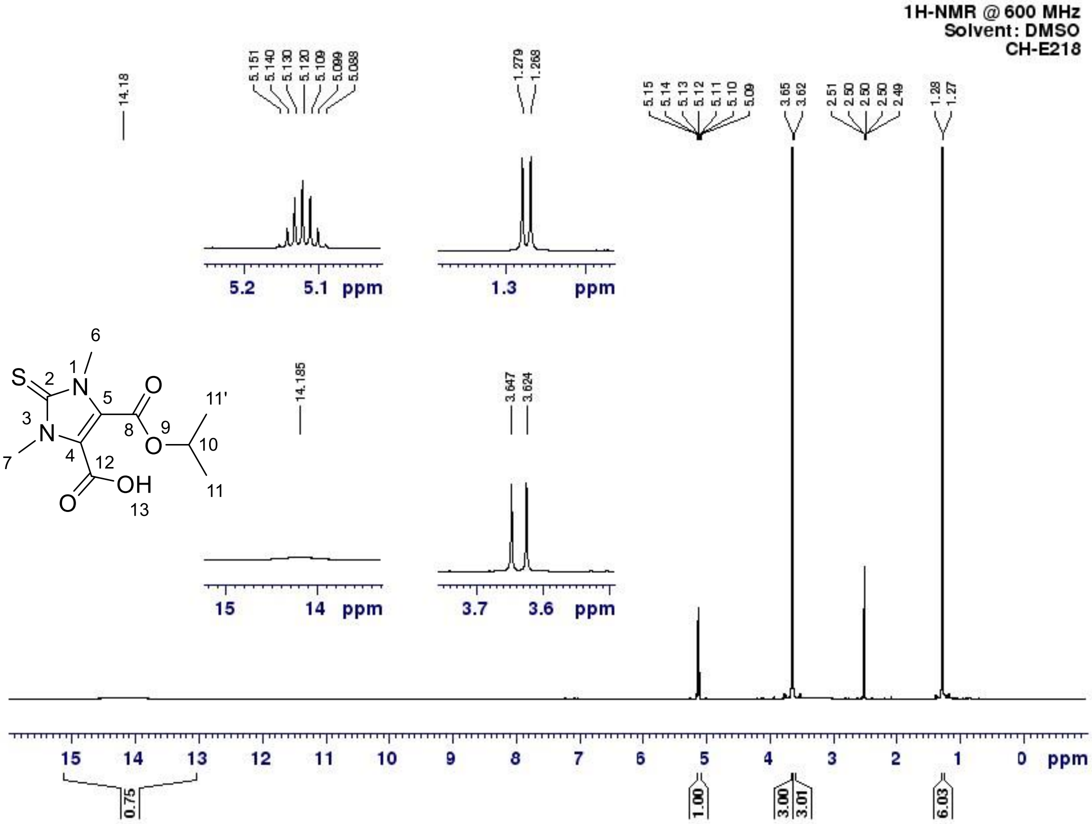

Figure S120: ^{1}H NMR spectrum of **16d** in DMSO-d6.

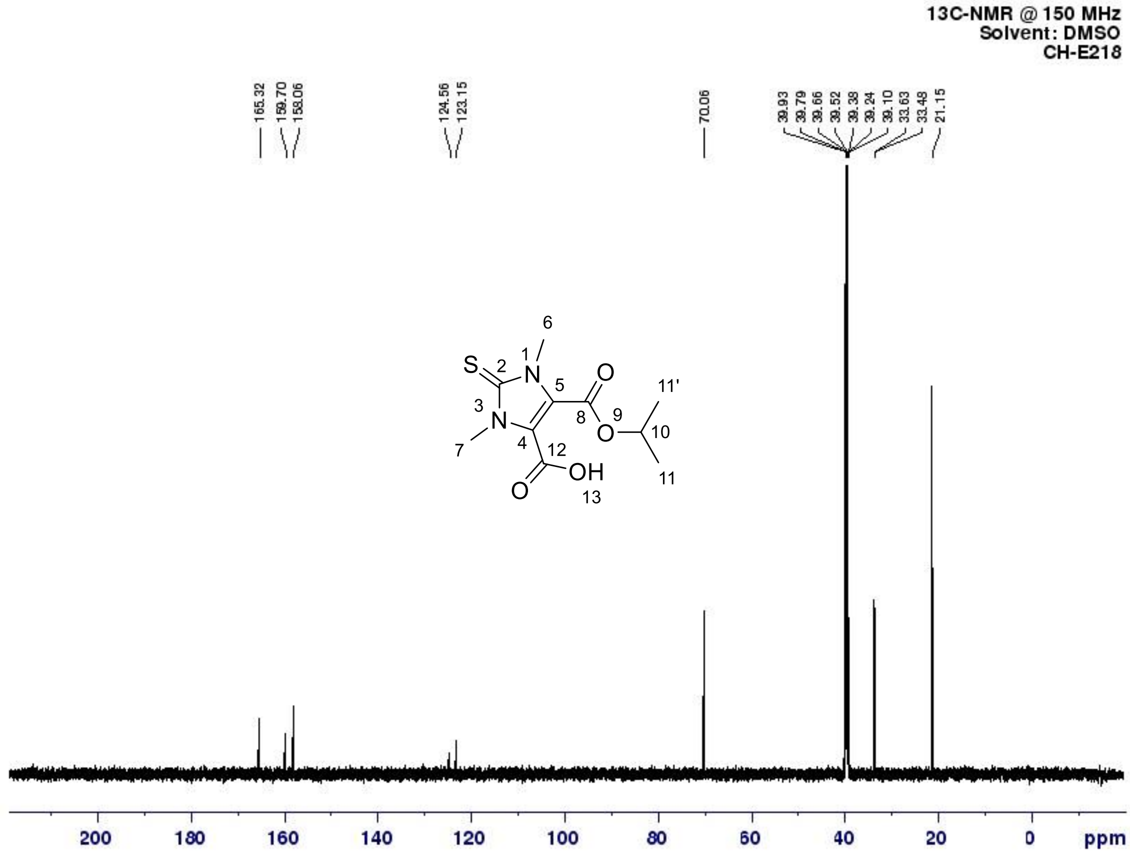

Figure S121: $^{13}C\{^{1}H\}$ NMR spectrum of **16d** in DMSO-d$_6$.

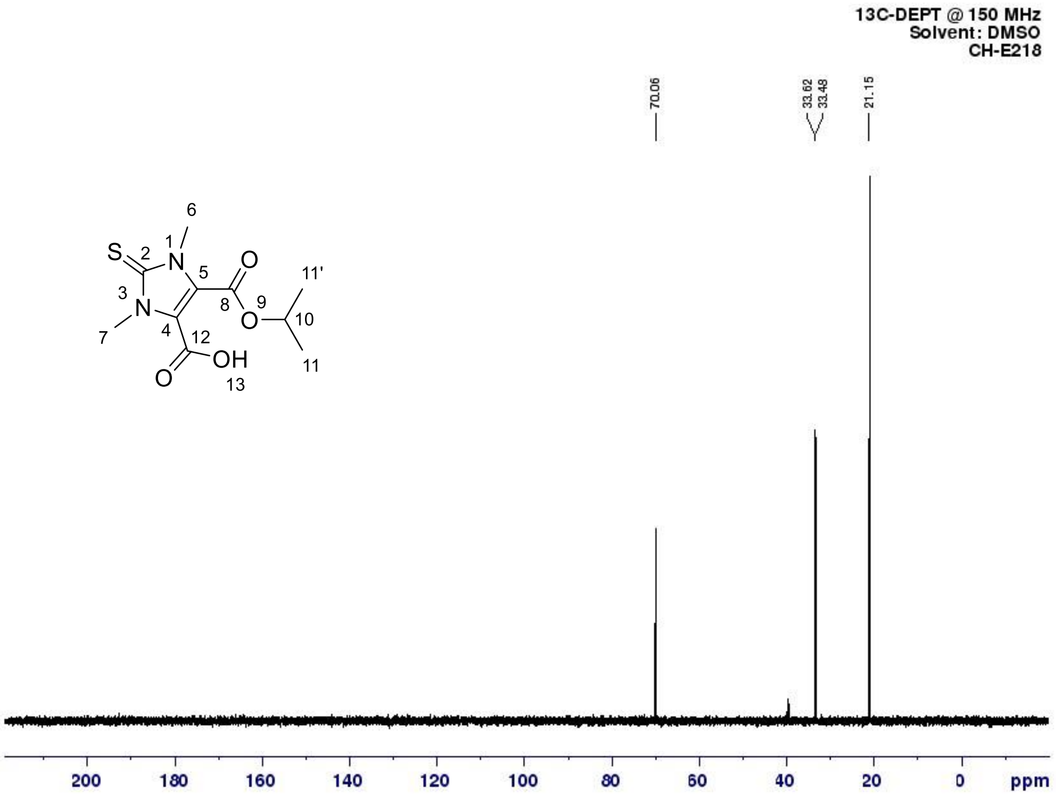

Figure S122: $^{13}C\{^{1}H\}$ DEPT NMR spectrum of **16d** in DMSO-d$_6$.

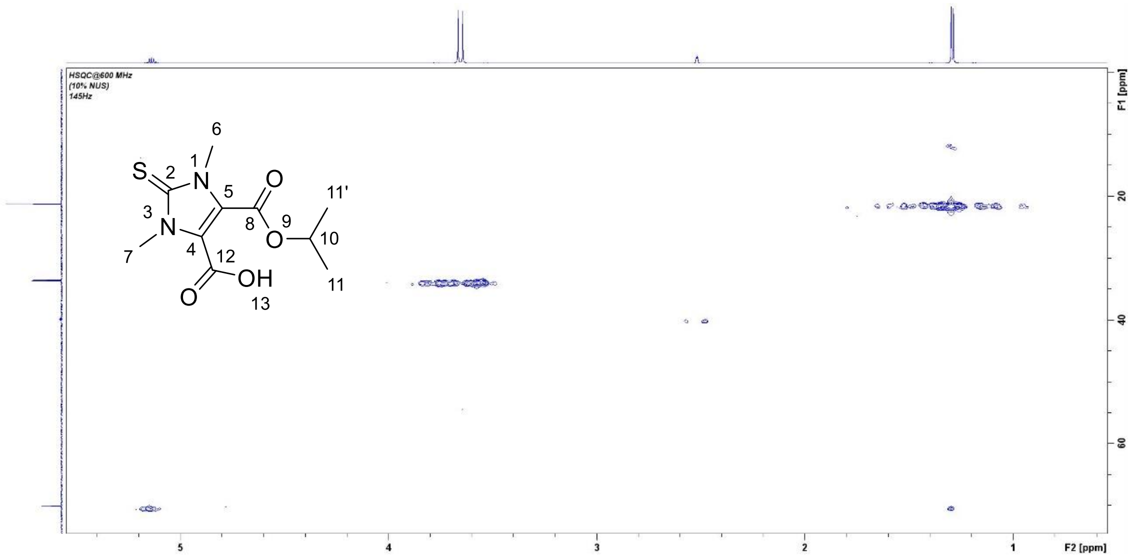

Figure S123: HSQC NMR spectrum of **16d** in DMSO-d_6.

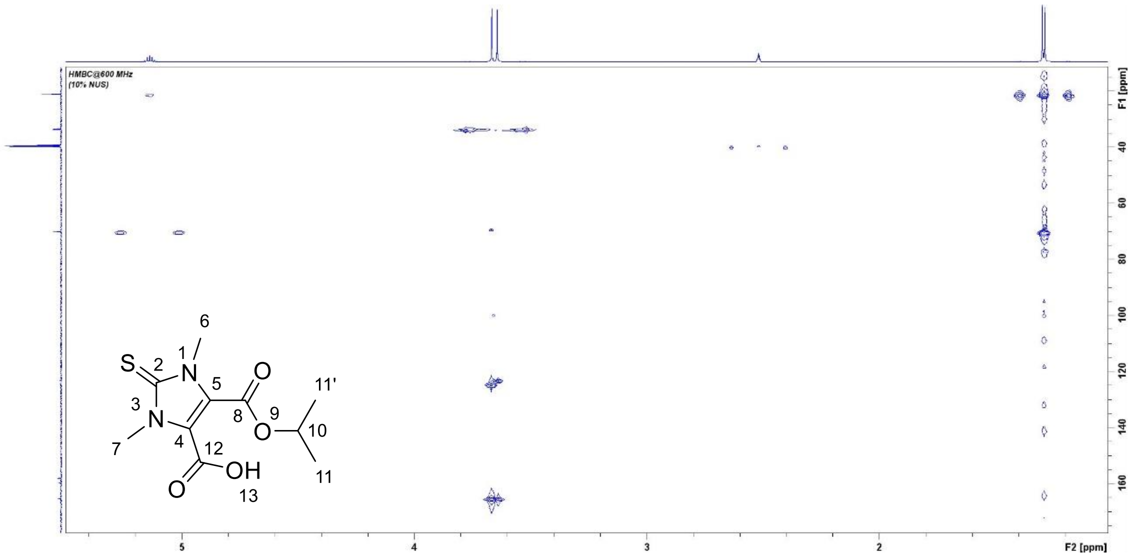

Figure S124: HMBC NMR spectrum of **16d** in DMSO-d_6.

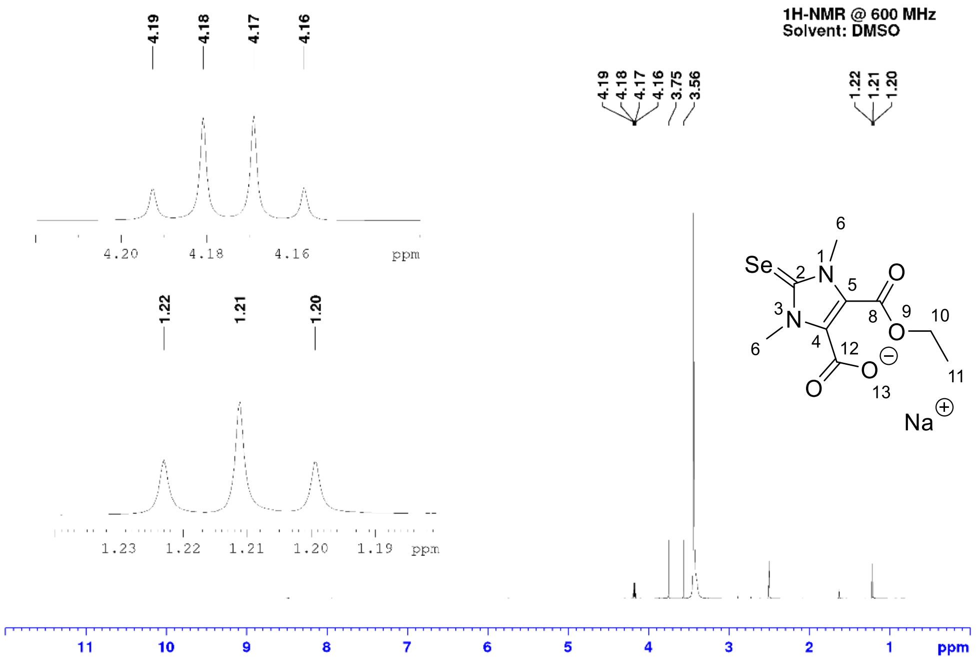

Figure S125: ^{1}H NMR spectrum of **17a** in DMSO-d_6.

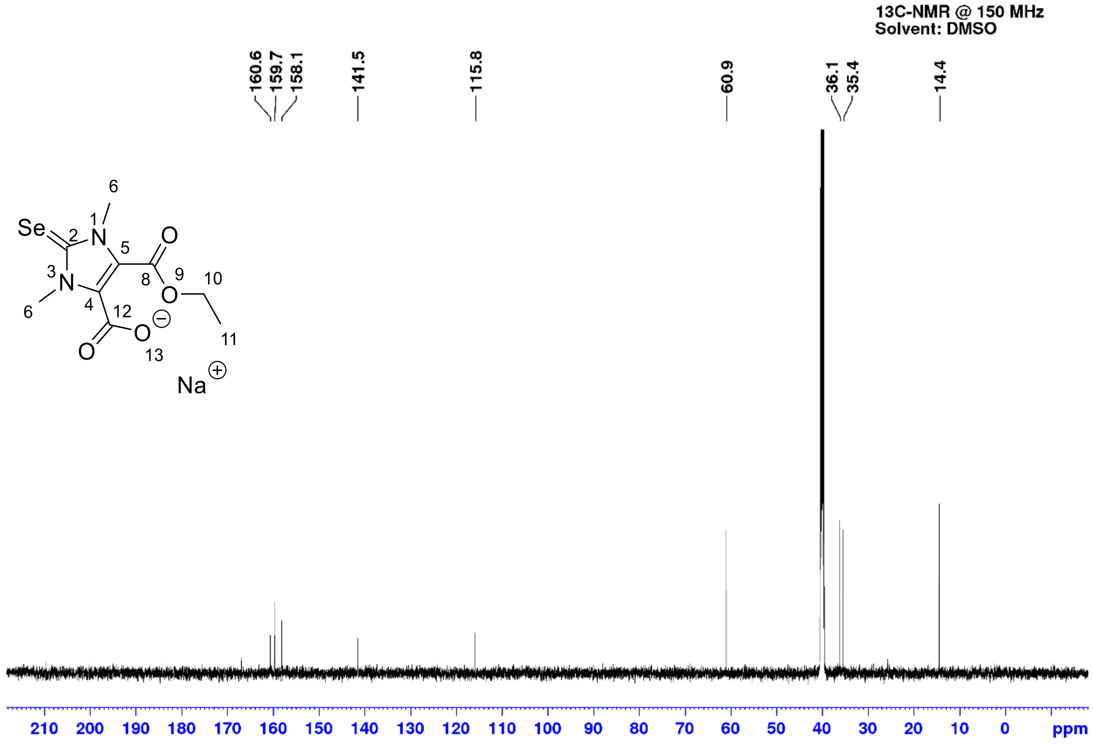

Figure S126: $^{13}C\{^{1}H\}$ NMR spectrum of **17a** in DMSO-d_6.

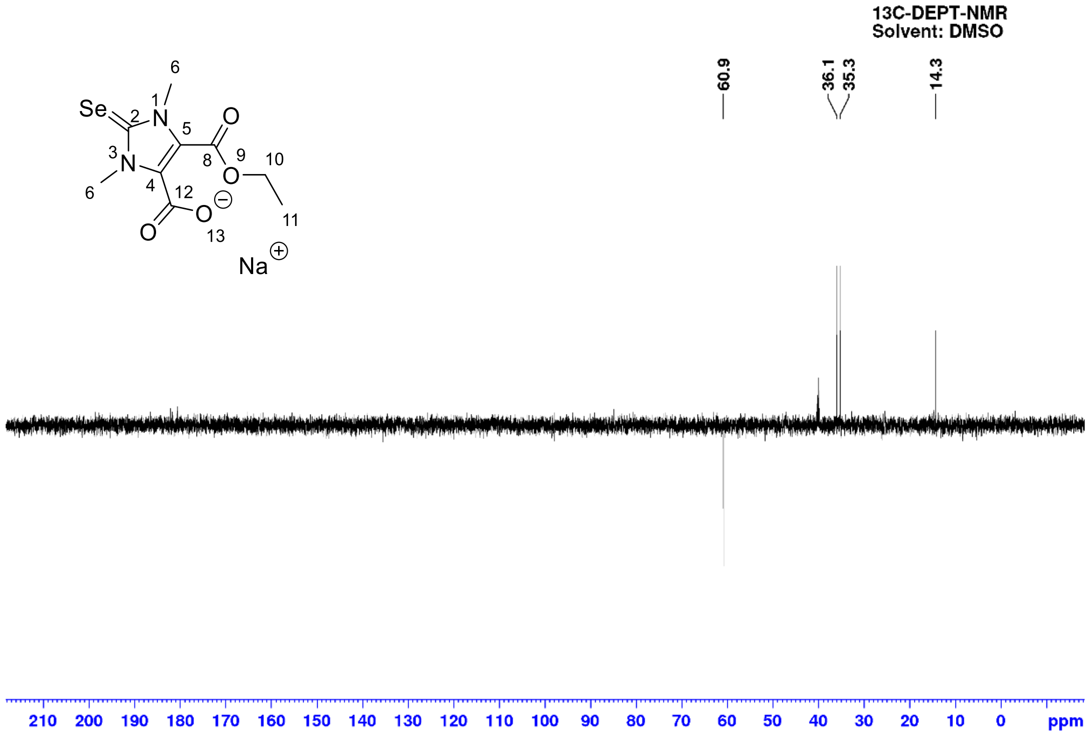

Figure S127: $^{13}C\{^{1}H\}$ DEPT-NMR spectrum of **17a** in DMSO-d$_6$.

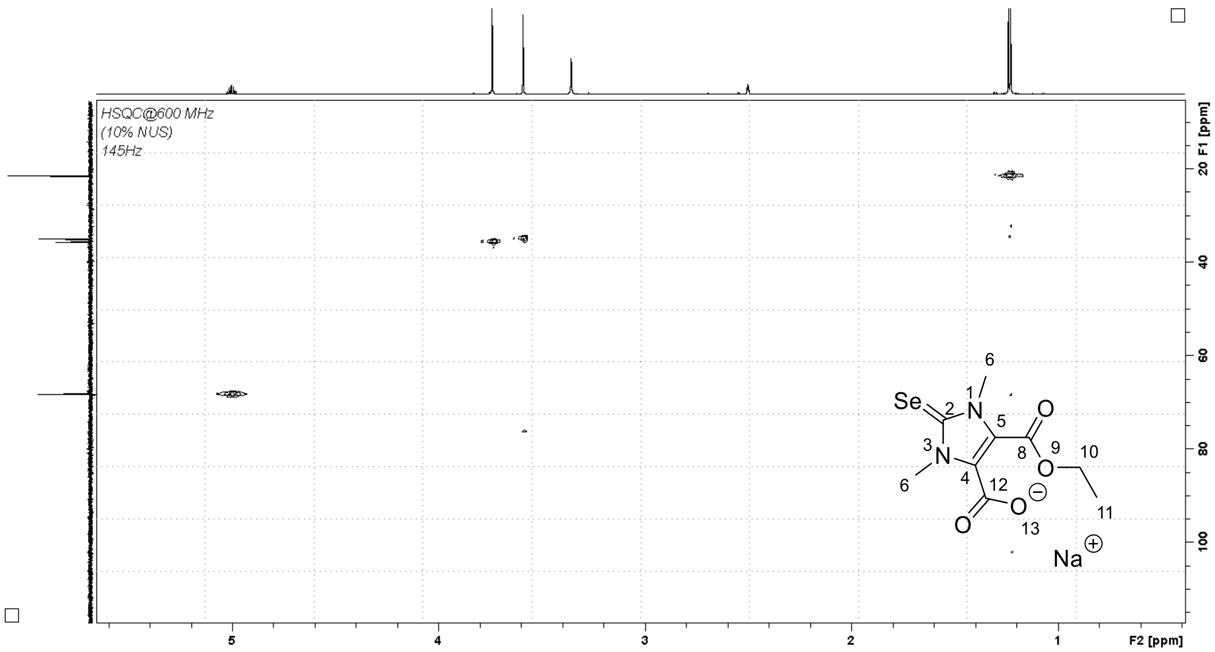

Figure S128: HSQC NMR spectrum of **17a** in DMSO-d$_6$.

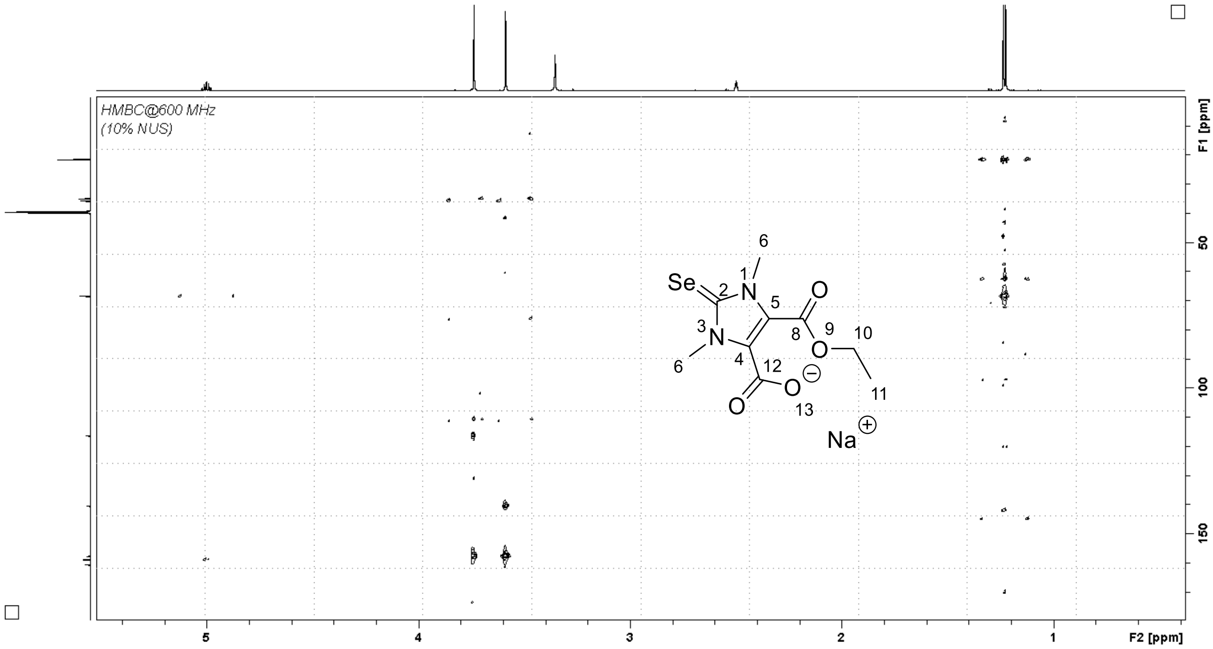

Figure S129: HMBC NMR spectrum of **17a** in DMSO-d6.

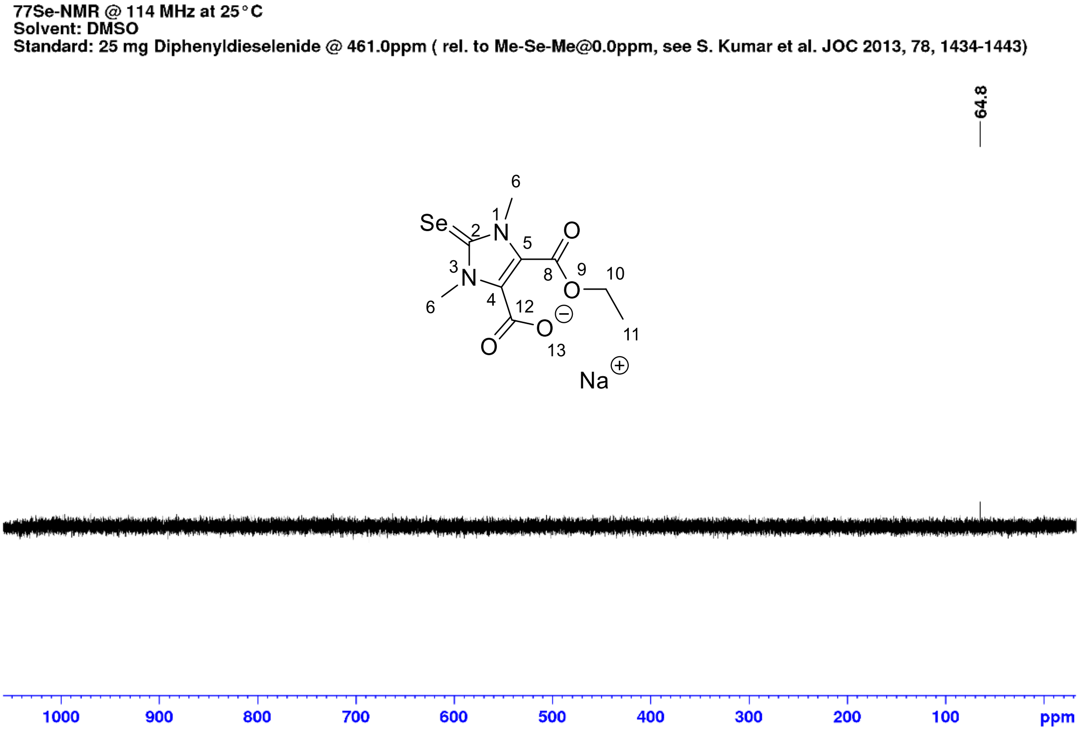

Figure S130: ^{77}Se NMR spectrum of **17a** in DMSO-d6.

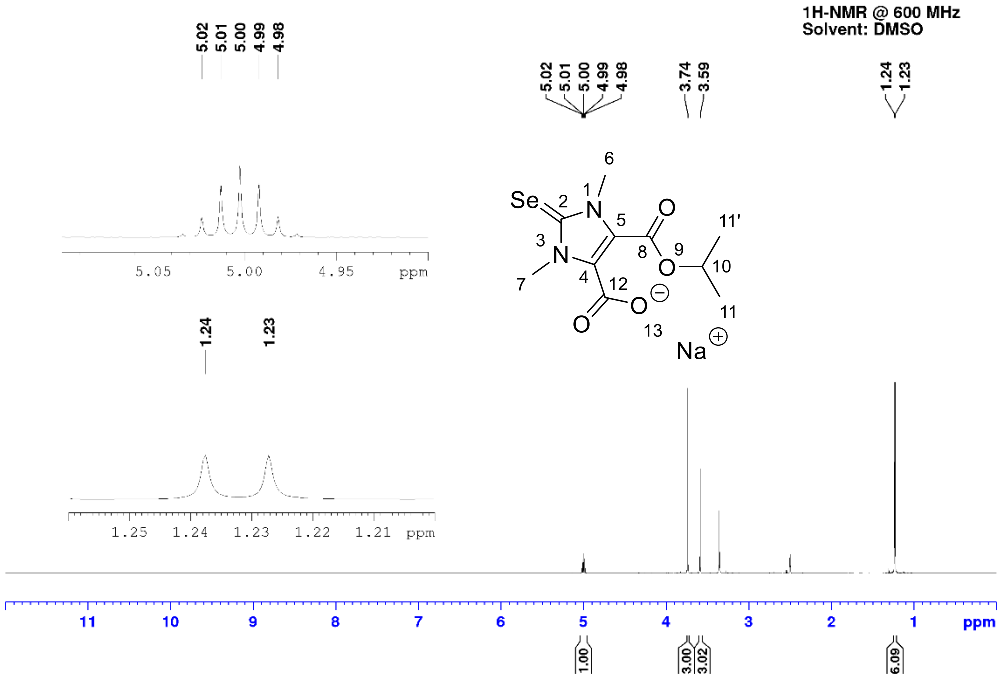

Figure S131: ^{1}H NMR spectrum of **17b** in DMSO-d$_6$.

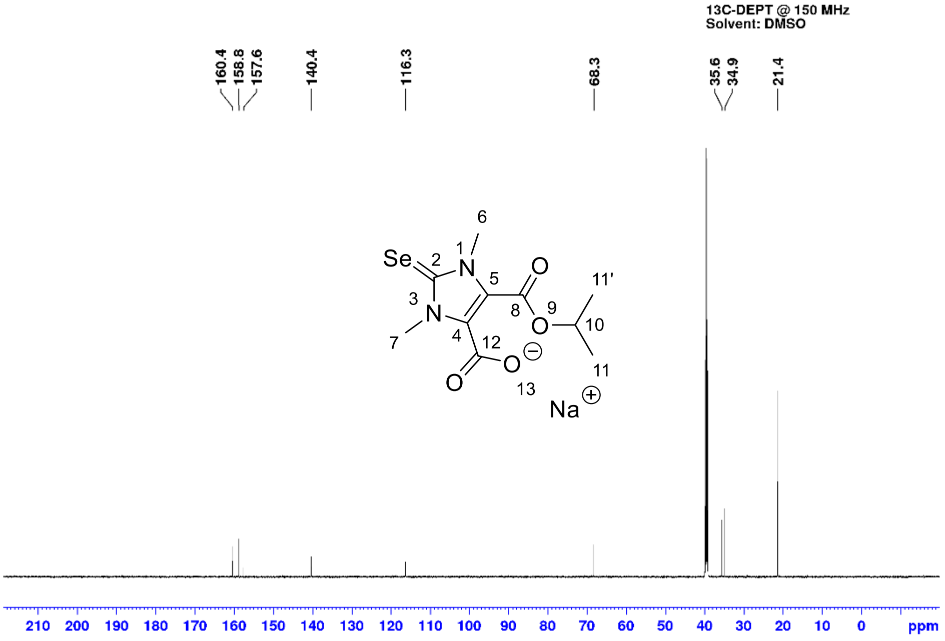

Figure S132: ^{13}C{^{1}H} NMR spectrum of **17b** in DMSO-d$_6$.

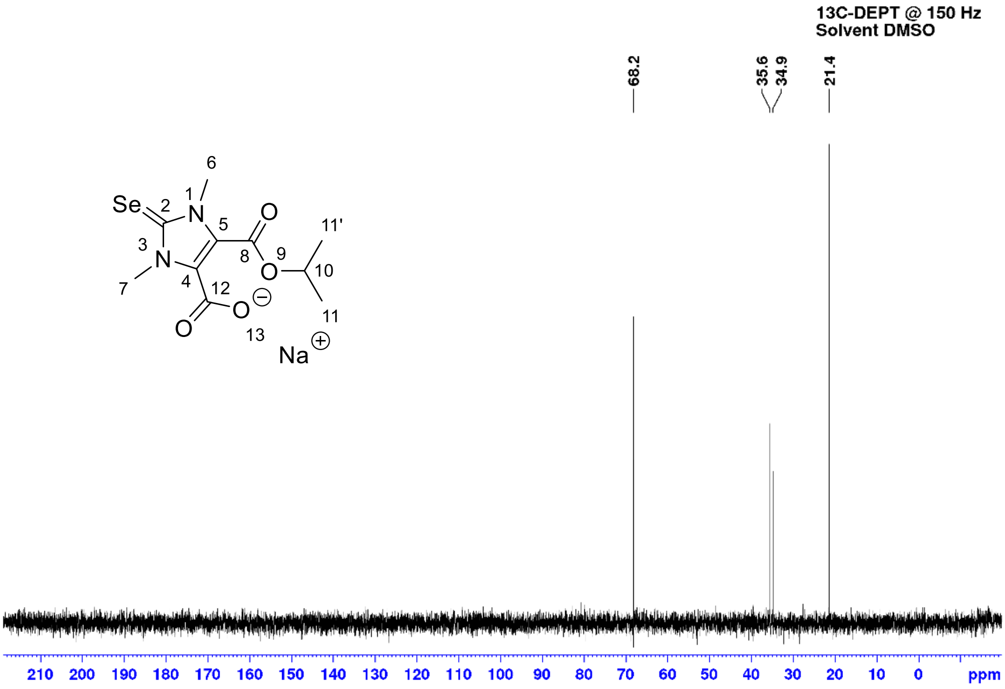

Figure S133: $^{13}C\{^{1}H\}$ DEPT NMR spectrum of **17b** in DMSO-d_6.

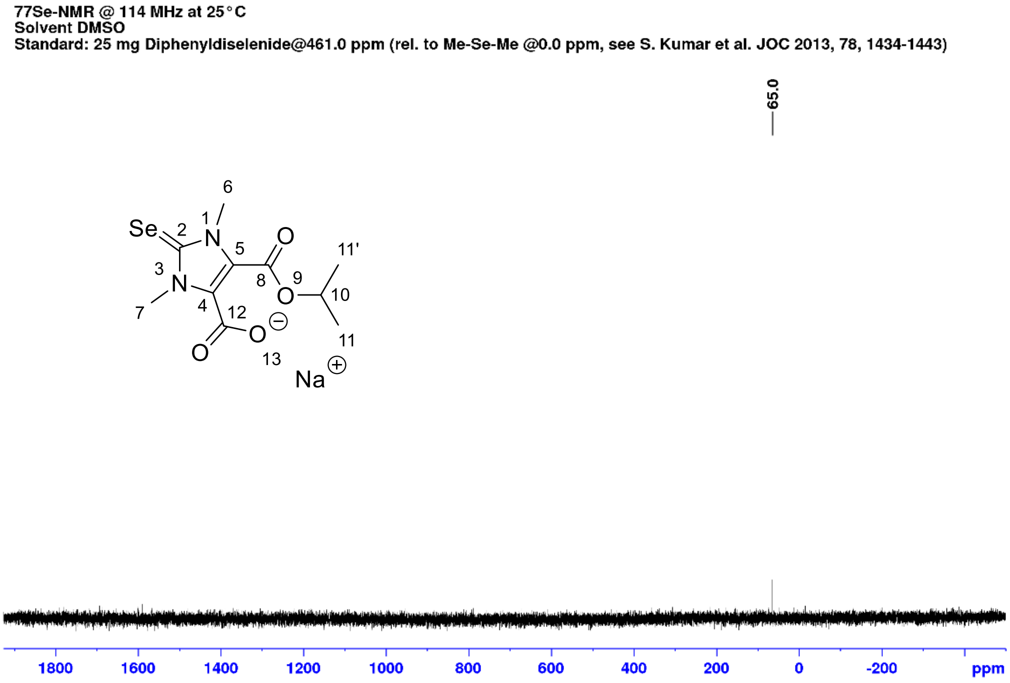

Figure S134: ^{77}Se NMR spectrum of **17b** in DMSO-d_6.

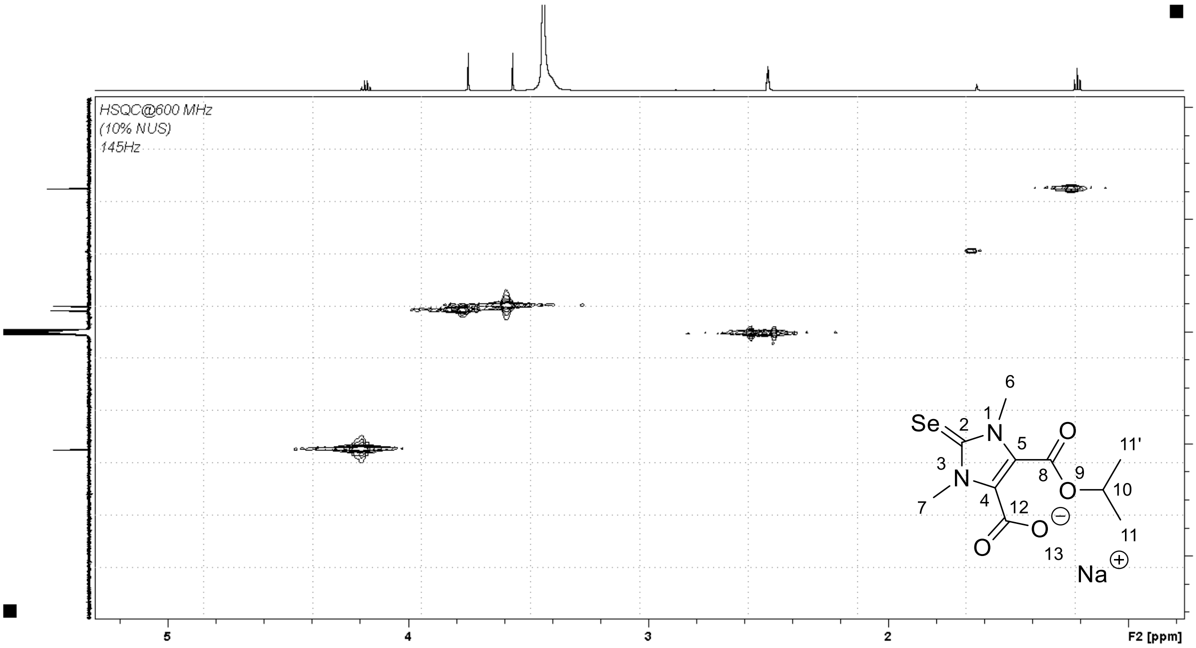

Figure S135: HSQC NMR spectrum of **17b** in DMSO-d_6.

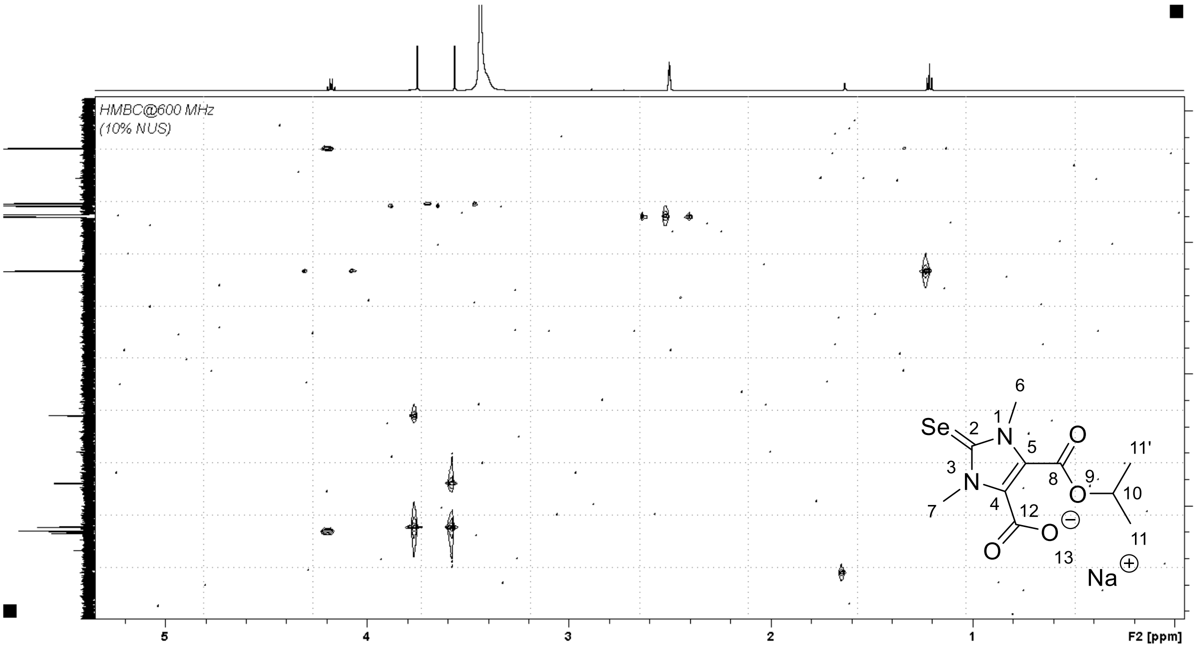

Figure S136: HMBC NMR spectrum of **17b** in DMSO-d_6.

DFT calculations (HOMO/LUMO coefficients and orbital energies)

HOMO-LUMO DFT calculations were performed using the Spartan Software (***Spartan'20***, Wavefunction, Inc., Irvine, CA. Available from: http://www.wavefun.com) running on a MS Windows 10 Pro PC system with an AMD Ryzen Threadripper 3970X 32-Core and 128 GB. MMFF optimized structures were used as starting geometries for the geometry optimizations with the B3LYP density functional and the 6-311++G(d,p) basis set carried out as vacuum calculations. Subsequent frequency calculations of all final structures evidenced the absence of imaginary frequencies and thus the presence of true minima on the potential energy surface.

Calculation of 12a

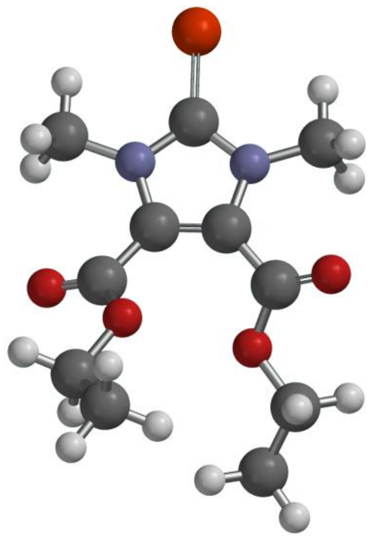

Charge: 0

Number of imaginary frequencies: 0

E: -3241.0248279995 hartrees

Coordinates (Angstroem)

ATOM	X	Y	Z
C	-0.0000000054	0.0000000341	-3.3576384327
N	-1.0806544218	-0.1696221913	-2.5235025982
C	-0.6776262622	-0.0999850620	-1.1993637378
C	0.6776263073	0.0999850276	-1.1993637454
N	1.0806544394	0.1696222286	-2.5235026175
Se	0.0000000413	0.0000000074	-5.1978439139
C	2.4574457498	0.3285873867	-2.9936729988
C	-2.4574457484	-0.3285873374	-2.9936729227
C	-1.6411961001	-0.2267449930	-0.0815771529
C	1.6411961529	0.2267449480	-0.0815771809
O	2.6116812197	0.9486067830	-0.1116496018
O	1.3273411806	-0.5802402360	0.9420065447
C	2.1798583112	-0.4893933853	2.1156217287
C	1.7280327524	-1.5517147840	3.0955515353
O	-2.6116811491	-0.9486068547	-0.1116495657

O	-1.3273411407	0.5802402034	0.9420065721
C	-2.1798582991	0.4893933589	2.1156217414
C	-1.7280328268	1.5517148214	3.0955515443
H	3.0792868749	-0.4783665984	-2.6036517467
H	2.4280829366	0.2825167682	-4.0802751935
H	2.8566405014	1.2844420845	-2.6601208589
H	-2.8566404137	-1.2844421069	-2.6601208804
H	-2.4280829723	-0.2825167313	-4.0802751095
H	-3.0792869058	0.4783665565	-2.6036515318
H	3.2157346423	-0.6327572732	1.8027624479
H	2.0905488133	0.5193309660	2.5262504160
H	1.8223758047	-2.5500164971	2.6630947565
H	0.6882180713	-1.4009538333	3.3935420448
H	2.3508509596	-1.5064604567	3.9927253700
H	-2.0905487711	-0.5193309744	2.5262504706
H	-3.2157346222	0.6327571966	1.8027624235
H	-0.6882181414	1.4009539336	3.3935420941
H	-1.8223759140	2.5500165104	2.6630947384
H	-2.3508510613	1.5064604896	3.9927253604

LUMO+2 (-0.65 eV)

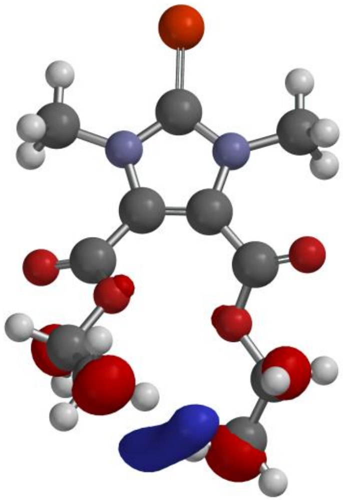

LUMO+1 (-0.73 eV)

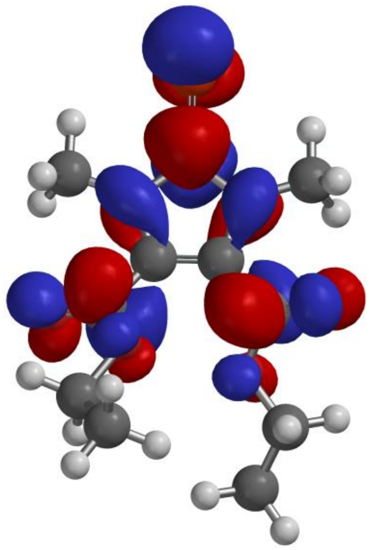

LUMO (-2.18 eV)

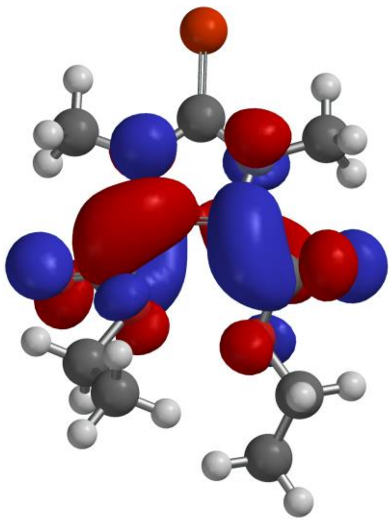

HOMO (-5.37 eV)

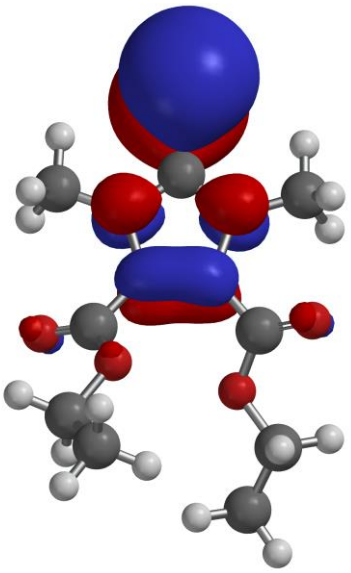

HOMO-1 (-5.50 eV)

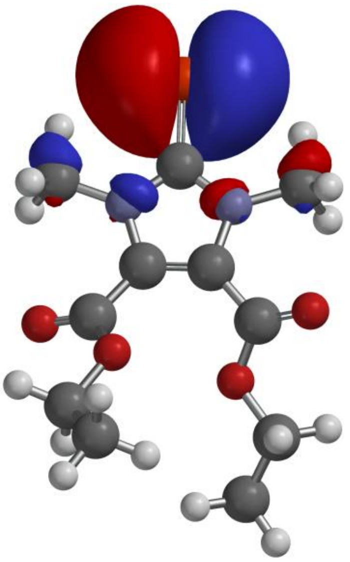

HOMO-2 (-7.94 eV)

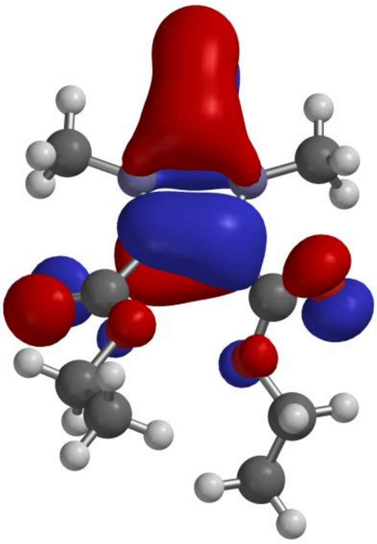

HOMO-3 (-8.44 eV)

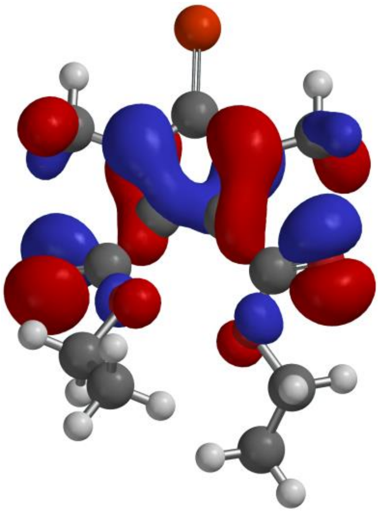

HOMO-4 (-8.60 eV)

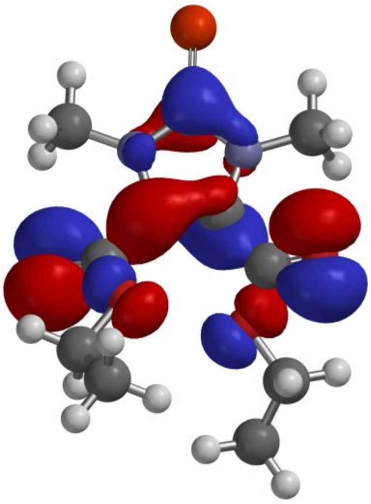

Calculation of 12b

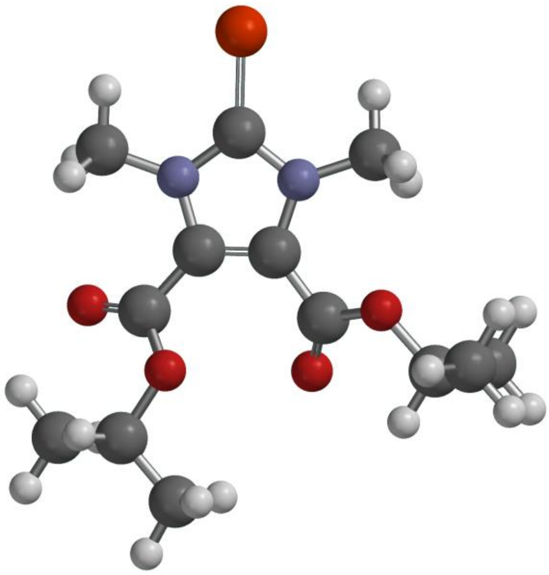

Charge: 0

Number of imaginary frequencies: 0

E: -3319.6812800500 hartrees

Coordinates (Angstroem)

ATOM	X	Y	Z
C	0.5648197382	1.0337890039	-3.1410013789
N	0.4172693193	-0.1710757161	-2.4914078532
C	-0.0717846628	0.0336572504	-1.2089935980
C	-0.2359054855	1.3850194326	-1.0534616981
N	0.1523999118	1.9855353997	-2.2401184743
Se	1.1894807957	1.3053931647	-4.8500504144
C	0.1770007065	3.4243529589	-2.5057738453
C	0.6604736863	-1.4616168244	-3.1335917477
C	-0.3992872654	-1.0416924714	-0.2384141774
C	-0.7341846371	2.1722038677	0.1005463417
O	-1.3824142410	3.1875825676	-0.0238398482
O	-0.3621651967	1.6306504774	1.2629842345
C	-0.9212567567	2.2042386293	2.4948778980
C	-1.0071826508	1.0548132866	3.4848307870
O	-1.3549433653	-1.0127063242	0.4992237300
O	0.5038422562	-2.0363135442	-0.2875928462
C	0.3114252210	-3.1780285105	0.6178028500
C	1.6953355719	-3.7630517131	0.8439518360
C	-0.0382872298	3.3542288886	2.9591300181
C	-0.6757747219	-4.1597322815	0.0010472146
H	0.6624585351	3.5594627517	-3.4699049155
H	-0.8382076476	3.8171205238	-2.5317514619
H	0.7389226727	3.9361298785	-1.7244906313
H	1.5514436788	-1.9303863854	-2.7177007263
H	0.8033505317	-1.2657816360	-4.1944731650
H	-0.1989852819	-2.1153790360	-2.9852035780
H	-1.9170220963	2.5756180809	2.2465095349
H	-0.0100498892	0.6791135014	3.7302146317
H	-1.5978862985	0.2352734211	3.0726922209
H	-1.4802047593	1.3997581384	4.4080255743
H	-0.0951808614	-2.7780232997	1.5482835675
H	2.1242930303	-4.1260647679	-0.0936621119
H	2.3698893165	-3.0173146881	1.2690310185
H	1.6298564157	-4.6047796297	1.5381633447

H	0.9795327338	3.0047652642	3.1524809270
H	-0.0070705580	4.1485566045	2.2118519553
H	-0.4381049207	3.7751456175	3.8857745636
H	-0.2977935152	-4.5452709873	-0.9498880368
H	-1.6459446545	-3.6884043393	-0.1628114382
H	-0.8221574259	-5.0067865544	0.6767096980

LUMO+2 (-0.55 eV)

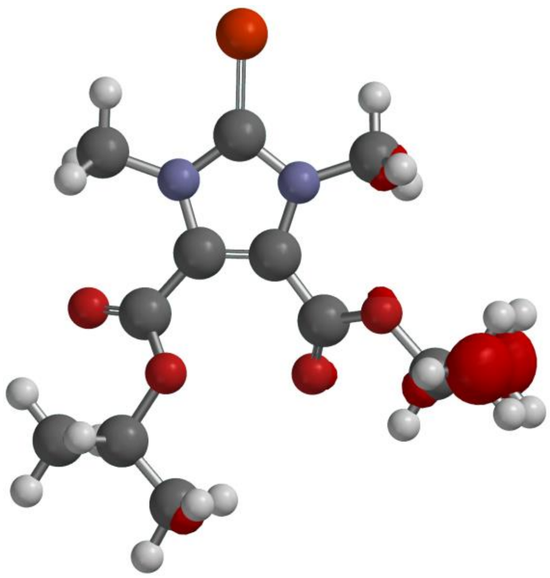

LUMO+1 (-0.66 eV)

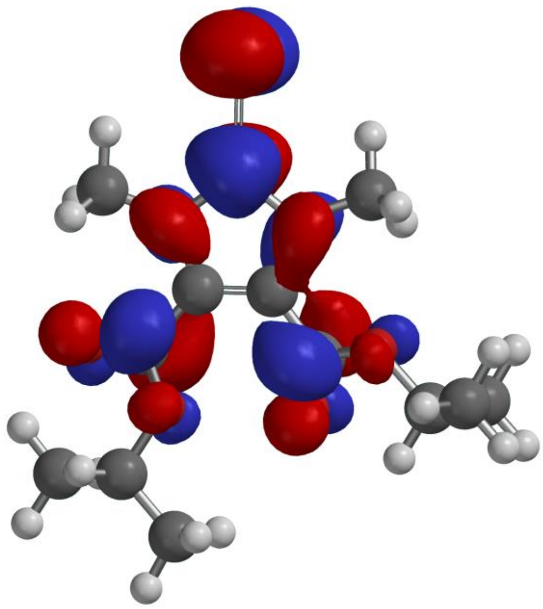

LUMO (-2.06 eV)

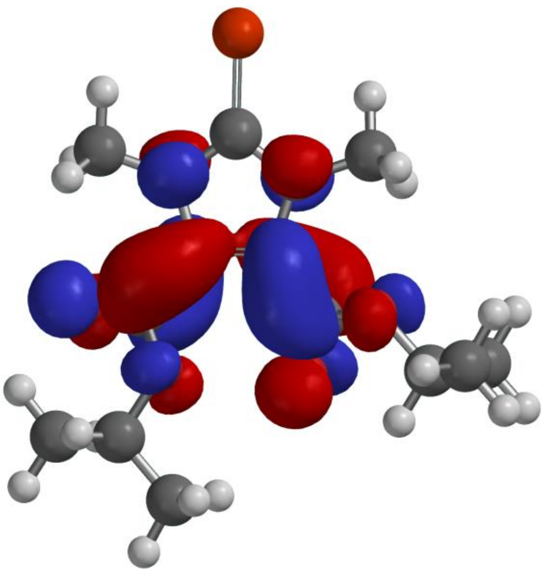

HOMO (-5.33 eV)

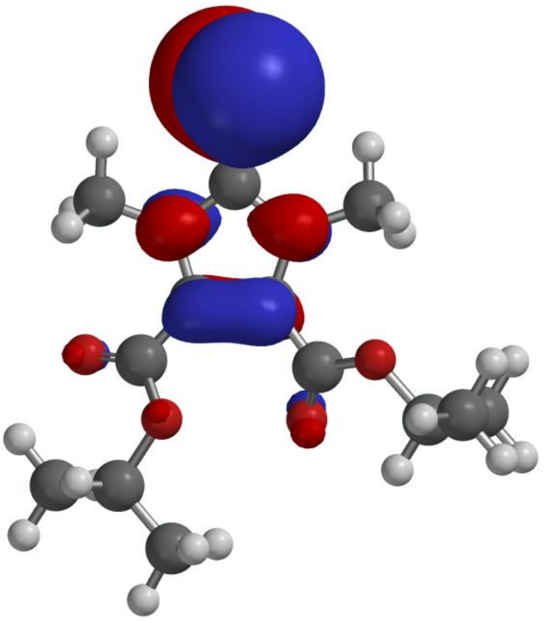

HOMO-1 (-5.50 eV)

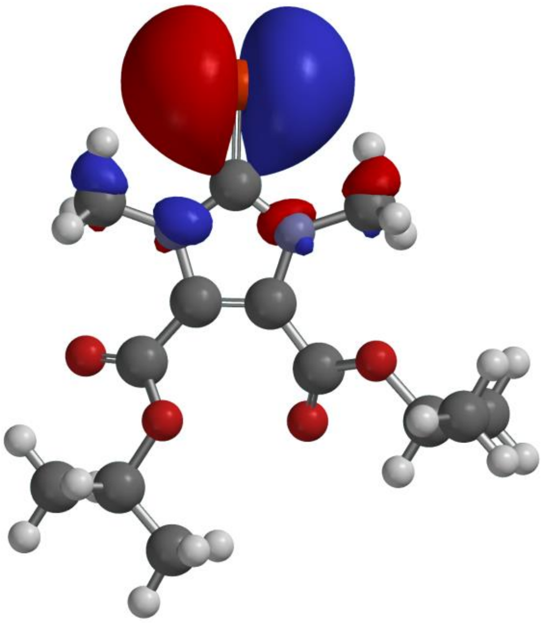

HOMO-2 (-7.84 eV)

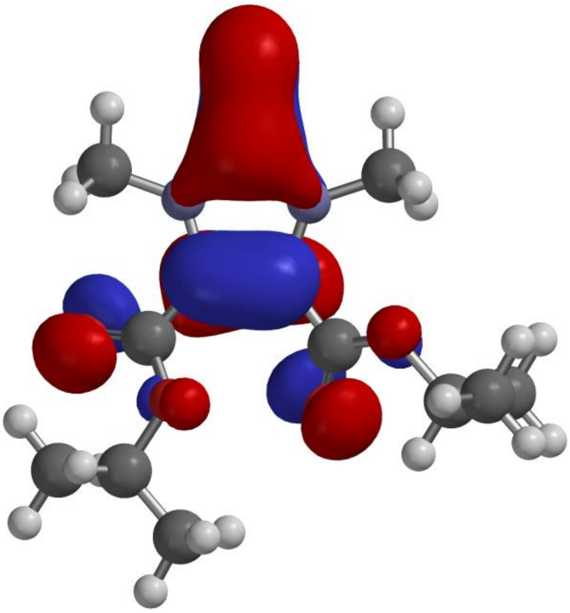

HOMO-3 (-8.31 eV)

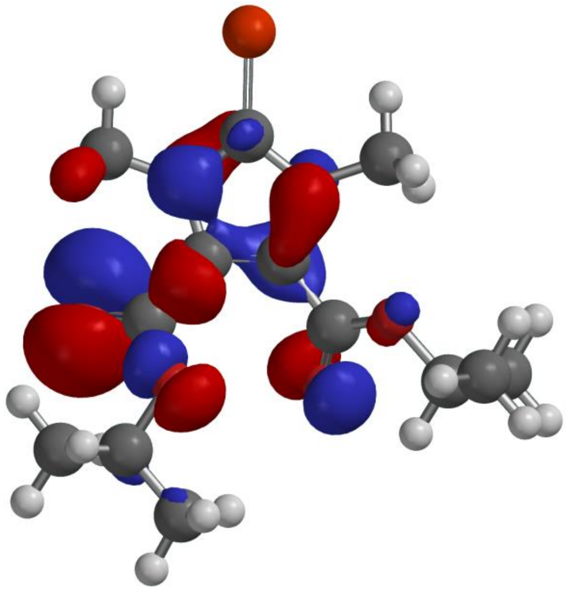

HOMO-4 (-8.43 eV)

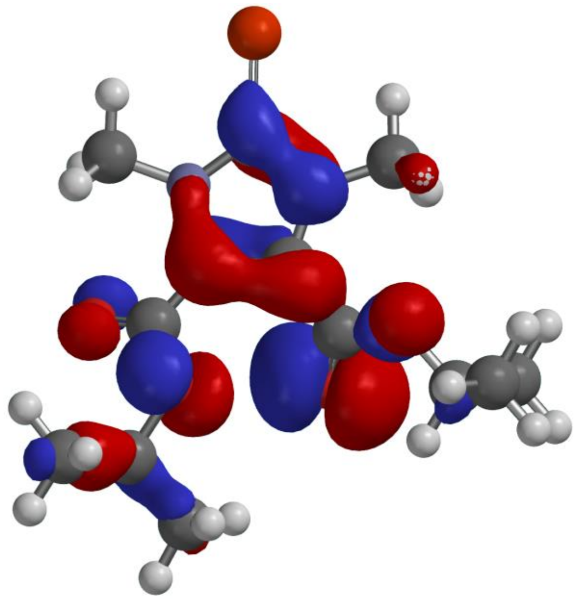

Calculation of 13a/13e

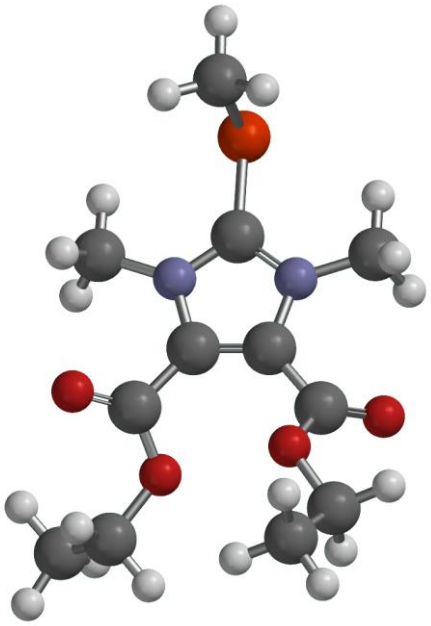

Charge: +1

Number of imaginary frequencies: 0

E: - 3280.7165017418 hartrees

Coordinates (Angstroem)

ATOM	X	Y	Z
C	0.6584356067	0.9453483463	-2.3542105827
N	0.3001284984	-0.3058071958	-1.9876679515
C	0.1763242255	-0.3560778779	-0.6116771954
C	0.4643233779	0.8982242331	-0.1353524292
N	0.7695736932	1.6873750375	-1.2292934028
Se	1.0403091571	1.5370422413	-4.1355998855
C	1.1286619827	3.1186588394	-1.1710461683
C	0.1143624926	-1.4500965922	-2.9011520408
C	-0.2305490836	-1.6148674635	0.0910516190
C	0.5058495075	1.4406607048	1.2602712017
O	1.3366270613	2.2433653446	1.6076197661
O	-0.4779771393	0.9488800911	1.9965699118
C	-0.5246532857	1.3718461740	3.4048223583
C	-1.7973420166	0.8204853579	4.0047915555
O	-1.0422040967	-2.3713320645	-0.3831506857
O	0.4256029210	-1.7574737540	1.2312557455
C	0.1080313855	-2.9331704590	2.0585889241
C	0.9506340029	-4.1276683560	1.6573422795
C	-0.8084578241	1.9927936781	-4.6925577109
H	1.2842970520	3.4664451097	-2.1882655019
H	2.0325031278	3.2352428211	-0.5788377496
H	0.3161045237	3.6740834298	-0.7043211703
H	0.7520659758	-2.2714415318	-2.5787438779
H	0.4037445416	-1.1308205022	-3.8984462720
H	-0.9247500337	-1.7690287820	-2.8728754978
H	0.3732347548	0.9860622528	3.8908537079
H	-0.4873948772	2.4615121459	3.4258646515
H	-1.8211121555	-0.2699816647	3.9560227306
H	-2.6783840116	1.2144002529	3.4945537765
H	-1.8528612376	1.1150479096	5.0554655255
H	-0.9593479338	-3.1318914835	1.9668357667

H	0.3363900806	-2.5959277062	3.0682435077
H	0.7038092170	-4.4711116108	0.6513527803
H	2.0157479796	-3.8929673571	1.7095963253
H	0.7511286039	-4.9498321524	2.3495284061
H	-0.7000678652	2.3279031096	-5.7239914012
H	-1.4460697097	1.1131241106	-4.6551715887
H	-1.1967184991	2.8009953634	-4.0782694277

LUMO+2 (-3.90 eV)

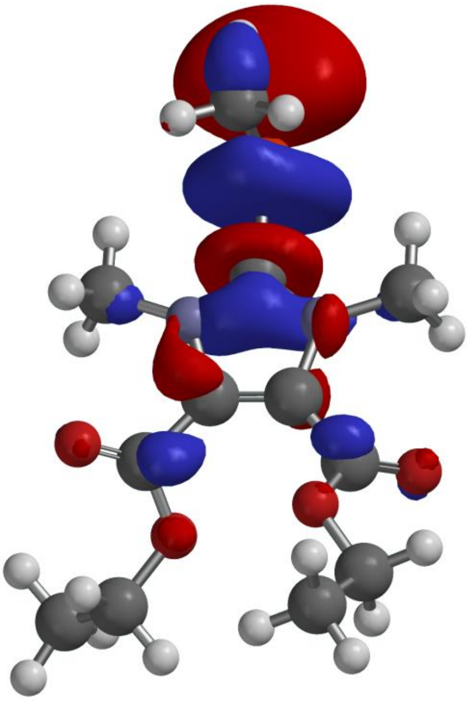

LUMO+1 (-5.40 eV)

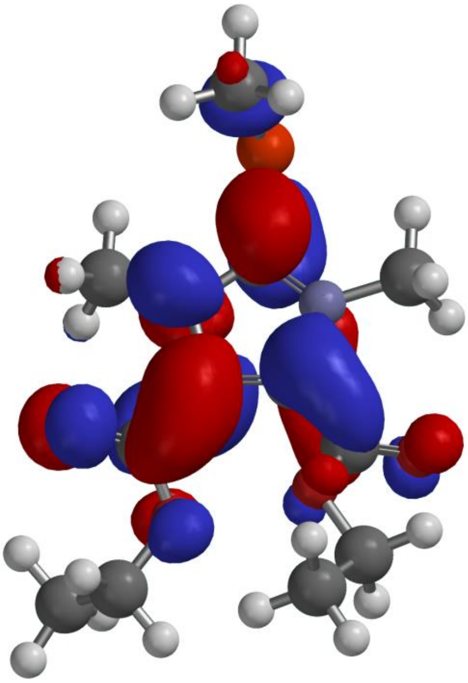

LUMO (-5.41 eV)

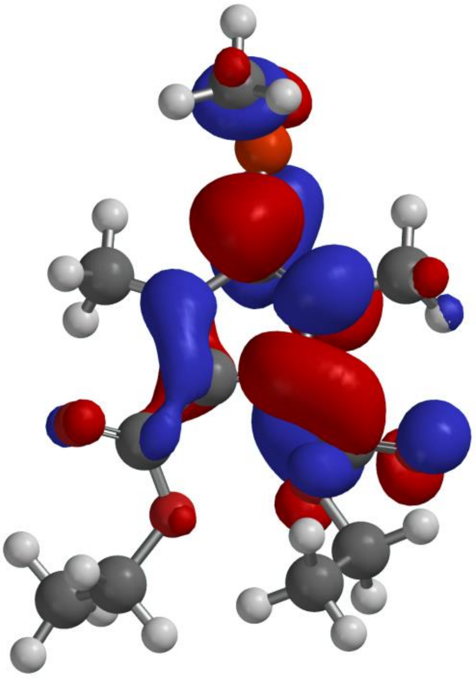

HOMO (-10.53 eV)

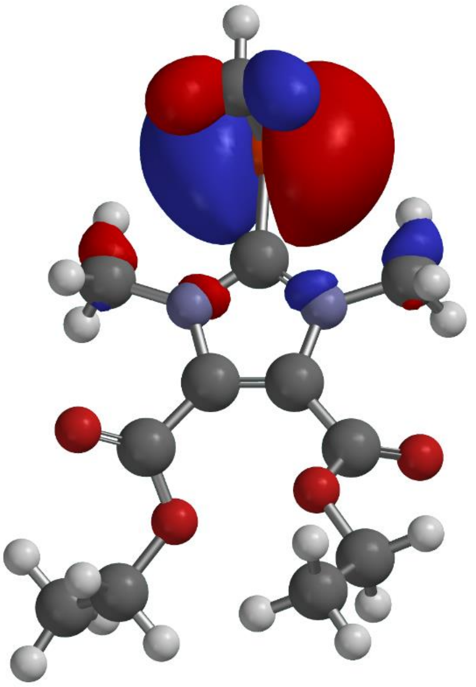

HOMO-1 (-11.16 eV)

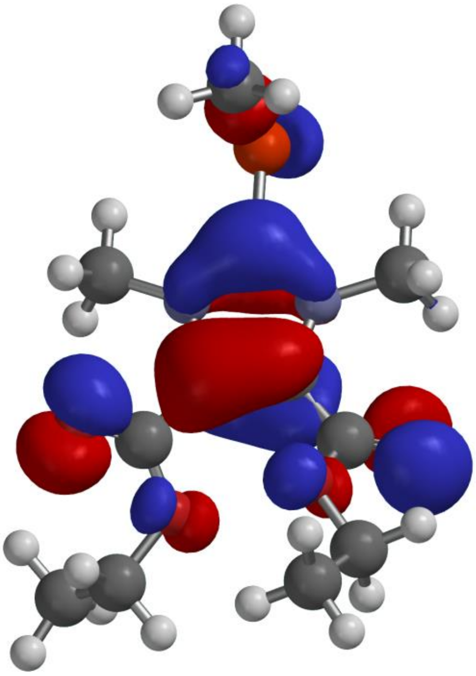

HOMO-2 (-11.60 eV)

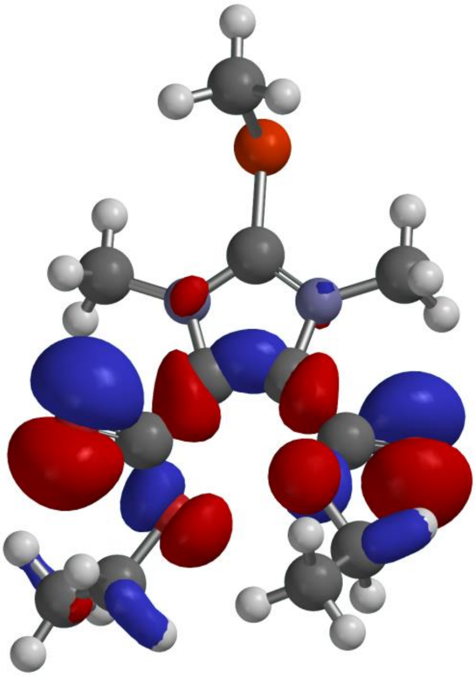

HOMO-3 (-11.70 eV)

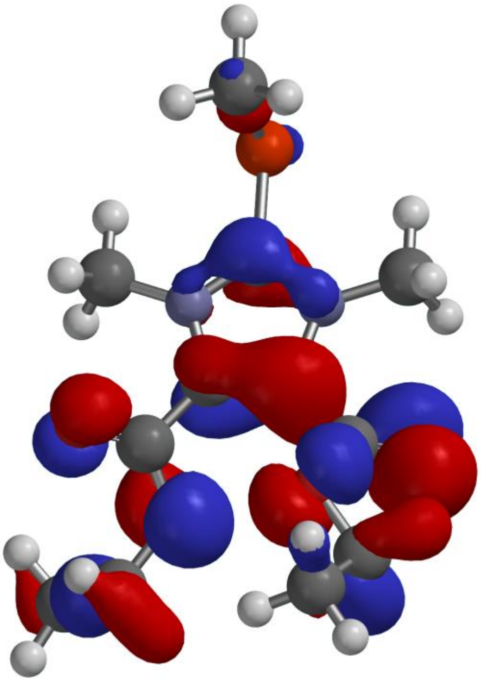

HOMO-4 (-11.84 eV)

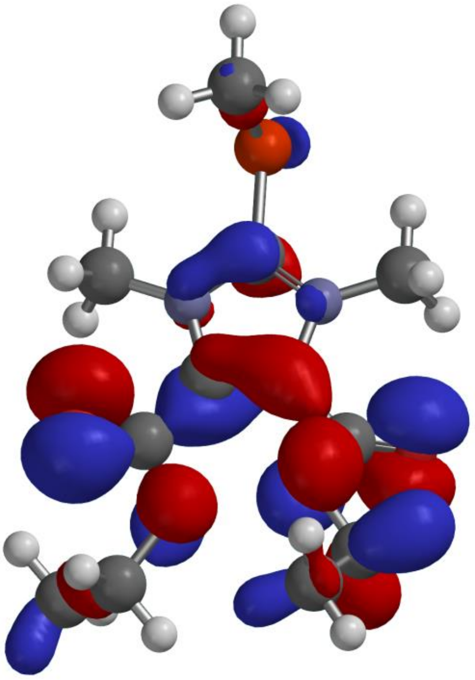

Calculation of 13b

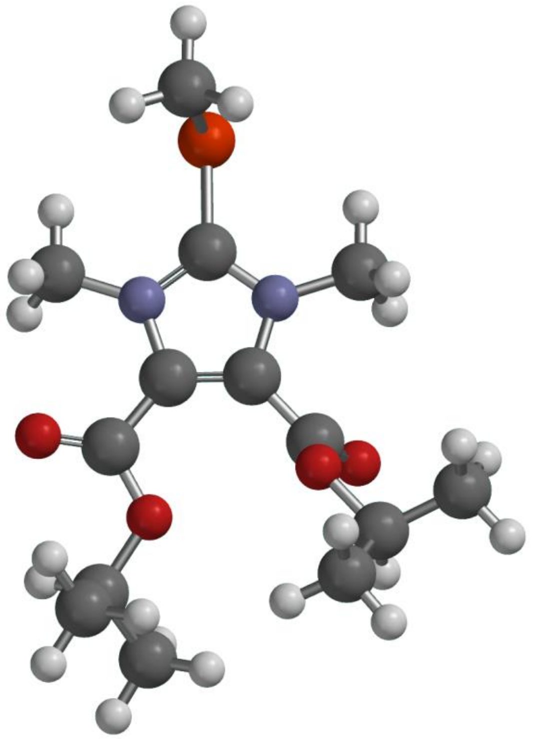

Charge: +1

Number of imaginary frequencies: 0

E: - 3359.3747450457 hartrees

Coordinates (Angstroem)

ATOM	X	Y	Z
C	-0.6254787132	0.9279791767	-2.8817034642
N	-0.6040194582	-0.3095492769	-2.3291091552
C	-0.5191118516	-0.1925670715	-0.9591046958
C	-0.5019153962	1.1467113445	-0.6659233393
N	-0.5709408989	1.8254703450	-1.8763210235
Se	-0.8135825095	1.3099898169	-4.7479670264
C	-0.6157841510	3.2918966392	-2.0461227594
C	-0.6674569374	-1.5944522335	-3.0482867057
C	-0.5512499573	-1.4208954284	-0.0848290892
C	-0.4823301694	1.8519409182	0.6484469815
O	-0.2804682055	3.0396236731	0.7428647847
O	-0.7420665242	1.0094608232	1.6366687627
C	-0.8312920288	1.5657037497	3.0168440664
C	-1.7335949383	0.6140734321	3.7786784056
O	-1.5250969757	-2.1311407806	-0.0625995270
O	0.5962934019	-1.6012478589	0.5428819220
C	0.7266846322	-2.8004062693	1.4203767195
C	1.7925481501	-2.4502955360	2.4404632579
C	1.0827647699	1.7165492245	-5.1648851363
C	0.5662367837	1.7070115857	3.5965918088
C	1.0693270773	-4.0070849325	0.5614357727
H	-1.4230556702	3.6983477951	-1.4423869396
H	-0.7923297255	3.4968570728	-3.0986277220
H	0.3243796138	3.7259850850	-1.7138574259
H	0.2406729681	-2.1633654683	-2.8479763391
H	-0.7486228009	-1.3896311743	-4.1117840055
H	-1.5371513032	-2.1492965167	-2.7011348716

H	-1.2977479025	2.5464761104	2.9139312406
H	-1.2903418748	-0.3824791432	3.8446688116
H	-2.7124550297	0.5312081160	3.3030554328
H	-1.8788762360	0.9893944874	4.7942001601
H	-0.2461833253	-2.9344259788	1.8954586792
H	1.5112340822	-1.5720959242	3.0232932192
H	1.9200368158	-3.2882974422	3.1296340854
H	2.7526156281	-2.2571812321	1.9560803280
H	1.0834129971	1.9023100793	-6.2387929924
H	1.4063124970	2.6081660601	-4.6340926854
H	1.7095128991	0.8589969739	-4.9352684971
H	1.0678088390	0.7379341130	3.6474041515
H	1.1751288893	2.3958268717	3.0096999051
H	0.4937681364	2.1054691171	4.6115213235
H	2.0177148207	-3.8556271759	0.0394237435
H	0.2822033621	-4.2167586903	-0.1642754845
H	1.1724962194	-4.8865844767	1.2014253230

LUMO+2 (-3.83 eV)

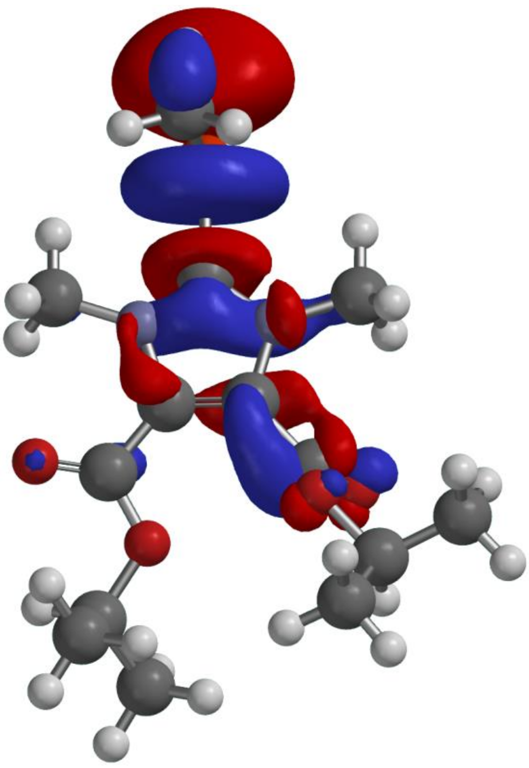

LUMO+1 (-5.04 eV)

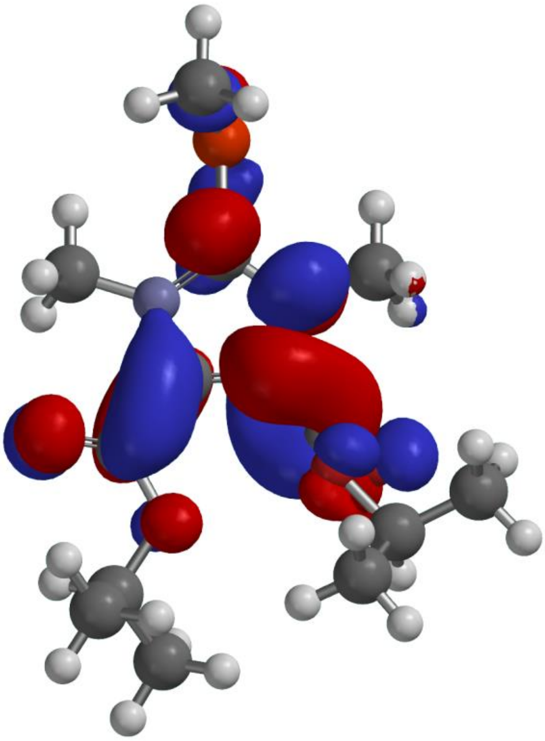

LUMO (-5.38 eV)

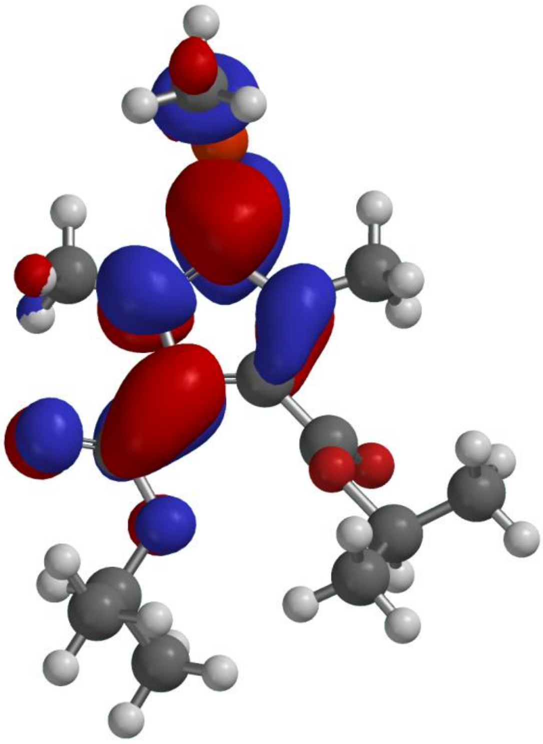

HOMO (-10.46 eV)

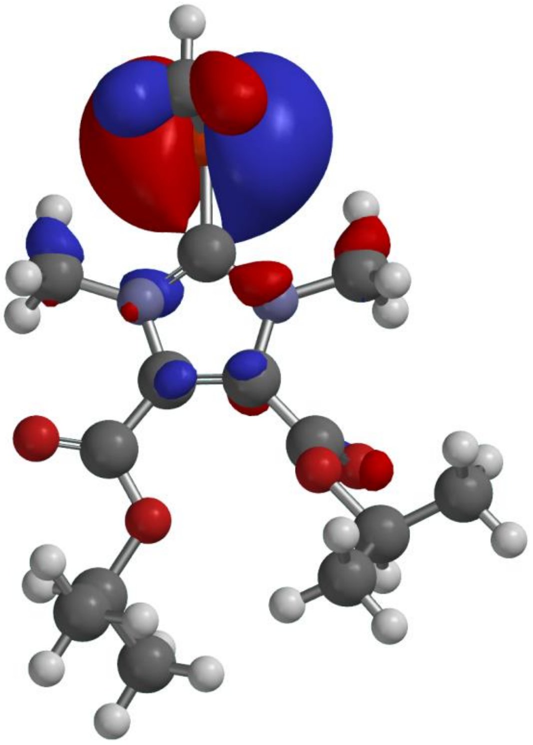

HOMO-1 (-11.04 eV)

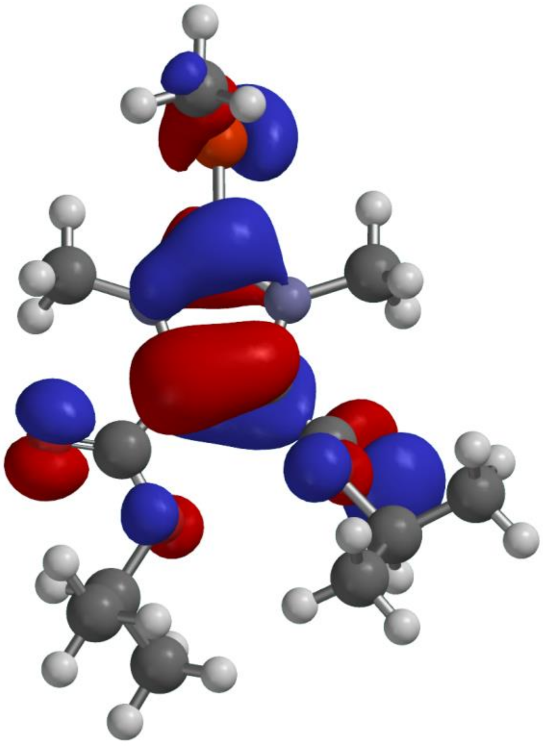

HOMO-2 (-11.38 eV)

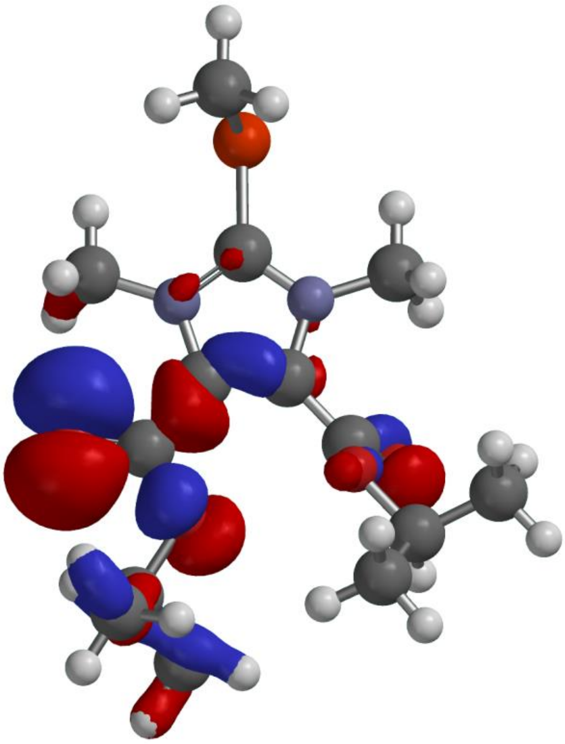

HOMO-3 (-11.55 eV)

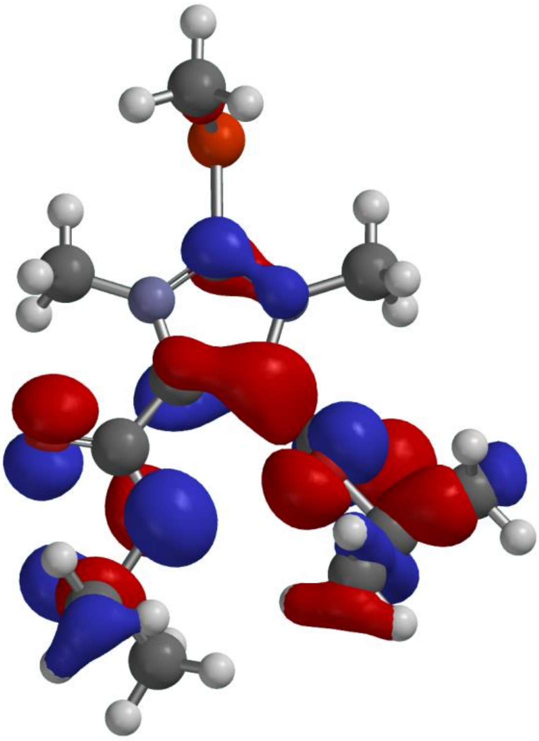

HOMO-4 (-11.74 eV)

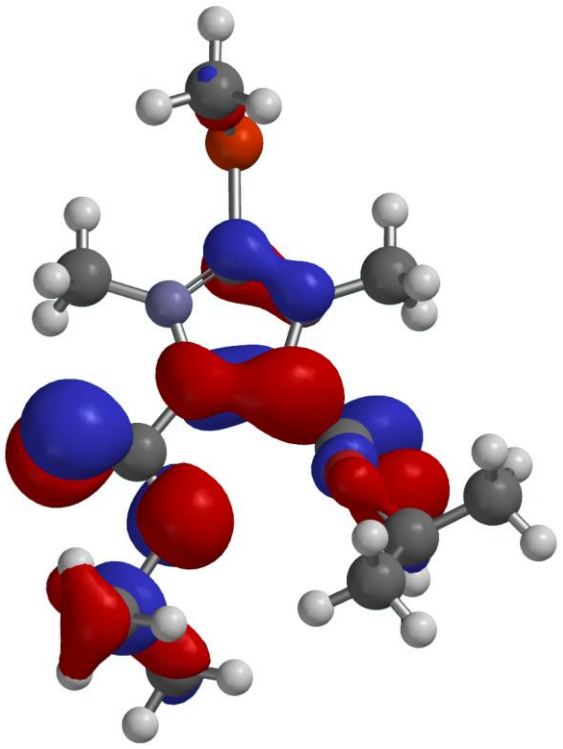

Calculation of **14a**

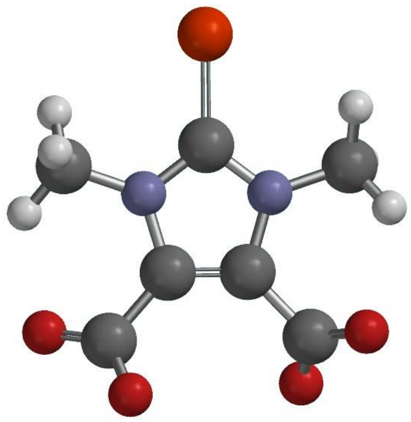

Charge: -2

Number of imaginary frequencies: 0

E: - 3082.5815222373 hartrees

Coordinates (Angstroem)

ATOM	X	Y	Z
C	0.0137831865	1.1446237359	-2.4592397280
C	-0.0070182883	0.0007578380	-1.3746924829
N	-1.0866858009	-0.0964383125	-0.5619907267
C	-0.6967481184	-0.0573979654	0.7933474853
C	0.6686120771	0.0518429597	0.7950508023
N	1.0678364581	0.0922680267	-0.5502752787
Se	0.0020124829	0.0165775464	-3.2647712346

C	2.4484617814	0.1604171057	-1.0034645577
C	-2.4604672479	-0.1382866069	-1.0431018438
C	-1.7255873187	-0.1197914880	1.9396080966
C	1.7388487317	0.0891644483	1.9087135333
O	2.5035382779	1.0891585651	1.8623690855
O	1.7714275561	-0.8949128893	2.6706543588
O	-2.6711765802	-0.9393688132	1.7649202089
O	-1.5320482695	0.6597654075	2.8891966353
H	2.8679307740	-0.8468049789	-1.1038427161
H	2.4669156907	0.6431875138	-1.9801437568
H	3.0094674421	0.7232633286	-0.2584489460
H	-2.4857768321	-0.7100632008	-1.9708646783
H	-2.8201868146	0.8751717060	-1.2539658696
H	-3.0593560015	-0.5985101909	-0.2582981146

LUMO+2 (5.31 eV)

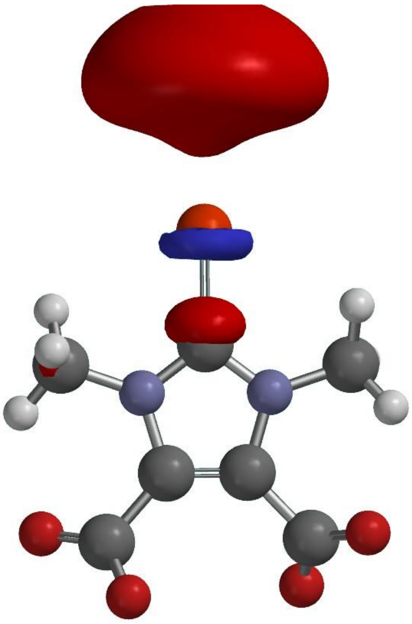

LUMO+1 (4.91 eV)

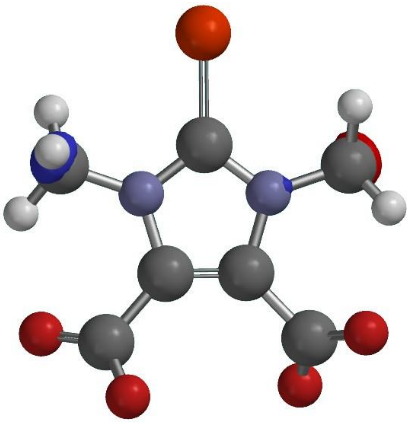

LUMO (4.67 eV)

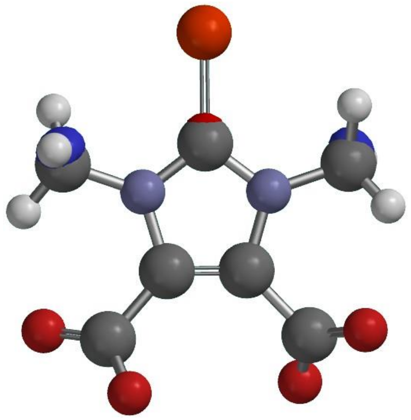

HOMO (1.28 eV)

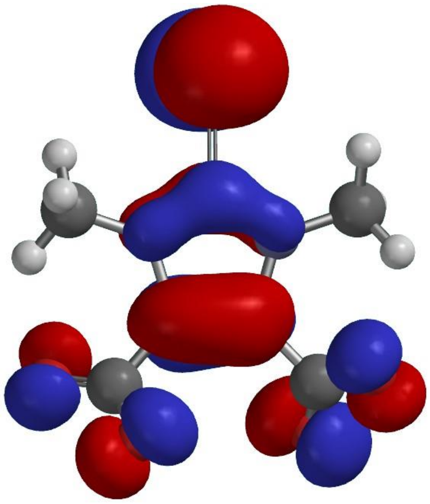

HOMO-1 (0.55 eV)

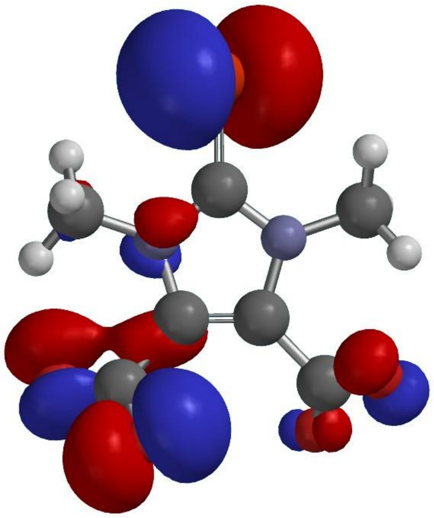

HOMO-2 (0.49 eV)

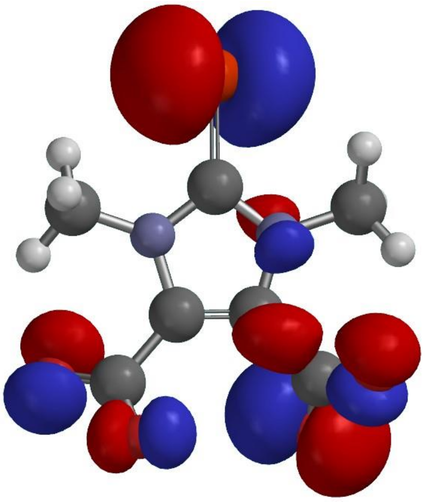

HOMO-3 (0.40 eV)

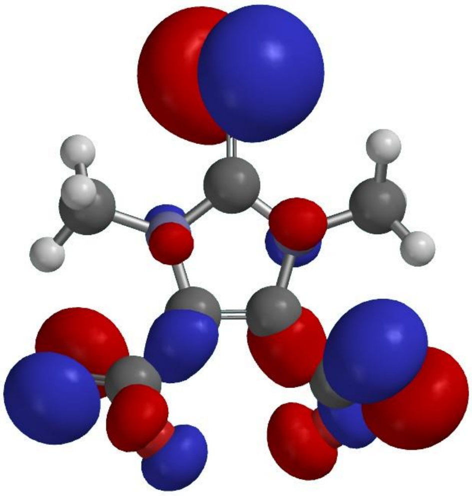

HOMO-4 (0.27 eV)

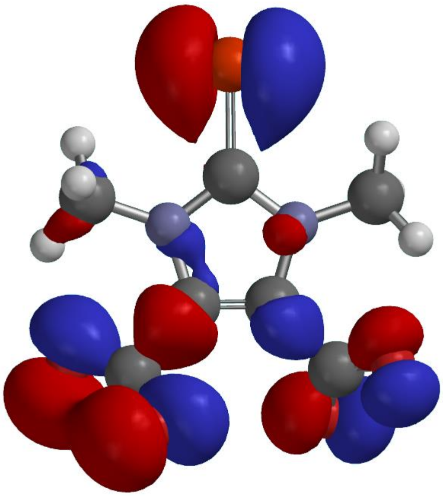

Calculation of 16a

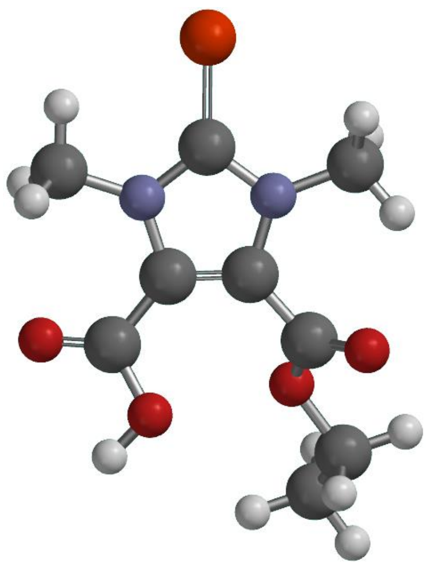

Charge: 0

Number of imaginary frequencies: 0

E: - 3162.3848986637 hartrees

Coordinates (Angstroem)

ATOM	X	Y	Z
C	0.3074874940	0.9288061349	-2.5424267732
N	0.0925269619	-0.4178139462	-2.4026483968
C	-0.2737299139	-0.7074095317	-1.0905501680
C	-0.2834029377	0.4809032931	-0.4045652928
N	0.0516462529	1.4730408702	-1.2994024672
Se	0.8420533469	1.8528100076	-4.0345846205
C	0.2116623704	2.8971118220	-1.0084932532
C	0.1991643329	-1.3725378111	-3.5070656731
C	-0.6156730410	-2.0618984561	-0.6440932353
C	-0.5870822802	0.7732921412	1.0264981941
O	-1.3737048482	1.6190045032	1.3813700768
O	0.1511933055	0.0200703284	1.8441983498
C	-0.1024720035	0.1693104075	3.2702183118
C	0.8703574691	-0.7298568462	4.0024467838
O	-0.3258014766	-3.0797326182	-1.2299013236
O	-1.3302648043	-2.0589826013	0.5060908263
H	-0.2135488383	3.4621401527	-1.8363918387
H	-0.3107216047	3.1284283466	-0.0847816153
H	1.2721171763	3.1453526102	-0.9311429835
H	0.9840502251	-2.0981265113	-3.3009489855
H	0.4376614705	-0.7944284509	-4.3975735217
H	-0.7461766539	-1.8982953825	-3.6393113408
H	0.0268174293	1.2207076047	3.5342922505
H	-1.1431424587	-0.1024007188	3.4599318614
H	1.9032825208	-0.4538737579	3.7801264045
H	0.7141823599	-0.6329008880	5.0800247046
H	0.7238342464	-1.7763348526	3.7273234224
H	-1.4823161007	-2.9863858494	0.7413603028

LUMO+2 (-0.68 eV)

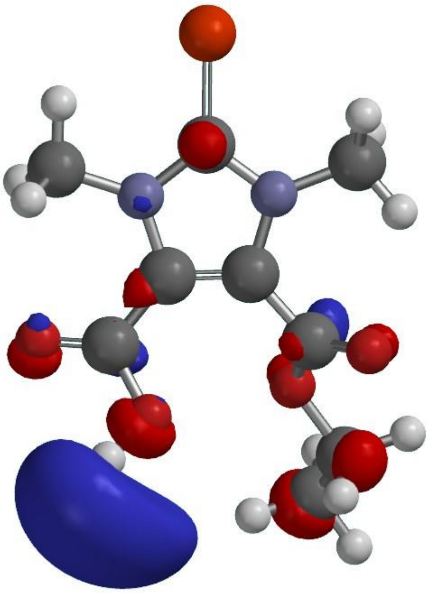

LUMO+1 (-0.88 eV)

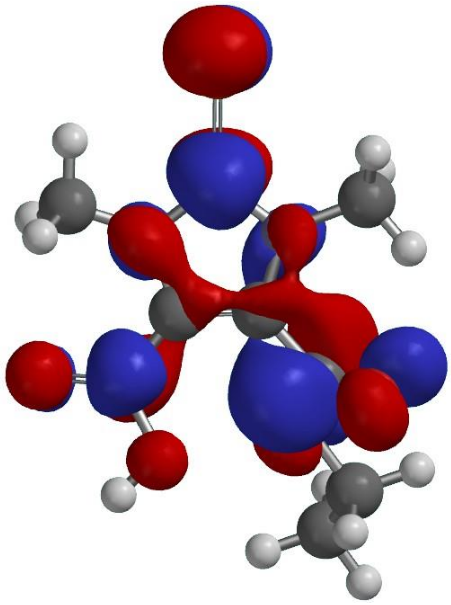

LUMO (-2.30 eV)

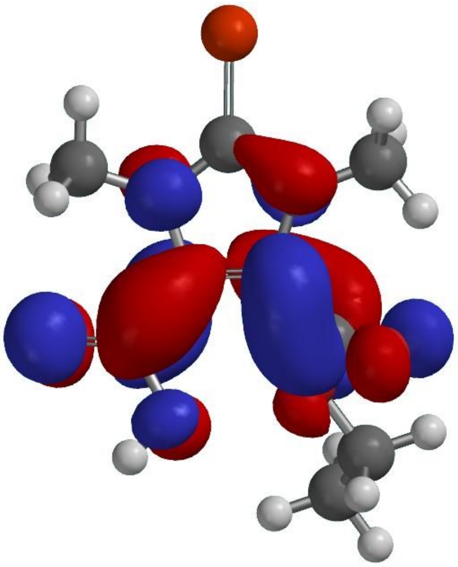

HOMO (-5.51 eV)

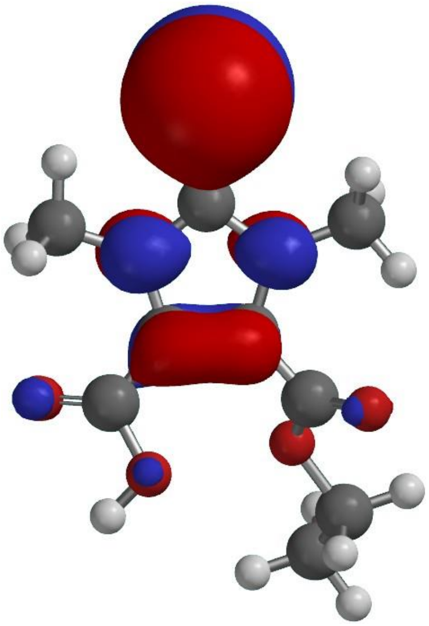

HOMO-1 (-5.60 eV)

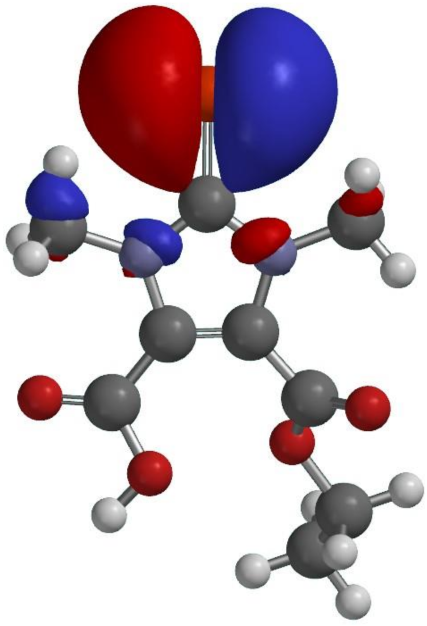

HOMO-2 (-8.08 eV)

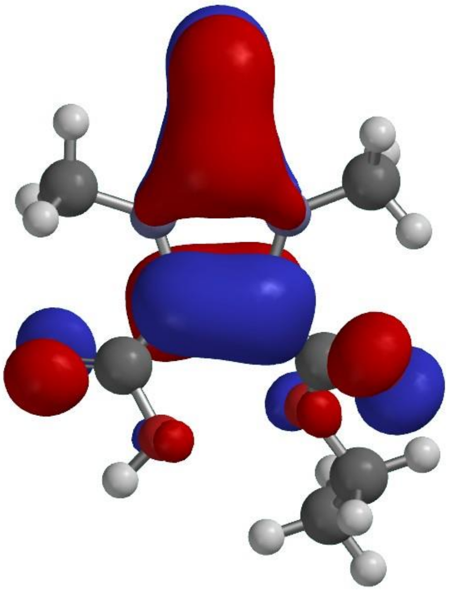

HOMO-3 (-8.56 eV)

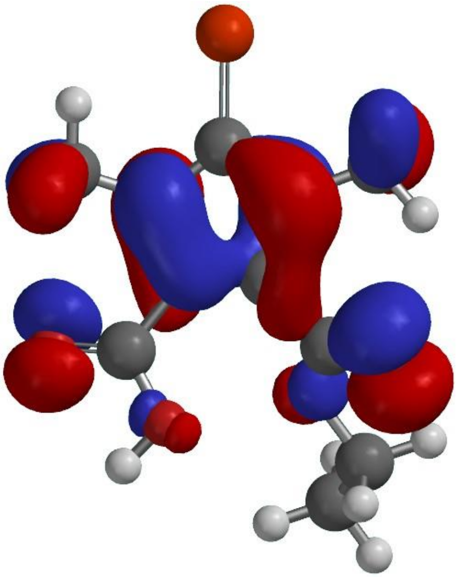

HOMO-4 (-8.65 eV)

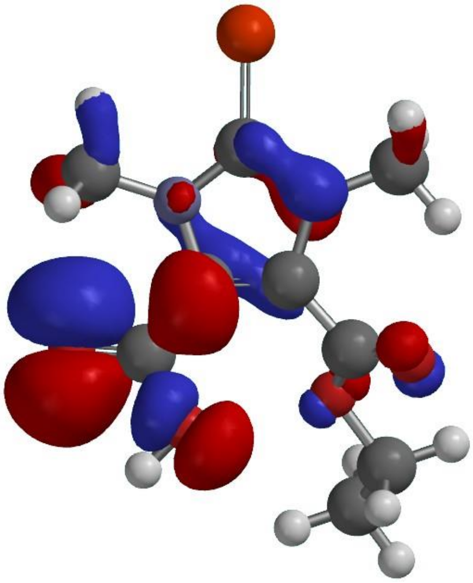

Calculation of 16b

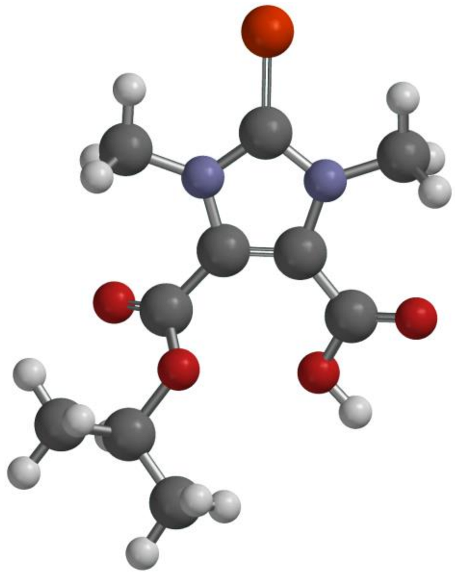

Charge: 0

Number of imaginary frequencies: 0

E: -3201.7131795003 hartrees

Coordinates (Angstroem)

ATOM	X	Y	Z
C	-0.3523422458	0.2129330216	-3.0373180305
N	0.9647342953	0.1925377134	-2.6558403553
C	1.0672420299	-0.1366461396	-1.3065957676
C	-0.2115322514	-0.3021351047	-0.8377625906
N	-1.0677080541	-0.0943594705	-1.8958201302
Se	-1.0368593202	0.5624105519	-4.7050467762
C	-2.5291831151	-0.1393626344	-1.8325727861
C	2.0765257430	0.4115899449	-3.5833440285
C	2.3577370027	-0.2938139641	-0.6239405735
C	-0.7290164727	-0.6580584442	0.5150441407
O	-1.5638856316	-1.5151131561	0.6887597259
O	-0.1898671279	0.1072752565	1.4651960693
C	-0.5528961870	-0.1772365959	2.8615824080
C	0.6568465538	0.2115982904	3.6940759876
O	3.4049411461	0.1818151802	-0.9987969681
O	2.2637738553	-1.0685715272	0.4817418410
C	-1.8145332117	0.5980396588	3.2149111991
H	-2.8943003349	0.6101077519	-1.1277740575
H	-2.8965867240	0.0877788242	-2.8313405480
H	-2.8551626729	-1.1285140989	-1.5169610818
H	1.6441599054	0.7845528028	-4.5094494717
H	2.5972398718	-0.5285533395	-3.7765462017
H	2.7742257613	1.1271218297	-3.1571235464
H	-0.7415719583	-1.2500443679	2.9279675899
H	0.8760868549	1.2771170053	3.5893570871
H	1.5367920529	-0.3574984490	3.3895376359
H	0.4597350095	0.0026363741	4.7485980689
H	3.1460699584	-1.0866318862	0.8815855909
H	-1.6508698283	1.6730787813	3.1017809536
H	-2.6501209497	0.2957166486	2.5817887695
H	-2.0896739546	0.4002295425	4.2543058460

LUMO+2 (-0.67 eV)

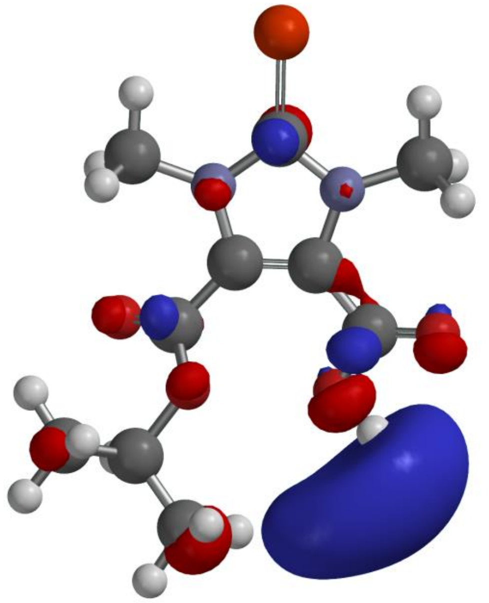

LUMO+1 (-0.85 eV)

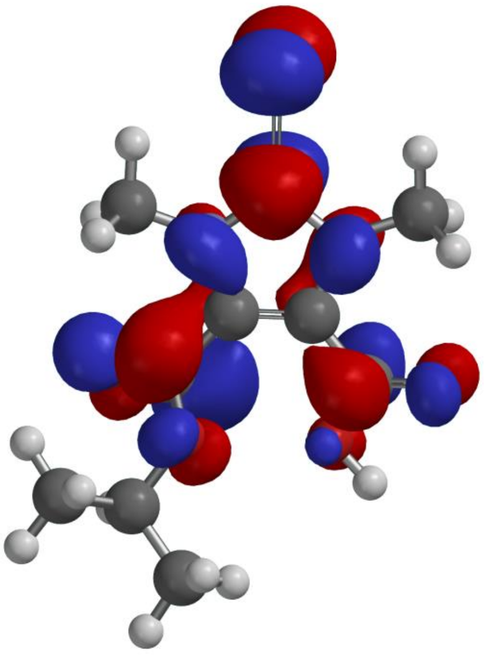

LUMO (-2.31 eV)

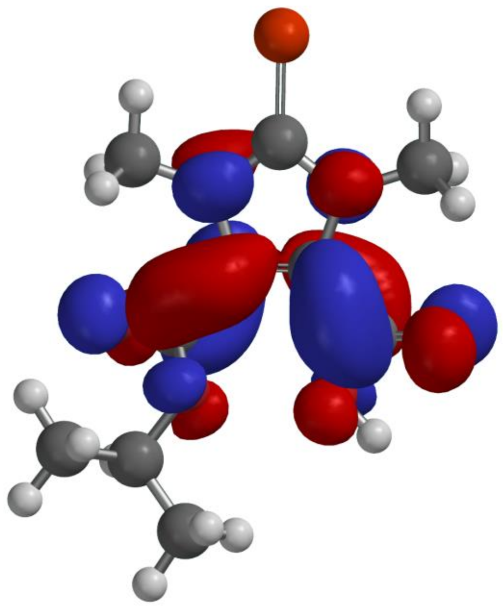

HOMO (-5.47 eV)

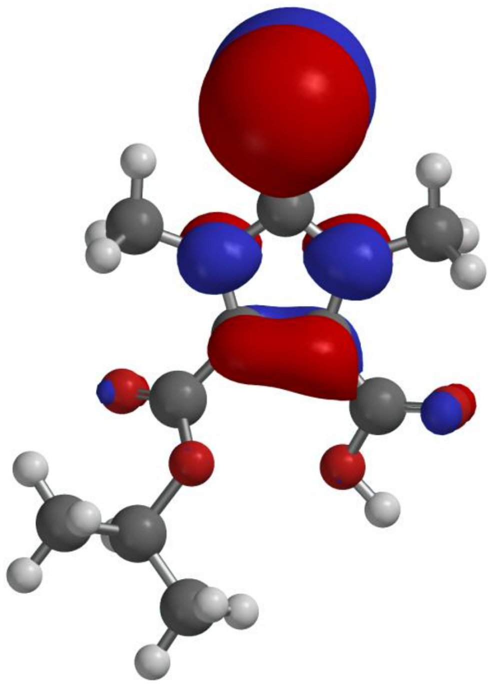

HOMO-1 (-5.60 eV)

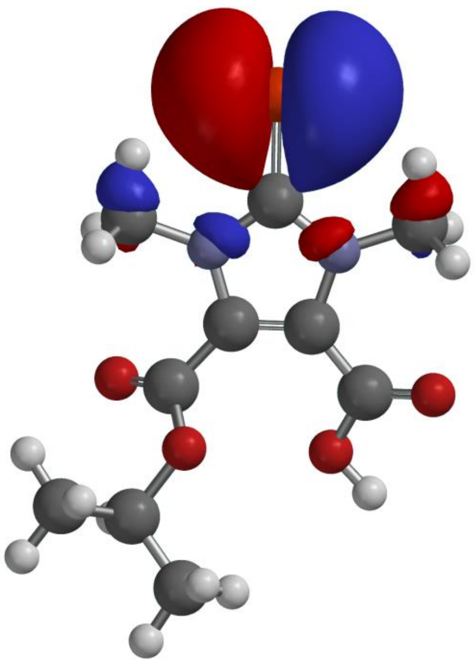

HOMO-2 (-8.07 eV)

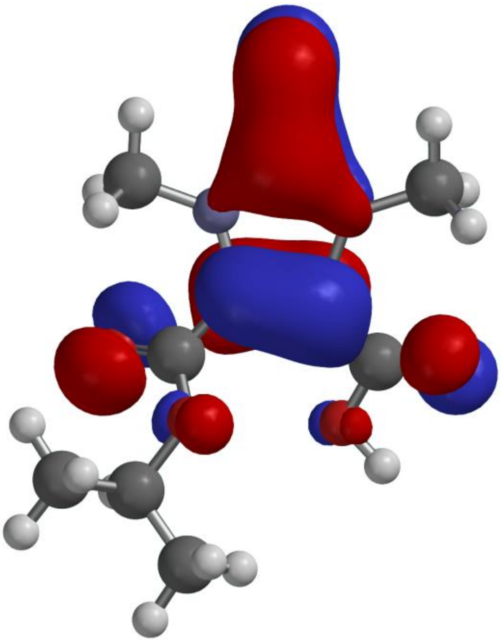

HOMO-3 (-8.58 eV)

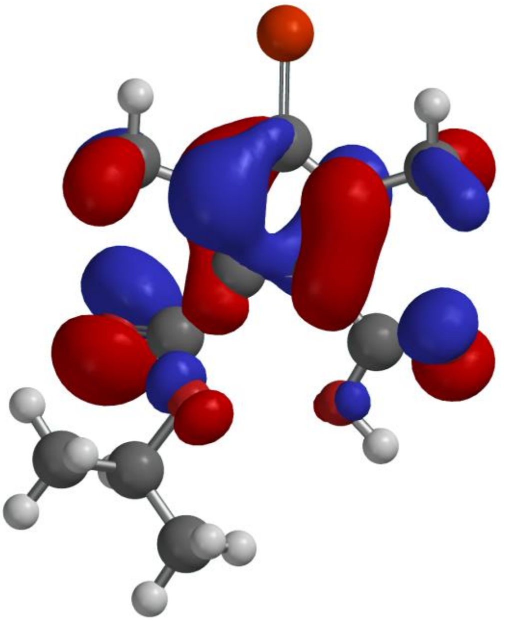

HOMO-4 (-8.65 eV)

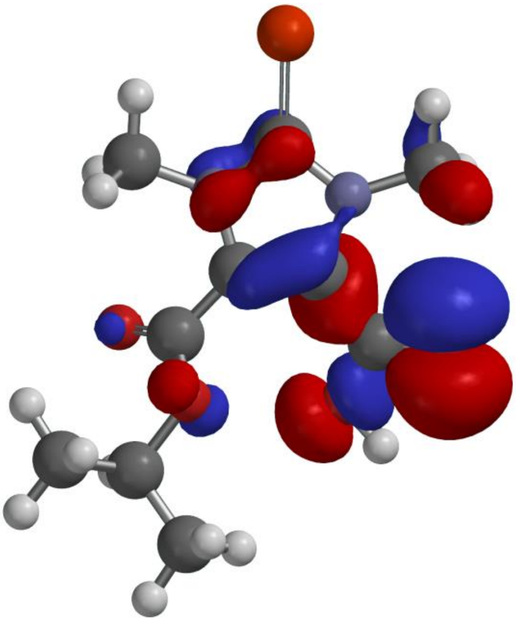

Calculation of 17a

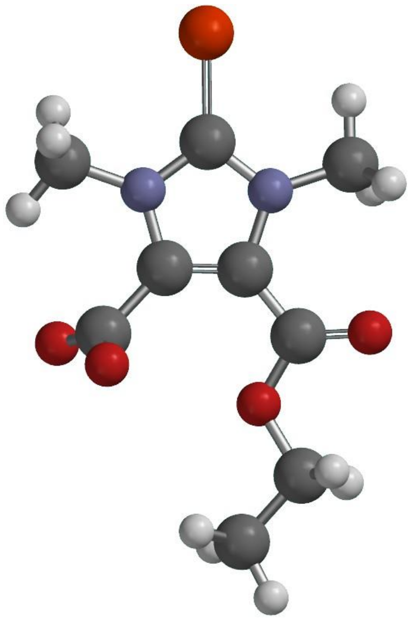

Charge: -1

Number of imaginary frequencies: 0

E: -3161.8668676293 hartrees

Coordinates (Angstroem)

ATOM	X	Y	Z
C	0.0137831865	1.1446237359	-2.4592397280
N	0.0418298616	-0.2215707985	-2.2996694071
C	0.0194678919	-0.5698746701	-0.9663464928
C	-0.0081511747	0.6123509440	-0.2631542177
N	-0.0201154550	1.6606539146	-1.2056069633
Se	0.0256352695	2.0618577728	-4.0766319573
C	-0.0043482542	3.0864729619	-0.9074389911
C	0.0640314884	-1.1751397335	-3.4034924314
C	-0.0149366475	-2.0525807909	-0.5243987197
C	-0.0947135229	0.8278222292	1.1710703001
O	-0.3882606399	1.8792226350	1.7196838187
O	0.1895976366	-0.3024758531	1.8470916347
O	-1.1101303977	-2.4322619811	-0.0770599040
O	1.0536819311	-2.6606148873	-0.7327897928
C	-0.0924503809	-0.3188683222	3.2559442964
C	0.1229112697	-1.7435043179	3.7304970145
H	0.0081792188	3.6110062726	-1.8613893512
H	-0.8840035466	3.3590223004	-0.3261961966
H	0.8856458152	3.3393452647	-0.3280736001
H	0.4306529652	-2.1229971944	-3.0146927437
H	0.7241978515	-0.7941695564	-4.1815350415
H	-0.9385196502	-1.2915019540	-3.8215218871
H	0.5670897589	0.3907258365	3.7653310420
H	-1.1227063290	0.0115715875	3.4147433643
H	1.1623307053	-2.0474057702	3.5863746577
H	-0.1206916225	-1.8228220933	4.7948657093
H	-0.5100072292	-2.4288875323	3.1636355877

LUMO+2 (+2.31 eV)

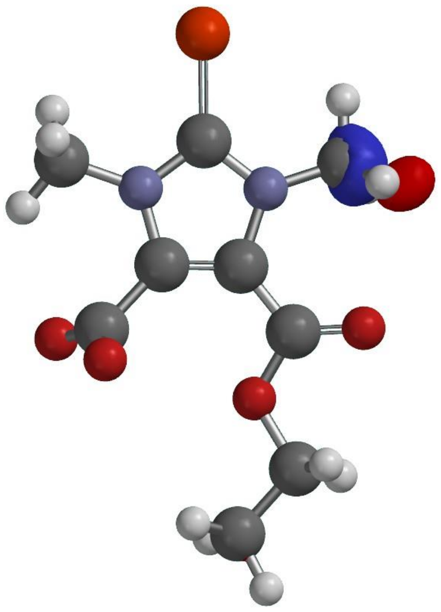

LUMO+1 (+1.97 eV)

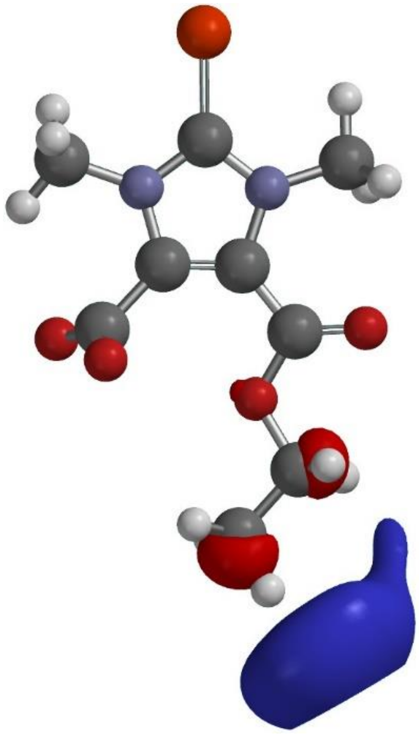

LUMO (+1.82 eV)

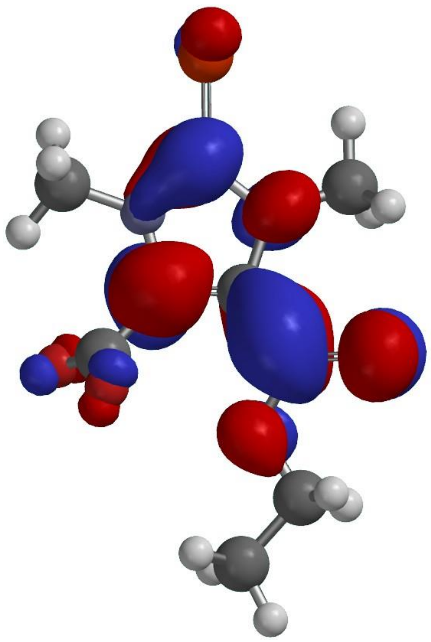

HOMO (-2.24 eV)

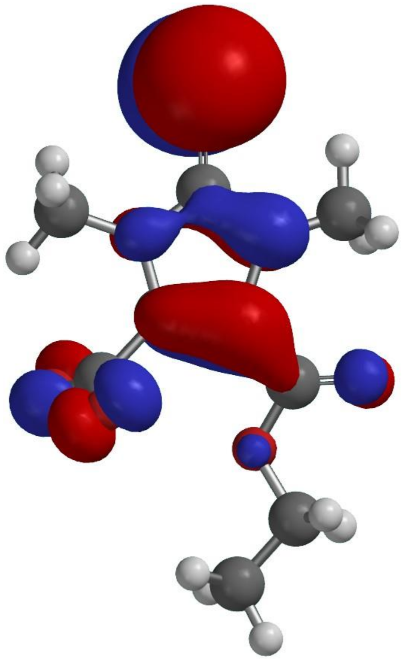

HOMO-1 (-2.60 eV)

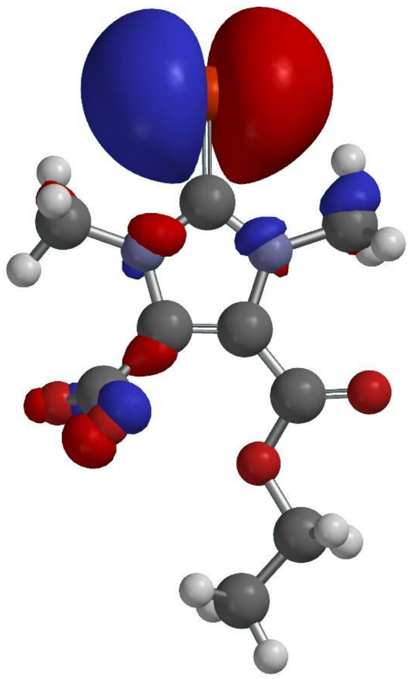

HOMO-2 (-2.93 eV)

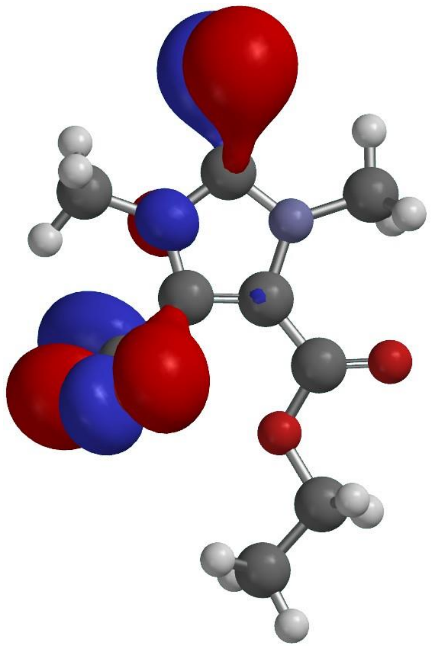

HOMO-3 (-3.08 eV)

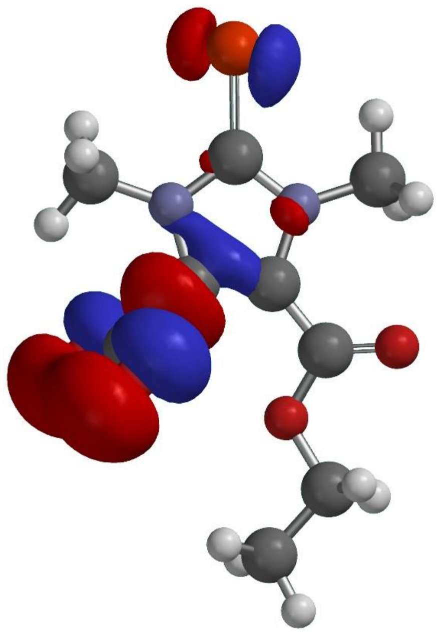

HOMO-4 (-3.26 eV)

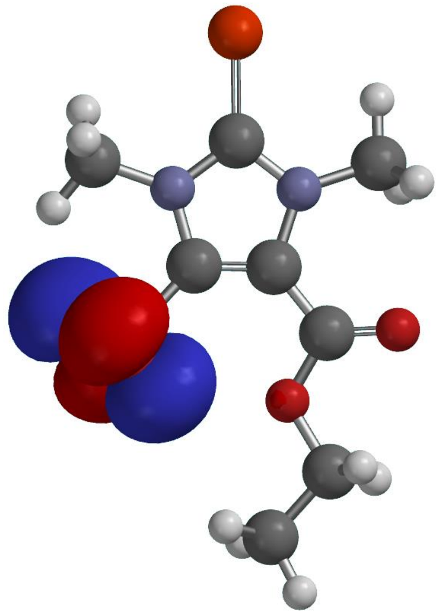

Calculation of 17b

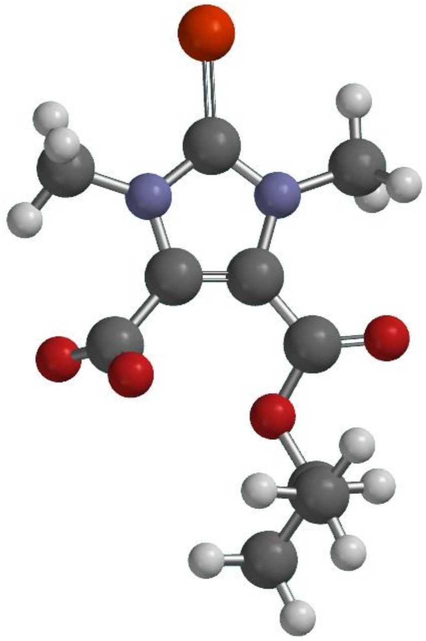

Charge: -1

Number of imaginary frequencies: 0

E: -3201.1954433489 hartrees

Coordinates (Angstroem)

ATOM	X	Y	Z
C	0.3314779445	1.1197609132	-2.8252407063
N	0.1741882181	-0.2334497381	-2.6343329451
C	0.0950645414	-0.5427415616	-1.2927811834
C	0.2258782526	0.6489837040	-0.6182777100
N	0.3602728041	1.6655393041	-1.5840310967
Se	0.4668811770	1.9914325693	-4.4633169241
C	0.5868848944	3.0791824193	-1.3150009274
C	0.0761160900	-1.2078110661	-3.7162935210
C	-0.1583413502	-1.9914009119	-0.8098134898
C	0.1294029188	0.9053983190	0.8100775428
O	-0.0945808987	1.9906401301	1.3244123621
O	0.3227463179	-0.2299644379	1.5094360346
O	-1.2788689408	-2.1792738671	-0.3049896546
O	0.7866078051	-2.7708751093	-1.0428595989
C	-0.0989536441	-0.2727944389	2.8961808401
C	0.6920631516	-1.4108676414	3.5247847522
H	0.6447416466	3.5827536010	-2.2785404658
H	-0.2255769050	3.4795124084	-0.7112004998
H	1.5237343823	3.2114547488	-0.7688276140
H	0.3474073479	-2.1803267777	-3.3101494601
H	0.7577712458	-0.9118395896	-4.5125279381
H	-0.9407928601	-1.2280516677	-4.1154279303
H	0.1646088234	0.6823365499	3.3581387725
H	1.7657265086	-1.2218940195	3.4505496853
H	0.4284619981	-1.5129225748	4.5819287895
H	0.4731234448	-2.3506589138	3.0123689106
C	-1.6088183767	-0.4893247581	2.9494432487
H	-2.1365009241	0.3678368193	2.5258280991
H	-1.9350339616	-0.6125591723	3.9875260252
H	-1.8756916515	-1.3780752406	2.3729366026

LUMO+2 (+2.30 eV)

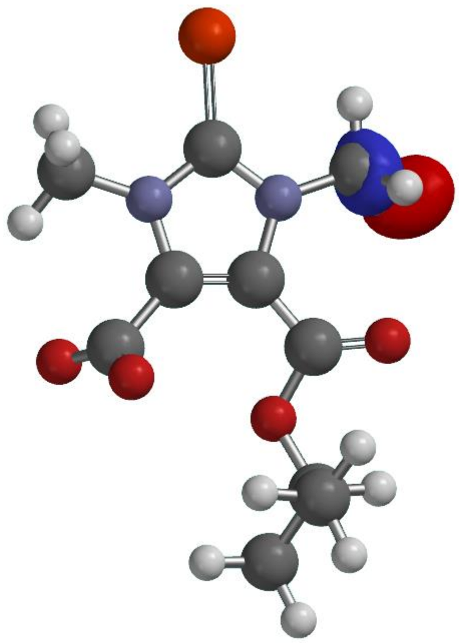

LUMO+1 (+1.93 eV)

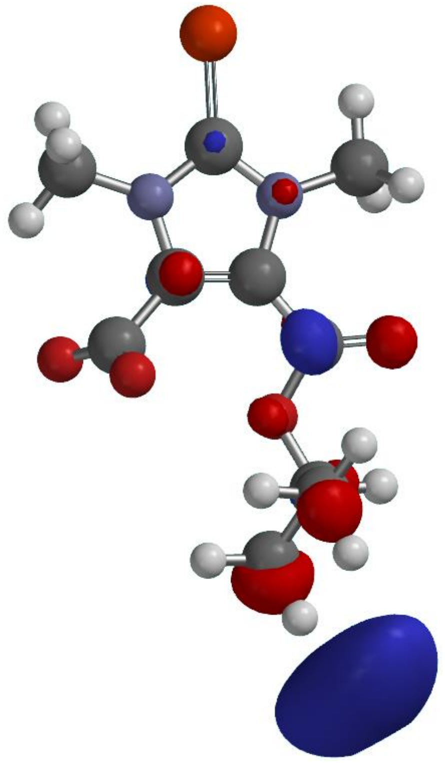

LUMO (+1.78 eV)

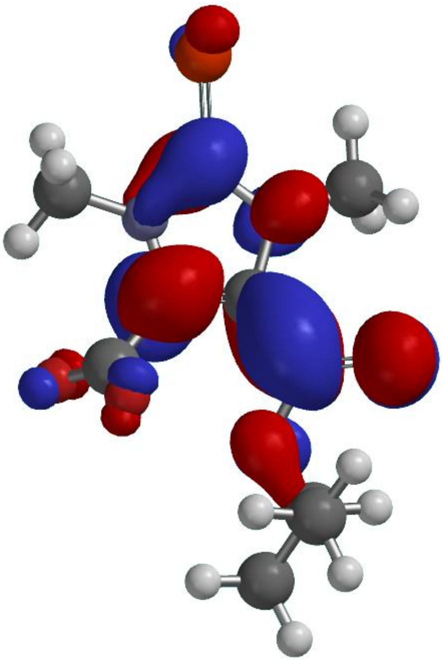

HOMO (-2.25 eV)

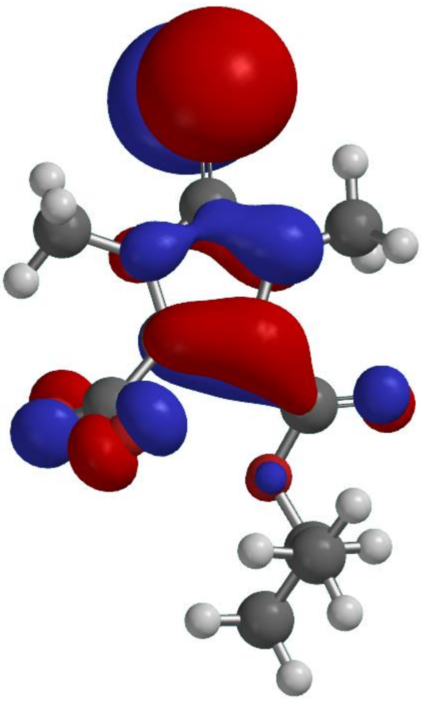

HOMO-1 (-2.60 eV)

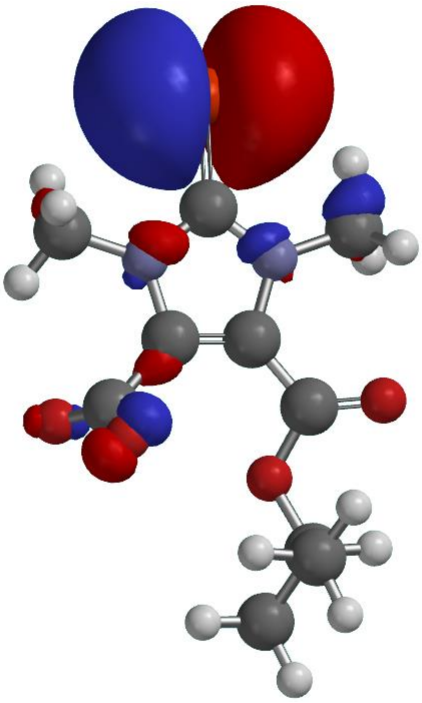

HOMO-2 (-2.95 eV)

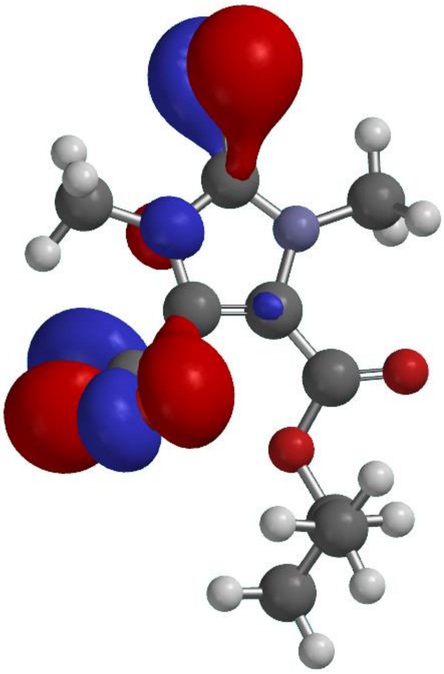

HOMO-3 (-3.12 eV)

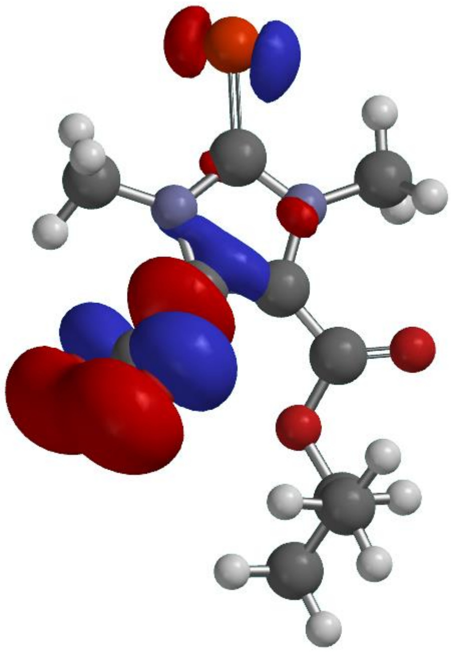

HOMO-4 (-3.29 eV)

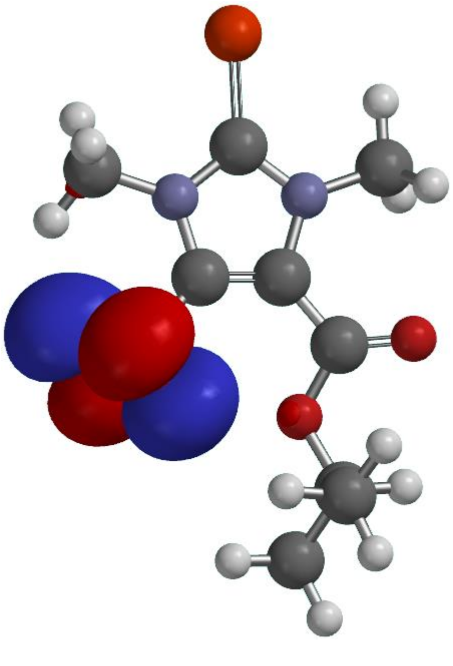

Calculation of carbene of 12b

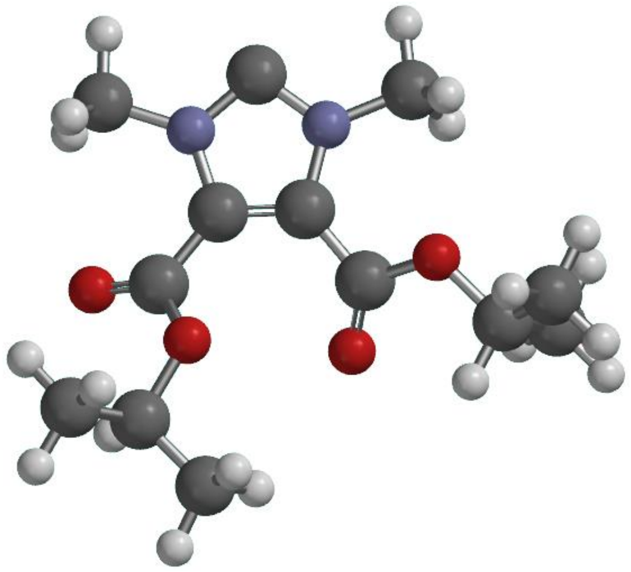

Charge: 0

Number of imaginary frequencies: 0

E: -918.0794365633 hartrees

Coordinates (Angstroem)

ATOM	X	Y	Z
C	-1.0650756496	0.5955612277	-3.2588900025
N	0.1148522624	0.4875706912	-2.5785627885
C	-0.0572596917	-0.0059163587	-1.2871890649
C	-1.4076289635	-0.2138816555	-1.1442555470
N	-1.9842898255	0.1561884445	-2.3512649749
C	-3.4156792478	0.1448004138	-2.6539085951
C	1.3849027836	0.8021891448	-3.2302506699
C	1.0188625088	-0.3255221225	-0.3211312192
C	-2.1963142606	-0.7316636344	-0.0033744435
O	-3.1861974160	-1.4183540511	-0.1385673522
O	-1.6983843242	-0.3276419746	1.1705000447
C	-2.2822104493	-0.8913276683	2.3914687135
C	-1.1511952760	-0.9468861743	3.4052290881
O	0.9838330576	-1.2544571027	0.4514677324
O	2.0445859553	0.5450136658	-0.4153367902
C	3.1970170889	0.3328276353	0.4656192115
C	3.8318478570	1.7003967594	0.6573410846
C	-3.4602366757	-0.0311755919	2.8291715681
C	4.1337704991	-0.6941636296	-0.1574796633
H	-3.5210891813	0.5353025775	-3.6632467580
H	-3.8127717081	-0.8669061060	-2.5901218900
H	-3.9610562567	0.7816395649	-1.9547137858
H	1.8738566455	1.6435432349	-2.7407546315
H	1.1472408334	1.0535202679	-4.2612117053
H	2.0541621015	-0.0596345286	-3.2084319464
H	-2.6282185900	-1.8965655328	2.1443669636
H	-0.8018154009	0.0595448599	3.6518431671
H	-0.3108933951	-1.5199915570	3.0103901837
H	-1.5026130703	-1.4233561984	4.3243279044
H	2.8091487593	-0.0471667496	1.4123160423
H	4.1868491674	2.1037592523	-0.2947246218
H	3.1173947819	2.4048900013	1.0879485750

H	4.6864748360	1.6192748882	1.3341374485
H	-3.1364375530	0.9954873605	3.0209340085
H	-4.2405582339	-0.0213502546	2.0664335559
H	-3.8903477806	-0.4332065370	3.7507711479
H	4.5060075385	-0.3430296691	-1.1239852490
H	3.6269252661	-1.6503729588	-0.2959919234
H	4.9925410075	-0.8589399345	0.4991271827

LUMO+2 (-0.17 eV)

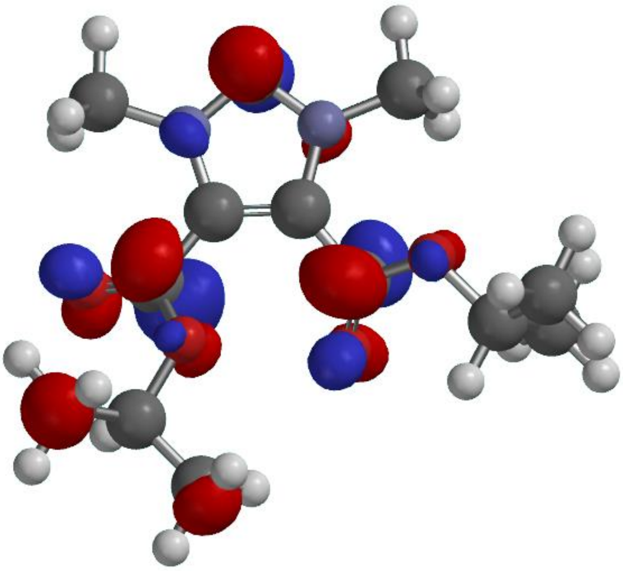

LUMO+1 (-0.43 eV)

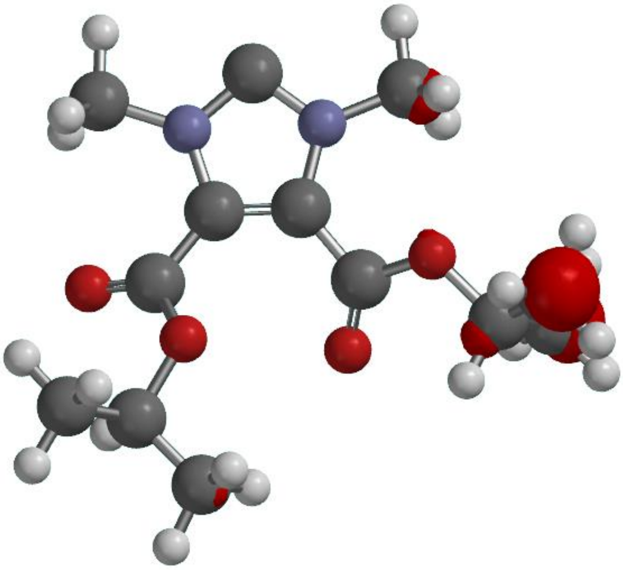

LUMO (-1.73 eV)

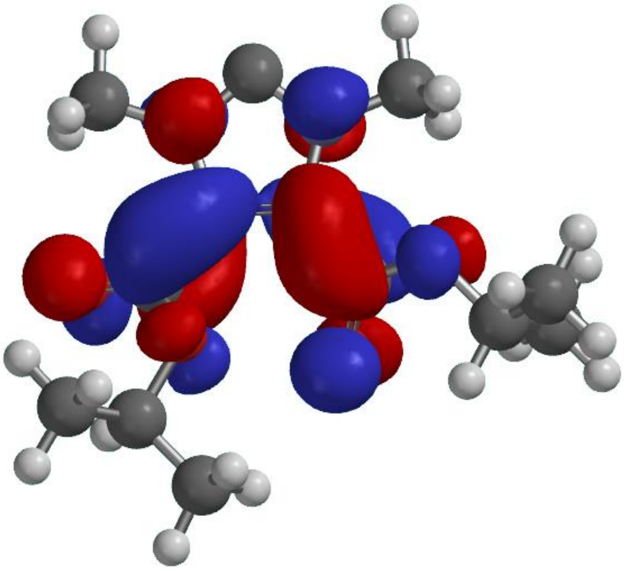

HOMO (-6.20 eV)

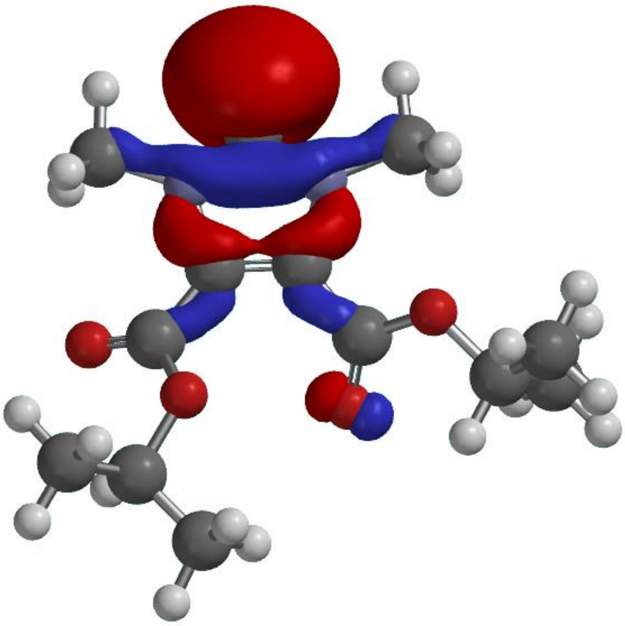

HOMO-1 (-6.77 eV)

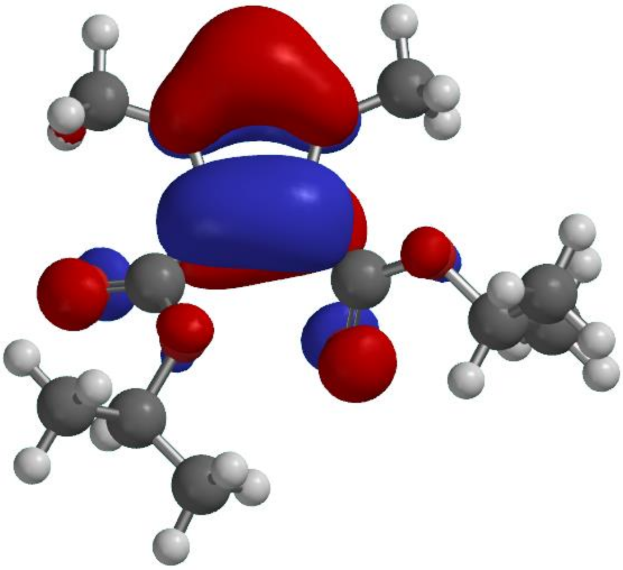

HOMO-2 (-7.89 eV)

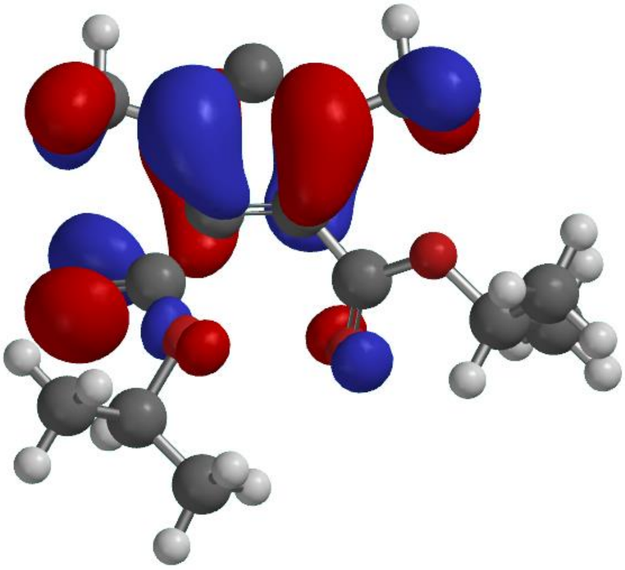

HOMO-3 (-8.03 eV)

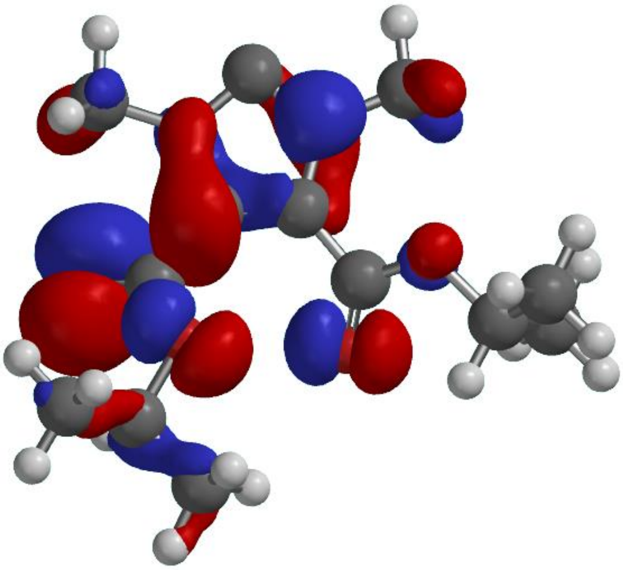

HOMO-4 (-8.14 eV)

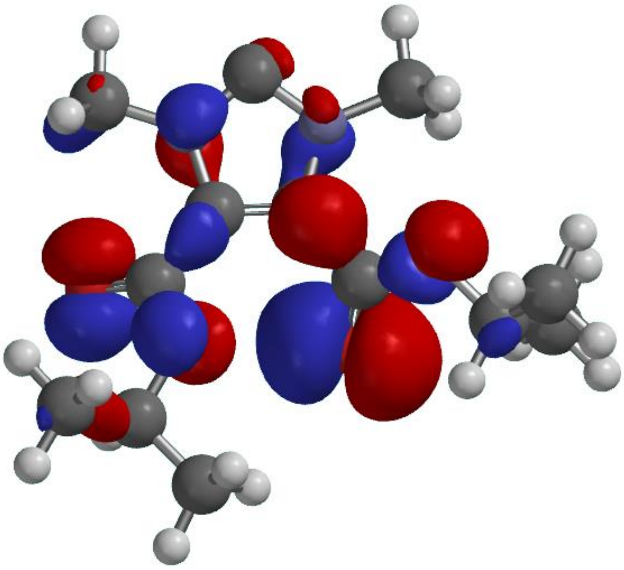

Calculation of the carbene of 16a

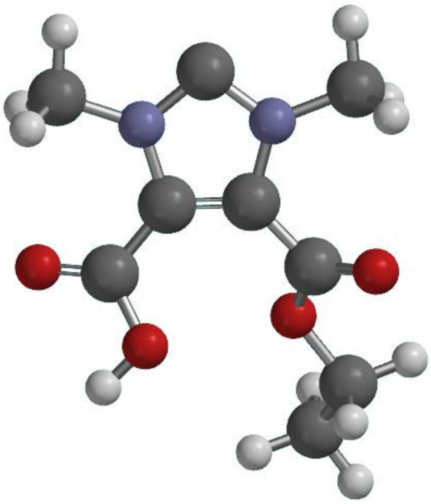

Charge: 0

Number of imaginary frequencies: 0

E: -760.7838671699 hartrees

Coordinates (Angstroem)

ATOM	X	Y	Z
C	0.2395493336	1.0135629333	-2.7359443668
N	0.0766101357	-0.3234685357	-2.5403156959
C	-0.2123934866	-0.6380968958	-1.2127802537
C	-0.2235958927	0.5603015735	-0.5382069920
N	0.0428441028	1.5339401987	-1.4835931365
C	0.1609536584	2.9677540732	-1.2187072449
C	0.1585823690	-1.2721822012	-3.6524618574
C	-0.4848865304	-2.0061761932	-0.7573489402
C	-0.4595548560	0.8898298624	0.8896002506
O	-1.1374178398	1.8240795572	1.2528697371
O	0.2029323446	0.0667169396	1.7114175926
C	-0.0168952981	0.2590521437	3.1346433161
C	0.8502144595	-0.7414144803	3.8693453938
O	-0.1276967505	-3.0092672281	-1.3335419006
O	-1.2240777199	-2.0457441565	0.3761720427
H	0.4159445634	3.4380039965	-2.1651554522
H	-0.7785461348	3.3642325482	-0.8363015612
H	0.9504992938	3.1597466256	-0.4893328269
H	0.9585968828	-1.9915754460	-3.4851061360
H	0.3552004080	-0.6817360912	-4.5441025937
H	-0.7807414479	-1.8152373851	-3.7655264648
H	0.2369465479	1.2900769368	3.3893881285
H	-1.0801584503	0.1136922099	3.3395575519
H	1.9076749014	-0.5813509368	3.6479189259
H	0.7047130921	-0.6283608636	4.9469554291
H	0.5894628757	-1.7651765820	3.5930777387
H	-1.3247605618	-2.9812026031	0.6074793159

LUMO+2 (-0.36 eV)

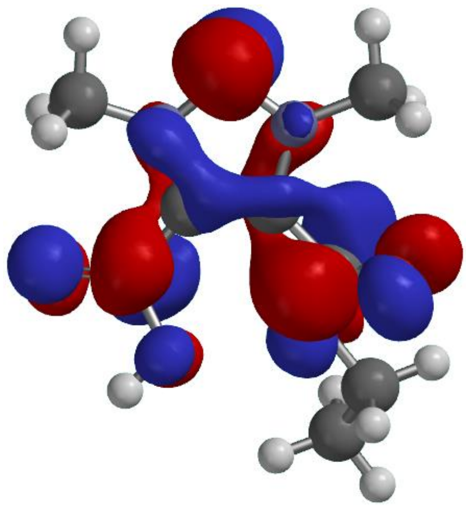

LUMO+1 (-0.54 eV)

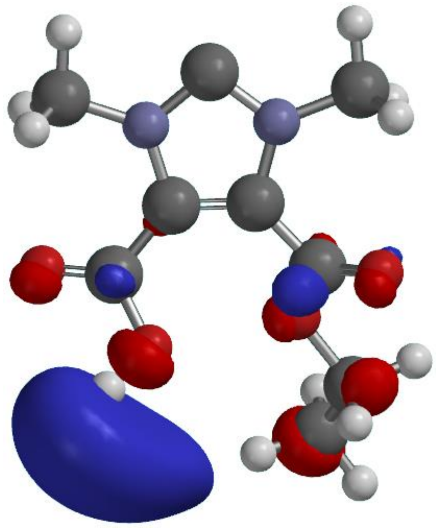

LUMO (-2.02 eV)

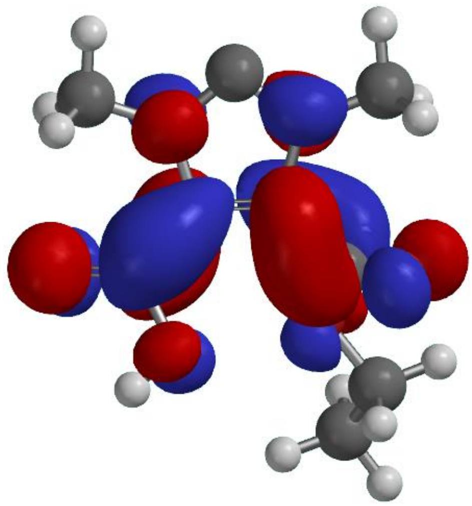

HOMO (-6.37 eV)

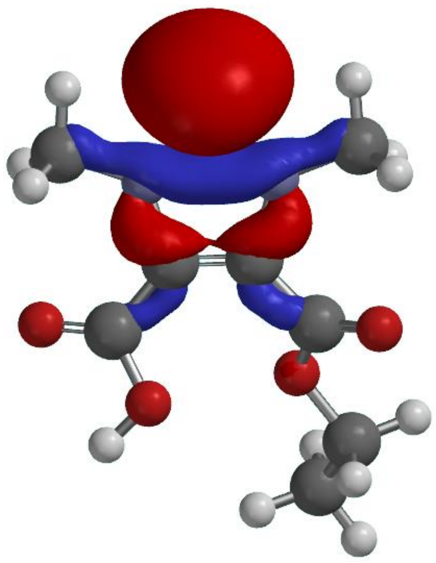

HOMO-1 (-7.02 eV)

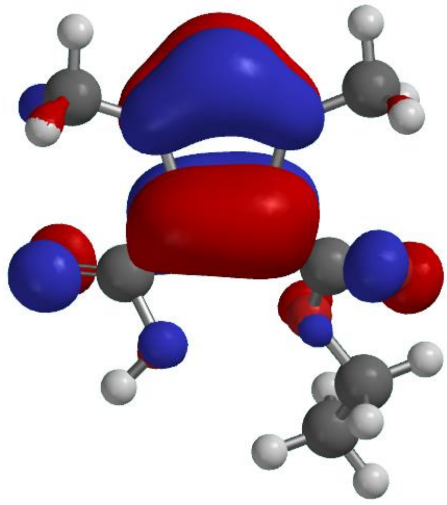

HOMO-2 (-8.07 eV)

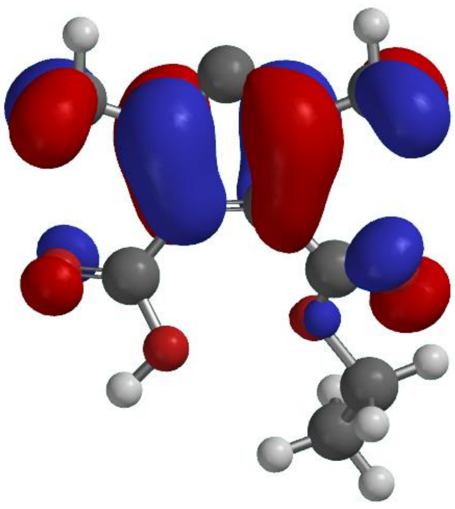

HOMO-3 (-8.35 eV)

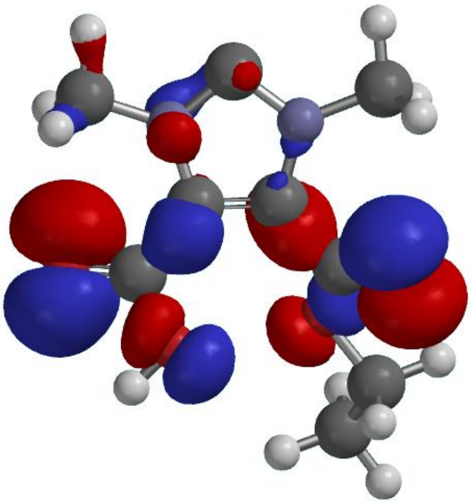

HOMO-4 (-8.38 eV)

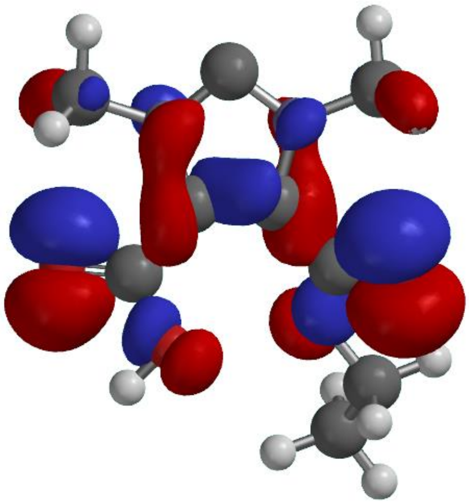

Calculation of the carbene of 16b

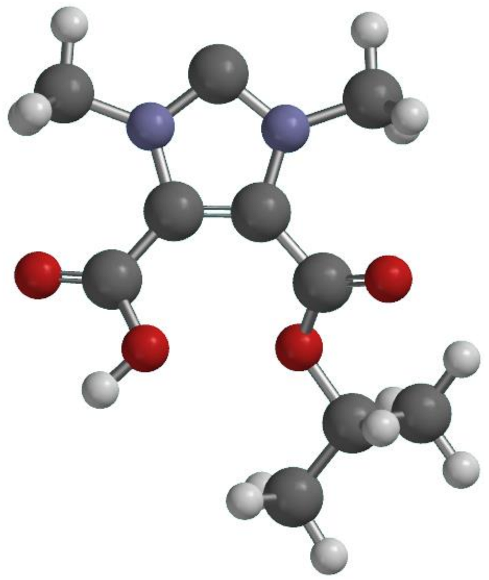

Charge: 0

Number of imaginary frequencies: 0

E: -800.1122624798 hartrees

Coordinates (Angstroem)

ATOM	X	Y	Z
C	-0.3916719582	0.2030331522	-3.2146830358
N	0.9020536522	0.1966006205	-2.7919776906
C	1.0196455877	-0.1046370356	-1.4355536807
C	-0.2649405796	-0.2886556797	-0.9798513876
N	-1.0888109959	-0.1038154603	-2.0759282890
C	-2.5503661331	-0.1628810591	-2.0640034063
C	2.0074936179	0.4378660953	-3.7208141872
C	2.3159356014	-0.2229081296	-0.7577509119
C	-0.8031438291	-0.6144552807	0.3653938439
O	-1.7048891900	-1.4035558054	0.5407191039
O	-0.2065661094	0.0978520348	1.3276578911
C	-0.5939108437	-0.1699353343	2.7171954363
C	0.6367730360	0.1279511262	3.5576486910
O	3.3411621305	0.2977518313	-1.1369756832
O	2.2693784228	-1.0197574821	0.3355042812
C	-1.8002252725	0.6876760534	3.0760513922
H	-2.9592614757	0.5836245616	-1.3797810002
H	-2.8772095761	0.0551090177	-3.0777866911
H	-2.8907681850	-1.1504790942	-1.7561899878
H	1.5573485247	0.5871730907	-4.6992615875
H	2.6821400351	-0.4189933189	-3.7493022361
H	2.5752288080	1.3180683362	-3.4232215186
H	-0.8577098052	-1.2272046682	2.7780244405
H	0.9356224426	1.1740928145	3.4527118022
H	1.4733223945	-0.5047268486	3.2561778883
H	0.4204114522	-0.0648394807	4.6115473864
H	3.1585565000	-1.0099043025	0.7202528317
H	-1.5626974235	1.7496214064	2.9699703539

H	-2.6537486521	0.4480544009	2.4400627118
H	-2.0891521763	0.5022744384	4.1141632396

LUMO+2 (-0.32 eV)

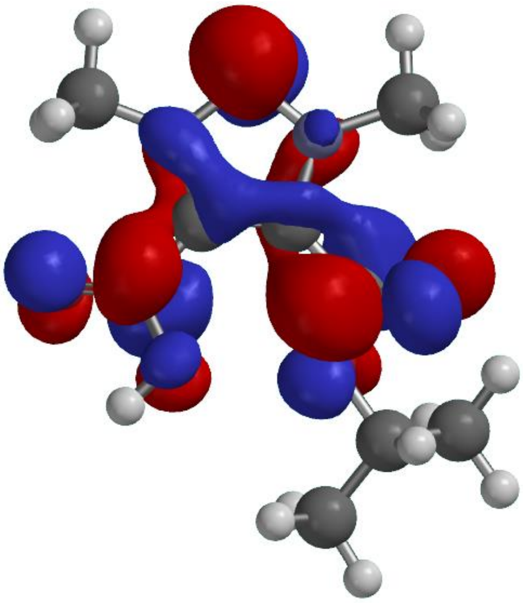

LUMO+1 (-0.53 eV)

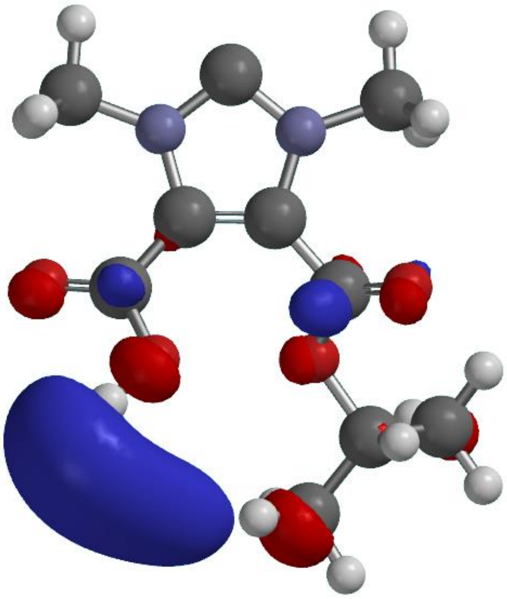

LUMO (-2.00 eV)

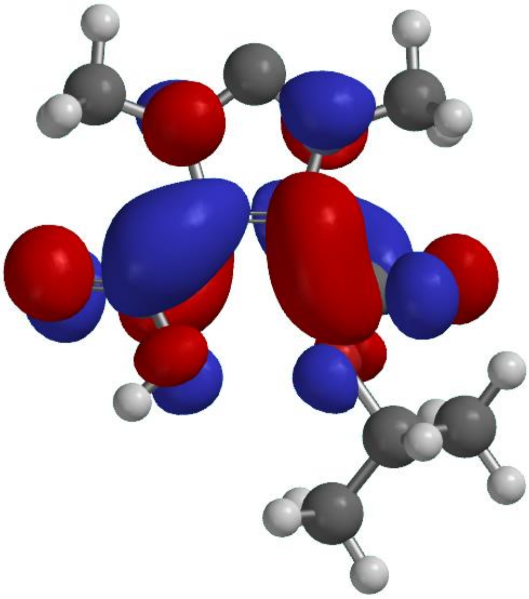

HOMO (-6.35 eV)

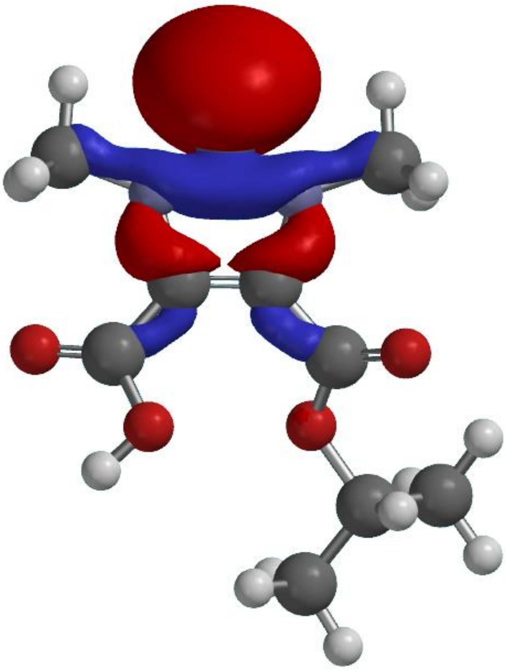

HOMO-1 (-7.00 eV)

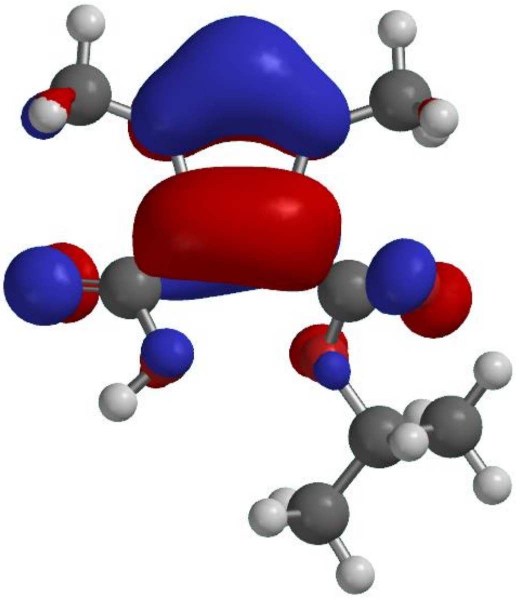

HOMO-2 (-8.05 eV)

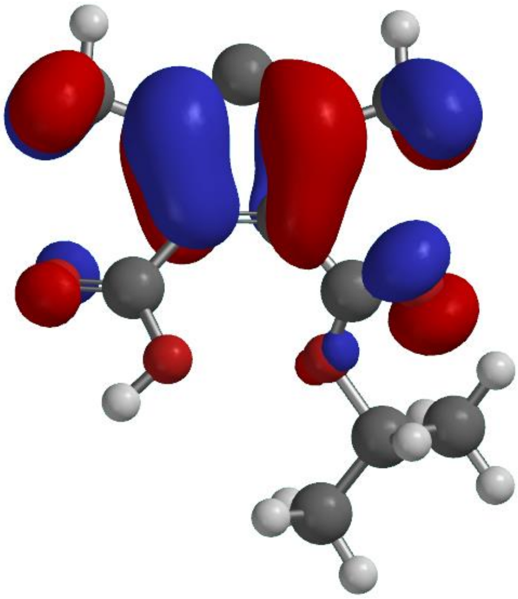

HOMO-3 (-8.29 eV)

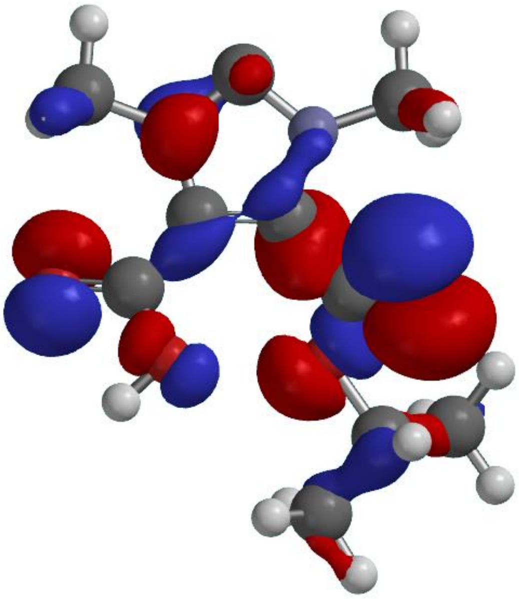

HOMO-4 (-8.36 eV)

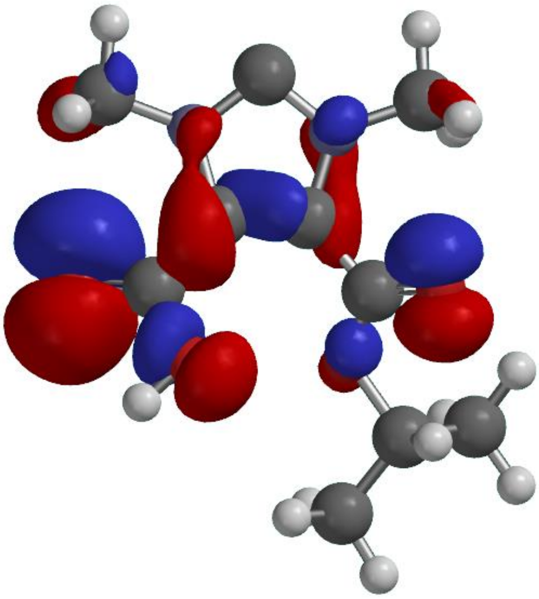

Calculation of the carbene of 17a

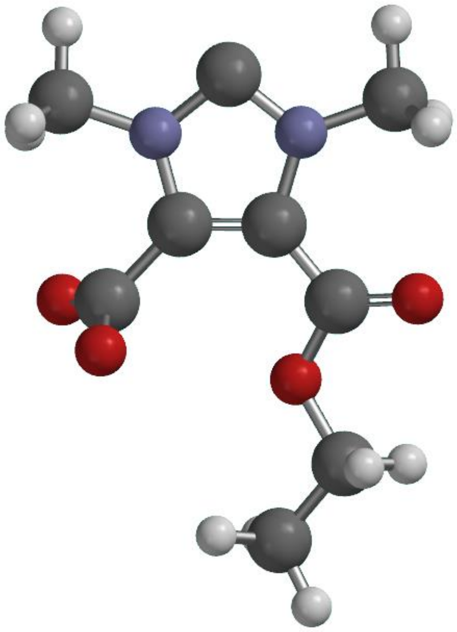

Charge: -1

Number of imaginary frequencies: 0

E: -760.2516044815 hartrees

Coordinates (Angstroem)

ATOM	X	Y	Z
N	-0.0212361490	-2.4500842809	-0.0939786325
C	-0.0809706918	-1.1606229518	0.3949182655
C	0.0657480121	-0.3511717271	-0.7105496948
C	0.0265496584	1.0941930919	-0.8095392635
C	-0.3029460251	-0.8746366920	1.8934802016
O	-0.1257895590	1.7408085270	-1.8371387160
O	-1.4064123601	-0.3751460469	2.1825617681
O	0.6475281269	-1.2400696666	2.6184045502
O	0.1810978607	1.6749927101	0.4006443250
C	-0.0091665162	3.4421922909	1.9697278144
C	-0.0993160305	3.0781462249	0.4989760045
C	-0.1380564208	-3.6342045176	0.7465657150
H	0.9994987668	3.2650622227	2.3504223949
H	-0.2555885673	4.4998958042	2.1095576877
H	-0.7010770983	2.8301757722	2.5514911629
H	0.6181563231	3.6376852749	-0.1099651688
H	-1.0977227752	3.2706774953	0.0954332624
H	0.0704184563	-4.5005092086	0.1209797328
H	-1.1479071850	-3.7117984595	1.1590157428
H	0.5690630079	-3.5660906005	1.5739737235
N	0.1972631468	-1.2176358985	-1.8192135726
C	0.1540977573	-2.5209279740	-1.4589967621
C	0.4195825391	-0.8048190955	-3.1970848064
H	1.3351245246	-0.2141842777	-3.2834409032
H	-0.4083700941	-0.1952570644	-3.5607448053
H	0.5104312925	-1.7166709524	-3.7855000262

LUMO+2 (+2.40 eV)

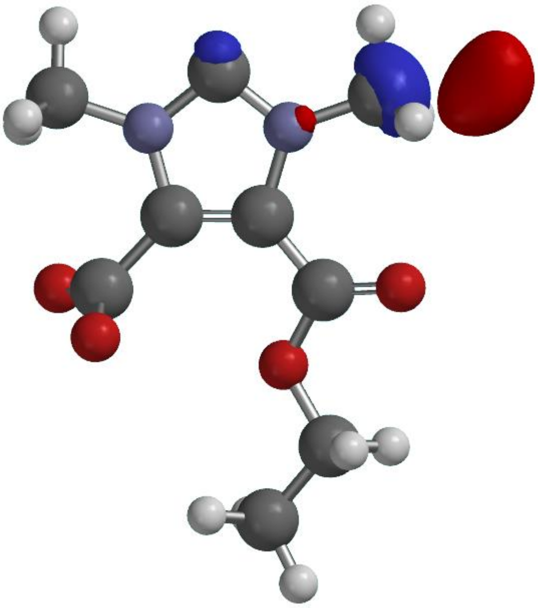

LUMO+1 (+2.34 eV)

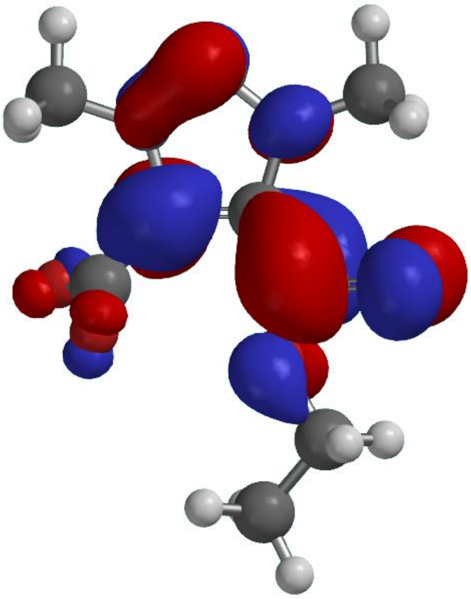

LUMO (+2.13 eV)

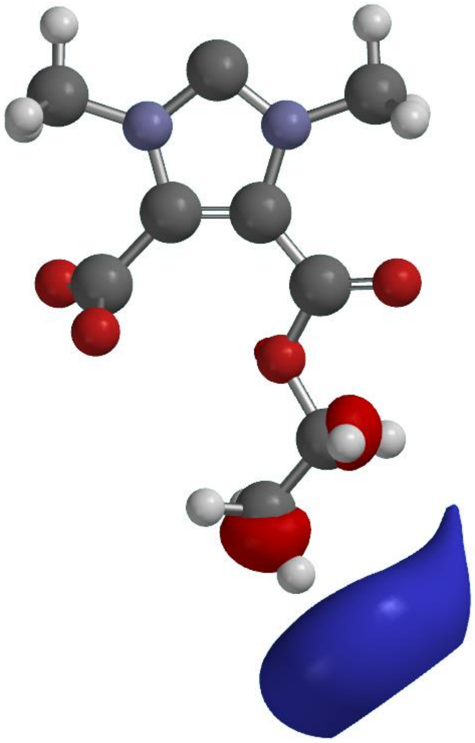

HOMO (-2.19 eV)

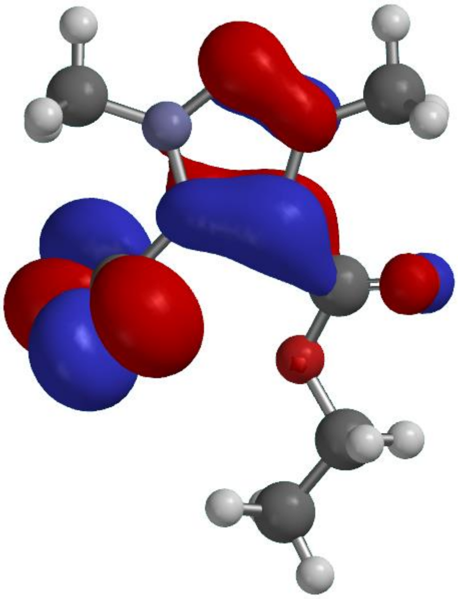

HOMO-1 (-2.48 eV)

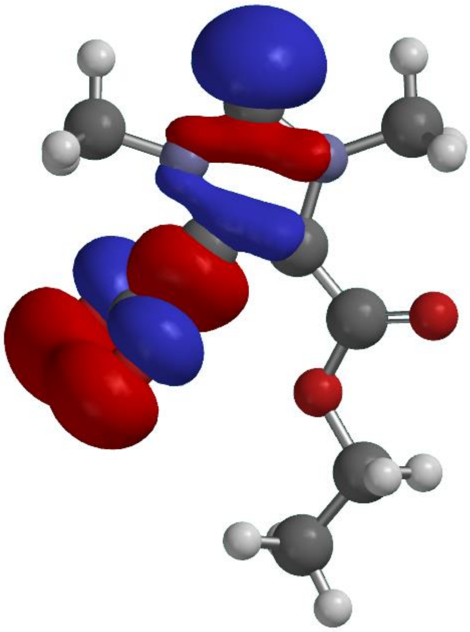

HOMO-2 (-2.79 eV)

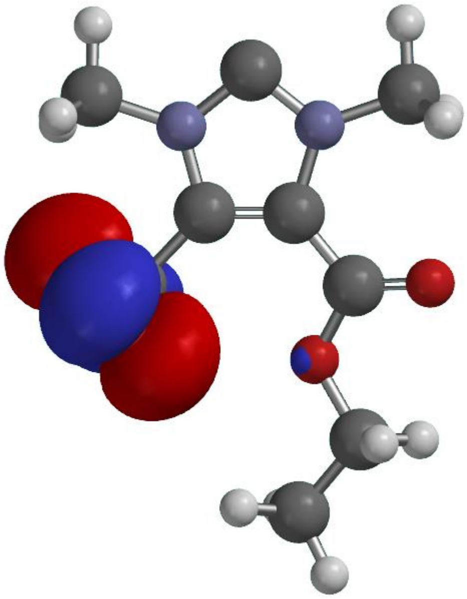

HOMO-3 (-3.07 eV)

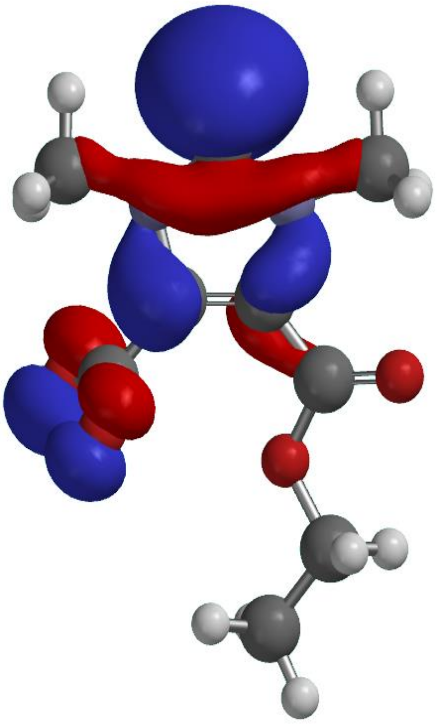

HOMO-4 (-3.35 eV)

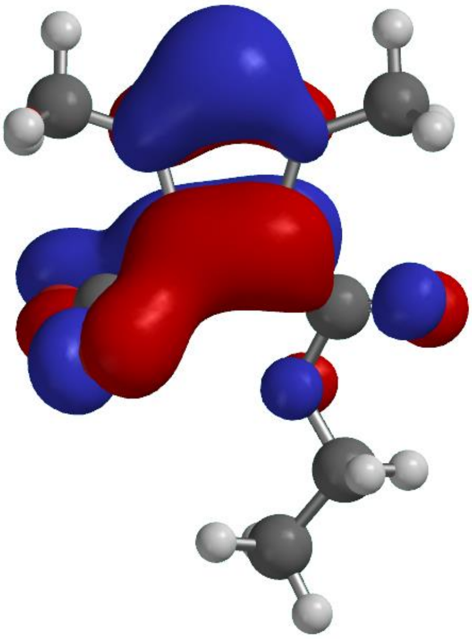

Calculation of the carbene of 17b

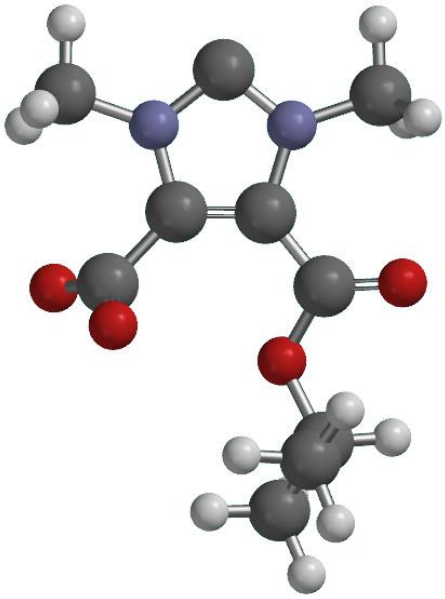

Charge: -1

Number of imaginary frequencies: 0

E: -790.5802357842 hartrees

Coordinates (Angstroem)

ATOM	X	Y	Z
C	0.3314779445	1.1197609132	-2.8252407063
C	0.3986748902	1.2018551578	-2.9999425140
N	0.1970046642	-0.1434240721	-2.7789871975
C	0.0946509071	-0.4796655015	-1.4436776795
C	0.2422782826	0.7087925781	-0.7623637636
N	0.4121448082	1.7082653579	-1.7452630695
C	0.6540312497	3.1193649398	-1.4841717890
C	0.0942058810	-1.1107423117	-3.8633584356
C	-0.1768706314	-1.9273421461	-0.9897635954
C	0.1482096368	0.9683237692	0.6615602131
O	-0.0610966392	2.0572383538	1.1786499797
O	0.3302875258	-0.1674196854	1.3719472465
O	-1.2860032325	-2.1185120313	-0.4543333719
O	0.7434484327	-2.7241549878	-1.2721777009
C	-0.1011646461	-0.2001859287	2.7524568774
C	0.6925857759	-1.3264737172	3.4001307854
H	0.7566010358	3.6043646429	-2.4538335957
H	-0.1697661836	3.5574125474	-0.9200575869
H	1.5708593081	3.2567934450	-0.9042748216
H	0.7975271408	-1.9266469710	-3.6943517071
H	0.3157112622	-0.5846283623	-4.7905095692
H	-0.9154982017	-1.5283869835	-3.9087236392
H	0.1498411383	0.7607226131	3.2099477722
H	1.7654411851	-1.1289525535	3.3343530181
H	0.4198345650	-1.4226932839	4.4557472552
H	0.4868619975	-2.2722309346	2.8930013191
C	-1.6100198738	-0.4296185382	2.7955450808
H	-2.1405102575	0.4214364548	2.3629190757
H	-1.9450275016	-0.5517886429	3.8313102548
H	-1.8642425196	-1.3217032084	2.2182211583

LUMO+2 (+2.39 eV)

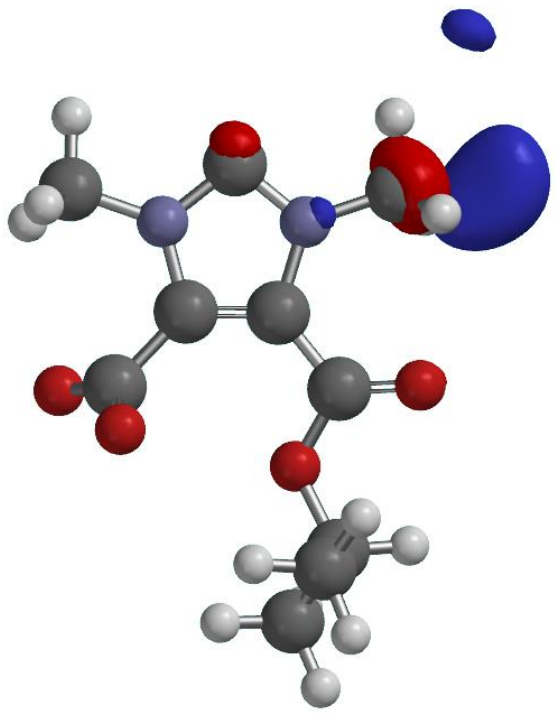

LUMO+1 (+2.31 eV)

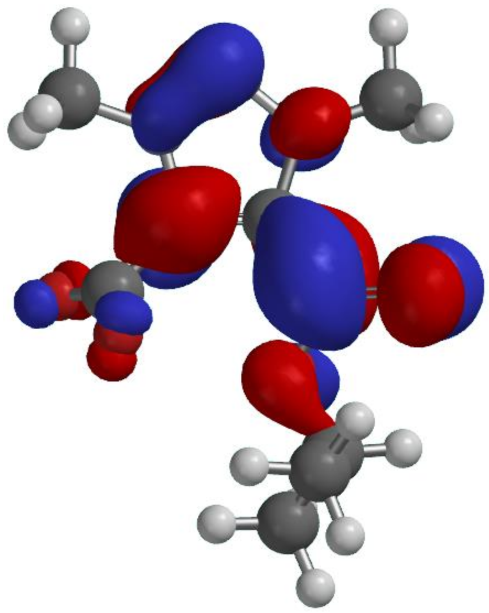

LUMO (+2.07 eV)

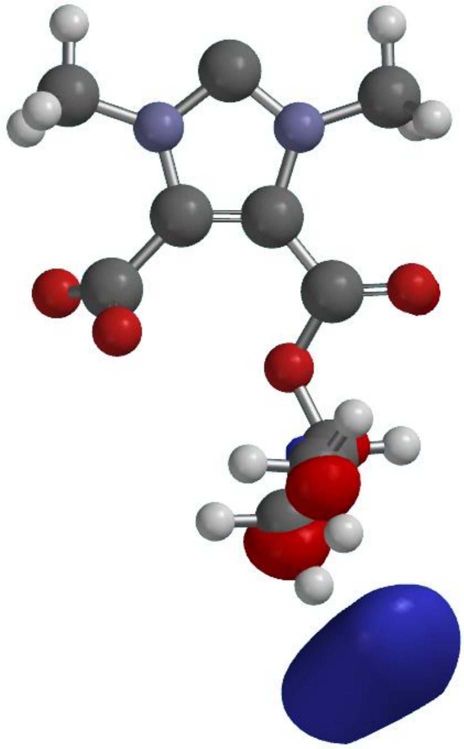

HOMO (-2.22 eV)

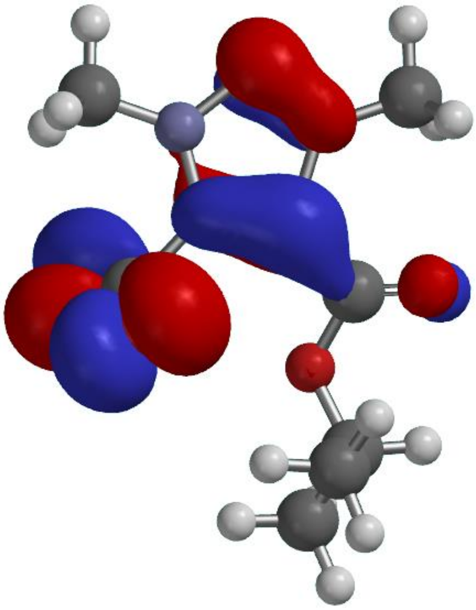

HOMO-1 (-2.51 eV)

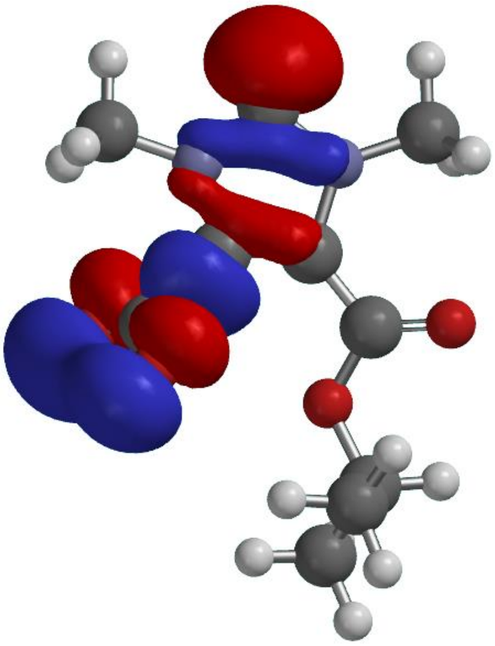

HOMO-2 (-2.82 eV)

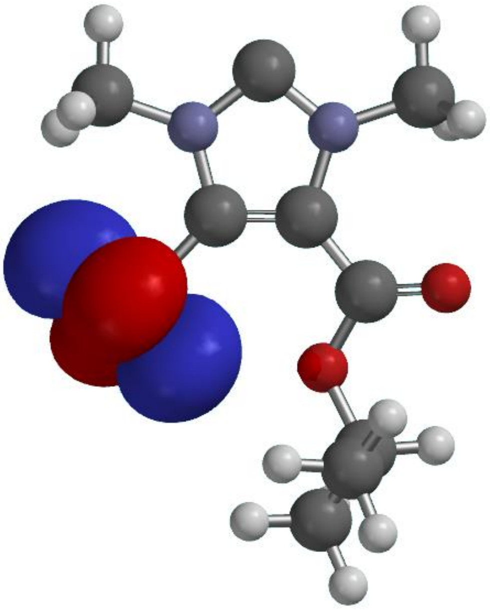

HOMO-3 (-3.08 eV)

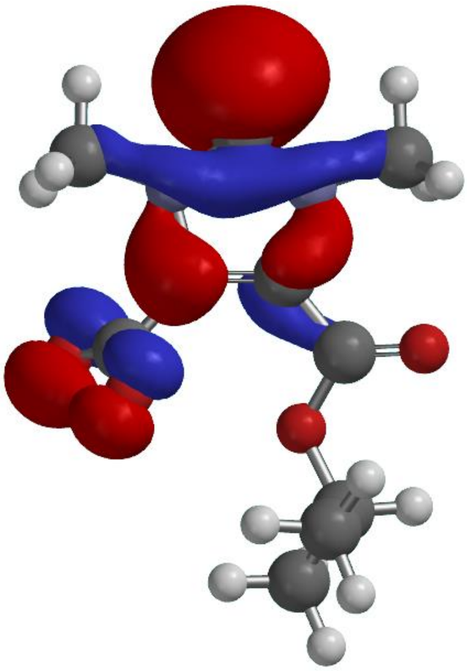

HOMO-4 (-3.36 eV)

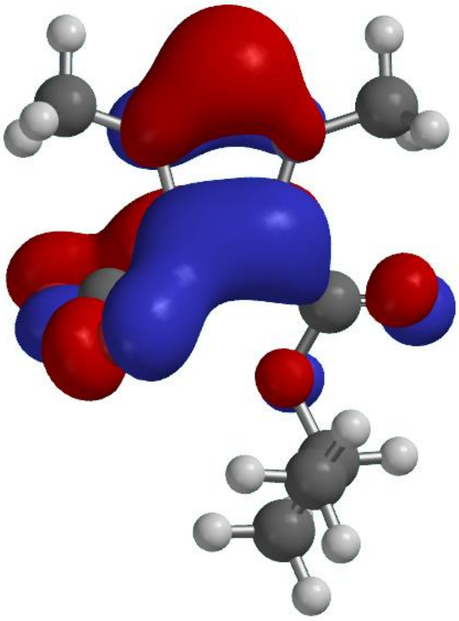

Supplement: Supplementary file 1 — jo4c02581_si_001.pdf [file jo4c02581_si_001.pdf]
